# Study on the effect of heat treatment on amethyst color and the cause of coloration

**DOI:** 10.1038/s41598-020-71786-1

**Published:** 2020-09-10

**Authors:** Renping Cheng, Ying Guo

**Affiliations:** Department of Gemmology, China University of Geosciences, Beijing, 100083 China

**Keywords:** Materials science, Materials for optics, Physics, Optical physics, Optics and photonics, Applied optics, Optical materials and structures, Chemistry, Materials chemistry, Physical chemistry

## Abstract

The effect of heat treatment on amethyst color was studied from a new perspective of chromaticity of gemstones and the cause of amethyst coloration was discussed based on the results of X-ray diffraction, ultraviolet–visible spectroscopy. The results show that the amethyst color has no significant relationship with cell parameters but the crystallinity index decreases as temperature rises. The absorption band at 545 nm in the UV–visible spectrum can be related to a charge-transfer transition of Fe^3+^ and O^2−^, which has a significant relationship with amethyst lightness and chroma. The color at different temperatures can be divided into three stages: The amethyst stage with temperature below 420 °C, the prasiolite stage with temperature between 420 and 440 °C where the color center is the most unstable, the citrine stage with temperature above 440 °C. The color change degree of heated amethyst is related to its initial color. When the initial color is darker, the color difference of heated amethyst is larger, and the easier it is to change the color after heat treatment. A more appropriate heating temperature to obtain citrine by heating amethyst is about 560 °C.

## Introduction

Amethyst is a kind of α-quartz with beautiful violet color. It is widely distributed in the crust and the origins are all over the world, such as Brazil, Uruguay, Canada, Sri Lanka^1^, Rwanda^2^, Morocco^3^ and Arizona^4^, etc. When the quartz contains impurities such as iron, aluminum and titanium, it will make quartz appear rich in color^5^. The colors of amethyst^6,7^, citrine^8^ and prasiolite^7,9,10^ are all related to iron. It was postulated that amethyst color can be related to the replacing Si^4+^ with Fe^3+^, and alkali metal ions (Li^+^, Na^+^ or H^+^) are introduced to maintain the charge balance^11^. In addition, holes created during quartz irradiation can also act as charge compensators^12^.

Many scholars have studied the enhancement methods of amethyst to discuss the mechanism of the coloration^7,13,14^. Amethyst could become citrine when the temperature is over 500 °C^10^, and the violet and green color could be appeared by irradiating the colorless quartz^14^. The color change of amethyst after heating and irradiation is related to the “color center activated by iron”^7,15^. The study of UV–visible spectroscopy of amethyst also provides support for the study of the existing form of iron element in amethyst. The main absorption bands of amethyst in UV–Vis spectra are located at 950 nm, 540 nm and 360 nm, which is considered to be related to the transition of Fe^4+^ in tetrahedral coordination^9,12,16,17^, and these absorption bands show different degrees of changes after irradiation or heating.

In order to obtain and analyze the color of minerals, the color grading method of colored diamond was proposed in 1994^18^. In this method, the colored diamonds were graded in standard illumination box and a 6504 K fluorescent lamp was used as lighting source. Then the International Commission on Illumination (CIE) 1976 L*a*b* uniform color space system has also been gradually applied to the mineral color evaluation and grading system, such as sapphire^19^, alexandrite^20,21^, color change garnet^22,23^, peridot^24,25^, tourmaline^26,27^, citrine^28^ and jadeite^29–31^. However, there are few studies on the color evaluation and analysis in the process of heat treatment of amethyst. The CIE 1976 L*a*b* uniform color space system is composed of colorimetric coordinates a*, b* and lightness (L*). Chroma (C*) and hue angle (h°) can be calculated by a* and b* as following:

1 $$ C_{{}}^{*} = \sqrt {(a^{*} )^{2} + (b^{*} )^{2} } $$

2 $$ h_{{}}^{o} = \arctan \frac{{b^{*} }}{{a^{*} }} $$

The CIE DE2000 (1:1:1) formula (formula 3)^32^ is used to calculate the color difference (ΔE_00_) of amethyst under different heating temperatures. ΔL*, ΔC* and ΔH*, represent the difference of lightness, chroma and hue angle respectively. R_T_ is a conversion function to reduce the interaction between chroma and hue in the blue area. S_L_, S_C_, and S_H_ are functions to calibrate the absence of visual uniformity of the CIE LAB formula. K_L_, K_C_, K_H_ are correction parameters of the experimental environment and K_L_ = 1, K_C_ = 1, K_H_ = 1.

3 $$ \Delta E_{00} = \left[ {\left( {\frac{{\Delta L^{*} }}{{K_{L} S_{L} }}} \right)^{2} + \left( {\frac{{\Delta C^{*} }}{{K_{C} S_{C} }}} \right)^{2} + \left( {\frac{{\Delta H^{*} }}{{K_{H} S_{H} }}} \right)^{2} + R_{T} \left( {\frac{{\Delta C^{*} }}{{K_{C} S_{C} }}} \right)^{2} \left( {\frac{{\Delta H^{*} }}{{K_{H} S_{H} }}} \right)^{2} } \right]^{\frac{1}{2}} $$

**Figure 1:**
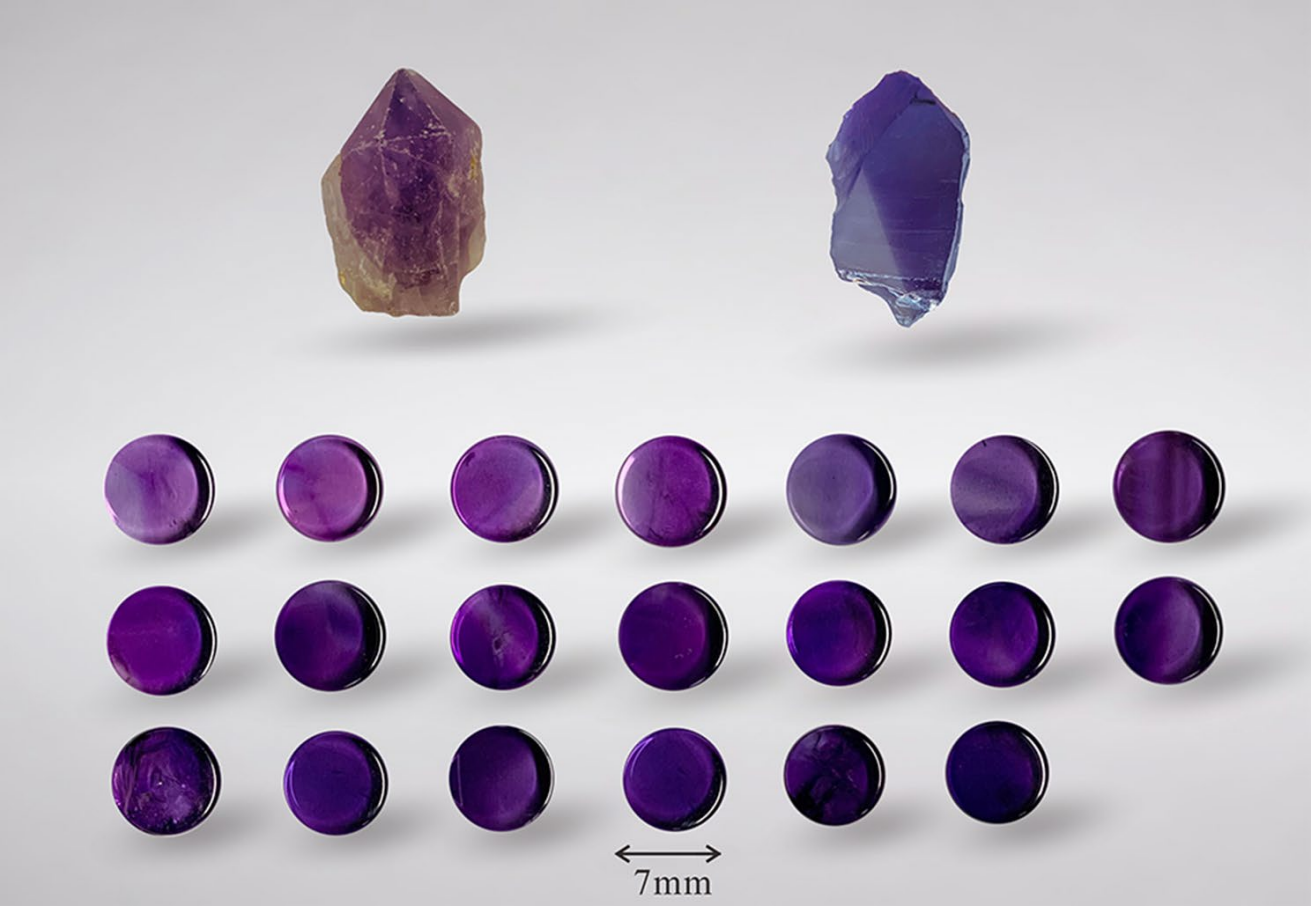
The picture of samples. Round flat samples which are used for color parameters testing have an average weight of 4.01 g and the raw stones at the top of the picture are used for XRD measurement. The lightness of these samples ranges from 21.99 to 53.47, the chroma ranges from 31.80 to 51.25 and the hue angle ranges from 312.1 to 322.5. The figure was taken and drawn by Renping Cheng using CorelDRAW Graphics Suite X8 2016 18.0.0.405 (https://www.corel.com).

We used 20 pieces of natural amethyst from South Africa (Fig. 1), whose colors display continuously from pink violet to blue violet. To get the color parameters effectively, all of them are cut into a polished round plane with a diameter of 7 mm and a thickness of 4 mm from different crystallographic orientations. There is no obvious inclusion in the inner part of the samples observed by naked eyes and some samples have straight or angular color bands. In this paper, the cause of amethyst coloration is discussed based on the results of the X-ray diffraction (XRD) and UV–Vis spectroscopy measurement of amethyst at different temperatures. Based on CIE1976 L*a*b* uniform color space system, the effect of heat treatment on amethyst color is studied and a more appropriate temperature range is defined to obtain the best color of citrine by heating amethyst.

## Results

### X-ray diffraction measurement

**Figure 2:**
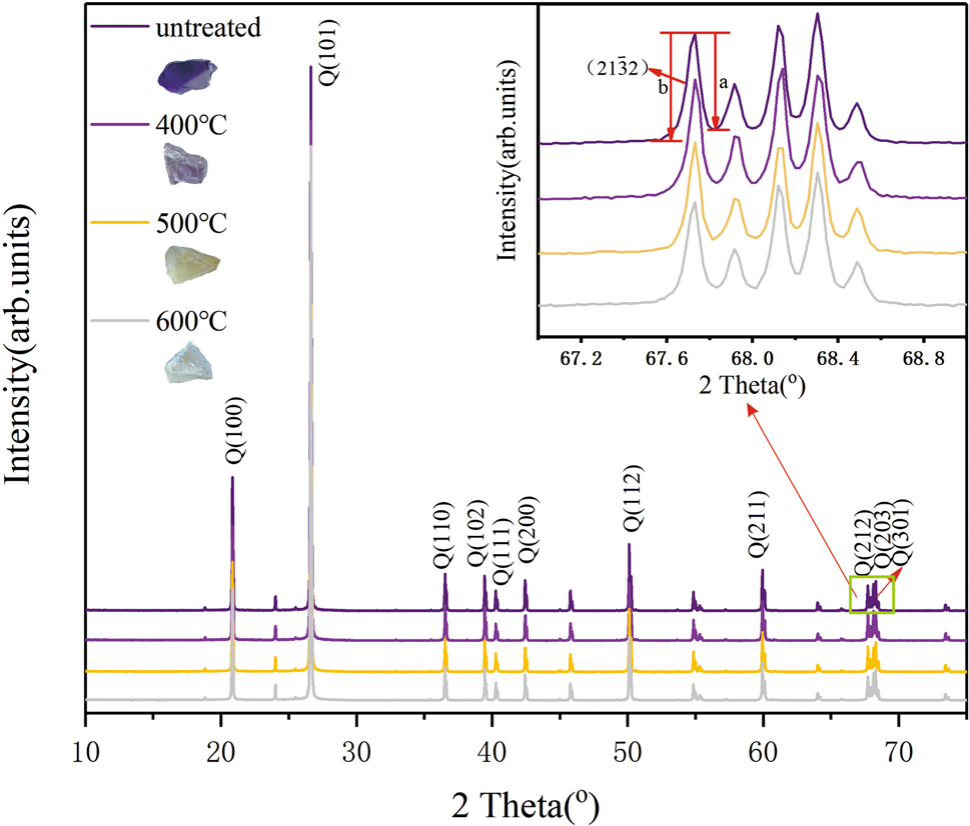
X-ray diffraction patterns of four samples under different temperature, the top right is the ${(}21\overline{3} 2)$ diffraction peak of quartz, a and b values of crystallinity index CI. The figure was drawn using the OriginPro 2017C Beta b9.4.1.268 (https://www.OriginLab.com).

When the temperature arrives 573 °C, the amethyst will change from α-quartz to β-quartz and the color of amethyst cannot be recovered by γ ray irradiation^33^. It may indicate that when the heating temperature is higher than the phase transition temperature, the color center of amethyst may be permanently destroyed^34^. In order to find out whether the color of amethyst at different heating temperatures is related to phase transition and crystal structure, four samples were cut from the same raw stone. One of them was not heat treated, and the other three were heated to 400 °C, 500 °C, and 600 °C. After being heated, they became light violet, yellow and milky white respectively. Then we tested the samples by XRD measurement and the pattern is as shown in the Fig. 2.

Four samples are all quartz, which matched the standard card PDF Card-46–1,045. It can be concluded that the crystal plane index, d-spacing (Å) and the unit cell parameters of samples heated at different temperatures are not significantly changed. As shown in Table 1, the cell volumes of the samples are decreasing to 111.61 (Å^3^) as the temperature rises to 500 °C. When it reaches 600 °C, the cell volume begins to increase to 115.81 (Å^3^) and the increase of cell volume could be related to the crystallization of nuclei of ferrous silicate^34^.

**Table 1:** XRD analysis data of samples under different heat treatment temperatures.

Temperature	Unheated		400 °C		500 °C		600 °C	
Color	Violet		Light violet		Yellow		Milk white	
[hkl]	d-spacing (Å)	2θ	d-spacing (Å)	2θ	d-spacing (Å)	2θ	d-spacing (Å)	2θ
[100]	4.26	20.85	4.26	20.86	4.26	20.86	4.26	20.85
[101]	3.34	26.63	3.34	26.64	3.34	26.64	3.34	26.63
[110]	2.46	36.53	2.46	36.54	2.46	36.54	2.46	36.53
[102]	2.28	39.45	2.28	39.46	2.28	39.46	2.28	39.45
[200]	2.13	42.44	2.13	42.44	2.13	42.44	–	–
[112]	1.82	50.12	1.82	50.13	1.82	50.13	1.82	50.12
[211]	1.54	59.94	1.54	59.95	1.54	59.94	1.54	59.94
[212]	1.38	67.72	1.38	67.73	1.38	67.73	–	–
[203]	1.38	68.12	1.38	68.13	1.38	68.13	1.38	68.13
[301]	1.37	68.30	1.37	68.31	1.37	68.31	1.37	68.30
a = b Å	4.9686(3)		4.9217(1)		4.8861(2)		4.9615(3)	
c Å	5.4262(5)		5.4138(3)		5.3981(5)		5.4323(6)	
α	90°		90°		90°		90°	
β	90°		90°		90°		90°	
γ	120°		120°		120°		120°	
Cell volume Å^3^	116.01(1)		113.57(1)		111.61(1)		115.81(2)	
Crystallinity index(CI)	10.00		10.00		9.79		9.55	

The crystallinity index (CI) of samples slightly decreases from 10 to 9.55 with the increase of heat treatment temperature. The crystallinity index CI^35^ can be calculated by a and b values of the ${(}21\overline{3} 2{)}$ peak as follows (F is the scaling factor of 1.12):

4 $$ CI = \frac{10aF}{b} $$

When the temperature is above 600 °C (higher than the phase transition temperature of amethyst), the amethyst becomes milky and turns lighter. It can be related to the destruction of the S_1_ centers ([Fe^3+^O_4_/M_i_^+^]) and I centers (Fe^3+^ in an interstitial site)^34^ and the aggregation of water to form “water bubbles”. The diameter of the “water bubbles” is from 20 to 100 nm, which can cause Rayleigh scattering of light^36^.

### UV–Vis spectrum analysis

**Figure 3:**
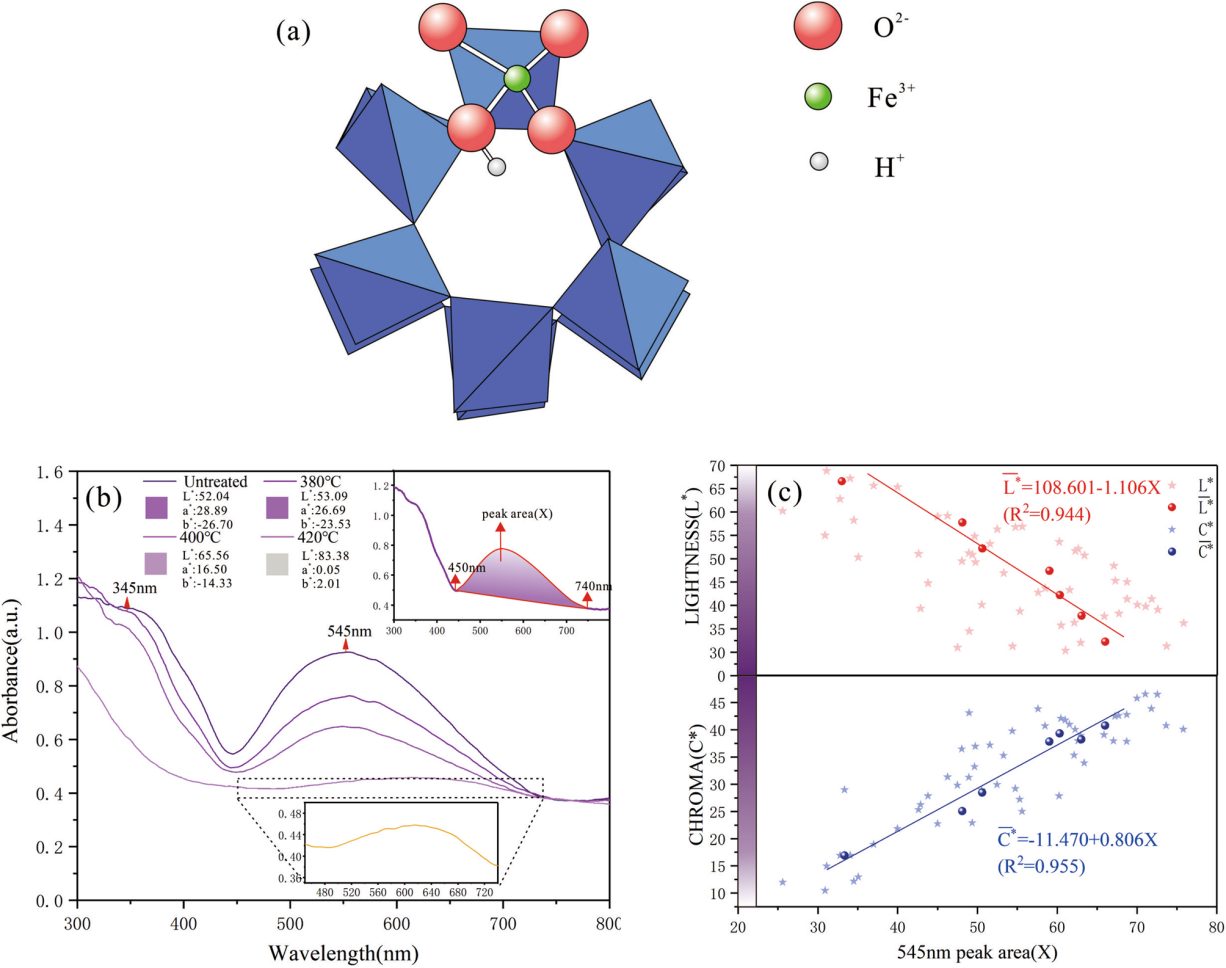
(**a**) Structural models of the Fe substitutional sites, obtained from DFT calculations^38^: the Fe^3+^, H^+^ are shown with the channels along the c-axis projection. In the model, Fe bonds three O ions at 1.81 Å and the fourth (bonded to the proton) at 2.06 Å. (**b**) The UV–Vis spectra of amethysts heated at different temperatures. The top right is the peak area (X) at 545 nm obtained by integrating from 450 to 740 nm. (c) The relationship between peak area (X) at 545 nm and lightness (L*) and chroma (C*). The larger the peak area (X) at 545 nm is, the darker the color of the amethyst. The figures were drawn using the OriginPro 2017C Beta b9.4.1.268 (https://www.OriginLab.com.) and CorelDRAW Graphics Suite X8 2016 18.0.0.405 (https://www.corel.com).

The absorption bands at different wavelengths of amethyst UV–Vis spectra correspond to the relevant color centers^12,16,37^. Therefore, the samples heated at different temperatures were tested by UV–Vis spectroscopy. The UV–Vis spectra of amethysts had a broad absorption bands at 545 nm and 345 nm. As the temperature rises, the violet color gradually became lighter and the absorption bands at 545 nm and 345 nm were gradually weakened. The color of amethyst became almost colorless and the absorption bands at 545 nm almost disappeared at 420 °C (Fig. 3b).

Lehmann^9^ believed that amethyst color is related to the intensity of the optical absorption near 545 nm and ascribed the color to a charge-transfer transition of Fe^3+^ and O^2−^_._ Rossman^11^ reported that the violet coloration is observed only in samples where specific sites are occupied by Fe^3+^ ions. Czaja^7^ conducted Mössbauer spectroscopy measurements on quartz with radiation and heat treatment and the results showed that only Fe^3+^ was found in the prasiolite and amethyst. According to the result of the XANES evidences and DFT structural features, Di Benedetto^38^ also suggest that numerous local distortions are occurring as a consequence of the presence of Fe^3+^ variably compensated by H^+^, when Fe replaces Si in its tetrahedral site in amethyst. They provided the evidence for the existence of Fe^3+^-H^+^ complex structure in amethyst by calculation and proposed the structural model of the Fe substitutional sites (Fig. 3a).

According to Halliburton, the exposure of quartz to ionizing radiation may also lead to the creation of a hole (H^+^) in a non-bonding p orbital of an oxygen atom adjacent to substitutional aluminum^39^. Since H^+^ is a small and light ion, it can easily diffuses through the quartz lattice and may occupy an interstitial sites previously occupied by alkali ions ($M_{i}^{ + }$)^40^. The color center formation of could be related to the following mechanism^41^:

5 $$ \left[ {Al_{si} O_{4} /M_{i}^{ + } } \right]^{0} \mathop{\longrightarrow}\limits{\gamma }\left[ {Al_{si} O_{4} /H^{ + } } \right]^{0} + M_{i}^{ + } + e^{ - } $$

A similar centers process of Amethyst have been proposed when iron is substituting for silicon in the quartz lattice as reaction (6)^12,42^. The alkali ion released in reaction may eventually react with an electron, leading to the creation of an interstitial neutral atom as reaction (7)^14,41^:

6 $$ \left[ {Fe_{si} O_{4} /M_{i}^{ + } } \right]^{0} \mathop{\longrightarrow}\limits{\gamma }\left[ {Fe_{si} O_{4} /H^{ + } } \right]^{0} + M_{i}^{ + } + e^{ - } $$

7 $$ M_{i}^{ + } + e^{ - } \to M_{i}^{0} $$

Citrine can be obtained by heating amethyst at a temperature of about 500 °C^10,40^. As for the cause of the color of citrine, During the heat treatment, the transformation of amethyst to citrine is related to the increase in the concentration of interstitial Fe^3+^ related defects and the precipitation of iron particles in the quartz lattice^17,34^_._ According to Stock and Lehmann, the size of these iron particles is about 100 nm^43^. The brown color is partly due to these inclusions and partly due to a shift of the charge-transfer band into the visible as S_1_ centers are converted to less constricted I centers and the charge-transfer is as following (Fe_i_ represents an Fe ion at an interstitial site)^34^:

8 $$ O^{2 - } + Fe_{i}^{3 + } \leftrightarrow O^{ - } + Fe_{i}^{2 + } $$

In order to find out whether the absorption band at 545 nm is related to the color of amethyst, we analyzed the color parameters and the peak area (X) of the absorption band at 545 nm which is obtained by heating the samples below 400 °C. To get the average peak area ($\overline{X}$) of each category, the peak area (X) is divided into 7 categories according to their initial lightness (L*) range from 30 to 70 (Table 2).

**Table 2:** The average peak area ($\overline{X}$) at 545 nm and average color parameters of 7 categories.

Categories	$\overline{X}$	$\overline{{L^{*} }}$	$\overline{{a^{*} }}$	$\overline{{b^{*} }}$	$\overline{{C^{*} }}$	$\overline{{h^{o} }}$	Simulated color
L_1_*(< 35)	66.00	32.26	28.35	− 29.31	40.78	314.05	
L_2_*(35–40)	63.02	37.81	27.40	− 26.67	38.23	315.77	
L_3_*(40–45)	60.31	42.21	28.36	− 27.25	39.33	316.14	
L_4_*(45–50)	59.01	47.41	27.84	− 25.52	37.76	317.49	
L_5_*(50–55)	50.60	52.21	20.32	− 19.80	28.37	315.74	
L_6_*(55–60)	48.10	57.73	18.21	− 17.06	24.95	316.88	
L_7_*(> 60)	36.03	66.57	14.03	− 12.17	18.57	319.05	

According to Fig. 3c, as the average peak area ($\overline{X}$) at 545 nm increases, the average lightness ($\overline{{L^{*} }}$) decreases and the average chroma ($\overline{{C^{*} }}$) increases simultaneously. The correlation (R^2^) between them is 0.944 and 0.955 respectively, which means the absorption band at 545 nm has a significant relationship with the color of amethyst. The larger of the peak area at 545 nm is, the darker the color of amethyst. However, the hue angle (h°) has no significant relationship with the peak area (X) at 545 nm, which is because the hue angle of amethyst has no obvious change when temperature is below 400 °C. The relationship between $\overline{{L^{*} }}$, $\overline{{C^{*} }}$ and $\overline{X}$ as follows:

9 $$  \overline{{L^{*} }} = 108.601 \pm 6.094SEM - 1.106 \pm 0.110SEM\overline{X} \quad {\text{(R}}^{{2}} { = 0}{\text{.944)}} $$

10 $$  \overline{{C^{*} }} = - 11.470 \pm 3.057SEM + 0.806 \pm 0.071SEM\overline{X} \quad {\text{(R}}^{2} { = 0}{\text{.955)}} $$

### The effect of heat treatment on amethyst color

**Figure 4:**
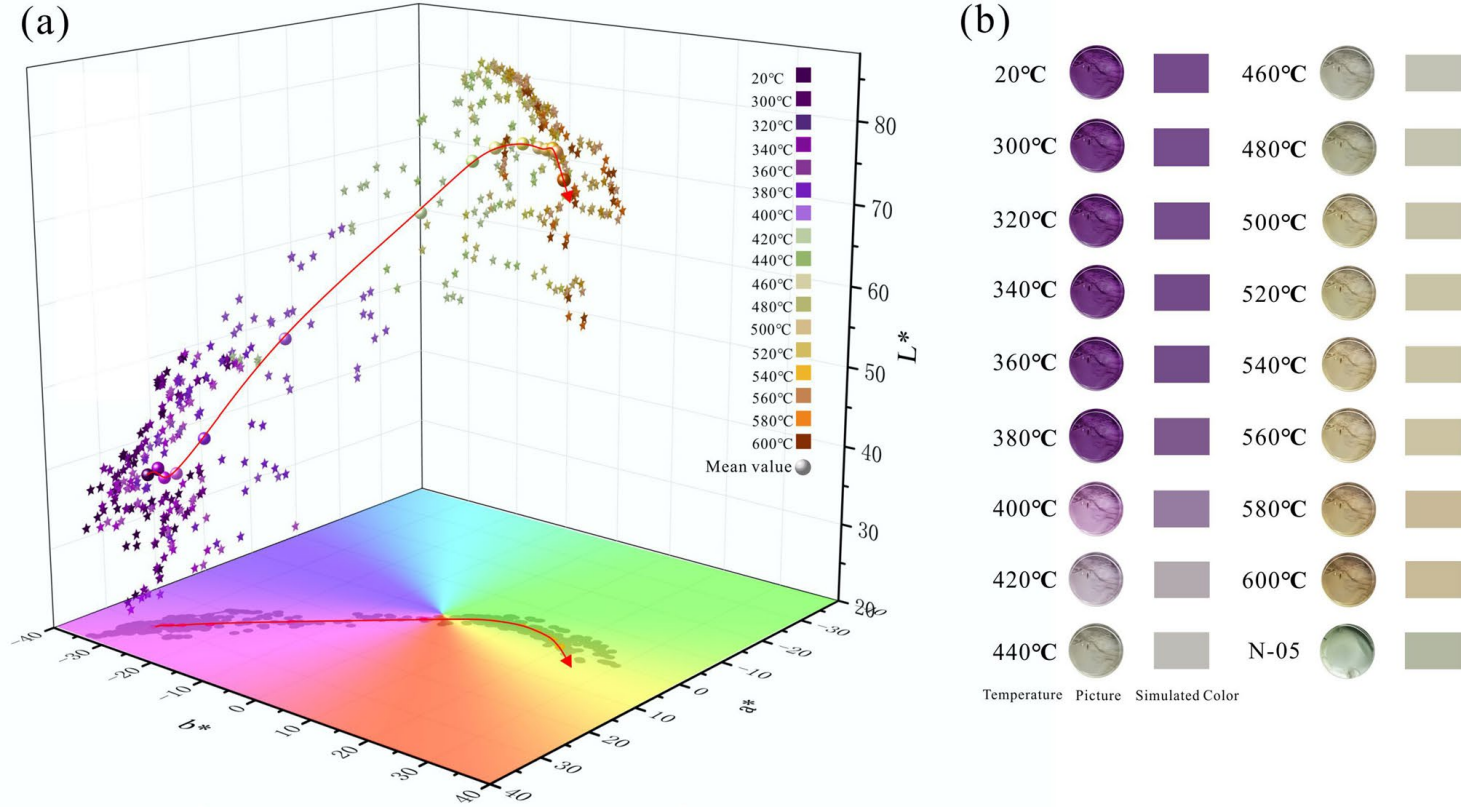
The color and distribution of amethyst at different temperatures (a) Color parameters of the amethyst at different temperature in CIE1976 L*a*b* uniform color space. (b) The pictures of amethyst in different temperatures and the simulated color. The figures were drawn using the OriginPro 2017C Beta b9.4.1.268 (https://www.OriginLab.com) and CorelDRAW Graphics Suite X8 2016 18.0.0.405 (https://www.corel.com).

The temperature of heat treatment has a great effect on amethyst color. Instead of observing by naked eye, we quantified the amethyst color based on CIE1976 L*a*b* uniform color space system. The distribution of the color parameters of the amethyst at different temperature in CIE1976 L*a*b* uniform color space is shown in the Fig. 4a. After heat treatment, the color of amethyst is stable and will not change obviously in dark conditions. The average color parameters and the range of a* (R_a_), b* (R_b_) and color difference (ΔE_00_) of the samples between current temperatures and the previous adjacent temperature is shown in Table 3.

**Table 3:** Mean value of color parameters, R_a_, R_b_ and color difference (ΔE_00_) at different temperatures.

Temperatures (°C)	$\overline{{L^{*} }}$	$\overline{{a^{*} }}$	$\overline{{b^{*} }}$	$\overline{{C^{*} }}$	$\overline{{h^{{_{0} }} }}$	Ra	R_b_	ΔE_00_
20	39.25 (δ = 8.50)	30.59 (δ = 4.59)	− 29.51 (δ = 2.86)	42.50	316.02	19.18	8.99	–
300	39.76 (δ = 7.99)	29.30 (δ = 4.25)	− 28.78 (δ = 2.45)	41.07	315.51	17.23	8.57	0.72
320	39.96 (δ = 8.07)	29.33 (δ = 4.29)	− 28.73 (δ = 2.55)	41.06	315.59	17.83	8.79	0.18
340	38.77 (δ = 8.54)	28.81 (δ = 4.33)	− 28.03 (δ = 2.86)	40.20	315.78	18.78	9.15	1.09
360	39.30 (δ = 8.27)	27.73 (δ = 4.90)	− 26.73 (δ = 3.25)	38.51	316.05	21.60	15.37	0.77
380	43.59 (δ = 7.11)	24.96 (δ = 5.11)	− 23.80 (δ = 3.31)	34.49	316.36	17.29	12.96	4.15
400	55.53 (δ = 7.77)	16.82 (δ = 6.69)	− 15.59 (δ = 6.02)	22.93	317.17	25.51	21.22	12.99
420	70.49 (δ = 8.69)	4.07 (δ = 7.56)	− 1.72 (δ = 8.21)	4.42	337.11	26.92	28.53	18.22
440	76.52 (δ = 8.03)	− 0.62 (δ = 3.27)	3.63 (δ = 4.26)	3.68	99.76	12.46	15.98	9.09
460	78.16 (δ = 7.85)	− 2.17 (δ = 2.47)	6.37 (δ = 3.83)	6.73	108.79	10.60	14.87	3.15
480	78.94 (δ = 7.48)	− 3.16 (δ = 1.70)	10.32 (δ = 4.36)	10.79	107.01	6.86	13.00	3.03
500	78.81 (δ = 7.25)	− 2.93 (δ = 1.17)	13.26 (δ = 4.96)	13.58	102.44	4.97	14.97	2.01
520	78.79 (δ = 6.84)	− 2.69 (δ = 1.04)	14.79 (δ = 4.85)	15.03	100.32	4.14	14.98	1.03
540	79.11 (δ = 6.48)	− 2.34 (δ = 0.88)	16.09 (δ = 5.30)	16.25	98.27	3.08	17.02	0.95
560	78.90 (δ = 6.53)	− 1.74 (δ = 0.83)	17.17 (δ = 5.50)	17.26	95.79	2.68	18.25	0.98
580	76.15 (δ = 6.91)	− 0.60 (δ = 0.94)	19.29 (δ = 5.94)	19.30	91.79	3.47	20.32	2.65
600	76.15 (δ = 6.50)	− 0.08 (δ = 0.84)	19.77 (δ = 6.09)	19.77	90.23	3.28	18.79	0.63

When the temperature is lower than 360 °C, the amethyst color does not change significantly. As the temperature rises to 380 °C, the violet color begins to fade out and gradually becomes colorless. Then it changes to light green at 420–440 °C. Therefore, prasiolite can be obtained by heating amethyst from 420 to 440 °C, such as the N-05 sample at 440 °C (Fig. 4b). As the temperature continues to rise, it slowly changes to yellow and gradually deepens. Since some samples has color bands, we have noticed that they have different degrees of fading during the heating process at different areas of the color bands. For example, when the samples are heated to 420 °C, some areas become colorless and other areas are still light violet. It seems that the degree of color change of amethyst after heating may be related to its’ initial color^44^.

**Figure 5:**
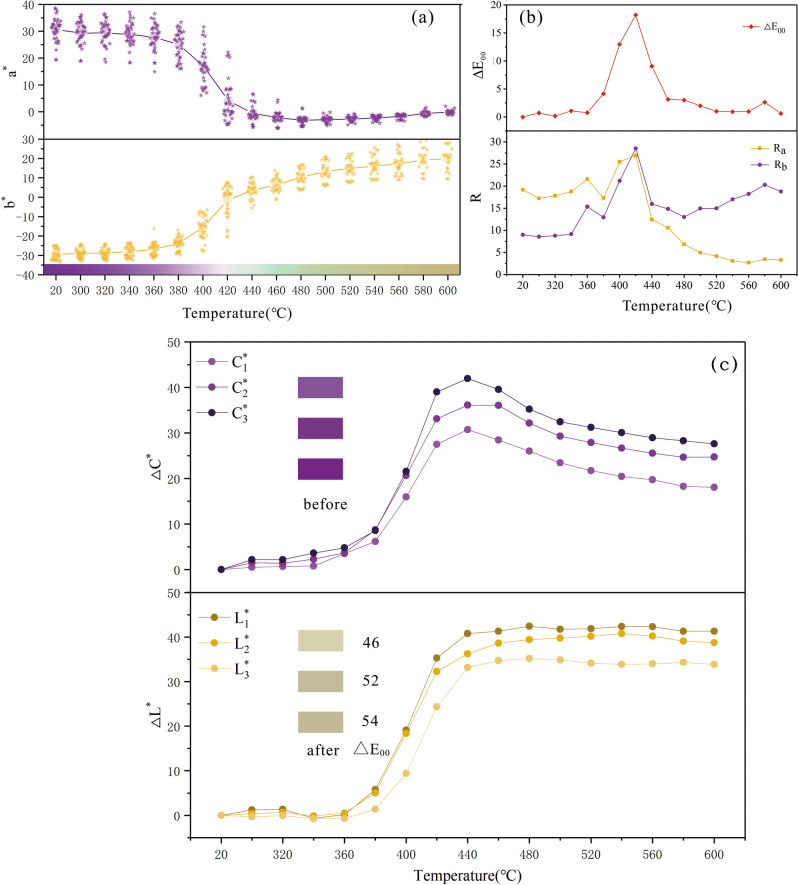
The change of amethyst color parameters under different heat treatment temperature. (**a**) The box plot of relationship between a*, b* value and temperature. (**b**) The relationship between R_a_, R_b_, ΔE_00_ and temperature. R_a_, R_b_ and ΔE_00_ reach the maximum value at 420 °C. (**c**) The relationship between the degree of color change of amethyst and the initial color. ΔE_00_ represents the color difference of each categories before and after heat treatment. When the initial color is darker, the amethyst color is easier to change after heat treatment. The figures were drawn using the OriginPro 2017C Beta b9.4.1.268 (https://www.OriginLab.com) and CorelDRAW Graphics Suite X8 2016 18.0.0.405 (https://www.corel.com).

However, when the temperature arrives 420 °C, the hue gradually shifts from green towards yellow as the temperature rises. The reason for the hue change can be explained by the changes of a* and b* (Fig. 5a). The positive and negative of a* axis represent red and green respectively. a* shows a trend of decreasing from positive to negative and then slowly rising to 0 with increasing temperature, which causes the hue to change from red to green and then return to red. The positive and negative of the b* axis represent yellow and blue respectively, b* keeps rising from negative to positive which causes the hue to change from blue to yellow. When the temperature reaches 420 °C, R_a_, R_b_ and ΔE_00_ shown in Table 3 respectively reach the maximum value of 26.92, 28.53, 18.22 (Fig. 5b). It seems that the color center of amethyst is the most unstable at 420 °C. Besides, we noticed that the color difference at 580 °C is 2.65 and it’s different from that of 560 °C (0.98) and 600 °C (0.63). We believe that the suddenly increase of color difference at 580 °C can be related to the irreversible destruction of color center when the temperature is above the phase transition temperature (573 °C) of amethyst^34^.

In order to investigate whether the degree of color change of amethyst after heat treatment is related to its initial color, the amethyst color is respectively divided into three categories according to their initial lightness and chroma: L_1_* (20–35), L_1_* (35–45), L_1_* (45–55) and C_1_* (30–40), C_2_* (40–45), C_3_* (45–55). ΔL* and ΔC* respectively represent the difference between the values at current temperature of each categories and the corresponding initial values. The relationship between ΔC*, ΔL* and temperature are shown in the Fig. 5c. It can be concluded that the higher the initial chroma is, the greater the difference in chroma and the lower the initial lightness is, the greater the difference in lightness after heat treatment. Taking the change of color difference into account, the ΔE_00_ of each category before and after heat treatment are calculated (Fig. 5c.). It can be known that the when the initial color is darker, the color difference of samples after heat treatment is larger. Therefore, the initial color of amethyst has an effect on its color change after heat treatment. The darker the initial color is, the easier it is to change the color after heat treatment, which explains why different areas of the same sample have different degrees of fading after heating.

**Figure 6:**
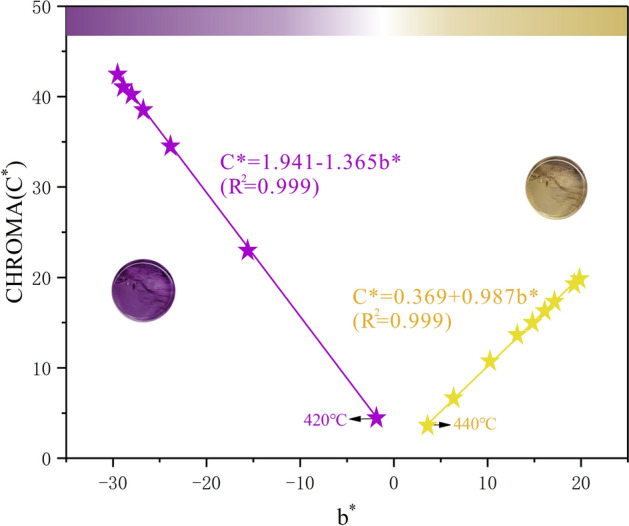
The relationship between b* and C* at different temperatures. The figure was drawn using the OriginPro 2017C Beta b9.4.1.268 (https://www.OriginLab.com).

The b* and C* of samples mean value at different temperatures are plotted in Fig. 6. C* first decreases and then rises with b* and the change of C* is divided into two parts: when the temperature is lower than 420 °C, C* is negatively correlated with b*; When the temperature is higher than 440 °C, C* is positively correlated with b*, and the relationship between C* and b* is as follows:

11 $${\text{C}}^{*} = {1}.{941}\left( { \pm 0.{186}0{\text{SEM}}} \right) - {1}.{365}\left( { \pm 0.00{\text{76 SEM}}} \right){\text{b}}^{*} \quad ({\text{R}}^{{2}} = 0.{999},\quad {\text{T}}{ \leqq }{42}0^\circ {\text{C}})$$

12 $$ {\text{C}}^{*} = 0.{369}\left( { \pm 0.{\text{1458 SEM}}} \right) + 0.{987}\left( { \pm 0.0{1}0{\text{1 SEM}}} \right){\text{b}}^{*} \quad ({\text{R}}^{{2}} = 0.{999},\quad {\text{T}}{ \geqq }{44}0^\circ {\text{C}}) $$

In summary, the color change of amethyst can be divides into three stages with temperature: the amethyst stage with temperature below 420 °C, violet gradually weakens and the color center gradually destructs with the temperature; the prasiolite stage with temperature between 420 and 440 °C, which is the stage of transition from amethyst to citrine and the color center is the most unstable at 420 °C; the citrine stage with temperature above 440 °C, yellow gradually deepens with the temperature.

### Color grading of heat-treated amethyst

In order to get a more appropriate temperature range of amethyst heat treatment, the color parameters in the citrine stage (when temperature is above 440 °C) were classified. The standard of a more appropriate temperature range is the largest number of samples in the best color category after classification. Based on CIE1976 L*a*b* uniform color space system, the classification uses two mathematical methods: K-means clustering analysis and Fisher discriminant analysis. These two methods have been verified in peridot^24,25^, tourmaline^26,27^, jadeite^29–31^ and other colored minerals. K-means Cluster analysis is a statistical analysis technology that divides the research objects into relatively homogeneous groups. Fisher discriminant analysis is one of the important methods of multivariate statistical analysis^24^.

In this paper, three independent color parameters L*, a* and b* are selected for K-means cluster analysis. The clustering effect is the best (sig < 0.001), when the number of categories is 5 (Table 4), which shows that the classification effect is obvious and the accuracy of color data updating is 98.4%. Therefore, the scheme of dividing citrine color into five categories is effective and feasible. Fisher discriminant function is used to test the clustering effect, and the discriminant function corresponding to the color of five categories of citrine color is obtained as follows (Table 5):

13 $$ {\text{F}}1 = 0.987{\text{L}}^{*} - 0.326{\text{a}}^{*} + 0.922{\text{b}}^{*} - 327.807 $$

14 $$ {\text{F}}2 = {8}.561{\text{L}}^{*} - {1}.116{\text{a}}^{*} + {2}.191{\text{b}}^{*} - 307.956 $$

15 $$ {\text{F}}3 = {9}.765{\text{L}}^{*} - {1}.446{\text{a}}^{*} + {2}.777{\text{b}}^{*} - 406.711 $$

16 $$ {\text{F}}4 = {1}0.991{\text{L}}^{*} - {1}.035{\text{a}}^{*} + {1}.{2}04{\text{b}}^{*} - 480.334 $$

17 $$ {\text{F}}5 = {1}0.345{\text{L}}^{*} - {1}.437{\text{a}}^{*} + {1}.792{\text{b}}^{*} - {433}.{888} $$

**Table 4:** ANOVA of L*, a*, and b* of citrine color stage.

	Clustering		Error		F	Sig
	Mean square	df	Mean square	Df		
L*	2,151.393	4	7.851	187	274.044	0.000
a*	18.327	4	2.487	187	7.368	0.000
b*	1634.685	4	9.475	187	172.525	0.000

**Table 5:** Cluster center and Fisher discriminant accuracy.

Fisher discriminant accuracy	1	2	3	4	5	Total
1	100%	0	0	0	0	100%
2	0	93.3%	6.7%	0	0	100%
3	0	0	100%	0	0	100%
4	0	0	0	100%	0	100%
5	1.9%	0	0	0	98.1%	100%

Classification						
Cluster center	1	2	3	4	5	
L*	71.126	66.7703	76.024	86.013	80.806	
a*	− 0.408	− 1.719	− 2.243	− 2.068	− 2.748	
b*	6.245	1.148	23.249	8.267	13.783	
Simulated color						

**Figure 7:**
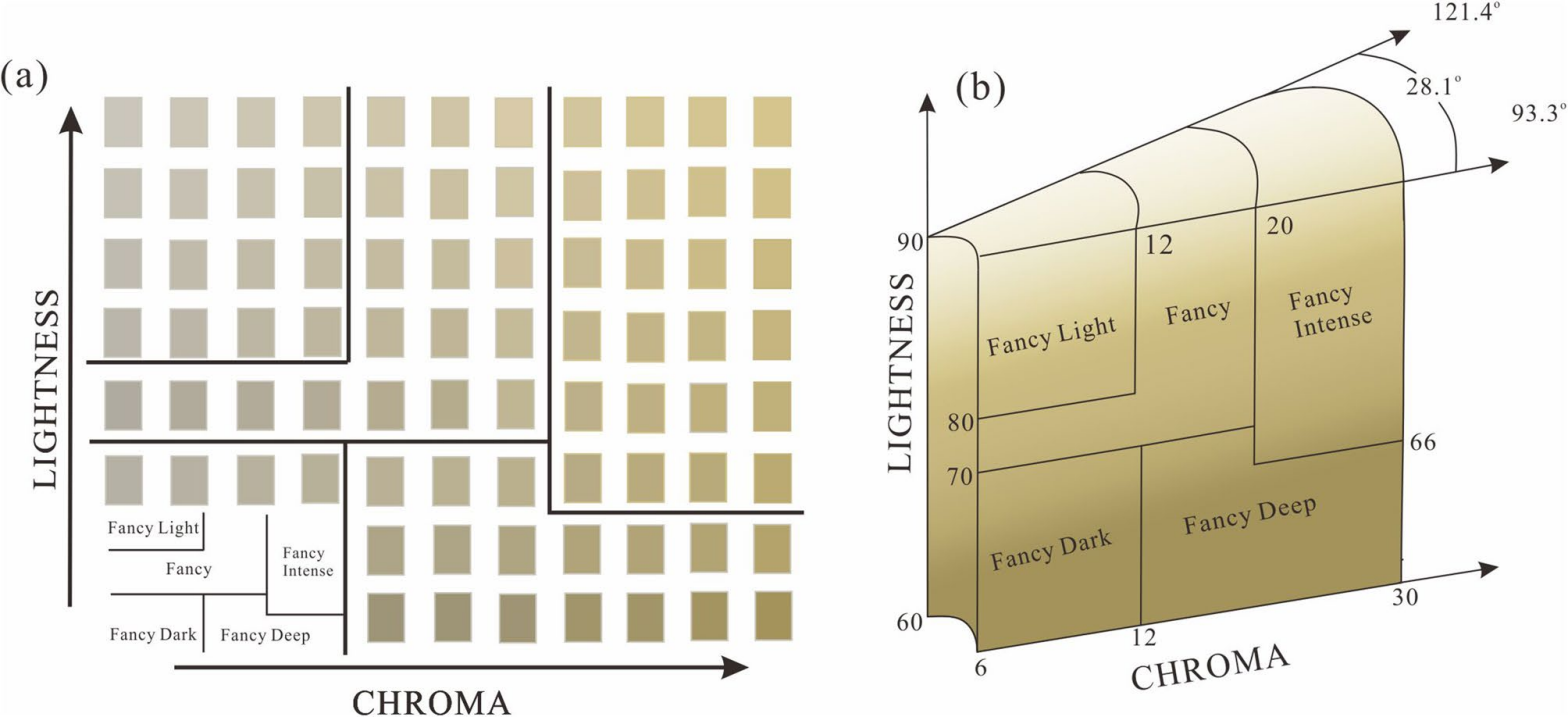
Color Grading of heat-treated amethyst. (**a**) The color of citrine was graded into five levels: Fancy Intense, Fancy Deep, Fancy Dark, Fancy and Fancy Light. (**b**) Citrine heated from amethyst color grading system. The system considers lightness, chroma, and hue. The figures were drawn using CorelDRAW Graphics Suite X8 2016 18.0.0.405 (https://www.corel.com).

For citrine color appreciation, L* should be considered as the primary factor because it can correspond visual effect typically and directly, and then take hue and chroma into account together comprehensively^28^. According to the results of K-means cluster analysis and Fisher discriminant analysis, by imitating GIA’s colored diamond grading system^15^, the color in the citrine stage were divided into five grades: Fancy Intense, Fancy Deep, Fancy Dark , Fancy and Fancy Light, when hue angle ranges from is 93.3° to 121.4° (Fig. 7). The best grade “fancy intense” has the lightness ranges from 66 to 90 and the chroma ranges from 20 to 30; the second grade “fancy” has the lightness ranges from 70 to 90 and the chroma ranges from 6 to 20; the third grade “fancy deep” has the lightness ranges from 60 to 70 and the chroma ranges from 12 to 30; the fourth grade “fancy dark” has the lightness ranges from 60 to 70 and the chroma ranges from 6 to 12; the fifth grade “fancy light” has the lightness ranges from 80 to 90 and the chroma ranges from 6 to 12. The temperatures with the largest number of samples in the Fancy intense category are 560 °C and 600 °C. Because 600 °C is higher than the phase transition temperature of α-quartz and milky white color may appear after heat treatment, it is suggested that a more appropriate temperature range to obtain the best color of citrine by heating amethyst is about 560 °C.

## Discussion

The color at different heated temperatures has no obvious relationship with cell parameters, but the crystallinity index CI of amethyst decreases as the temperature rises. When the temperature is above 600 °C (higher than the phase transition temperature of amethyst), the amethyst becomes milky and turns lighter, which can be related to the destruction of the color centers and Rayleigh scattering of light due to the aggregation of water.

In the UV–Vis spectra, the absorption band at 545 nm (which is related to a charge-transfer transition of Fe^3+^ and O^2−^) has a significant relationship with the color of amethyst. The larger the band area at 545 nm is, the lower the lightness and the higher the chroma, which means the amethyst color will be darker.

The color of amethyst under heat treatment can be divided into three stages with temperature: the amethyst stage with temperature below 420 °C, violet gradually weakens with the temperature; the prasiolite stage with temperature between 420 °C and 440 °C, the color center of amethyst is the most unstable at 420 °C; the citrine stage with temperature above 440 °C, yellow gradually deepens with the temperature. When the temperature is over 600 °C, it may appear milky white. The increase of color difference at 580 °C can be related to the irreversible destruction of color center when the temperature is above the phase transition temperature (573 °C) of amethyst.

The color change degree of amethyst after heat treatment is related to the initial color. The darker the initial color, the larger the color difference of samples after heat treatment, and the easier it is to change the color. The color of citrine obtained from heating amethyst can be divided into five categories and a more appropriate heating temperature to obtain citrine is about 560 °C.

## Materials and methods

### Heating method

The heating instrument is the KSL-1100X-S miniature box furnace of Hefei Kejing whose heating element is resistance wire. The temperature control system adopts intelligent programmable control, and the accuracy is ± 1 °C with the heating power keeping 1200 W. In order to heat these samples evenly, they were buried in pure quartz powder and placed in an alumina crucible. The samples were heated 16 times in the oxidation atmosphere, and the temperature ranges from 300 to 600 °C and the temperature is increased by 20 °C for each heating time. After holding for 10 min, the samples were naturally cooled down to room temperature in the furnace and then taken out to test and the cooling rate was 1.5 °C per minute.

### X-ray diffraction

Amethyst was powdered into 200 orders in an agate mortar. After drying, it was tested by BRUKER D8 Advance from Germany in State Key Laboratory of Earthquake Dynamics, Institute of Geology, China Earthquake Administration. The test conditions are: Cu K-α radiation (wavelength = 1.54 Å); scan step, 0.02; 40 kV tube voltage; 40 mA tube current; test angle of 3°–90°; test time of 434 s; scan mode: continuous PSD fast.

### UV–Vis spectroscopy

The UV–Vis spectra were tested by using UV-3600 UV–VIS spectrophotometer. The test conditions are as follows: range, 300–800 nm; single scanning mode; high scanning speed, sampling interval, 0.5 s; beam mode, double beam; slit width, 2.0 nm; slit program, normal; grating conversion wavelength, 720.00 nm; detector conversion wavelength, 900.00 nm; S/R conversion, standard; automatic lock the detector; step correction.

### Colorimetric analysis

The color parameters were measured by X-Rite SP6 spectrophotometer in a standard illumination box with a fluorescent lamp (CCT 6504K, PHILIPS MASTERTL-D 90 De Luxe 18W/965, Holland) and the background was N9 gray level of Munsell neutral color chips. In order to obtain accurate color parameters, the average value of each sample was taken after three tests. The X-Rite SP6 spectrophotometer collects the surface reflection signal of the sample through the integrating sphere and then converts the signal into the color parameters. The test conditions are: reflection method, not including the specular reflection, D65 standard lighting source, observer view of 2°, measurement time of less than 2.5 s, measurement range from 400 to 700 nm, wavelength interval of 10 nm.

## Supplementary information

Supplementary information.
