# Supplementary material for: Study on the effect of heat treatment on amethyst color and the cause of coloration
Source: Sci Rep. 2020 Sep 10;10:14927. doi: 10.1038/s41598-020-71786-1 (PMC7483767; doi:10.1038/s41598-020-71786-1)
Supplement: Supplementary file 1 — Supplementary information. [file 41598_2020_71786_MOESM1_ESM.docx]

**Study on the Effect of Heat Treatment on Amethyst Color and the Cause of Coloration**

Renping Cheng^1^ & Ying Guo^2&(Corresponding Author)^

^1^ Department of Gemmology, China University of Geosciences (Beijing), 100083, China. E-mail:chengrenping@foxmail.com

^2^ Department of Gemmology, China University of Geosciences (Beijing), 100083, China. E-mail:guoying@cugb.edu.cn

| **XRD data of amethyst at different temperatures (figure 2)** | | | | | | | | | |
| --- | --- | --- | --- | --- | --- | --- | --- | --- | --- |
| 2θ | | unteated | | 400℃ | | 500℃ | | 600℃ | |
| 3.0001 | | 2253 | | 2338 | | 2091 | | 2245 | |
| 3.02055 | | 2123 | | 2251 | | 2036 | | 2160 | |
| 3.041 | | 2040 | | 2163 | | 1993 | | 1992 | |
| 3.06146 | | 1947 | | 2122 | | 1907 | | 1941 | |
| 3.08191 | | 1914 | | 2022 | | 1807 | | 1777 | |
| 3.10236 | | 1759 | | 1912 | | 1818 | | 1778 | |
| 3.12281 | | 1670 | | 1843 | | 1584 | | 1743 | |
| 3.14326 | | 1598 | | 1744 | | 1487 | | 1565 | |
| 3.16372 | | 1527 | | 1627 | | 1537 | | 1551 | |
| 3.18417 | | 1553 | | 1599 | | 1463 | | 1487 | |
| 3.20462 | | 1420 | | 1483 | | 1346 | | 1438 | |
| 3.22507 | | 1290 | | 1427 | | 1252 | | 1305 | |
| 3.24552 | | 1258 | | 1373 | | 1218 | | 1265 | |
| 3.26598 | | 1204 | | 1227 | | 1136 | | 1160 | |
| 3.28643 | | 1141 | | 1191 | | 1118 | | 1139 | |
| 3.30688 | | 1025 | | 1119 | | 1032 | | 1065 | |
| 3.32733 | | 973 | | 1067 | | 912 | | 1006 | |
| 3.34778 | | 917 | | 955 | | 875 | | 948 | |
| 3.36824 | | 885 | | 939 | | 853 | | 884 | |
| 3.38869 | | 883 | | 893 | | 728 | | 822 | |
| 3.40914 | | 785 | | 791 | | 698 | | 741 | |
| 3.42959 | | 729 | | 779 | | 686 | | 755 | |
| 3.45004 | | 677 | | 738 | | 625 | | 721 | |
| 3.47049 | | 671 | | 742 | | 607 | | 607 | |
| 3.49095 | | 614 | | 656 | | 555 | | 601 | |
| 3.5114 | | 618 | | 606 | | 543 | | 607 | |
| 3.53185 | | 496 | | 585 | | 501 | | 511 | |
| 3.5523 | | 480 | | 554 | | 506 | | 535 | |
| 3.57275 | | 526 | | 523 | | 437 | | 519 | |
| 3.59321 | | 484 | | 544 | | 464 | | 499 | |
| 3.61366 | | 480 | | 566 | | 462 | | 451 | |
| 3.63411 | | 490 | | 504 | | 454 | | 471 | |
| 3.65456 | | 447 | | 498 | | 415 | | 477 | |
| 3.67501 | | 415 | | 469 | | 396 | | 440 | |
| 3.69547 | | 447 | | 451 | | 432 | | 430 | |
| 3.71592 | | 422 | | 453 | | 445 | | 430 | |
| 3.73637 | | 444 | | 436 | | 401 | | 411 | |
| 3.75682 | | 416 | | 449 | | 392 | | 428 | |
| 3.77727 | | 394 | | 414 | | 395 | | 394 | |
| 3.79773 | | 420 | | 427 | | 383 | | 402 | |
| 3.81818 | | 392 | | 416 | | 372 | | 394 | |
| 3.83863 | | 402 | | 454 | | 357 | | 366 | |
| 3.85908 | | 370 | | 396 | | 360 | | 402 | |
| 3.87953 | | 363 | | 434 | | 386 | | 381 | |
| 3.89999 | | 372 | | 417 | | 357 | | 368 | |
| 3.92044 | | 402 | | 387 | | 340 | | 362 | |
| 3.94089 | | 381 | | 411 | | 349 | | 334 | |
| 3.96134 | | 353 | | 393 | | 337 | | 350 | |
| 3.98179 | | 357 | | 364 | | 340 | | 382 | |
| 4.00225 | | 343 | | 363 | | 350 | | 336 | |
| 4.0227 | | 312 | | 347 | | 332 | | 340 | |
| 4.04315 | | 342 | | 338 | | 304 | | 359 | |
| 4.0636 | | 379 | | 350 | | 322 | | 318 | |
| 4.08405 | | 342 | | 359 | | 340 | | 325 | |
| 4.10451 | | 335 | | 324 | | 303 | | 346 | |
| 4.12496 | | 344 | | 369 | | 297 | | 324 | |
| 4.14541 | | 301 | | 326 | | 279 | | 319 | |
| 4.16586 | | 314 | | 352 | | 306 | | 305 | |
| 4.18631 | | 336 | | 334 | | 279 | | 312 | |
| 4.20676 | | 278 | | 303 | | 251 | | 293 | |
| 4.22722 | | 274 | | 311 | | 300 | | 300 | |
| 4.24767 | | 325 | | 346 | | 292 | | 323 | |
| 4.26812 | | 266 | | 283 | | 269 | | 289 | |
| 4.28857 | | 329 | | 313 | | 260 | | 305 | |
| 4.30902 | | 271 | | 295 | | 291 | | 274 | |
| 4.32948 | | 282 | | 304 | | 286 | | 283 | |
| 4.34993 | | 308 | | 286 | | 279 | | 268 | |
| 4.37038 | | 308 | | 291 | | 252 | | 276 | |
| 4.39083 | | 248 | | 306 | | 271 | | 270 | |
| 4.41128 | | 283 | | 276 | | 266 | | 277 | |
| 4.43174 | | 261 | | 291 | | 238 | | 249 | |
| 4.45219 | | 246 | | 279 | | 267 | | 262 | |
| 4.47264 | | 264 | | 277 | | 257 | | 263 | |
| 4.49309 | | 278 | | 280 | | 239 | | 257 | |
| 4.51354 | | 231 | | 261 | | 253 | | 257 | |
| 4.534 | | 273 | | 239 | | 254 | | 253 | |
| 4.55445 | | 257 | | 273 | | 233 | | 271 | |
| 4.5749 | | 266 | | 261 | | 227 | | 242 | |
| 4.59535 | | 233 | | 252 | | 211 | | 259 | |
| 4.6158 | | 251 | | 264 | | 236 | | 209 | |
| 4.63626 | | 229 | | 257 | | 271 | | 239 | |
| 4.65671 | | 203 | | 246 | | 234 | | 243 | |
| 4.67716 | | 221 | | 239 | | 236 | | 218 | |
| 4.69761 | | 217 | | 248 | | 235 | | 232 | |
| 4.71806 | | 225 | | 220 | | 213 | | 239 | |
| 4.73852 | | 224 | | 217 | | 213 | | 225 | |
| 4.75897 | | 214 | | 213 | | 222 | | 250 | |
| 4.77942 | | 245 | | 225 | | 222 | | 206 | |
| 4.79987 | | 212 | | 231 | | 210 | | 241 | |
| 4.82032 | | 223 | | 216 | | 193 | | 233 | |
| 4.84078 | | 188 | | 229 | | 182 | | 204 | |
| 4.86123 | | 191 | | 214 | | 210 | | 209 | |
| 4.88168 | | 196 | | 199 | | 190 | | 197 | |
| 4.90213 | | 203 | | 212 | | 192 | | 210 | |
| 4.92258 | | 193 | | 214 | | 200 | | 185 | |
| 4.94303 | | 197 | | 230 | | 192 | | 185 | |
| 4.96349 | | 167 | | 211 | | 172 | | 194 | |
| 4.98394 | | 236 | | 198 | | 201 | | 207 | |
| 5.00439 | | 212 | | 182 | | 168 | | 190 | |
| 5.02484 | | 208 | | 201 | | 197 | | 204 | |
| 5.04529 | | 214 | | 201 | | 176 | | 160 | |
| 5.06575 | | 197 | | 190 | | 179 | | 205 | |
| 5.0862 | | 187 | | 181 | | 175 | | 191 | |
| 5.10665 | | 166 | | 166 | | 180 | | 180 | |
| 5.1271 | | 207 | | 185 | | 185 | | 189 | |
| 5.14755 | | 178 | | 179 | | 207 | | 168 | |
| 5.16801 | | 176 | | 169 | | 171 | | 195 | |
| 5.18846 | | 179 | | 194 | | 197 | | 183 | |
| 5.20891 | | 180 | | 175 | | 181 | | 188 | |
| 5.22936 | | 157 | | 205 | | 171 | | 170 | |
| 5.24981 | | 174 | | 185 | | 160 | | 185 | |
| 5.27027 | | 181 | | 200 | | 160 | | 166 | |
| 5.29072 | | 167 | | 198 | | 162 | | 178 | |
| 5.31117 | | 182 | | 194 | | 160 | | 143 | |
| 5.33162 | | 180 | | 206 | | 168 | | 171 | |
| 5.35207 | | 168 | | 163 | | 166 | | 177 | |
| 5.37253 | | 192 | | 188 | | 173 | | 169 | |
| 5.39298 | | 183 | | 176 | | 181 | | 173 | |
| 5.41343 | | 158 | | 155 | | 179 | | 191 | |
| 5.43388 | | 165 | | 182 | | 163 | | 160 | |
| 5.45433 | | 189 | | 145 | | 174 | | 161 | |
| 5.47479 | | 182 | | 179 | | 160 | | 155 | |
| 5.49524 | | 164 | | 181 | | 168 | | 161 | |
| 5.51569 | | 185 | | 170 | | 134 | | 161 | |
| 5.53614 | | 145 | | 172 | | 169 | | 156 | |
| 5.55659 | | 166 | | 183 | | 145 | | 171 | |
| 5.57705 | | 162 | | 164 | | 167 | | 134 | |
| 5.5975 | | 120 | | 158 | | 159 | | 187 | |
| 5.61795 | | 154 | | 187 | | 158 | | 170 | |
| 5.6384 | | 173 | | 169 | | 165 | | 158 | |
| 5.65885 | | 156 | | 181 | | 142 | | 145 | |
| 5.6793 | | 144 | | 168 | | 131 | | 176 | |
| 5.69976 | | 165 | | 178 | | 162 | | 167 | |
| 5.72021 | | 140 | | 166 | | 174 | | 161 | |
| 5.74066 | | 164 | | 154 | | 168 | | 151 | |
| 5.76111 | | 145 | | 174 | | 139 | | 173 | |
| 5.78156 | | 183 | | 160 | | 161 | | 139 | |
| 5.80202 | | 152 | | 173 | | 158 | | 136 | |
| 5.82247 | | 129 | | 171 | | 170 | | 168 | |
| 5.84292 | | 153 | | 141 | | 148 | | 162 | |
| 5.86337 | | 132 | | 158 | | 144 | | 148 | |
| 5.88382 | | 142 | | 164 | | 165 | | 155 | |
| 5.90428 | | 142 | | 180 | | 146 | | 152 | |
| 5.92473 | | 137 | | 141 | | 146 | | 130 | |
| 5.94518 | | 140 | | 175 | | 142 | | 146 | |
| 5.96563 | | 171 | | 146 | | 160 | | 161 | |
| 5.98608 | | 142 | | 139 | | 164 | | 161 | |
| 6.00654 | | 135 | | 134 | | 149 | | 172 | |
| 6.02699 | | 161 | | 148 | | 134 | | 168 | |
| 6.04744 | | 164 | | 146 | | 140 | | 155 | |
| 6.06789 | | 127 | | 162 | | 159 | | 137 | |
| 6.08834 | | 178 | | 130 | | 157 | | 155 | |
| 6.1088 | | 138 | | 150 | | 158 | | 135 | |
| 6.12925 | | 142 | | 160 | | 148 | | 149 | |
| 6.1497 | | 153 | | 148 | | 144 | | 156 | |
| 6.17015 | | 144 | | 136 | | 146 | | 138 | |
| 6.1906 | | 153 | | 178 | | 141 | | 148 | |
| 6.21106 | | 154 | | 146 | | 181 | | 114 | |
| 6.23151 | | 156 | | 146 | | 141 | | 124 | |
| 6.25196 | | 139 | | 150 | | 154 | | 148 | |
| 6.27241 | | 147 | | 140 | | 142 | | 134 | |
| 6.29286 | | 144 | | 134 | | 151 | | 152 | |
| 6.31332 | | 129 | | 135 | | 153 | | 137 | |
| 6.33377 | | 132 | | 136 | | 138 | | 140 | |
| 6.35422 | | 130 | | 146 | | 145 | | 132 | |
| 6.37467 | | 114 | | 151 | | 133 | | 141 | |
| 6.39512 | | 114 | | 146 | | 130 | | 134 | |
| 6.41557 | | 134 | | 123 | | 131 | | 127 | |
| 6.43603 | | 148 | | 140 | | 138 | | 151 | |
| 6.45648 | | 145 | | 156 | | 132 | | 128 | |
| 6.47693 | | 114 | | 131 | | 147 | | 141 | |
| 6.49738 | | 154 | | 143 | | 133 | | 173 | |
| 6.51783 | | 124 | | 127 | | 136 | | 133 | |
| 6.53829 | | 142 | | 157 | | 129 | | 146 | |
| 6.55874 | | 143 | | 160 | | 113 | | 111 | |
| 6.57919 | | 143 | | 148 | | 129 | | 121 | |
| 6.59964 | | 142 | | 152 | | 144 | | 133 | |
| 6.62009 | | 125 | | 149 | | 157 | | 140 | |
| 6.64055 | | 135 | | 153 | | 140 | | 163 | |
| 6.661 | | 156 | | 137 | | 144 | | 137 | |
| 6.68145 | | 140 | | 154 | | 120 | | 152 | |
| 6.7019 | | 126 | | 126 | | 158 | | 131 | |
| 6.72235 | | 121 | | 159 | | 148 | | 142 | |
| 6.74281 | | 152 | | 136 | | 137 | | 150 | |
| 6.76326 | | 128 | | 140 | | 143 | | 152 | |
| 6.78371 | | 159 | | 125 | | 124 | | 149 | |
| 6.80416 | | 119 | | 144 | | 125 | | 139 | |
| 6.82461 | | 144 | | 143 | | 139 | | 152 | |
| 6.84507 | | 120 | | 144 | | 128 | | 138 | |
| 6.86552 | | 122 | | 134 | | 126 | | 146 | |
| 6.88597 | | 156 | | 137 | | 153 | | 118 | |
| 6.90642 | | 148 | | 130 | | 135 | | 119 | |
| 6.92687 | | 141 | | 151 | | 128 | | 129 | |
| 6.94733 | | 162 | | 133 | | 141 | | 126 | |
| 6.96778 | | 115 | | 140 | | 149 | | 135 | |
| 6.98823 | | 132 | | 140 | | 139 | | 134 | |
| 7.00868 | | 128 | | 144 | | 131 | | 131 | |
| 7.02913 | | 128 | | 138 | | 121 | | 136 | |
| 7.04959 | | 144 | | 141 | | 127 | | 125 | |
| 7.07004 | | 130 | | 134 | | 154 | | 127 | |
| 7.09049 | | 134 | | 122 | | 123 | | 138 | |
| 7.11094 | | 146 | | 131 | | 127 | | 123 | |
| 7.13139 | | 114 | | 140 | | 129 | | 131 | |
| 7.15184 | | 127 | | 130 | | 131 | | 144 | |
| 7.1723 | | 123 | | 140 | | 134 | | 126 | |
| 7.19275 | | 143 | | 140 | | 119 | | 129 | |
| 7.2132 | | 128 | | 131 | | 129 | | 115 | |
| 7.23365 | | 121 | | 152 | | 130 | | 138 | |
| 7.2541 | | 117 | | 126 | | 133 | | 132 | |
| 7.27456 | | 139 | | 134 | | 145 | | 136 | |
| 7.29501 | | 136 | | 119 | | 154 | | 120 | |
| 7.31546 | | 126 | | 143 | | 129 | | 137 | |
| 7.33591 | | 135 | | 124 | | 120 | | 117 | |
| 7.35636 | | 118 | | 132 | | 133 | | 116 | |
| 7.37682 | | 120 | | 128 | | 121 | | 114 | |
| 7.39727 | | 111 | | 119 | | 135 | | 113 | |
| 7.41772 | | 107 | | 126 | | 132 | | 134 | |
| 7.43817 | | 121 | | 133 | | 124 | | 136 | |
| 7.45862 | | 98 | | 144 | | 136 | | 117 | |
| 7.47908 | | 115 | | 135 | | 118 | | 139 | |
| 7.49953 | | 146 | | 128 | | 136 | | 146 | |
| 7.51998 | | 99 | | 132 | | 136 | | 112 | |
| 7.54043 | | 134 | | 123 | | 130 | | 126 | |
| 7.56088 | | 116 | | 108 | | 118 | | 115 | |
| 7.58134 | | 125 | | 124 | | 117 | | 108 | |
| 7.60179 | | 121 | | 133 | | 130 | | 150 | |
| 7.62224 | | 128 | | 129 | | 145 | | 130 | |
| 7.64269 | | 137 | | 127 | | 126 | | 110 | |
| 7.66314 | | 123 | | 126 | | 136 | | 143 | |
| 7.6836 | | 115 | | 123 | | 130 | | 140 | |
| 7.70405 | | 133 | | 150 | | 126 | | 108 | |
| 7.7245 | | 127 | | 131 | | 125 | | 133 | |
| 7.74495 | | 136 | | 138 | | 139 | | 122 | |
| 7.7654 | | 126 | | 152 | | 144 | | 134 | |
| 7.78586 | | 99 | | 115 | | 125 | | 126 | |
| 7.80631 | | 129 | | 136 | | 148 | | 128 | |
| 7.82676 | | 134 | | 141 | | 134 | | 140 | |
| 7.84721 | | 125 | | 140 | | 122 | | 134 | |
| 7.86766 | | 114 | | 116 | | 134 | | 145 | |
| 7.88811 | | 130 | | 118 | | 124 | | 131 | |
| 7.90857 | | 143 | | 120 | | 139 | | 134 | |
| 7.92902 | | 121 | | 127 | | 138 | | 137 | |
| 7.94947 | | 121 | | 132 | | 130 | | 110 | |
| 7.96992 | | 120 | | 111 | | 142 | | 115 | |
| 7.99037 | | 109 | | 140 | | 128 | | 126 | |
| 8.01083 | | 121 | | 113 | | 112 | | 132 | |
| 8.03128 | | 117 | | 130 | | 146 | | 124 | |
| 8.05173 | | 118 | | 118 | | 131 | | 115 | |
| 8.07218 | | 116 | | 126 | | 127 | | 100 | |
| 8.09263 | | 117 | | 128 | | 126 | | 133 | |
| 8.11309 | | 133 | | 123 | | 136 | | 120 | |
| 8.13354 | | 127 | | 126 | | 140 | | 106 | |
| 8.15399 | | 126 | | 119 | | 116 | | 135 | |
| 8.17444 | | 118 | | 146 | | 116 | | 117 | |
| 8.19489 | | 117 | | 124 | | 131 | | 130 | |
| 8.21535 | | 118 | | 131 | | 114 | | 121 | |
| 8.2358 | | 110 | | 113 | | 126 | | 146 | |
| 8.25625 | | 145 | | 113 | | 127 | | 125 | |
| 8.2767 | | 139 | | 135 | | 127 | | 109 | |
| 8.29715 | | 121 | | 114 | | 124 | | 125 | |
| 8.31761 | | 97 | | 120 | | 113 | | 129 | |
| 8.33806 | | 123 | | 119 | | 114 | | 139 | |
| 8.35851 | | 118 | | 114 | | 126 | | 121 | |
| 8.37896 | | 140 | | 144 | | 129 | | 130 | |
| 8.39941 | | 129 | | 140 | | 118 | | 114 | |
| 8.41987 | | 107 | | 119 | | 126 | | 122 | |
| 8.44032 | | 117 | | 121 | | 107 | | 138 | |
| 8.46077 | | 122 | | 133 | | 130 | | 131 | |
| 8.48122 | | 124 | | 119 | | 130 | | 107 | |
| 8.50167 | | 116 | | 103 | | 108 | | 130 | |
| 8.52213 | | 135 | | 134 | | 116 | | 109 | |
| 8.54258 | | 120 | | 128 | | 118 | | 108 | |
| 8.56303 | | 108 | | 121 | | 113 | | 121 | |
| 8.58348 | | 141 | | 132 | | 109 | | 128 | |
| 8.60393 | | 128 | | 127 | | 126 | | 118 | |
| 8.62438 | | 113 | | 127 | | 130 | | 120 | |
| 8.64484 | | 122 | | 122 | | 131 | | 111 | |
| 8.66529 | | 112 | | 127 | | 129 | | 130 | |
| 8.68574 | | 119 | | 125 | | 108 | | 115 | |
| 8.70619 | | 134 | | 120 | | 121 | | 121 | |
| 8.72664 | | 119 | | 114 | | 124 | | 127 | |
| 8.7471 | | 117 | | 111 | | 110 | | 115 | |
| 8.76755 | | 121 | | 115 | | 103 | | 119 | |
| 8.788 | | 130 | | 124 | | 133 | | 122 | |
| 8.80845 | | 124 | | 142 | | 138 | | 120 | |
| 8.8289 | | 132 | | 117 | | 111 | | 115 | |
| 8.84936 | | 117 | | 135 | | 127 | | 130 | |
| 8.86981 | | 98 | | 123 | | 113 | | 116 | |
| 8.89026 | | 122 | | 108 | | 126 | | 90 | |
| 8.91071 | | 112 | | 120 | | 136 | | 117 | |
| 8.93116 | | 110 | | 151 | | 117 | | 108 | |
| 8.95162 | | 123 | | 104 | | 113 | | 116 | |
| 8.97207 | | 107 | | 132 | | 128 | | 122 | |
| 8.99252 | | 128 | | 116 | | 107 | | 110 | |
| 9.01297 | | 116 | | 136 | | 106 | | 112 | |
| 9.03342 | | 116 | | 110 | | 114 | | 105 | |
| 9.05388 | | 108 | | 126 | | 124 | | 114 | |
| 9.07433 | | 124 | | 125 | | 123 | | 104 | |
| 9.09478 | | 106 | | 108 | | 116 | | 125 | |
| 9.11523 | | 116 | | 132 | | 110 | | 113 | |
| 9.13568 | | 139 | | 130 | | 126 | | 138 | |
| 9.15614 | | 108 | | 121 | | 137 | | 116 | |
| 9.17659 | | 128 | | 119 | | 124 | | 103 | |
| 9.19704 | | 124 | | 112 | | 134 | | 100 | |
| 9.21749 | | 122 | | 118 | | 123 | | 124 | |
| 9.23794 | | 121 | | 104 | | 104 | | 125 | |
| 9.2584 | | 117 | | 124 | | 130 | | 124 | |
| 9.27885 | | 119 | | 103 | | 105 | | 97 | |
| 9.2993 | | 109 | | 122 | | 104 | | 124 | |
| 9.31975 | | 123 | | 128 | | 112 | | 111 | |
| 9.3402 | | 104 | | 140 | | 126 | | 114 | |
| 9.36065 | | 110 | | 107 | | 112 | | 128 | |
| 9.38111 | | 122 | | 93 | | 120 | | 136 | |
| 9.40156 | | 94 | | 118 | | 100 | | 103 | |
| 9.42201 | | 115 | | 126 | | 120 | | 91 | |
| 9.44246 | | 117 | | 103 | | 122 | | 119 | |
| 9.46291 | | 134 | | 102 | | 97 | | 94 | |
| 9.48337 | | 112 | | 134 | | 120 | | 127 | |
| 9.50382 | | 121 | | 131 | | 110 | | 114 | |
| 9.52427 | | 118 | | 115 | | 115 | | 112 | |
| 9.54472 | | 103 | | 132 | | 108 | | 117 | |
| 9.56517 | | 105 | | 114 | | 109 | | 128 | |
| 9.58563 | | 123 | | 132 | | 125 | | 119 | |
| 9.60608 | | 119 | | 106 | | 121 | | 115 | |
| 9.62653 | | 111 | | 125 | | 113 | | 105 | |
| 9.64698 | | 109 | | 114 | | 110 | | 109 | |
| 9.66743 | | 99 | | 113 | | 89 | | 112 | |
| 9.68789 | | 112 | | 131 | | 112 | | 103 | |
| 9.70834 | | 113 | | 117 | | 123 | | 115 | |
| 9.72879 | | 113 | | 105 | | 120 | | 113 | |
| 9.74924 | | 119 | | 118 | | 98 | | 108 | |
| 9.76969 | | 103 | | 106 | | 97 | | 109 | |
| 9.79015 | | 121 | | 123 | | 106 | | 105 | |
| 9.8106 | | 111 | | 119 | | 105 | | 122 | |
| 9.83105 | | 128 | | 127 | | 98 | | 101 | |
| 9.8515 | | 92 | | 102 | | 134 | | 94 | |
| 9.87195 | | 107 | | 109 | | 101 | | 117 | |
| 9.89241 | | 100 | | 128 | | 112 | | 114 | |
| 9.91286 | | 96 | | 122 | | 122 | | 97 | |
| 9.93331 | | 113 | | 102 | | 115 | | 122 | |
| 9.95376 | | 108 | | 129 | | 91 | | 92 | |
| 9.97421 | | 118 | | 112 | | 94 | | 125 | |
| 9.99467 | | 114 | | 111 | | 130 | | 104 | |
| 10.0151 | | 103 | | 139 | | 114 | | 97 | |
| 10.0356 | | 117 | | 129 | | 112 | | 97 | |
| 10.056 | | 82 | | 128 | | 99 | | 107 | |
| 10.0765 | | 111 | | 102 | | 119 | | 111 | |
| 10.0969 | | 83 | | 107 | | 92 | | 109 | |
| 10.1174 | | 106 | | 111 | | 104 | | 122 | |
| 10.1378 | | 122 | | 115 | | 107 | | 97 | |
| 10.1583 | | 117 | | 104 | | 107 | | 106 | |
| 10.1787 | | 101 | | 131 | | 95 | | 105 | |
| 10.1992 | | 122 | | 101 | | 103 | | 97 | |
| 10.2196 | | 105 | | 120 | | 87 | | 108 | |
| 10.2401 | | 92 | | 126 | | 103 | | 107 | |
| 10.2605 | | 102 | | 125 | | 83 | | 138 | |
| 10.281 | | 123 | | 138 | | 117 | | 113 | |
| 10.3014 | | 87 | | 91 | | 115 | | 125 | |
| 10.3219 | | 117 | | 115 | | 123 | | 86 | |
| 10.3423 | | 97 | | 102 | | 101 | | 117 | |
| 10.3628 | | 93 | | 114 | | 110 | | 100 | |
| 10.3833 | | 125 | | 111 | | 106 | | 112 | |
| 10.4037 | | 112 | | 101 | | 109 | | 107 | |
| 10.4242 | | 93 | | 104 | | 112 | | 107 | |
| 10.4446 | | 94 | | 119 | | 85 | | 115 | |
| 10.4651 | | 90 | | 98 | | 118 | | 102 | |
| 10.4855 | | 103 | | 89 | | 97 | | 92 | |
| 10.506 | | 99 | | 97 | | 86 | | 110 | |
| 10.5264 | | 97 | | 104 | | 105 | | 88 | |
| 10.5469 | | 100 | | 120 | | 109 | | 114 | |
| 10.5673 | | 109 | | 121 | | 115 | | 108 | |
| 10.5878 | | 78 | | 113 | | 90 | | 108 | |
| 10.6082 | | 106 | | 122 | | 107 | | 111 | |
| 10.6287 | | 111 | | 102 | | 107 | | 106 | |
| 10.6491 | | 110 | | 103 | | 102 | | 100 | |
| 10.6696 | | 100 | | 106 | | 95 | | 103 | |
| 10.69 | | 106 | | 105 | | 106 | | 128 | |
| 10.7105 | | 98 | | 116 | | 94 | | 101 | |
| 10.7309 | | 102 | | 115 | | 109 | | 102 | |
| 10.7514 | | 86 | | 115 | | 105 | | 109 | |
| 10.7718 | | 80 | | 103 | | 107 | | 99 | |
| 10.7923 | | 106 | | 138 | | 100 | | 101 | |
| 10.8127 | | 103 | | 116 | | 95 | | 87 | |
| 10.8332 | | 110 | | 112 | | 113 | | 95 | |
| 10.8536 | | 83 | | 96 | | 89 | | 102 | |
| 10.8741 | | 95 | | 108 | | 91 | | 98 | |
| 10.8946 | | 100 | | 113 | | 101 | | 102 | |
| 10.915 | | 98 | | 111 | | 111 | | 107 | |
| 10.9355 | | 100 | | 121 | | 97 | | 89 | |
| 10.9559 | | 89 | | 127 | | 96 | | 95 | |
| 10.9764 | | 86 | | 112 | | 101 | | 99 | |
| 10.9968 | | 95 | | 94 | | 124 | | 109 | |
| 11.0173 | | 104 | | 97 | | 80 | | 116 | |
| 11.0377 | | 115 | | 109 | | 80 | | 105 | |
| 11.0582 | | 107 | | 105 | | 100 | | 91 | |
| 11.0786 | | 100 | | 114 | | 95 | | 115 | |
| 11.0991 | | 94 | | 98 | | 91 | | 109 | |
| 11.1195 | | 97 | | 85 | | 103 | | 95 | |
| 11.14 | | 89 | | 96 | | 87 | | 101 | |
| 11.1604 | | 98 | | 115 | | 108 | | 98 | |
| 11.1809 | | 80 | | 106 | | 105 | | 111 | |
| 11.2013 | | 98 | | 108 | | 106 | | 97 | |
| 11.2218 | | 98 | | 102 | | 87 | | 102 | |
| 11.2422 | | 105 | | 109 | | 113 | | 103 | |
| 11.2627 | | 109 | | 132 | | 105 | | 114 | |
| 11.2831 | | 99 | | 91 | | 98 | | 93 | |
| 11.3036 | | 80 | | 135 | | 106 | | 105 | |
| 11.324 | | 94 | | 104 | | 84 | | 90 | |
| 11.3445 | | 103 | | 119 | | 90 | | 102 | |
| 11.3649 | | 98 | | 109 | | 65 | | 106 | |
| 11.3854 | | 108 | | 119 | | 78 | | 103 | |
| 11.4058 | | 81 | | 119 | | 90 | | 106 | |
| 11.4263 | | 101 | | 97 | | 95 | | 110 | |
| 11.4468 | | 101 | | 118 | | 95 | | 105 | |
| 11.4672 | | 101 | | 98 | | 92 | | 102 | |
| 11.4877 | | 91 | | 93 | | 82 | | 93 | |
| 11.5081 | | 94 | | 120 | | 87 | | 106 | |
| 11.5286 | | 87 | | 115 | | 98 | | 113 | |
| 11.549 | | 100 | | 106 | | 97 | | 114 | |
| 11.5695 | | 91 | | 121 | | 107 | | 93 | |
| 11.5899 | | 78 | | 103 | | 81 | | 96 | |
| 11.6104 | | 111 | | 115 | | 83 | | 95 | |
| 11.6308 | | 78 | | 124 | | 82 | | 89 | |
| 11.6513 | | 82 | | 103 | | 96 | | 101 | |
| 11.6717 | | 89 | | 112 | | 85 | | 84 | |
| 11.6922 | | 86 | | 89 | | 94 | | 82 | |
| 11.7126 | | 78 | | 112 | | 115 | | 81 | |
| 11.7331 | | 76 | | 90 | | 102 | | 98 | |
| 11.7535 | | 83 | | 107 | | 83 | | 101 | |
| 11.774 | | 78 | | 96 | | 81 | | 106 | |
| 11.7944 | | 83 | | 97 | | 87 | | 75 | |
| 11.8149 | | 81 | | 106 | | 104 | | 107 | |
| 11.8353 | | 80 | | 98 | | 89 | | 73 | |
| 11.8558 | | 85 | | 92 | | 94 | | 95 | |
| 11.8762 | | 93 | | 107 | | 98 | | 100 | |
| 11.8967 | | 84 | | 115 | | 75 | | 86 | |
| 11.9171 | | 93 | | 116 | | 112 | | 90 | |
| 11.9376 | | 88 | | 101 | | 71 | | 92 | |
| 11.9581 | | 111 | | 89 | | 87 | | 86 | |
| 11.9785 | | 87 | | 107 | | 84 | | 86 | |
| 11.999 | | 84 | | 102 | | 83 | | 99 | |
| 12.0194 | | 85 | | 112 | | 96 | | 98 | |
| 12.0399 | | 86 | | 109 | | 95 | | 84 | |
| 12.0603 | | 85 | | 92 | | 97 | | 92 | |
| 12.0808 | | 83 | | 92 | | 76 | | 83 | |
| 12.1012 | | 84 | | 94 | | 87 | | 90 | |
| 12.1217 | | 89 | | 112 | | 91 | | 93 | |
| 12.1421 | | 99 | | 112 | | 86 | | 104 | |
| 12.1626 | | 97 | | 102 | | 87 | | 105 | |
| 12.183 | | 74 | | 118 | | 96 | | 106 | |
| 12.2035 | | 82 | | 86 | | 71 | | 83 | |
| 12.2239 | | 90 | | 120 | | 86 | | 95 | |
| 12.2444 | | 87 | | 89 | | 64 | | 104 | |
| 12.2648 | | 89 | | 111 | | 88 | | 68 | |
| 12.2853 | | 85 | | 92 | | 68 | | 100 | |
| 12.3057 | | 76 | | 87 | | 90 | | 88 | |
| 12.3262 | | 77 | | 108 | | 76 | | 84 | |
| 12.3466 | | 86 | | 92 | | 79 | | 91 | |
| 12.3671 | | 71 | | 109 | | 88 | | 118 | |
| 12.3875 | | 71 | | 96 | | 95 | | 90 | |
| 12.408 | | 93 | | 114 | | 94 | | 104 | |
| 12.4284 | | 83 | | 112 | | 81 | | 106 | |
| 12.4489 | | 106 | | 97 | | 83 | | 95 | |
| 12.4694 | | 67 | | 100 | | 78 | | 76 | |
| 12.4898 | | 85 | | 104 | | 100 | | 67 | |
| 12.5103 | | 70 | | 98 | | 82 | | 74 | |
| 12.5307 | | 100 | | 84 | | 85 | | 98 | |
| 12.5512 | | 95 | | 90 | | 111 | | 84 | |
| 12.5716 | | 95 | | 84 | | 109 | | 87 | |
| 12.5921 | | 90 | | 99 | | 83 | | 88 | |
| 12.6125 | | 74 | | 98 | | 87 | | 85 | |
| 12.633 | | 100 | | 107 | | 96 | | 94 | |
| 12.6534 | | 84 | | 103 | | 89 | | 83 | |
| 12.6739 | | 94 | | 98 | | 98 | | 92 | |
| 12.6943 | | 81 | | 107 | | 82 | | 84 | |
| 12.7148 | | 77 | | 129 | | 77 | | 92 | |
| 12.7352 | | 94 | | 103 | | 85 | | 100 | |
| 12.7557 | | 99 | | 86 | | 84 | | 79 | |
| 12.7761 | | 79 | | 101 | | 93 | | 73 | |
| 12.7966 | | 75 | | 96 | | 69 | | 109 | |
| 12.817 | | 74 | | 88 | | 100 | | 99 | |
| 12.8375 | | 72 | | 91 | | 86 | | 98 | |
| 12.8579 | | 82 | | 78 | | 93 | | 83 | |
| 12.8784 | | 82 | | 84 | | 78 | | 88 | |
| 12.8988 | | 87 | | 100 | | 71 | | 82 | |
| 12.9193 | | 90 | | 96 | | 94 | | 110 | |
| 12.9397 | | 85 | | 106 | | 82 | | 85 | |
| 12.9602 | | 86 | | 83 | | 83 | | 92 | |
| 12.9806 | | 82 | | 109 | | 76 | | 79 | |
| 13.0011 | | 80 | | 119 | | 73 | | 94 | |
| 13.0216 | | 77 | | 77 | | 78 | | 85 | |
| 13.042 | | 86 | | 85 | | 72 | | 93 | |
| 13.0625 | | 85 | | 111 | | 71 | | 81 | |
| 13.0829 | | 82 | | 113 | | 95 | | 82 | |
| 13.1034 | | 81 | | 90 | | 81 | | 90 | |
| 13.1238 | | 105 | | 103 | | 84 | | 94 | |
| 13.1443 | | 67 | | 97 | | 89 | | 84 | |
| 13.1647 | | 73 | | 109 | | 103 | | 89 | |
| 13.1852 | | 75 | | 107 | | 80 | | 84 | |
| 13.2056 | | 74 | | 90 | | 88 | | 92 | |
| 13.2261 | | 65 | | 97 | | 104 | | 77 | |
| 13.2465 | | 75 | | 117 | | 81 | | 73 | |
| 13.267 | | 87 | | 99 | | 91 | | 84 | |
| 13.2874 | | 82 | | 101 | | 85 | | 91 | |
| 13.3079 | | 65 | | 85 | | 100 | | 82 | |
| 13.3283 | | 83 | | 96 | | 82 | | 71 | |
| 13.3488 | | 86 | | 96 | | 70 | | 106 | |
| 13.3692 | | 94 | | 94 | | 83 | | 81 | |
| 13.3897 | | 73 | | 78 | | 91 | | 65 | |
| 13.4101 | | 76 | | 76 | | 76 | | 71 | |
| 13.4306 | | 78 | | 94 | | 77 | | 87 | |
| 13.451 | | 76 | | 115 | | 89 | | 87 | |
| 13.4715 | | 82 | | 90 | | 84 | | 65 | |
| 13.4919 | | 71 | | 92 | | 76 | | 79 | |
| 13.5124 | | 64 | | 97 | | 88 | | 82 | |
| 13.5329 | | 81 | | 83 | | 77 | | 88 | |
| 13.5533 | | 74 | | 91 | | 88 | | 87 | |
| 13.5738 | | 76 | | 111 | | 86 | | 83 | |
| 13.5942 | | 76 | | 96 | | 90 | | 101 | |
| 13.6147 | | 85 | | 108 | | 100 | | 77 | |
| 13.6351 | | 77 | | 92 | | 72 | | 74 | |
| 13.6556 | | 80 | | 120 | | 83 | | 86 | |
| 13.676 | | 88 | | 98 | | 69 | | 90 | |
| 13.6965 | | 61 | | 93 | | 72 | | 86 | |
| 13.7169 | | 85 | | 88 | | 74 | | 100 | |
| 13.7374 | | 75 | | 89 | | 81 | | 92 | |
| 13.7578 | | 81 | | 84 | | 71 | | 77 | |
| 13.7783 | | 77 | | 91 | | 69 | | 86 | |
| 13.7987 | | 69 | | 81 | | 101 | | 79 | |
| 13.8192 | | 86 | | 99 | | 77 | | 82 | |
| 13.8396 | | 81 | | 109 | | 85 | | 86 | |
| 13.8601 | | 63 | | 98 | | 82 | | 72 | |
| 13.8805 | | 78 | | 114 | | 71 | | 85 | |
| 13.901 | | 66 | | 85 | | 80 | | 65 | |
| 13.9214 | | 76 | | 80 | | 81 | | 70 | |
| 13.9419 | | 90 | | 99 | | 91 | | 81 | |
| 13.9623 | | 77 | | 99 | | 79 | | 72 | |
| 13.9828 | | 64 | | 109 | | 88 | | 68 | |
| 14.0032 | | 83 | | 94 | | 79 | | 100 | |
| 14.0237 | | 67 | | 83 | | 67 | | 87 | |
| 14.0442 | | 80 | | 86 | | 76 | | 86 | |
| 14.0646 | | 93 | | 93 | | 84 | | 79 | |
| 14.0851 | | 88 | | 106 | | 64 | | 81 | |
| 14.1055 | | 67 | | 89 | | 81 | | 76 | |
| 14.126 | | 85 | | 86 | | 69 | | 74 | |
| 14.1464 | | 69 | | 78 | | 86 | | 94 | |
| 14.1669 | | 67 | | 81 | | 88 | | 87 | |
| 14.1873 | | 71 | | 71 | | 70 | | 88 | |
| 14.2078 | | 82 | | 103 | | 77 | | 95 | |
| 14.2282 | | 78 | | 87 | | 91 | | 77 | |
| 14.2487 | | 82 | | 75 | | 80 | | 72 | |
| 14.2691 | | 77 | | 98 | | 63 | | 87 | |
| 14.2896 | | 63 | | 90 | | 81 | | 88 | |
| 14.31 | | 64 | | 90 | | 81 | | 72 | |
| 14.3305 | | 77 | | 88 | | 85 | | 92 | |
| 14.3509 | | 74 | | 87 | | 75 | | 74 | |
| 14.3714 | | 68 | | 76 | | 67 | | 78 | |
| 14.3918 | | 69 | | 81 | | 80 | | 81 | |
| 14.4123 | | 77 | | 83 | | 94 | | 83 | |
| 14.4327 | | 82 | | 91 | | 78 | | 92 | |
| 14.4532 | | 92 | | 83 | | 76 | | 91 | |
| 14.4736 | | 81 | | 88 | | 69 | | 86 | |
| 14.4941 | | 72 | | 84 | | 82 | | 80 | |
| 14.5145 | | 75 | | 97 | | 84 | | 86 | |
| 14.535 | | 74 | | 83 | | 79 | | 74 | |
| 14.5554 | | 72 | | 81 | | 75 | | 90 | |
| 14.5759 | | 78 | | 99 | | 74 | | 61 | |
| 14.5964 | | 82 | | 85 | | 64 | | 76 | |
| 14.6168 | | 75 | | 86 | | 82 | | 80 | |
| 14.6373 | | 84 | | 88 | | 75 | | 89 | |
| 14.6577 | | 57 | | 88 | | 80 | | 78 | |
| 14.6782 | | 93 | | 77 | | 94 | | 77 | |
| 14.6986 | | 64 | | 95 | | 84 | | 90 | |
| 14.7191 | | 89 | | 71 | | 79 | | 75 | |
| 14.7395 | | 66 | | 93 | | 79 | | 65 | |
| 14.76 | | 75 | | 81 | | 72 | | 77 | |
| 14.7804 | | 69 | | 70 | | 74 | | 86 | |
| 14.8009 | | 67 | | 100 | | 86 | | 71 | |
| 14.8213 | | 68 | | 90 | | 85 | | 53 | |
| 14.8418 | | 91 | | 109 | | 91 | | 94 | |
| 14.8622 | | 85 | | 71 | | 88 | | 86 | |
| 14.8827 | | 81 | | 103 | | 91 | | 82 | |
| 14.9031 | | 92 | | 97 | | 75 | | 70 | |
| 14.9236 | | 65 | | 97 | | 87 | | 75 | |
| 14.944 | | 82 | | 103 | | 62 | | 85 | |
| 14.9645 | | 78 | | 90 | | 78 | | 74 | |
| 14.9849 | | 67 | | 88 | | 67 | | 82 | |
| 15.0054 | | 78 | | 76 | | 57 | | 80 | |
| 15.0258 | | 75 | | 93 | | 87 | | 81 | |
| 15.0463 | | 103 | | 92 | | 90 | | 79 | |
| 15.0667 | | 74 | | 103 | | 84 | | 90 | |
| 15.0872 | | 70 | | 94 | | 87 | | 86 | |
| 15.1077 | | 95 | | 106 | | 80 | | 85 | |
| 15.1281 | | 86 | | 92 | | 92 | | 74 | |
| 15.1486 | | 78 | | 89 | | 66 | | 87 | |
| 15.169 | | 78 | | 73 | | 77 | | 68 | |
| 15.1895 | | 69 | | 97 | | 90 | | 85 | |
| 15.2099 | | 68 | | 98 | | 77 | | 67 | |
| 15.2304 | | 89 | | 85 | | 80 | | 80 | |
| 15.2508 | | 68 | | 85 | | 82 | | 70 | |
| 15.2713 | | 63 | | 78 | | 94 | | 83 | |
| 15.2917 | | 57 | | 81 | | 73 | | 97 | |
| 15.3122 | | 67 | | 103 | | 90 | | 71 | |
| 15.3326 | | 76 | | 87 | | 76 | | 66 | |
| 15.3531 | | 78 | | 87 | | 80 | | 90 | |
| 15.3735 | | 89 | | 91 | | 80 | | 74 | |
| 15.394 | | 90 | | 85 | | 73 | | 64 | |
| 15.4144 | | 68 | | 94 | | 89 | | 72 | |
| 15.4349 | | 71 | | 75 | | 80 | | 80 | |
| 15.4553 | | 83 | | 81 | | 87 | | 76 | |
| 15.4758 | | 67 | | 98 | | 83 | | 90 | |
| 15.4962 | | 72 | | 78 | | 79 | | 73 | |
| 15.5167 | | 78 | | 85 | | 81 | | 91 | |
| 15.5371 | | 93 | | 73 | | 82 | | 72 | |
| 15.5576 | | 81 | | 81 | | 68 | | 72 | |
| 15.578 | | 76 | | 93 | | 76 | | 79 | |
| 15.5985 | | 70 | | 83 | | 79 | | 82 | |
| 15.6189 | | 65 | | 90 | | 69 | | 71 | |
| 15.6394 | | 62 | | 81 | | 88 | | 57 | |
| 15.6599 | | 75 | | 92 | | 79 | | 77 | |
| 15.6803 | | 81 | | 92 | | 89 | | 80 | |
| 15.7008 | | 76 | | 100 | | 73 | | 72 | |
| 15.7212 | | 66 | | 82 | | 83 | | 87 | |
| 15.7417 | | 55 | | 91 | | 83 | | 71 | |
| 15.7621 | | 83 | | 102 | | 67 | | 71 | |
| 15.7826 | | 72 | | 87 | | 78 | | 86 | |
| 15.803 | | 84 | | 97 | | 56 | | 82 | |
| 15.8235 | | 68 | | 96 | | 80 | | 91 | |
| 15.8439 | | 85 | | 102 | | 82 | | 75 | |
| 15.8644 | | 69 | | 89 | | 86 | | 78 | |
| 15.8848 | | 74 | | 105 | | 88 | | 82 | |
| 15.9053 | | 85 | | 108 | | 85 | | 84 | |
| 15.9257 | | 78 | | 84 | | 92 | | 75 | |
| 15.9462 | | 64 | | 102 | | 94 | | 78 | |
| 15.9666 | | 88 | | 108 | | 72 | | 64 | |
| 15.9871 | | 83 | | 88 | | 87 | | 82 | |
| 16.0075 | | 84 | | 111 | | 71 | | 63 | |
| 16.028 | | 72 | | 93 | | 81 | | 81 | |
| 16.0484 | | 76 | | 96 | | 77 | | 83 | |
| 16.0689 | | 92 | | 94 | | 81 | | 89 | |
| 16.0893 | | 75 | | 92 | | 83 | | 81 | |
| 16.1098 | | 93 | | 80 | | 85 | | 76 | |
| 16.1302 | | 59 | | 90 | | 72 | | 75 | |
| 16.1507 | | 86 | | 79 | | 74 | | 93 | |
| 16.1712 | | 82 | | 80 | | 80 | | 75 | |
| 16.1916 | | 77 | | 96 | | 91 | | 81 | |
| 16.2121 | | 82 | | 96 | | 74 | | 79 | |
| 16.2325 | | 71 | | 99 | | 91 | | 78 | |
| 16.253 | | 74 | | 76 | | 87 | | 84 | |
| 16.2734 | | 78 | | 88 | | 67 | | 68 | |
| 16.2939 | | 81 | | 112 | | 78 | | 81 | |
| 16.3143 | | 77 | | 78 | | 75 | | 79 | |
| 16.3348 | | 99 | | 89 | | 76 | | 76 | |
| 16.3552 | | 60 | | 95 | | 81 | | 88 | |
| 16.3757 | | 77 | | 87 | | 85 | | 100 | |
| 16.3961 | | 81 | | 108 | | 80 | | 83 | |
| 16.4166 | | 74 | | 101 | | 78 | | 84 | |
| 16.437 | | 77 | | 96 | | 70 | | 86 | |
| 16.4575 | | 96 | | 87 | | 80 | | 81 | |
| 16.4779 | | 69 | | 115 | | 83 | | 73 | |
| 16.4984 | | 60 | | 98 | | 81 | | 88 | |
| 16.5188 | | 73 | | 85 | | 84 | | 89 | |
| 16.5393 | | 75 | | 101 | | 78 | | 77 | |
| 16.5597 | | 84 | | 90 | | 86 | | 78 | |
| 16.5802 | | 82 | | 94 | | 89 | | 89 | |
| 16.6006 | | 90 | | 97 | | 84 | | 78 | |
| 16.6211 | | 88 | | 114 | | 63 | | 94 | |
| 16.6415 | | 88 | | 102 | | 83 | | 78 | |
| 16.662 | | 78 | | 100 | | 92 | | 75 | |
| 16.6825 | | 82 | | 85 | | 88 | | 90 | |
| 16.7029 | | 59 | | 82 | | 81 | | 88 | |
| 16.7234 | | 98 | | 99 | | 78 | | 84 | |
| 16.7438 | | 66 | | 94 | | 80 | | 84 | |
| 16.7643 | | 66 | | 80 | | 76 | | 80 | |
| 16.7847 | | 82 | | 100 | | 82 | | 106 | |
| 16.8052 | | 96 | | 87 | | 80 | | 78 | |
| 16.8256 | | 86 | | 83 | | 93 | | 70 | |
| 16.8461 | | 99 | | 104 | | 73 | | 89 | |
| 16.8665 | | 90 | | 84 | | 93 | | 83 | |
| 16.887 | | 76 | | 89 | | 95 | | 80 | |
| 16.9074 | | 72 | | 96 | | 77 | | 84 | |
| 16.9279 | | 85 | | 99 | | 64 | | 69 | |
| 16.9483 | | 103 | | 99 | | 93 | | 83 | |
| 16.9688 | | 76 | | 86 | | 93 | | 88 | |
| 16.9892 | | 71 | | 89 | | 81 | | 69 | |
| 17.0097 | | 77 | | 89 | | 86 | | 70 | |
| 17.0301 | | 77 | | 90 | | 83 | | 92 | |
| 17.0506 | | 88 | | 99 | | 94 | | 87 | |
| 17.071 | | 81 | | 102 | | 81 | | 70 | |
| 17.0915 | | 80 | | 86 | | 83 | | 66 | |
| 17.1119 | | 83 | | 103 | | 95 | | 77 | |
| 17.1324 | | 90 | | 96 | | 83 | | 87 | |
| 17.1528 | | 85 | | 88 | | 92 | | 75 | |
| 17.1733 | | 108 | | 91 | | 84 | | 84 | |
| 17.1937 | | 70 | | 78 | | 79 | | 90 | |
| 17.2142 | | 91 | | 111 | | 93 | | 84 | |
| 17.2347 | | 84 | | 96 | | 86 | | 86 | |
| 17.2551 | | 73 | | 112 | | 89 | | 85 | |
| 17.2756 | | 79 | | 104 | | 63 | | 84 | |
| 17.296 | | 84 | | 90 | | 90 | | 79 | |
| 17.3165 | | 90 | | 95 | | 85 | | 75 | |
| 17.3369 | | 85 | | 107 | | 114 | | 92 | |
| 17.3574 | | 82 | | 106 | | 82 | | 77 | |
| 17.3778 | | 67 | | 104 | | 83 | | 88 | |
| 17.3983 | | 86 | | 99 | | 80 | | 66 | |
| 17.4187 | | 83 | | 101 | | 78 | | 85 | |
| 17.4392 | | 90 | | 78 | | 98 | | 101 | |
| 17.4596 | | 77 | | 95 | | 92 | | 85 | |
| 17.4801 | | 90 | | 87 | | 86 | | 81 | |
| 17.5005 | | 84 | | 97 | | 77 | | 71 | |
| 17.521 | | 101 | | 94 | | 102 | | 95 | |
| 17.5414 | | 71 | | 109 | | 78 | | 85 | |
| 17.5619 | | 88 | | 89 | | 78 | | 96 | |
| 17.5823 | | 83 | | 101 | | 84 | | 69 | |
| 17.6028 | | 88 | | 79 | | 92 | | 87 | |
| 17.6232 | | 88 | | 93 | | 80 | | 83 | |
| 17.6437 | | 72 | | 88 | | 89 | | 83 | |
| 17.6641 | | 90 | | 103 | | 103 | | 99 | |
| 17.6846 | | 75 | | 121 | | 76 | | 90 | |
| 17.705 | | 90 | | 108 | | 86 | | 91 | |
| 17.7255 | | 77 | | 101 | | 93 | | 84 | |
| 17.746 | | 82 | | 109 | | 85 | | 91 | |
| 17.7664 | | 72 | | 104 | | 98 | | 88 | |
| 17.7869 | | 77 | | 110 | | 81 | | 79 | |
| 17.8073 | | 97 | | 103 | | 85 | | 88 | |
| 17.8278 | | 81 | | 93 | | 89 | | 93 | |
| 17.8482 | | 86 | | 93 | | 103 | | 78 | |
| 17.8687 | | 89 | | 68 | | 75 | | 69 | |
| 17.8891 | | 95 | | 96 | | 82 | | 85 | |
| 17.9096 | | 80 | | 104 | | 88 | | 92 | |
| 17.93 | | 77 | | 109 | | 100 | | 79 | |
| 17.9505 | | 69 | | 110 | | 103 | | 77 | |
| 17.9709 | | 87 | | 104 | | 81 | | 89 | |
| 17.9914 | | 70 | | 89 | | 88 | | 85 | |
| 18.0118 | | 72 | | 106 | | 84 | | 105 | |
| 18.0323 | | 90 | | 103 | | 98 | | 97 | |
| 18.0527 | | 76 | | 89 | | 75 | | 92 | |
| 18.0732 | | 82 | | 112 | | 110 | | 84 | |
| 18.0936 | | 86 | | 86 | | 72 | | 96 | |
| 18.1141 | | 80 | | 111 | | 108 | | 97 | |
| 18.1345 | | 77 | | 94 | | 90 | | 86 | |
| 18.155 | | 88 | | 108 | | 94 | | 97 | |
| 18.1754 | | 88 | | 90 | | 93 | | 73 | |
| 18.1959 | | 92 | | 106 | | 92 | | 105 | |
| 18.2163 | | 71 | | 105 | | 92 | | 82 | |
| 18.2368 | | 96 | | 97 | | 85 | | 83 | |
| 18.2573 | | 87 | | 107 | | 100 | | 84 | |
| 18.2777 | | 96 | | 106 | | 81 | | 78 | |
| 18.2982 | | 80 | | 110 | | 100 | | 72 | |
| 18.3186 | | 92 | | 91 | | 85 | | 87 | |
| 18.3391 | | 83 | | 111 | | 101 | | 87 | |
| 18.3595 | | 94 | | 100 | | 93 | | 72 | |
| 18.38 | | 73 | | 105 | | 96 | | 76 | |
| 18.4004 | | 69 | | 97 | | 89 | | 81 | |
| 18.4209 | | 87 | | 110 | | 103 | | 86 | |
| 18.4413 | | 89 | | 86 | | 88 | | 84 | |
| 18.4618 | | 82 | | 112 | | 95 | | 84 | |
| 18.4822 | | 101 | | 104 | | 106 | | 82 | |
| 18.5027 | | 75 | | 115 | | 88 | | 96 | |
| 18.5231 | | 86 | | 113 | | 83 | | 86 | |
| 18.5436 | | 71 | | 133 | | 92 | | 72 | |
| 18.564 | | 91 | | 127 | | 93 | | 97 | |
| 18.5845 | | 87 | | 108 | | 96 | | 87 | |
| 18.6049 | | 94 | | 87 | | 93 | | 88 | |
| 18.6254 | | 119 | | 104 | | 107 | | 99 | |
| 18.6458 | | 113 | | 100 | | 103 | | 89 | |
| 18.6663 | | 98 | | 101 | | 104 | | 104 | |
| 18.6867 | | 99 | | 106 | | 123 | | 121 | |
| 18.7072 | | 117 | | 125 | | 138 | | 110 | |
| 18.7276 | | 87 | | 133 | | 113 | | 127 | |
| 18.7481 | | 125 | | 143 | | 143 | | 139 | |
| 18.7685 | | 147 | | 155 | | 150 | | 145 | |
| 18.789 | | 241 | | 235 | | 236 | | 225 | |
| 18.8095 | | 335 | | 294 | | 328 | | 295 | |
| 18.8299 | | 322 | | 346 | | 317 | | 269 | |
| 18.8504 | | 206 | | 230 | | 183 | | 171 | |
| 18.8708 | | 123 | | 139 | | 118 | | 140 | |
| 18.8913 | | 107 | | 135 | | 119 | | 107 | |
| 18.9117 | | 103 | | 108 | | 126 | | 105 | |
| 18.9322 | | 107 | | 113 | | 112 | | 92 | |
| 18.9526 | | 103 | | 114 | | 124 | | 109 | |
| 18.9731 | | 106 | | 114 | | 92 | | 100 | |
| 18.9935 | | 106 | | 107 | | 90 | | 110 | |
| 19.014 | | 111 | | 113 | | 102 | | 108 | |
| 19.0344 | | 102 | | 140 | | 116 | | 83 | |
| 19.0549 | | 107 | | 106 | | 114 | | 121 | |
| 19.0753 | | 106 | | 142 | | 111 | | 111 | |
| 19.0958 | | 111 | | 136 | | 95 | | 99 | |
| 19.1162 | | 105 | | 122 | | 114 | | 80 | |
| 19.1367 | | 84 | | 123 | | 106 | | 86 | |
| 19.1571 | | 113 | | 134 | | 112 | | 104 | |
| 19.1776 | | 89 | | 113 | | 106 | | 99 | |
| 19.198 | | 94 | | 116 | | 115 | | 127 | |
| 19.2185 | | 102 | | 125 | | 123 | | 105 | |
| 19.2389 | | 81 | | 95 | | 112 | | 102 | |
| 19.2594 | | 104 | | 125 | | 110 | | 110 | |
| 19.2798 | | 85 | | 119 | | 131 | | 113 | |
| 19.3003 | | 104 | | 115 | | 115 | | 98 | |
| 19.3208 | | 111 | | 131 | | 106 | | 96 | |
| 19.3412 | | 113 | | 112 | | 107 | | 108 | |
| 19.3617 | | 125 | | 104 | | 120 | | 111 | |
| 19.3821 | | 110 | | 116 | | 84 | | 121 | |
| 19.4026 | | 104 | | 102 | | 117 | | 127 | |
| 19.423 | | 111 | | 110 | | 107 | | 124 | |
| 19.4435 | | 93 | | 109 | | 109 | | 108 | |
| 19.4639 | | 106 | | 122 | | 122 | | 103 | |
| 19.4844 | | 133 | | 144 | | 107 | | 103 | |
| 19.5048 | | 101 | | 129 | | 108 | | 103 | |
| 19.5253 | | 119 | | 152 | | 130 | | 102 | |
| 19.5457 | | 94 | | 125 | | 110 | | 114 | |
| 19.5662 | | 113 | | 131 | | 115 | | 108 | |
| 19.5866 | | 118 | | 135 | | 134 | | 111 | |
| 19.6071 | | 104 | | 114 | | 122 | | 111 | |
| 19.6275 | | 115 | | 100 | | 106 | | 105 | |
| 19.648 | | 102 | | 124 | | 127 | | 109 | |
| 19.6684 | | 109 | | 128 | | 126 | | 127 | |
| 19.6889 | | 100 | | 138 | | 100 | | 119 | |
| 19.7093 | | 107 | | 140 | | 124 | | 120 | |
| 19.7298 | | 122 | | 119 | | 106 | | 131 | |
| 19.7502 | | 131 | | 146 | | 117 | | 105 | |
| 19.7707 | | 109 | | 143 | | 118 | | 111 | |
| 19.7911 | | 125 | | 131 | | 136 | | 115 | |
| 19.8116 | | 120 | | 120 | | 138 | | 98 | |
| 19.8321 | | 126 | | 130 | | 128 | | 123 | |
| 19.8525 | | 118 | | 136 | | 122 | | 110 | |
| 19.873 | | 130 | | 140 | | 134 | | 135 | |
| 19.8934 | | 126 | | 127 | | 140 | | 117 | |
| 19.9139 | | 126 | | 130 | | 140 | | 131 | |
| 19.9343 | | 121 | | 157 | | 151 | | 135 | |
| 19.9548 | | 139 | | 144 | | 164 | | 138 | |
| 19.9752 | | 168 | | 178 | | 189 | | 166 | |
| 19.9957 | | 161 | | 169 | | 152 | | 132 | |
| 20.0161 | | 124 | | 135 | | 134 | | 154 | |
| 20.0366 | | 120 | | 131 | | 126 | | 120 | |
| 20.057 | | 119 | | 148 | | 132 | | 131 | |
| 20.0775 | | 95 | | 137 | | 148 | | 142 | |
| 20.0979 | | 118 | | 177 | | 133 | | 120 | |
| 20.1184 | | 131 | | 146 | | 123 | | 137 | |
| 20.1388 | | 139 | | 136 | | 151 | | 131 | |
| 20.1593 | | 102 | | 120 | | 148 | | 118 | |
| 20.1797 | | 140 | | 148 | | 137 | | 126 | |
| 20.2002 | | 130 | | 124 | | 141 | | 124 | |
| 20.2206 | | 142 | | 176 | | 139 | | 125 | |
| 20.2411 | | 133 | | 124 | | 142 | | 139 | |
| 20.2615 | | 129 | | 143 | | 126 | | 120 | |
| 20.282 | | 146 | | 151 | | 153 | | 129 | |
| 20.3024 | | 130 | | 167 | | 159 | | 131 | |
| 20.3229 | | 139 | | 140 | | 119 | | 153 | |
| 20.3433 | | 122 | | 166 | | 138 | | 141 | |
| 20.3638 | | 129 | | 158 | | 130 | | 125 | |
| 20.3843 | | 115 | | 165 | | 158 | | 135 | |
| 20.4047 | | 142 | | 147 | | 135 | | 138 | |
| 20.4252 | | 134 | | 166 | | 158 | | 144 | |
| 20.4456 | | 136 | | 125 | | 174 | | 152 | |
| 20.4661 | | 161 | | 166 | | 153 | | 133 | |
| 20.4865 | | 146 | | 176 | | 149 | | 135 | |
| 20.507 | | 160 | | 172 | | 163 | | 173 | |
| 20.5274 | | 154 | | 189 | | 163 | | 165 | |
| 20.5479 | | 164 | | 211 | | 162 | | 169 | |
| 20.5683 | | 178 | | 178 | | 172 | | 183 | |
| 20.5888 | | 179 | | 206 | | 200 | | 183 | |
| 20.6092 | | 190 | | 235 | | 189 | | 217 | |
| 20.6297 | | 194 | | 259 | | 211 | | 239 | |
| 20.6501 | | 225 | | 242 | | 270 | | 269 | |
| 20.6706 | | 236 | | 326 | | 280 | | 303 | |
| 20.691 | | 335 | | 384 | | 340 | | 351 | |
| 20.7115 | | 424 | | 399 | | 410 | | 442 | |
| 20.7319 | | 575 | | 588 | | 556 | | 603 | |
| 20.7524 | | 858 | | 829 | | 803 | | 872 | |
| 20.7728 | | 1355 | | 1209 | | 1254 | | 1439 | |
| 20.7933 | | 2440 | | 2010 | | 2266 | | 2647 | |
| 20.8137 | | 4733 | | 3852 | | 4193 | | 4957 | |
| 20.8342 | | 9695 | | 7219 | | 7454 | | 8408 | |
| 20.8546 | | 11536 | | 9930 | | 9512 | | 9590 | |
| 20.8751 | | 6725 | | 6690 | | 6078 | | 6372 | |
| 20.8956 | | 6295 | | 5446 | | 5081 | | 5445 | |
| 20.916 | | 4356 | | 4489 | | 3964 | | 3864 | |
| 20.9365 | | 1356 | | 1756 | | 1470 | | 1457 | |
| 20.9569 | | 633 | | 793 | | 737 | | 719 | |
| 20.9774 | | 455 | | 516 | | 532 | | 503 | |
| 20.9978 | | 349 | | 387 | | 395 | | 396 | |
| 21.0183 | | 329 | | 321 | | 318 | | 342 | |
| 21.0387 | | 239 | | 294 | | 267 | | 239 | |
| 21.0592 | | 212 | | 222 | | 261 | | 239 | |
| 21.0796 | | 222 | | 247 | | 223 | | 221 | |
| 21.1001 | | 197 | | 224 | | 214 | | 198 | |
| 21.1205 | | 189 | | 187 | | 197 | | 202 | |
| 21.141 | | 179 | | 190 | | 185 | | 174 | |
| 21.1614 | | 157 | | 191 | | 166 | | 148 | |
| 21.1819 | | 160 | | 166 | | 165 | | 169 | |
| 21.2023 | | 147 | | 184 | | 154 | | 147 | |
| 21.2228 | | 149 | | 160 | | 170 | | 147 | |
| 21.2432 | | 135 | | 162 | | 139 | | 155 | |
| 21.2637 | | 162 | | 162 | | 113 | | 134 | |
| 21.2841 | | 160 | | 159 | | 125 | | 137 | |
| 21.3046 | | 122 | | 142 | | 144 | | 127 | |
| 21.325 | | 125 | | 131 | | 142 | | 122 | |
| 21.3455 | | 126 | | 160 | | 148 | | 129 | |
| 21.3659 | | 105 | | 171 | | 145 | | 135 | |
| 21.3864 | | 139 | | 143 | | 129 | | 146 | |
| 21.4069 | | 132 | | 135 | | 141 | | 123 | |
| 21.4273 | | 129 | | 147 | | 140 | | 129 | |
| 21.4478 | | 126 | | 166 | | 144 | | 142 | |
| 21.4682 | | 109 | | 140 | | 120 | | 113 | |
| 21.4887 | | 117 | | 128 | | 132 | | 123 | |
| 21.5091 | | 113 | | 118 | | 132 | | 114 | |
| 21.5296 | | 100 | | 143 | | 139 | | 115 | |
| 21.55 | | 107 | | 119 | | 138 | | 128 | |
| 21.5705 | | 118 | | 122 | | 124 | | 124 | |
| 21.5909 | | 105 | | 130 | | 136 | | 103 | |
| 21.6114 | | 114 | | 137 | | 134 | | 123 | |
| 21.6318 | | 141 | | 150 | | 110 | | 103 | |
| 21.6523 | | 111 | | 134 | | 142 | | 120 | |
| 21.6727 | | 99 | | 117 | | 105 | | 116 | |
| 21.6932 | | 123 | | 101 | | 130 | | 99 | |
| 21.7136 | | 96 | | 132 | | 139 | | 77 | |
| 21.7341 | | 96 | | 130 | | 136 | | 122 | |
| 21.7545 | | 126 | | 137 | | 113 | | 115 | |
| 21.775 | | 100 | | 127 | | 134 | | 126 | |
| 21.7954 | | 99 | | 154 | | 117 | | 101 | |
| 21.8159 | | 115 | | 138 | | 108 | | 112 | |
| 21.8363 | | 99 | | 128 | | 118 | | 107 | |
| 21.8568 | | 105 | | 114 | | 122 | | 86 | |
| 21.8772 | | 120 | | 132 | | 130 | | 117 | |
| 21.8977 | | 118 | | 131 | | 102 | | 103 | |
| 21.9181 | | 107 | | 125 | | 121 | | 121 | |
| 21.9386 | | 116 | | 142 | | 115 | | 91 | |
| 21.9591 | | 120 | | 134 | | 119 | | 109 | |
| 21.9795 | | 119 | | 131 | | 111 | | 122 | |
| 22 | | 107 | | 113 | | 110 | | 98 | |
| 22.0204 | | 112 | | 140 | | 108 | | 113 | |
| 22.0409 | | 96 | | 124 | | 118 | | 127 | |
| 22.0613 | | 133 | | 106 | | 117 | | 84 | |
| 22.0818 | | 103 | | 102 | | 113 | | 88 | |
| 22.1022 | | 99 | | 128 | | 124 | | 98 | |
| 22.1227 | | 88 | | 119 | | 109 | | 112 | |
| 22.1431 | | 110 | | 129 | | 111 | | 93 | |
| 22.1636 | | 99 | | 108 | | 108 | | 105 | |
| 22.184 | | 102 | | 130 | | 114 | | 102 | |
| 22.2045 | | 100 | | 124 | | 126 | | 110 | |
| 22.2249 | | 99 | | 123 | | 98 | | 100 | |
| 22.2454 | | 102 | | 127 | | 113 | | 102 | |
| 22.2658 | | 92 | | 126 | | 117 | | 104 | |
| 22.2863 | | 91 | | 127 | | 104 | | 92 | |
| 22.3067 | | 102 | | 109 | | 91 | | 97 | |
| 22.3272 | | 91 | | 119 | | 103 | | 93 | |
| 22.3476 | | 93 | | 104 | | 111 | | 91 | |
| 22.3681 | | 106 | | 150 | | 115 | | 84 | |
| 22.3885 | | 93 | | 105 | | 108 | | 103 | |
| 22.409 | | 98 | | 130 | | 110 | | 95 | |
| 22.4294 | | 89 | | 98 | | 104 | | 97 | |
| 22.4499 | | 107 | | 119 | | 105 | | 94 | |
| 22.4704 | | 94 | | 97 | | 110 | | 109 | |
| 22.4908 | | 91 | | 119 | | 112 | | 105 | |
| 22.5113 | | 97 | | 105 | | 110 | | 112 | |
| 22.5317 | | 91 | | 107 | | 100 | | 106 | |
| 22.5522 | | 89 | | 102 | | 105 | | 100 | |
| 22.5726 | | 96 | | 102 | | 105 | | 99 | |
| 22.5931 | | 96 | | 115 | | 110 | | 98 | |
| 22.6135 | | 97 | | 115 | | 100 | | 86 | |
| 22.634 | | 88 | | 105 | | 112 | | 92 | |
| 22.6544 | | 85 | | 128 | | 122 | | 87 | |
| 22.6749 | | 117 | | 98 | | 95 | | 93 | |
| 22.6953 | | 88 | | 102 | | 91 | | 99 | |
| 22.7158 | | 92 | | 100 | | 98 | | 85 | |
| 22.7362 | | 76 | | 126 | | 90 | | 100 | |
| 22.7567 | | 106 | | 128 | | 109 | | 102 | |
| 22.7771 | | 103 | | 114 | | 112 | | 101 | |
| 22.7976 | | 89 | | 118 | | 74 | | 120 | |
| 22.818 | | 83 | | 96 | | 107 | | 92 | |
| 22.8385 | | 89 | | 117 | | 94 | | 96 | |
| 22.8589 | | 96 | | 100 | | 100 | | 93 | |
| 22.8794 | | 83 | | 143 | | 97 | | 82 | |
| 22.8998 | | 85 | | 107 | | 103 | | 82 | |
| 22.9203 | | 69 | | 115 | | 117 | | 102 | |
| 22.9407 | | 91 | | 126 | | 89 | | 98 | |
| 22.9612 | | 108 | | 116 | | 104 | | 92 | |
| 22.9816 | | 81 | | 115 | | 108 | | 107 | |
| 23.0021 | | 107 | | 114 | | 106 | | 96 | |
| 23.0226 | | 87 | | 97 | | 107 | | 89 | |
| 23.043 | | 100 | | 111 | | 98 | | 91 | |
| 23.0635 | | 94 | | 102 | | 106 | | 73 | |
| 23.0839 | | 83 | | 99 | | 101 | | 97 | |
| 23.1044 | | 90 | | 108 | | 89 | | 88 | |
| 23.1248 | | 66 | | 106 | | 89 | | 82 | |
| 23.1453 | | 75 | | 103 | | 99 | | 87 | |
| 23.1657 | | 109 | | 87 | | 114 | | 107 | |
| 23.1862 | | 86 | | 97 | | 114 | | 92 | |
| 23.2066 | | 78 | | 103 | | 122 | | 90 | |
| 23.2271 | | 87 | | 108 | | 103 | | 91 | |
| 23.2475 | | 73 | | 103 | | 104 | | 93 | |
| 23.268 | | 94 | | 108 | | 88 | | 103 | |
| 23.2884 | | 93 | | 115 | | 107 | | 103 | |
| 23.3089 | | 99 | | 108 | | 98 | | 82 | |
| 23.3293 | | 92 | | 120 | | 101 | | 88 | |
| 23.3498 | | 80 | | 98 | | 101 | | 101 | |
| 23.3702 | | 94 | | 101 | | 90 | | 114 | |
| 23.3907 | | 94 | | 99 | | 115 | | 79 | |
| 23.4111 | | 72 | | 105 | | 113 | | 94 | |
| 23.4316 | | 89 | | 125 | | 94 | | 83 | |
| 23.452 | | 108 | | 103 | | 85 | | 91 | |
| 23.4725 | | 83 | | 112 | | 93 | | 81 | |
| 23.4929 | | 86 | | 98 | | 97 | | 83 | |
| 23.5134 | | 89 | | 101 | | 86 | | 101 | |
| 23.5339 | | 93 | | 96 | | 99 | | 77 | |
| 23.5543 | | 92 | | 97 | | 91 | | 100 | |
| 23.5748 | | 92 | | 109 | | 90 | | 91 | |
| 23.5952 | | 95 | | 94 | | 80 | | 88 | |
| 23.6157 | | 88 | | 101 | | 104 | | 102 | |
| 23.6361 | | 84 | | 101 | | 110 | | 90 | |
| 23.6566 | | 88 | | 101 | | 100 | | 86 | |
| 23.677 | | 111 | | 105 | | 90 | | 107 | |
| 23.6975 | | 83 | | 102 | | 107 | | 79 | |
| 23.7179 | | 73 | | 114 | | 103 | | 88 | |
| 23.7384 | | 85 | | 97 | | 95 | | 103 | |
| 23.7588 | | 93 | | 110 | | 126 | | 107 | |
| 23.7793 | | 125 | | 113 | | 113 | | 104 | |
| 23.7997 | | 88 | | 128 | | 106 | | 121 | |
| 23.8202 | | 110 | | 132 | | 130 | | 137 | |
| 23.8406 | | 115 | | 122 | | 148 | | 108 | |
| 23.8611 | | 123 | | 139 | | 131 | | 127 | |
| 23.8815 | | 130 | | 135 | | 138 | | 130 | |
| 23.902 | | 123 | | 148 | | 139 | | 146 | |
| 23.9224 | | 136 | | 166 | | 157 | | 169 | |
| 23.9429 | | 220 | | 229 | | 218 | | 214 | |
| 23.9633 | | 311 | | 353 | | 361 | | 362 | |
| 23.9838 | | 638 | | 623 | | 621 | | 700 | |
| 24.0042 | | 1077 | | 988 | | 1054 | | 1100 | |
| 24.0247 | | 1299 | | 1289 | | 1333 | | 1393 | |
| 24.0452 | | 659 | | 934 | | 846 | | 833 | |
| 24.0656 | | 308 | | 378 | | 316 | | 369 | |
| 24.0861 | | 161 | | 227 | | 215 | | 213 | |
| 24.1065 | | 160 | | 186 | | 147 | | 152 | |
| 24.127 | | 149 | | 158 | | 156 | | 169 | |
| 24.1474 | | 135 | | 168 | | 151 | | 155 | |
| 24.1679 | | 113 | | 159 | | 150 | | 127 | |
| 24.1883 | | 136 | | 146 | | 98 | | 121 | |
| 24.2088 | | 114 | | 133 | | 138 | | 128 | |
| 24.2292 | | 110 | | 137 | | 129 | | 131 | |
| 24.2497 | | 100 | | 147 | | 113 | | 106 | |
| 24.2701 | | 120 | | 128 | | 96 | | 110 | |
| 24.2906 | | 97 | | 158 | | 132 | | 107 | |
| 24.311 | | 119 | | 111 | | 118 | | 110 | |
| 24.3315 | | 122 | | 116 | | 120 | | 116 | |
| 24.3519 | | 100 | | 140 | | 107 | | 119 | |
| 24.3724 | | 92 | | 118 | | 113 | | 127 | |
| 24.3928 | | 115 | | 111 | | 138 | | 105 | |
| 24.4133 | | 100 | | 124 | | 116 | | 144 | |
| 24.4337 | | 111 | | 150 | | 117 | | 112 | |
| 24.4542 | | 113 | | 115 | | 116 | | 125 | |
| 24.4746 | | 98 | | 132 | | 131 | | 128 | |
| 24.4951 | | 115 | | 139 | | 126 | | 117 | |
| 24.5155 | | 115 | | 129 | | 115 | | 117 | |
| 24.536 | | 109 | | 141 | | 107 | | 116 | |
| 24.5564 | | 102 | | 135 | | 135 | | 102 | |
| 24.5769 | | 108 | | 153 | | 126 | | 109 | |
| 24.5974 | | 123 | | 132 | | 105 | | 108 | |
| 24.6178 | | 140 | | 154 | | 109 | | 146 | |
| 24.6383 | | 134 | | 123 | | 119 | | 97 | |
| 24.6587 | | 118 | | 133 | | 120 | | 128 | |
| 24.6792 | | 151 | | 141 | | 132 | | 148 | |
| 24.6996 | | 94 | | 157 | | 138 | | 91 | |
| 24.7201 | | 119 | | 131 | | 136 | | 138 | |
| 24.7405 | | 119 | | 145 | | 128 | | 106 | |
| 24.761 | | 92 | | 111 | | 108 | | 117 | |
| 24.7814 | | 120 | | 128 | | 120 | | 120 | |
| 24.8019 | | 119 | | 126 | | 129 | | 111 | |
| 24.8223 | | 119 | | 145 | | 136 | | 135 | |
| 24.8428 | | 110 | | 140 | | 138 | | 129 | |
| 24.8632 | | 118 | | 138 | | 131 | | 105 | |
| 24.8837 | | 118 | | 152 | | 121 | | 121 | |
| 24.9041 | | 145 | | 134 | | 107 | | 128 | |
| 24.9246 | | 123 | | 147 | | 106 | | 122 | |
| 24.945 | | 116 | | 130 | | 137 | | 114 | |
| 24.9655 | | 140 | | 141 | | 152 | | 138 | |
| 24.9859 | | 115 | | 138 | | 123 | | 137 | |
| 25.0064 | | 126 | | 159 | | 130 | | 147 | |
| 25.0268 | | 129 | | 125 | | 135 | | 117 | |
| 25.0473 | | 124 | | 150 | | 128 | | 154 | |
| 25.0677 | | 124 | | 153 | | 144 | | 140 | |
| 25.0882 | | 127 | | 151 | | 145 | | 127 | |
| 25.1087 | | 122 | | 164 | | 146 | | 135 | |
| 25.1291 | | 123 | | 151 | | 166 | | 148 | |
| 25.1496 | | 122 | | 137 | | 139 | | 147 | |
| 25.17 | | 136 | | 150 | | 141 | | 158 | |
| 25.1905 | | 151 | | 147 | | 156 | | 134 | |
| 25.2109 | | 141 | | 154 | | 112 | | 150 | |
| 25.2314 | | 129 | | 171 | | 164 | | 137 | |
| 25.2518 | | 150 | | 148 | | 127 | | 144 | |
| 25.2723 | | 141 | | 160 | | 144 | | 157 | |
| 25.2927 | | 159 | | 175 | | 153 | | 172 | |
| 25.3132 | | 130 | | 171 | | 172 | | 155 | |
| 25.3336 | | 147 | | 160 | | 156 | | 165 | |
| 25.3541 | | 147 | | 180 | | 189 | | 155 | |
| 25.3745 | | 151 | | 143 | | 148 | | 154 | |
| 25.395 | | 158 | | 184 | | 170 | | 174 | |
| 25.4154 | | 158 | | 176 | | 176 | | 190 | |
| 25.4359 | | 183 | | 217 | | 193 | | 192 | |
| 25.4563 | | 224 | | 222 | | 240 | | 258 | |
| 25.4768 | | 274 | | 227 | | 275 | | 289 | |
| 25.4972 | | 363 | | 323 | | 345 | | 333 | |
| 25.5177 | | 266 | | 308 | | 292 | | 324 | |
| 25.5381 | | 175 | | 248 | | 227 | | 213 | |
| 25.5586 | | 170 | | 229 | | 172 | | 205 | |
| 25.579 | | 171 | | 206 | | 176 | | 177 | |
| 25.5995 | | 177 | | 192 | | 185 | | 184 | |
| 25.62 | | 179 | | 188 | | 171 | | 167 | |
| 25.6404 | | 162 | | 186 | | 193 | | 201 | |
| 25.6609 | | 161 | | 206 | | 161 | | 204 | |
| 25.6813 | | 184 | | 218 | | 193 | | 194 | |
| 25.7018 | | 177 | | 210 | | 165 | | 186 | |
| 25.7222 | | 191 | | 210 | | 150 | | 183 | |
| 25.7427 | | 163 | | 191 | | 167 | | 165 | |
| 25.7631 | | 149 | | 200 | | 155 | | 184 | |
| 25.7836 | | 184 | | 208 | | 183 | | 187 | |
| 25.804 | | 143 | | 178 | | 199 | | 167 | |
| 25.8245 | | 176 | | 208 | | 177 | | 192 | |
| 25.8449 | | 171 | | 207 | | 196 | | 197 | |
| 25.8654 | | 165 | | 203 | | 182 | | 212 | |
| 25.8858 | | 178 | | 210 | | 193 | | 193 | |
| 25.9063 | | 180 | | 203 | | 212 | | 241 | |
| 25.9267 | | 169 | | 207 | | 216 | | 210 | |
| 25.9472 | | 168 | | 207 | | 213 | | 186 | |
| 25.9676 | | 193 | | 210 | | 215 | | 189 | |
| 25.9881 | | 198 | | 244 | | 214 | | 214 | |
| 26.0085 | | 197 | | 222 | | 242 | | 241 | |
| 26.029 | | 193 | | 221 | | 232 | | 219 | |
| 26.0494 | | 179 | | 234 | | 233 | | 203 | |
| 26.0699 | | 218 | | 240 | | 216 | | 237 | |
| 26.0903 | | 198 | | 259 | | 216 | | 258 | |
| 26.1108 | | 197 | | 255 | | 210 | | 255 | |
| 26.1312 | | 225 | | 286 | | 209 | | 252 | |
| 26.1517 | | 221 | | 280 | | 239 | | 226 | |
| 26.1722 | | 215 | | 304 | | 257 | | 240 | |
| 26.1926 | | 239 | | 280 | | 255 | | 279 | |
| 26.2131 | | 232 | | 330 | | 282 | | 306 | |
| 26.2335 | | 295 | | 308 | | 261 | | 293 | |
| 26.254 | | 315 | | 355 | | 329 | | 301 | |
| 26.2744 | | 282 | | 347 | | 315 | | 349 | |
| 26.2949 | | 276 | | 397 | | 340 | | 369 | |
| 26.3153 | | 341 | | 401 | | 367 | | 379 | |
| 26.3358 | | 369 | | 450 | | 405 | | 425 | |
| 26.3562 | | 451 | | 478 | | 454 | | 453 | |
| 26.3767 | | 458 | | 579 | | 542 | | 557 | |
| 26.3971 | | 490 | | 620 | | 582 | | 661 | |
| 26.4176 | | 641 | | 754 | | 706 | | 696 | |
| 26.438 | | 725 | | 872 | | 715 | | 836 | |
| 26.4585 | | 860 | | 1067 | | 966 | | 1050 | |
| 26.4789 | | 1234 | | 1315 | | 1244 | | 1365 | |
| 26.4994 | | 1623 | | 1855 | | 1647 | | 1945 | |
| 26.5198 | | 2146 | | 2428 | | 2302 | | 2528 | |
| 26.5403 | | 3248 | | 3595 | | 3217 | | 3716 | |
| 26.5607 | | 5665 | | 5935 | | 5619 | | 6689 | |
| 26.5812 | | 12304 | | 11107 | | 11460 | | 13568 | |
| 26.6016 | | 27118 | | 22165 | | 23322 | | 27920 | |
| 26.6221 | | 46832 | | 40883 | | 42906 | | 47701 | |
| 26.6425 | | 35960 | | 44770 | | 42463 | | 42615 | |
| 26.663 | | 18238 | | 22116 | | 20920 | | 22874 | |
| 26.6835 | | 23472 | | 21135 | | 21845 | | 24460 | |
| 26.7039 | | 22565 | | 25273 | | 24701 | | 25365 | |
| 26.7244 | | 7741 | | 12383 | | 10959 | | 10825 | |
| 26.7448 | | 2731 | | 4052 | | 3592 | | 3909 | |
| 26.7653 | | 1656 | | 2280 | | 2022 | | 2224 | |
| 26.7857 | | 1160 | | 1619 | | 1453 | | 1584 | |
| 26.8062 | | 933 | | 1175 | | 1108 | | 1196 | |
| 26.8266 | | 745 | | 990 | | 898 | | 980 | |
| 26.8471 | | 613 | | 811 | | 781 | | 742 | |
| 26.8675 | | 518 | | 681 | | 641 | | 639 | |
| 26.888 | | 461 | | 520 | | 555 | | 571 | |
| 26.9084 | | 380 | | 514 | | 527 | | 480 | |
| 26.9289 | | 383 | | 408 | | 427 | | 451 | |
| 26.9493 | | 380 | | 447 | | 385 | | 392 | |
| 26.9698 | | 306 | | 386 | | 359 | | 403 | |
| 26.9902 | | 329 | | 368 | | 340 | | 347 | |
| 27.0107 | | 292 | | 357 | | 305 | | 360 | |
| 27.0311 | | 286 | | 344 | | 313 | | 326 | |
| 27.0516 | | 276 | | 332 | | 288 | | 315 | |
| 27.072 | | 239 | | 293 | | 290 | | 264 | |
| 27.0925 | | 203 | | 280 | | 264 | | 295 | |
| 27.1129 | | 198 | | 247 | | 255 | | 294 | |
| 27.1334 | | 220 | | 263 | | 243 | | 257 | |
| 27.1538 | | 220 | | 262 | | 234 | | 243 | |
| 27.1743 | | 202 | | 247 | | 229 | | 247 | |
| 27.1948 | | 203 | | 221 | | 228 | | 224 | |
| 27.2152 | | 183 | | 222 | | 227 | | 208 | |
| 27.2357 | | 174 | | 230 | | 206 | | 212 | |
| 27.2561 | | 174 | | 218 | | 217 | | 219 | |
| 27.2766 | | 188 | | 200 | | 194 | | 206 | |
| 27.297 | | 186 | | 222 | | 194 | | 173 | |
| 27.3175 | | 162 | | 200 | | 183 | | 196 | |
| 27.3379 | | 150 | | 202 | | 183 | | 188 | |
| 27.3584 | | 170 | | 183 | | 166 | | 201 | |
| 27.3788 | | 147 | | 190 | | 181 | | 168 | |
| 27.3993 | | 152 | | 187 | | 182 | | 174 | |
| 27.4197 | | 127 | | 188 | | 154 | | 163 | |
| 27.4402 | | 132 | | 180 | | 177 | | 163 | |
| 27.4606 | | 165 | | 198 | | 187 | | 156 | |
| 27.4811 | | 157 | | 158 | | 176 | | 168 | |
| 27.5015 | | 174 | | 161 | | 168 | | 159 | |
| 27.522 | | 134 | | 167 | | 171 | | 156 | |
| 27.5424 | | 142 | | 155 | | 152 | | 159 | |
| 27.5629 | | 125 | | 163 | | 144 | | 159 | |
| 27.5833 | | 142 | | 185 | | 144 | | 176 | |
| 27.6038 | | 122 | | 148 | | 152 | | 150 | |
| 27.6242 | | 114 | | 166 | | 141 | | 159 | |
| 27.6447 | | 131 | | 139 | | 135 | | 162 | |
| 27.6651 | | 121 | | 133 | | 153 | | 153 | |
| 27.6856 | | 125 | | 145 | | 145 | | 137 | |
| 27.706 | | 116 | | 160 | | 141 | | 144 | |
| 27.7265 | | 139 | | 172 | | 161 | | 122 | |
| 27.747 | | 127 | | 150 | | 108 | | 144 | |
| 27.7674 | | 134 | | 131 | | 145 | | 115 | |
| 27.7879 | | 120 | | 141 | | 148 | | 148 | |
| 27.8083 | | 113 | | 132 | | 107 | | 150 | |
| 27.8288 | | 136 | | 129 | | 136 | | 122 | |
| 27.8492 | | 115 | | 149 | | 139 | | 124 | |
| 27.8697 | | 122 | | 148 | | 127 | | 124 | |
| 27.8901 | | 99 | | 141 | | 124 | | 122 | |
| 27.9106 | | 108 | | 126 | | 129 | | 123 | |
| 27.931 | | 105 | | 131 | | 133 | | 117 | |
| 27.9515 | | 121 | | 132 | | 141 | | 119 | |
| 27.9719 | | 110 | | 127 | | 139 | | 108 | |
| 27.9924 | | 105 | | 123 | | 145 | | 132 | |
| 28.0128 | | 114 | | 121 | | 120 | | 103 | |
| 28.0333 | | 107 | | 118 | | 119 | | 107 | |
| 28.0537 | | 108 | | 129 | | 110 | | 120 | |
| 28.0742 | | 106 | | 141 | | 110 | | 129 | |
| 28.0946 | | 105 | | 124 | | 120 | | 120 | |
| 28.1151 | | 107 | | 117 | | 96 | | 107 | |
| 28.1355 | | 93 | | 120 | | 117 | | 94 | |
| 28.156 | | 94 | | 119 | | 118 | | 113 | |
| 28.1764 | | 110 | | 98 | | 95 | | 128 | |
| 28.1969 | | 89 | | 105 | | 124 | | 95 | |
| 28.2173 | | 89 | | 121 | | 102 | | 99 | |
| 28.2378 | | 112 | | 109 | | 108 | | 79 | |
| 28.2583 | | 85 | | 117 | | 105 | | 101 | |
| 28.2787 | | 110 | | 110 | | 89 | | 104 | |
| 28.2992 | | 103 | | 107 | | 97 | | 102 | |
| 28.3196 | | 94 | | 107 | | 102 | | 121 | |
| 28.3401 | | 94 | | 107 | | 93 | | 107 | |
| 28.3605 | | 90 | | 86 | | 101 | | 121 | |
| 28.381 | | 95 | | 111 | | 84 | | 102 | |
| 28.4014 | | 105 | | 97 | | 88 | | 93 | |
| 28.4219 | | 97 | | 116 | | 101 | | 86 | |
| 28.4423 | | 86 | | 89 | | 95 | | 75 | |
| 28.4628 | | 95 | | 94 | | 91 | | 114 | |
| 28.4832 | | 86 | | 107 | | 108 | | 96 | |
| 28.5037 | | 80 | | 103 | | 96 | | 87 | |
| 28.5241 | | 104 | | 112 | | 94 | | 80 | |
| 28.5446 | | 100 | | 95 | | 119 | | 96 | |
| 28.565 | | 88 | | 97 | | 80 | | 81 | |
| 28.5855 | | 97 | | 116 | | 104 | | 92 | |
| 28.6059 | | 65 | | 87 | | 89 | | 91 | |
| 28.6264 | | 81 | | 97 | | 90 | | 85 | |
| 28.6468 | | 88 | | 89 | | 80 | | 64 | |
| 28.6673 | | 81 | | 96 | | 68 | | 90 | |
| 28.6877 | | 72 | | 91 | | 72 | | 96 | |
| 28.7082 | | 76 | | 100 | | 83 | | 78 | |
| 28.7286 | | 72 | | 105 | | 91 | | 81 | |
| 28.7491 | | 73 | | 96 | | 75 | | 95 | |
| 28.7696 | | 55 | | 89 | | 91 | | 75 | |
| 28.79 | | 78 | | 93 | | 84 | | 79 | |
| 28.8105 | | 68 | | 105 | | 80 | | 94 | |
| 28.8309 | | 81 | | 106 | | 95 | | 61 | |
| 28.8514 | | 72 | | 105 | | 95 | | 61 | |
| 28.8718 | | 79 | | 75 | | 85 | | 83 | |
| 28.8923 | | 77 | | 85 | | 58 | | 71 | |
| 28.9127 | | 60 | | 85 | | 101 | | 65 | |
| 28.9332 | | 52 | | 74 | | 84 | | 79 | |
| 28.9536 | | 73 | | 85 | | 85 | | 83 | |
| 28.9741 | | 90 | | 94 | | 83 | | 82 | |
| 28.9945 | | 72 | | 94 | | 96 | | 103 | |
| 29.015 | | 55 | | 85 | | 81 | | 75 | |
| 29.0354 | | 72 | | 77 | | 75 | | 90 | |
| 29.0559 | | 85 | | 98 | | 91 | | 70 | |
| 29.0763 | | 73 | | 86 | | 88 | | 75 | |
| 29.0968 | | 56 | | 83 | | 80 | | 87 | |
| 29.1172 | | 63 | | 60 | | 78 | | 77 | |
| 29.1377 | | 67 | | 73 | | 64 | | 68 | |
| 29.1581 | | 78 | | 89 | | 58 | | 70 | |
| 29.1786 | | 73 | | 81 | | 63 | | 62 | |
| 29.199 | | 53 | | 84 | | 91 | | 65 | |
| 29.2195 | | 55 | | 72 | | 78 | | 81 | |
| 29.2399 | | 67 | | 74 | | 69 | | 57 | |
| 29.2604 | | 55 | | 91 | | 64 | | 62 | |
| 29.2808 | | 38 | | 67 | | 66 | | 73 | |
| 29.3013 | | 61 | | 69 | | 85 | | 72 | |
| 29.3218 | | 56 | | 62 | | 59 | | 82 | |
| 29.3422 | | 67 | | 69 | | 77 | | 61 | |
| 29.3627 | | 60 | | 77 | | 65 | | 64 | |
| 29.3831 | | 54 | | 87 | | 68 | | 77 | |
| 29.4036 | | 45 | | 82 | | 59 | | 75 | |
| 29.424 | | 58 | | 87 | | 57 | | 64 | |
| 29.4445 | | 58 | | 78 | | 69 | | 69 | |
| 29.4649 | | 57 | | 68 | | 63 | | 58 | |
| 29.4854 | | 58 | | 94 | | 52 | | 62 | |
| 29.5058 | | 78 | | 80 | | 55 | | 66 | |
| 29.5263 | | 60 | | 86 | | 57 | | 76 | |
| 29.5467 | | 76 | | 68 | | 70 | | 63 | |
| 29.5672 | | 54 | | 63 | | 65 | | 58 | |
| 29.5876 | | 63 | | 50 | | 67 | | 61 | |
| 29.6081 | | 51 | | 57 | | 63 | | 53 | |
| 29.6285 | | 50 | | 66 | | 78 | | 58 | |
| 29.649 | | 62 | | 66 | | 69 | | 59 | |
| 29.6694 | | 53 | | 72 | | 73 | | 69 | |
| 29.6899 | | 54 | | 63 | | 58 | | 59 | |
| 29.7103 | | 62 | | 79 | | 64 | | 68 | |
| 29.7308 | | 67 | | 60 | | 69 | | 59 | |
| 29.7512 | | 60 | | 68 | | 60 | | 62 | |
| 29.7717 | | 45 | | 76 | | 72 | | 53 | |
| 29.7921 | | 51 | | 67 | | 70 | | 70 | |
| 29.8126 | | 57 | | 63 | | 61 | | 53 | |
| 29.8331 | | 53 | | 62 | | 49 | | 65 | |
| 29.8535 | | 56 | | 59 | | 58 | | 57 | |
| 29.874 | | 56 | | 67 | | 70 | | 65 | |
| 29.8944 | | 45 | | 58 | | 68 | | 59 | |
| 29.9149 | | 45 | | 61 | | 71 | | 74 | |
| 29.9353 | | 59 | | 67 | | 53 | | 49 | |
| 29.9558 | | 43 | | 69 | | 61 | | 45 | |
| 29.9762 | | 51 | | 65 | | 58 | | 51 | |
| 29.9967 | | 61 | | 64 | | 59 | | 63 | |
| 30.0171 | | 53 | | 62 | | 54 | | 47 | |
| 30.0376 | | 62 | | 68 | | 51 | | 52 | |
| 30.058 | | 63 | | 60 | | 62 | | 51 | |
| 30.0785 | | 57 | | 66 | | 45 | | 74 | |
| 30.0989 | | 56 | | 56 | | 53 | | 41 | |
| 30.1194 | | 49 | | 72 | | 59 | | 50 | |
| 30.1398 | | 51 | | 62 | | 49 | | 68 | |
| 30.1603 | | 39 | | 61 | | 42 | | 55 | |
| 30.1807 | | 52 | | 55 | | 64 | | 51 | |
| 30.2012 | | 61 | | 59 | | 45 | | 46 | |
| 30.2216 | | 31 | | 54 | | 47 | | 51 | |
| 30.2421 | | 48 | | 60 | | 51 | | 68 | |
| 30.2625 | | 39 | | 56 | | 51 | | 42 | |
| 30.283 | | 57 | | 70 | | 48 | | 61 | |
| 30.3034 | | 56 | | 62 | | 47 | | 47 | |
| 30.3239 | | 45 | | 50 | | 53 | | 57 | |
| 30.3443 | | 52 | | 64 | | 57 | | 53 | |
| 30.3648 | | 46 | | 54 | | 49 | | 62 | |
| 30.3853 | | 55 | | 61 | | 59 | | 54 | |
| 30.4057 | | 55 | | 69 | | 54 | | 47 | |
| 30.4262 | | 56 | | 70 | | 73 | | 54 | |
| 30.4466 | | 38 | | 56 | | 44 | | 47 | |
| 30.4671 | | 54 | | 73 | | 53 | | 61 | |
| 30.4875 | | 60 | | 57 | | 57 | | 60 | |
| 30.508 | | 41 | | 53 | | 55 | | 60 | |
| 30.5284 | | 47 | | 62 | | 53 | | 63 | |
| 30.5489 | | 51 | | 62 | | 65 | | 55 | |
| 30.5693 | | 43 | | 71 | | 68 | | 44 | |
| 30.5898 | | 51 | | 50 | | 53 | | 46 | |
| 30.6102 | | 54 | | 48 | | 52 | | 41 | |
| 30.6307 | | 68 | | 61 | | 63 | | 54 | |
| 30.6511 | | 57 | | 62 | | 56 | | 56 | |
| 30.6716 | | 50 | | 66 | | 65 | | 37 | |
| 30.692 | | 44 | | 77 | | 58 | | 46 | |
| 30.7125 | | 54 | | 57 | | 61 | | 39 | |
| 30.7329 | | 55 | | 57 | | 48 | | 52 | |
| 30.7534 | | 54 | | 49 | | 51 | | 48 | |
| 30.7738 | | 47 | | 59 | | 40 | | 59 | |
| 30.7943 | | 62 | | 53 | | 50 | | 51 | |
| 30.8147 | | 54 | | 57 | | 62 | | 51 | |
| 30.8352 | | 51 | | 53 | | 54 | | 58 | |
| 30.8556 | | 46 | | 65 | | 54 | | 57 | |
| 30.8761 | | 46 | | 68 | | 52 | | 51 | |
| 30.8966 | | 56 | | 67 | | 47 | | 57 | |
| 30.917 | | 53 | | 64 | | 50 | | 52 | |
| 30.9375 | | 51 | | 59 | | 66 | | 49 | |
| 30.9579 | | 47 | | 60 | | 57 | | 47 | |
| 30.9784 | | 54 | | 67 | | 53 | | 57 | |
| 30.9988 | | 42 | | 60 | | 44 | | 45 | |
| 31.0193 | | 50 | | 56 | | 49 | | 50 | |
| 31.0397 | | 57 | | 54 | | 46 | | 55 | |
| 31.0602 | | 63 | | 63 | | 56 | | 61 | |
| 31.0806 | | 40 | | 53 | | 56 | | 45 | |
| 31.1011 | | 56 | | 55 | | 53 | | 44 | |
| 31.1215 | | 55 | | 49 | | 48 | | 51 | |
| 31.142 | | 48 | | 61 | | 65 | | 50 | |
| 31.1624 | | 43 | | 62 | | 36 | | 48 | |
| 31.1829 | | 45 | | 65 | | 46 | | 64 | |
| 31.2033 | | 46 | | 58 | | 64 | | 53 | |
| 31.2238 | | 61 | | 58 | | 55 | | 47 | |
| 31.2442 | | 61 | | 49 | | 58 | | 58 | |
| 31.2647 | | 40 | | 48 | | 44 | | 40 | |
| 31.2851 | | 46 | | 49 | | 66 | | 54 | |
| 31.3056 | | 50 | | 57 | | 46 | | 52 | |
| 31.326 | | 43 | | 56 | | 53 | | 53 | |
| 31.3465 | | 42 | | 49 | | 61 | | 47 | |
| 31.3669 | | 38 | | 53 | | 43 | | 48 | |
| 31.3874 | | 45 | | 49 | | 50 | | 53 | |
| 31.4079 | | 53 | | 57 | | 54 | | 54 | |
| 31.4283 | | 57 | | 48 | | 47 | | 42 | |
| 31.4488 | | 56 | | 59 | | 40 | | 50 | |
| 31.4692 | | 67 | | 55 | | 64 | | 58 | |
| 31.4897 | | 68 | | 56 | | 50 | | 42 | |
| 31.5101 | | 65 | | 60 | | 60 | | 64 | |
| 31.5306 | | 49 | | 67 | | 55 | | 52 | |
| 31.551 | | 45 | | 57 | | 52 | | 43 | |
| 31.5715 | | 55 | | 54 | | 44 | | 42 | |
| 31.5919 | | 45 | | 60 | | 48 | | 56 | |
| 31.6124 | | 41 | | 59 | | 55 | | 55 | |
| 31.6328 | | 40 | | 60 | | 54 | | 41 | |
| 31.6533 | | 51 | | 57 | | 66 | | 60 | |
| 31.6737 | | 53 | | 60 | | 63 | | 59 | |
| 31.6942 | | 52 | | 58 | | 49 | | 41 | |
| 31.7146 | | 46 | | 54 | | 60 | | 55 | |
| 31.7351 | | 60 | | 59 | | 50 | | 54 | |
| 31.7555 | | 50 | | 61 | | 59 | | 53 | |
| 31.776 | | 49 | | 67 | | 54 | | 43 | |
| 31.7964 | | 39 | | 73 | | 54 | | 52 | |
| 31.8169 | | 54 | | 53 | | 52 | | 54 | |
| 31.8373 | | 50 | | 54 | | 37 | | 59 | |
| 31.8578 | | 60 | | 70 | | 54 | | 44 | |
| 31.8782 | | 38 | | 58 | | 56 | | 49 | |
| 31.8987 | | 53 | | 44 | | 49 | | 48 | |
| 31.9191 | | 51 | | 57 | | 55 | | 44 | |
| 31.9396 | | 54 | | 59 | | 54 | | 45 | |
| 31.9601 | | 47 | | 69 | | 49 | | 56 | |
| 31.9805 | | 50 | | 65 | | 49 | | 62 | |
| 32.001 | | 45 | | 73 | | 53 | | 46 | |
| 32.0214 | | 50 | | 51 | | 48 | | 60 | |
| 32.0419 | | 38 | | 55 | | 59 | | 46 | |
| 32.0623 | | 45 | | 64 | | 43 | | 48 | |
| 32.0828 | | 35 | | 60 | | 41 | | 55 | |
| 32.1032 | | 54 | | 50 | | 52 | | 47 | |
| 32.1237 | | 52 | | 49 | | 52 | | 60 | |
| 32.1441 | | 57 | | 51 | | 65 | | 57 | |
| 32.1646 | | 53 | | 50 | | 54 | | 47 | |
| 32.185 | | 46 | | 68 | | 55 | | 42 | |
| 32.2055 | | 43 | | 53 | | 54 | | 42 | |
| 32.2259 | | 43 | | 54 | | 46 | | 52 | |
| 32.2464 | | 50 | | 51 | | 66 | | 46 | |
| 32.2668 | | 50 | | 49 | | 40 | | 47 | |
| 32.2873 | | 41 | | 45 | | 52 | | 49 | |
| 32.3077 | | 31 | | 60 | | 44 | | 45 | |
| 32.3282 | | 48 | | 58 | | 51 | | 39 | |
| 32.3486 | | 46 | | 45 | | 59 | | 45 | |
| 32.3691 | | 42 | | 48 | | 52 | | 61 | |
| 32.3895 | | 51 | | 49 | | 46 | | 60 | |
| 32.41 | | 56 | | 43 | | 53 | | 43 | |
| 32.4304 | | 49 | | 62 | | 51 | | 34 | |
| 32.4509 | | 54 | | 52 | | 59 | | 58 | |
| 32.4714 | | 45 | | 66 | | 52 | | 37 | |
| 32.4918 | | 58 | | 57 | | 46 | | 55 | |
| 32.5123 | | 46 | | 56 | | 59 | | 42 | |
| 32.5327 | | 45 | | 65 | | 48 | | 48 | |
| 32.5532 | | 41 | | 54 | | 50 | | 50 | |
| 32.5736 | | 47 | | 66 | | 47 | | 44 | |
| 32.5941 | | 40 | | 61 | | 46 | | 46 | |
| 32.6145 | | 39 | | 60 | | 48 | | 56 | |
| 32.635 | | 52 | | 58 | | 35 | | 52 | |
| 32.6554 | | 45 | | 50 | | 53 | | 60 | |
| 32.6759 | | 59 | | 48 | | 46 | | 68 | |
| 32.6963 | | 58 | | 78 | | 64 | | 34 | |
| 32.7168 | | 42 | | 63 | | 58 | | 53 | |
| 32.7372 | | 53 | | 53 | | 57 | | 50 | |
| 32.7577 | | 51 | | 67 | | 50 | | 55 | |
| 32.7781 | | 54 | | 57 | | 56 | | 44 | |
| 32.7986 | | 44 | | 61 | | 69 | | 66 | |
| 32.819 | | 56 | | 49 | | 49 | | 56 | |
| 32.8395 | | 71 | | 84 | | 61 | | 59 | |
| 32.8599 | | 69 | | 81 | | 80 | | 81 | |
| 32.8804 | | 94 | | 89 | | 104 | | 111 | |
| 32.9008 | | 129 | | 125 | | 116 | | 132 | |
| 32.9213 | | 114 | | 130 | | 128 | | 105 | |
| 32.9417 | | 64 | | 93 | | 77 | | 91 | |
| 32.9622 | | 61 | | 78 | | 65 | | 64 | |
| 32.9827 | | 65 | | 74 | | 59 | | 67 | |
| 33.0031 | | 55 | | 71 | | 49 | | 66 | |
| 33.0236 | | 51 | | 55 | | 58 | | 48 | |
| 33.044 | | 43 | | 52 | | 54 | | 55 | |
| 33.0645 | | 47 | | 66 | | 61 | | 58 | |
| 33.0849 | | 60 | | 52 | | 53 | | 52 | |
| 33.1054 | | 54 | | 46 | | 46 | | 46 | |
| 33.1258 | | 45 | | 55 | | 55 | | 52 | |
| 33.1463 | | 53 | | 71 | | 48 | | 56 | |
| 33.1667 | | 42 | | 56 | | 48 | | 44 | |
| 33.1872 | | 48 | | 57 | | 40 | | 54 | |
| 33.2076 | | 56 | | 67 | | 56 | | 37 | |
| 33.2281 | | 59 | | 67 | | 46 | | 47 | |
| 33.2485 | | 54 | | 48 | | 53 | | 68 | |
| 33.269 | | 44 | | 41 | | 49 | | 41 | |
| 33.2894 | | 38 | | 57 | | 45 | | 40 | |
| 33.3099 | | 48 | | 40 | | 41 | | 44 | |
| 33.3303 | | 45 | | 41 | | 49 | | 54 | |
| 33.3508 | | 41 | | 44 | | 46 | | 53 | |
| 33.3712 | | 46 | | 53 | | 49 | | 42 | |
| 33.3917 | | 68 | | 63 | | 64 | | 51 | |
| 33.4121 | | 47 | | 51 | | 47 | | 50 | |
| 33.4326 | | 51 | | 60 | | 50 | | 54 | |
| 33.453 | | 55 | | 44 | | 64 | | 52 | |
| 33.4735 | | 57 | | 61 | | 47 | | 51 | |
| 33.4939 | | 39 | | 47 | | 55 | | 42 | |
| 33.5144 | | 50 | | 64 | | 52 | | 47 | |
| 33.5349 | | 53 | | 68 | | 43 | | 61 | |
| 33.5553 | | 53 | | 62 | | 48 | | 41 | |
| 33.5758 | | 46 | | 65 | | 49 | | 50 | |
| 33.5962 | | 49 | | 51 | | 55 | | 59 | |
| 33.6167 | | 54 | | 59 | | 54 | | 51 | |
| 33.6371 | | 53 | | 57 | | 50 | | 46 | |
| 33.6576 | | 34 | | 68 | | 44 | | 49 | |
| 33.678 | | 58 | | 63 | | 42 | | 57 | |
| 33.6985 | | 52 | | 66 | | 43 | | 49 | |
| 33.7189 | | 43 | | 56 | | 51 | | 56 | |
| 33.7394 | | 41 | | 48 | | 52 | | 47 | |
| 33.7598 | | 51 | | 54 | | 43 | | 54 | |
| 33.7803 | | 51 | | 60 | | 50 | | 56 | |
| 33.8007 | | 37 | | 52 | | 56 | | 46 | |
| 33.8212 | | 42 | | 47 | | 48 | | 58 | |
| 33.8416 | | 50 | | 46 | | 54 | | 49 | |
| 33.8621 | | 63 | | 57 | | 56 | | 33 | |
| 33.8825 | | 43 | | 53 | | 51 | | 51 | |
| 33.903 | | 46 | | 50 | | 51 | | 57 | |
| 33.9234 | | 36 | | 53 | | 47 | | 59 | |
| 33.9439 | | 42 | | 62 | | 57 | | 51 | |
| 33.9643 | | 44 | | 48 | | 58 | | 49 | |
| 33.9848 | | 49 | | 63 | | 49 | | 57 | |
| 34.0052 | | 49 | | 62 | | 42 | | 51 | |
| 34.0257 | | 45 | | 59 | | 52 | | 59 | |
| 34.0462 | | 40 | | 48 | | 60 | | 50 | |
| 34.0666 | | 49 | | 56 | | 57 | | 57 | |
| 34.0871 | | 55 | | 57 | | 48 | | 48 | |
| 34.1075 | | 39 | | 41 | | 56 | | 51 | |
| 34.128 | | 49 | | 66 | | 47 | | 49 | |
| 34.1484 | | 45 | | 59 | | 59 | | 39 | |
| 34.1689 | | 54 | | 59 | | 51 | | 42 | |
| 34.1893 | | 56 | | 52 | | 52 | | 48 | |
| 34.2098 | | 49 | | 67 | | 42 | | 58 | |
| 34.2302 | | 44 | | 54 | | 55 | | 45 | |
| 34.2507 | | 43 | | 54 | | 51 | | 56 | |
| 34.2711 | | 43 | | 42 | | 45 | | 41 | |
| 34.2916 | | 46 | | 58 | | 50 | | 55 | |
| 34.312 | | 47 | | 49 | | 58 | | 48 | |
| 34.3325 | | 58 | | 50 | | 50 | | 51 | |
| 34.3529 | | 34 | | 55 | | 50 | | 55 | |
| 34.3734 | | 50 | | 54 | | 55 | | 55 | |
| 34.3938 | | 49 | | 53 | | 60 | | 48 | |
| 34.4143 | | 54 | | 49 | | 35 | | 39 | |
| 34.4347 | | 46 | | 39 | | 67 | | 59 | |
| 34.4552 | | 44 | | 45 | | 60 | | 49 | |
| 34.4756 | | 41 | | 67 | | 48 | | 51 | |
| 34.4961 | | 48 | | 68 | | 58 | | 47 | |
| 34.5165 | | 45 | | 39 | | 53 | | 61 | |
| 34.537 | | 40 | | 60 | | 50 | | 51 | |
| 34.5575 | | 46 | | 52 | | 57 | | 53 | |
| 34.5779 | | 64 | | 56 | | 51 | | 40 | |
| 34.5984 | | 49 | | 59 | | 59 | | 57 | |
| 34.6188 | | 45 | | 54 | | 50 | | 45 | |
| 34.6393 | | 52 | | 47 | | 50 | | 41 | |
| 34.6597 | | 49 | | 59 | | 60 | | 50 | |
| 34.6802 | | 47 | | 45 | | 53 | | 55 | |
| 34.7006 | | 45 | | 54 | | 49 | | 54 | |
| 34.7211 | | 51 | | 52 | | 58 | | 47 | |
| 34.7415 | | 43 | | 61 | | 46 | | 57 | |
| 34.762 | | 42 | | 49 | | 56 | | 32 | |
| 34.7824 | | 53 | | 43 | | 53 | | 51 | |
| 34.8029 | | 47 | | 54 | | 54 | | 59 | |
| 34.8233 | | 39 | | 50 | | 40 | | 42 | |
| 34.8438 | | 44 | | 65 | | 50 | | 44 | |
| 34.8642 | | 47 | | 54 | | 56 | | 53 | |
| 34.8847 | | 58 | | 50 | | 55 | | 55 | |
| 34.9051 | | 40 | | 61 | | 46 | | 49 | |
| 34.9256 | | 69 | | 70 | | 62 | | 56 | |
| 34.946 | | 64 | | 64 | | 66 | | 52 | |
| 34.9665 | | 63 | | 70 | | 51 | | 58 | |
| 34.9869 | | 57 | | 58 | | 65 | | 51 | |
| 35.0074 | | 46 | | 66 | | 61 | | 52 | |
| 35.0278 | | 61 | | 53 | | 45 | | 52 | |
| 35.0483 | | 42 | | 62 | | 58 | | 54 | |
| 35.0687 | | 43 | | 59 | | 70 | | 58 | |
| 35.0892 | | 50 | | 58 | | 51 | | 44 | |
| 35.1097 | | 44 | | 68 | | 51 | | 60 | |
| 35.1301 | | 47 | | 58 | | 55 | | 53 | |
| 35.1506 | | 52 | | 50 | | 69 | | 51 | |
| 35.171 | | 60 | | 58 | | 52 | | 50 | |
| 35.1915 | | 40 | | 41 | | 57 | | 39 | |
| 35.2119 | | 48 | | 49 | | 57 | | 64 | |
| 35.2324 | | 69 | | 60 | | 42 | | 63 | |
| 35.2528 | | 49 | | 66 | | 50 | | 62 | |
| 35.2733 | | 44 | | 44 | | 44 | | 60 | |
| 35.2937 | | 46 | | 56 | | 58 | | 48 | |
| 35.3142 | | 58 | | 65 | | 61 | | 58 | |
| 35.3346 | | 40 | | 61 | | 47 | | 61 | |
| 35.3551 | | 38 | | 53 | | 42 | | 58 | |
| 35.3755 | | 50 | | 63 | | 49 | | 50 | |
| 35.396 | | 57 | | 51 | | 51 | | 52 | |
| 35.4164 | | 55 | | 59 | | 56 | | 54 | |
| 35.4369 | | 53 | | 65 | | 49 | | 70 | |
| 35.4573 | | 56 | | 71 | | 61 | | 75 | |
| 35.4778 | | 79 | | 97 | | 85 | | 88 | |
| 35.4982 | | 119 | | 115 | | 100 | | 112 | |
| 35.5187 | | 115 | | 118 | | 135 | | 123 | |
| 35.5391 | | 96 | | 112 | | 125 | | 103 | |
| 35.5596 | | 74 | | 86 | | 73 | | 63 | |
| 35.58 | | 44 | | 69 | | 54 | | 64 | |
| 35.6005 | | 51 | | 72 | | 73 | | 73 | |
| 35.621 | | 58 | | 68 | | 58 | | 57 | |
| 35.6414 | | 61 | | 68 | | 64 | | 52 | |
| 35.6619 | | 54 | | 66 | | 53 | | 50 | |
| 35.6823 | | 50 | | 52 | | 58 | | 64 | |
| 35.7028 | | 45 | | 62 | | 58 | | 61 | |
| 35.7232 | | 64 | | 67 | | 61 | | 65 | |
| 35.7437 | | 57 | | 82 | | 52 | | 56 | |
| 35.7641 | | 49 | | 60 | | 52 | | 66 | |
| 35.7846 | | 47 | | 65 | | 63 | | 52 | |
| 35.805 | | 65 | | 67 | | 64 | | 63 | |
| 35.8255 | | 50 | | 63 | | 70 | | 51 | |
| 35.8459 | | 54 | | 64 | | 61 | | 62 | |
| 35.8664 | | 61 | | 62 | | 60 | | 71 | |
| 35.8868 | | 46 | | 67 | | 56 | | 59 | |
| 35.9073 | | 62 | | 59 | | 59 | | 58 | |
| 35.9277 | | 53 | | 76 | | 70 | | 67 | |
| 35.9482 | | 57 | | 83 | | 56 | | 66 | |
| 35.9686 | | 61 | | 76 | | 48 | | 55 | |
| 35.9891 | | 70 | | 73 | | 68 | | 66 | |
| 36.0095 | | 58 | | 70 | | 58 | | 60 | |
| 36.03 | | 59 | | 67 | | 65 | | 64 | |
| 36.0504 | | 57 | | 64 | | 72 | | 51 | |
| 36.0709 | | 58 | | 49 | | 65 | | 62 | |
| 36.0913 | | 61 | | 61 | | 61 | | 37 | |
| 36.1118 | | 55 | | 79 | | 73 | | 67 | |
| 36.1323 | | 66 | | 77 | | 67 | | 64 | |
| 36.1527 | | 60 | | 80 | | 58 | | 65 | |
| 36.1732 | | 74 | | 69 | | 74 | | 79 | |
| 36.1936 | | 81 | | 95 | | 87 | | 106 | |
| 36.2141 | | 84 | | 98 | | 86 | | 104 | |
| 36.2345 | | 107 | | 116 | | 108 | | 125 | |
| 36.255 | | 129 | | 126 | | 138 | | 123 | |
| 36.2754 | | 121 | | 124 | | 93 | | 128 | |
| 36.2959 | | 108 | | 132 | | 114 | | 107 | |
| 36.3163 | | 116 | | 130 | | 116 | | 116 | |
| 36.3368 | | 90 | | 133 | | 119 | | 140 | |
| 36.3572 | | 166 | | 158 | | 148 | | 143 | |
| 36.3777 | | 191 | | 177 | | 157 | | 167 | |
| 36.3981 | | 188 | | 192 | | 185 | | 186 | |
| 36.4186 | | 215 | | 228 | | 229 | | 232 | |
| 36.439 | | 299 | | 292 | | 273 | | 356 | |
| 36.4595 | | 481 | | 448 | | 423 | | 529 | |
| 36.4799 | | 923 | | 739 | | 733 | | 940 | |
| 36.5004 | | 1825 | | 1414 | | 1506 | | 1713 | |
| 36.5208 | | 2972 | | 2553 | | 2551 | | 2798 | |
| 36.5413 | | 3228 | | 3134 | | 2905 | | 2889 | |
| 36.5617 | | 1577 | | 1829 | | 1602 | | 1573 | |
| 36.5822 | | 1067 | | 1014 | | 974 | | 1181 | |
| 36.6026 | | 1386 | | 1211 | | 1164 | | 1323 | |
| 36.6231 | | 1809 | | 1624 | | 1659 | | 1678 | |
| 36.6435 | | 1382 | | 1430 | | 1267 | | 1271 | |
| 36.664 | | 560 | | 664 | | 604 | | 583 | |
| 36.6845 | | 292 | | 330 | | 269 | | 329 | |
| 36.7049 | | 207 | | 229 | | 213 | | 247 | |
| 36.7254 | | 151 | | 184 | | 167 | | 175 | |
| 36.7458 | | 146 | | 154 | | 138 | | 166 | |
| 36.7663 | | 103 | | 130 | | 131 | | 138 | |
| 36.7867 | | 115 | | 94 | | 128 | | 130 | |
| 36.8072 | | 80 | | 91 | | 100 | | 97 | |
| 36.8276 | | 91 | | 82 | | 84 | | 86 | |
| 36.8481 | | 77 | | 96 | | 88 | | 86 | |
| 36.8685 | | 83 | | 87 | | 77 | | 77 | |
| 36.889 | | 69 | | 65 | | 69 | | 77 | |
| 36.9094 | | 61 | | 69 | | 89 | | 69 | |
| 36.9299 | | 73 | | 77 | | 80 | | 72 | |
| 36.9503 | | 57 | | 73 | | 82 | | 70 | |
| 36.9708 | | 67 | | 63 | | 76 | | 66 | |
| 36.9912 | | 67 | | 60 | | 64 | | 66 | |
| 37.0117 | | 56 | | 57 | | 67 | | 82 | |
| 37.0321 | | 56 | | 82 | | 65 | | 66 | |
| 37.0526 | | 70 | | 69 | | 61 | | 67 | |
| 37.073 | | 59 | | 63 | | 62 | | 72 | |
| 37.0935 | | 60 | | 70 | | 56 | | 68 | |
| 37.1139 | | 55 | | 54 | | 63 | | 67 | |
| 37.1344 | | 64 | | 46 | | 71 | | 55 | |
| 37.1548 | | 59 | | 87 | | 63 | | 51 | |
| 37.1753 | | 59 | | 58 | | 55 | | 62 | |
| 37.1958 | | 42 | | 48 | | 53 | | 69 | |
| 37.2162 | | 58 | | 66 | | 60 | | 52 | |
| 37.2367 | | 72 | | 64 | | 63 | | 54 | |
| 37.2571 | | 60 | | 57 | | 66 | | 69 | |
| 37.2776 | | 69 | | 60 | | 64 | | 63 | |
| 37.298 | | 62 | | 68 | | 47 | | 62 | |
| 37.3185 | | 61 | | 60 | | 68 | | 62 | |
| 37.3389 | | 45 | | 62 | | 54 | | 51 | |
| 37.3594 | | 50 | | 66 | | 74 | | 65 | |
| 37.3798 | | 49 | | 58 | | 61 | | 58 | |
| 37.4003 | | 59 | | 56 | | 51 | | 56 | |
| 37.4207 | | 70 | | 61 | | 50 | | 63 | |
| 37.4412 | | 60 | | 58 | | 57 | | 49 | |
| 37.4616 | | 58 | | 60 | | 54 | | 65 | |
| 37.4821 | | 59 | | 64 | | 44 | | 54 | |
| 37.5025 | | 53 | | 50 | | 47 | | 69 | |
| 37.523 | | 60 | | 65 | | 54 | | 55 | |
| 37.5434 | | 48 | | 49 | | 49 | | 52 | |
| 37.5639 | | 75 | | 57 | | 55 | | 43 | |
| 37.5843 | | 56 | | 68 | | 53 | | 63 | |
| 37.6048 | | 58 | | 59 | | 66 | | 67 | |
| 37.6252 | | 60 | | 56 | | 69 | | 56 | |
| 37.6457 | | 63 | | 74 | | 61 | | 53 | |
| 37.6661 | | 62 | | 59 | | 55 | | 46 | |
| 37.6866 | | 75 | | 64 | | 76 | | 60 | |
| 37.707 | | 66 | | 62 | | 54 | | 54 | |
| 37.7275 | | 62 | | 63 | | 85 | | 54 | |
| 37.748 | | 65 | | 50 | | 59 | | 74 | |
| 37.7684 | | 52 | | 59 | | 66 | | 47 | |
| 37.7889 | | 52 | | 52 | | 63 | | 62 | |
| 37.8093 | | 63 | | 49 | | 65 | | 57 | |
| 37.8298 | | 47 | | 60 | | 61 | | 67 | |
| 37.8502 | | 53 | | 67 | | 55 | | 67 | |
| 37.8707 | | 59 | | 74 | | 45 | | 49 | |
| 37.8911 | | 43 | | 68 | | 67 | | 76 | |
| 37.9116 | | 54 | | 57 | | 44 | | 48 | |
| 37.932 | | 57 | | 69 | | 47 | | 60 | |
| 37.9525 | | 59 | | 49 | | 68 | | 55 | |
| 37.9729 | | 59 | | 55 | | 47 | | 62 | |
| 37.9934 | | 49 | | 43 | | 63 | | 54 | |
| 38.0138 | | 60 | | 59 | | 60 | | 61 | |
| 38.0343 | | 66 | | 69 | | 66 | | 50 | |
| 38.0547 | | 49 | | 53 | | 55 | | 62 | |
| 38.0752 | | 53 | | 63 | | 48 | | 62 | |
| 38.0956 | | 55 | | 80 | | 54 | | 42 | |
| 38.1161 | | 81 | | 55 | | 57 | | 63 | |
| 38.1365 | | 75 | | 68 | | 73 | | 75 | |
| 38.157 | | 101 | | 97 | | 105 | | 96 | |
| 38.1774 | | 135 | | 126 | | 100 | | 106 | |
| 38.1979 | | 121 | | 101 | | 109 | | 94 | |
| 38.2183 | | 77 | | 77 | | 69 | | 72 | |
| 38.2388 | | 56 | | 68 | | 64 | | 52 | |
| 38.2593 | | 68 | | 58 | | 69 | | 51 | |
| 38.2797 | | 62 | | 56 | | 54 | | 42 | |
| 38.3002 | | 54 | | 55 | | 52 | | 61 | |
| 38.3206 | | 55 | | 66 | | 53 | | 59 | |
| 38.3411 | | 52 | | 49 | | 61 | | 58 | |
| 38.3615 | | 45 | | 63 | | 61 | | 51 | |
| 38.382 | | 60 | | 55 | | 59 | | 51 | |
| 38.4024 | | 50 | | 38 | | 46 | | 64 | |
| 38.4229 | | 58 | | 62 | | 54 | | 60 | |
| 38.4433 | | 67 | | 60 | | 52 | | 62 | |
| 38.4638 | | 52 | | 53 | | 74 | | 55 | |
| 38.4842 | | 43 | | 62 | | 71 | | 57 | |
| 38.5047 | | 65 | | 58 | | 60 | | 73 | |
| 38.5251 | | 62 | | 60 | | 63 | | 48 | |
| 38.5456 | | 67 | | 52 | | 72 | | 60 | |
| 38.566 | | 64 | | 69 | | 56 | | 45 | |
| 38.5865 | | 53 | | 67 | | 67 | | 60 | |
| 38.6069 | | 53 | | 68 | | 64 | | 53 | |
| 38.6274 | | 52 | | 57 | | 45 | | 70 | |
| 38.6478 | | 46 | | 57 | | 59 | | 65 | |
| 38.6683 | | 55 | | 54 | | 61 | | 57 | |
| 38.6887 | | 35 | | 58 | | 61 | | 64 | |
| 38.7092 | | 61 | | 64 | | 55 | | 60 | |
| 38.7296 | | 59 | | 38 | | 49 | | 55 | |
| 38.7501 | | 54 | | 72 | | 45 | | 51 | |
| 38.7706 | | 46 | | 70 | | 55 | | 49 | |
| 38.791 | | 57 | | 65 | | 58 | | 69 | |
| 38.8115 | | 53 | | 55 | | 47 | | 53 | |
| 38.8319 | | 63 | | 72 | | 61 | | 54 | |
| 38.8524 | | 56 | | 65 | | 76 | | 56 | |
| 38.8728 | | 61 | | 77 | | 69 | | 57 | |
| 38.8933 | | 61 | | 81 | | 44 | | 56 | |
| 38.9137 | | 51 | | 56 | | 54 | | 67 | |
| 38.9342 | | 54 | | 73 | | 60 | | 46 | |
| 38.9546 | | 52 | | 63 | | 71 | | 54 | |
| 38.9751 | | 55 | | 58 | | 62 | | 59 | |
| 38.9955 | | 52 | | 63 | | 51 | | 54 | |
| 39.016 | | 61 | | 70 | | 53 | | 78 | |
| 39.0364 | | 63 | | 66 | | 75 | | 57 | |
| 39.0569 | | 63 | | 58 | | 59 | | 55 | |
| 39.0773 | | 61 | | 65 | | 61 | | 53 | |
| 39.0978 | | 72 | | 70 | | 56 | | 48 | |
| 39.1182 | | 75 | | 67 | | 70 | | 65 | |
| 39.1387 | | 52 | | 104 | | 86 | | 61 | |
| 39.1591 | | 64 | | 88 | | 71 | | 74 | |
| 39.1796 | | 79 | | 87 | | 79 | | 102 | |
| 39.2 | | 71 | | 85 | | 71 | | 70 | |
| 39.2205 | | 85 | | 99 | | 83 | | 95 | |
| 39.2409 | | 80 | | 123 | | 115 | | 103 | |
| 39.2614 | | 99 | | 93 | | 117 | | 104 | |
| 39.2818 | | 144 | | 127 | | 114 | | 136 | |
| 39.3023 | | 111 | | 158 | | 136 | | 150 | |
| 39.3228 | | 150 | | 159 | | 150 | | 140 | |
| 39.3432 | | 172 | | 203 | | 159 | | 189 | |
| 39.3637 | | 236 | | 263 | | 239 | | 270 | |
| 39.3841 | | 409 | | 459 | | 398 | | 413 | |
| 39.4046 | | 788 | | 703 | | 694 | | 748 | |
| 39.425 | | 1740 | | 1363 | | 1426 | | 1597 | |
| 39.4455 | | 3043 | | 2640 | | 2588 | | 2773 | |
| 39.4659 | | 2673 | | 2924 | | 2794 | | 2656 | |
| 39.4864 | | 939 | | 1407 | | 1187 | | 1228 | |
| 39.5068 | | 628 | | 746 | | 678 | | 765 | |
| 39.5273 | | 1021 | | 929 | | 927 | | 982 | |
| 39.5477 | | 1589 | | 1416 | | 1344 | | 1493 | |
| 39.5682 | | 1351 | | 1637 | | 1439 | | 1425 | |
| 39.5886 | | 496 | | 743 | | 583 | | 645 | |
| 39.6091 | | 224 | | 283 | | 241 | | 256 | |
| 39.6295 | | 168 | | 188 | | 196 | | 185 | |
| 39.65 | | 116 | | 155 | | 159 | | 161 | |
| 39.6704 | | 90 | | 129 | | 104 | | 130 | |
| 39.6909 | | 101 | | 105 | | 91 | | 100 | |
| 39.7113 | | 94 | | 122 | | 92 | | 97 | |
| 39.7318 | | 91 | | 100 | | 82 | | 96 | |
| 39.7522 | | 85 | | 75 | | 83 | | 75 | |
| 39.7727 | | 73 | | 77 | | 71 | | 85 | |
| 39.7931 | | 87 | | 96 | | 70 | | 78 | |
| 39.8136 | | 91 | | 101 | | 71 | | 89 | |
| 39.8341 | | 72 | | 80 | | 82 | | 65 | |
| 39.8545 | | 61 | | 82 | | 69 | | 79 | |
| 39.875 | | 66 | | 71 | | 79 | | 76 | |
| 39.8954 | | 71 | | 73 | | 71 | | 69 | |
| 39.9159 | | 64 | | 62 | | 89 | | 64 | |
| 39.9363 | | 90 | | 79 | | 82 | | 73 | |
| 39.9568 | | 59 | | 69 | | 70 | | 78 | |
| 39.9772 | | 59 | | 78 | | 71 | | 77 | |
| 39.9977 | | 72 | | 85 | | 71 | | 64 | |
| 40.0181 | | 81 | | 88 | | 77 | | 77 | |
| 40.0386 | | 72 | | 85 | | 74 | | 98 | |
| 40.059 | | 82 | | 100 | | 86 | | 93 | |
| 40.0795 | | 81 | | 112 | | 90 | | 97 | |
| 40.0999 | | 109 | | 97 | | 126 | | 103 | |
| 40.1204 | | 113 | | 121 | | 98 | | 115 | |
| 40.1408 | | 95 | | 133 | | 116 | | 128 | |
| 40.1613 | | 143 | | 123 | | 167 | | 145 | |
| 40.1817 | | 143 | | 165 | | 164 | | 182 | |
| 40.2022 | | 269 | | 224 | | 264 | | 260 | |
| 40.2226 | | 465 | | 345 | | 390 | | 454 | |
| 40.2431 | | 846 | | 662 | | 722 | | 747 | |
| 40.2635 | | 1606 | | 1107 | | 1265 | | 1354 | |
| 40.284 | | 1768 | | 1525 | | 1663 | | 1570 | |
| 40.3044 | | 872 | | 1050 | | 1062 | | 941 | |
| 40.3249 | | 498 | | 493 | | 563 | | 539 | |
| 40.3454 | | 561 | | 487 | | 498 | | 557 | |
| 40.3658 | | 818 | | 638 | | 701 | | 751 | |
| 40.3863 | | 999 | | 838 | | 898 | | 912 | |
| 40.4067 | | 501 | | 558 | | 633 | | 564 | |
| 40.4272 | | 248 | | 282 | | 274 | | 290 | |
| 40.4476 | | 158 | | 146 | | 171 | | 169 | |
| 40.4681 | | 105 | | 110 | | 159 | | 155 | |
| 40.4885 | | 96 | | 97 | | 111 | | 113 | |
| 40.509 | | 100 | | 96 | | 108 | | 94 | |
| 40.5294 | | 93 | | 85 | | 94 | | 95 | |
| 40.5499 | | 78 | | 84 | | 85 | | 71 | |
| 40.5703 | | 72 | | 75 | | 81 | | 89 | |
| 40.5908 | | 76 | | 72 | | 86 | | 78 | |
| 40.6112 | | 74 | | 82 | | 88 | | 85 | |
| 40.6317 | | 74 | | 64 | | 70 | | 73 | |
| 40.6521 | | 61 | | 72 | | 66 | | 61 | |
| 40.6726 | | 70 | | 65 | | 51 | | 73 | |
| 40.693 | | 52 | | 61 | | 62 | | 72 | |
| 40.7135 | | 68 | | 62 | | 48 | | 67 | |
| 40.7339 | | 47 | | 62 | | 62 | | 69 | |
| 40.7544 | | 59 | | 56 | | 67 | | 61 | |
| 40.7748 | | 62 | | 59 | | 66 | | 58 | |
| 40.7953 | | 71 | | 74 | | 63 | | 66 | |
| 40.8157 | | 60 | | 68 | | 48 | | 53 | |
| 40.8362 | | 48 | | 69 | | 55 | | 47 | |
| 40.8566 | | 46 | | 66 | | 54 | | 62 | |
| 40.8771 | | 55 | | 43 | | 57 | | 62 | |
| 40.8976 | | 67 | | 58 | | 61 | | 66 | |
| 40.918 | | 58 | | 56 | | 54 | | 48 | |
| 40.9385 | | 59 | | 51 | | 50 | | 61 | |
| 40.9589 | | 53 | | 57 | | 65 | | 61 | |
| 40.9794 | | 54 | | 44 | | 68 | | 61 | |
| 40.9998 | | 60 | | 54 | | 62 | | 47 | |
| 41.0203 | | 70 | | 52 | | 56 | | 60 | |
| 41.0407 | | 72 | | 60 | | 58 | | 57 | |
| 41.0612 | | 51 | | 66 | | 57 | | 68 | |
| 41.0816 | | 71 | | 70 | | 70 | | 73 | |
| 41.1021 | | 67 | | 62 | | 82 | | 67 | |
| 41.1225 | | 71 | | 87 | | 63 | | 81 | |
| 41.143 | | 108 | | 97 | | 68 | | 84 | |
| 41.1634 | | 89 | | 94 | | 83 | | 80 | |
| 41.1839 | | 57 | | 78 | | 80 | | 74 | |
| 41.2043 | | 59 | | 65 | | 51 | | 64 | |
| 41.2248 | | 47 | | 49 | | 55 | | 46 | |
| 41.2452 | | 56 | | 52 | | 56 | | 64 | |
| 41.2657 | | 46 | | 71 | | 56 | | 51 | |
| 41.2861 | | 55 | | 69 | | 44 | | 51 | |
| 41.3066 | | 61 | | 67 | | 61 | | 60 | |
| 41.327 | | 43 | | 51 | | 57 | | 52 | |
| 41.3475 | | 65 | | 58 | | 59 | | 55 | |
| 41.3679 | | 51 | | 43 | | 51 | | 50 | |
| 41.3884 | | 54 | | 64 | | 53 | | 46 | |
| 41.4089 | | 64 | | 52 | | 60 | | 54 | |
| 41.4293 | | 61 | | 54 | | 55 | | 49 | |
| 41.4498 | | 41 | | 47 | | 43 | | 48 | |
| 41.4702 | | 61 | | 45 | | 52 | | 60 | |
| 41.4907 | | 57 | | 47 | | 54 | | 42 | |
| 41.5111 | | 53 | | 60 | | 59 | | 45 | |
| 41.5316 | | 59 | | 45 | | 61 | | 63 | |
| 41.552 | | 51 | | 54 | | 57 | | 58 | |
| 41.5725 | | 64 | | 64 | | 56 | | 48 | |
| 41.5929 | | 53 | | 65 | | 51 | | 44 | |
| 41.6134 | | 45 | | 67 | | 57 | | 56 | |
| 41.6338 | | 64 | | 63 | | 56 | | 56 | |
| 41.6543 | | 55 | | 56 | | 53 | | 64 | |
| 41.6747 | | 47 | | 65 | | 46 | | 69 | |
| 41.6952 | | 58 | | 48 | | 52 | | 54 | |
| 41.7156 | | 53 | | 68 | | 58 | | 54 | |
| 41.7361 | | 61 | | 63 | | 67 | | 63 | |
| 41.7565 | | 44 | | 66 | | 62 | | 50 | |
| 41.777 | | 54 | | 45 | | 54 | | 66 | |
| 41.7974 | | 48 | | 45 | | 53 | | 47 | |
| 41.8179 | | 44 | | 56 | | 52 | | 62 | |
| 41.8383 | | 66 | | 51 | | 51 | | 48 | |
| 41.8588 | | 51 | | 46 | | 44 | | 50 | |
| 41.8792 | | 56 | | 58 | | 51 | | 53 | |
| 41.8997 | | 52 | | 52 | | 52 | | 69 | |
| 41.9202 | | 54 | | 58 | | 67 | | 50 | |
| 41.9406 | | 50 | | 53 | | 55 | | 55 | |
| 41.9611 | | 45 | | 60 | | 71 | | 75 | |
| 41.9815 | | 62 | | 49 | | 58 | | 51 | |
| 42.002 | | 53 | | 63 | | 66 | | 53 | |
| 42.0224 | | 48 | | 61 | | 49 | | 62 | |
| 42.0429 | | 54 | | 72 | | 62 | | 60 | |
| 42.0633 | | 71 | | 55 | | 71 | | 62 | |
| 42.0838 | | 55 | | 76 | | 61 | | 62 | |
| 42.1042 | | 40 | | 84 | | 77 | | 78 | |
| 42.1247 | | 77 | | 64 | | 63 | | 67 | |
| 42.1451 | | 69 | | 70 | | 71 | | 73 | |
| 42.1656 | | 66 | | 83 | | 86 | | 70 | |
| 42.186 | | 96 | | 77 | | 88 | | 85 | |
| 42.2065 | | 83 | | 92 | | 89 | | 86 | |
| 42.2269 | | 93 | | 101 | | 96 | | 99 | |
| 42.2474 | | 110 | | 102 | | 113 | | 97 | |
| 42.2678 | | 88 | | 111 | | 91 | | 136 | |
| 42.2883 | | 136 | | 113 | | 113 | | 128 | |
| 42.3087 | | 134 | | 145 | | 130 | | 143 | |
| 42.3292 | | 174 | | 176 | | 152 | | 181 | |
| 42.3496 | | 252 | | 234 | | 214 | | 247 | |
| 42.3701 | | 372 | | 336 | | 329 | | 440 | |
| 42.3905 | | 712 | | 621 | | 634 | | 829 | |
| 42.411 | | 1621 | | 1141 | | 1204 | | 1507 | |
| 42.4314 | | 2677 | | 2061 | | 2134 | | 2277 | |
| 42.4519 | | 2191 | | 2223 | | 2116 | | 1900 | |
| 42.4724 | | 788 | | 1051 | | 961 | | 1004 | |
| 42.4928 | | 550 | | 551 | | 514 | | 604 | |
| 42.5133 | | 710 | | 632 | | 614 | | 769 | |
| 42.5337 | | 1166 | | 943 | | 955 | | 1109 | |
| 42.5542 | | 1395 | | 1237 | | 1188 | | 1136 | |
| 42.5746 | | 671 | | 778 | | 710 | | 696 | |
| 42.5951 | | 277 | | 338 | | 264 | | 352 | |
| 42.6155 | | 156 | | 213 | | 150 | | 175 | |
| 42.636 | | 105 | | 156 | | 141 | | 138 | |
| 42.6564 | | 90 | | 127 | | 109 | | 99 | |
| 42.6769 | | 86 | | 81 | | 117 | | 96 | |
| 42.6973 | | 84 | | 76 | | 83 | | 101 | |
| 42.7178 | | 81 | | 76 | | 72 | | 76 | |
| 42.7382 | | 70 | | 67 | | 65 | | 90 | |
| 42.7587 | | 72 | | 67 | | 61 | | 69 | |
| 42.7791 | | 51 | | 50 | | 60 | | 68 | |
| 42.7996 | | 52 | | 65 | | 65 | | 61 | |
| 42.82 | | 62 | | 69 | | 62 | | 50 | |
| 42.8405 | | 51 | | 72 | | 57 | | 60 | |
| 42.8609 | | 55 | | 69 | | 67 | | 39 | |
| 42.8814 | | 56 | | 62 | | 62 | | 70 | |
| 42.9018 | | 55 | | 55 | | 68 | | 56 | |
| 42.9223 | | 65 | | 53 | | 65 | | 54 | |
| 42.9427 | | 50 | | 66 | | 72 | | 56 | |
| 42.9632 | | 51 | | 59 | | 52 | | 51 | |
| 42.9837 | | 50 | | 59 | | 69 | | 66 | |
| 43.0041 | | 51 | | 55 | | 51 | | 51 | |
| 43.0246 | | 47 | | 57 | | 60 | | 67 | |
| 43.045 | | 45 | | 68 | | 65 | | 56 | |
| 43.0655 | | 52 | | 64 | | 49 | | 48 | |
| 43.0859 | | 57 | | 56 | | 53 | | 60 | |
| 43.1064 | | 56 | | 52 | | 53 | | 46 | |
| 43.1268 | | 52 | | 44 | | 41 | | 48 | |
| 43.1473 | | 65 | | 55 | | 58 | | 51 | |
| 43.1677 | | 56 | | 56 | | 63 | | 43 | |
| 43.1882 | | 55 | | 66 | | 54 | | 51 | |
| 43.2086 | | 49 | | 56 | | 54 | | 44 | |
| 43.2291 | | 60 | | 61 | | 56 | | 52 | |
| 43.2495 | | 41 | | 42 | | 53 | | 65 | |
| 43.27 | | 48 | | 55 | | 42 | | 64 | |
| 43.2904 | | 51 | | 65 | | 40 | | 47 | |
| 43.3109 | | 40 | | 51 | | 45 | | 48 | |
| 43.3313 | | 43 | | 52 | | 60 | | 52 | |
| 43.3518 | | 53 | | 61 | | 49 | | 49 | |
| 43.3722 | | 34 | | 48 | | 36 | | 59 | |
| 43.3927 | | 54 | | 47 | | 48 | | 53 | |
| 43.4131 | | 46 | | 70 | | 53 | | 44 | |
| 43.4336 | | 52 | | 61 | | 61 | | 46 | |
| 43.454 | | 42 | | 50 | | 39 | | 46 | |
| 43.4745 | | 47 | | 50 | | 62 | | 59 | |
| 43.495 | | 50 | | 48 | | 53 | | 58 | |
| 43.5154 | | 46 | | 69 | | 48 | | 53 | |
| 43.5359 | | 49 | | 64 | | 63 | | 59 | |
| 43.5563 | | 39 | | 56 | | 49 | | 45 | |
| 43.5768 | | 42 | | 57 | | 53 | | 46 | |
| 43.5972 | | 48 | | 52 | | 61 | | 56 | |
| 43.6177 | | 39 | | 55 | | 50 | | 43 | |
| 43.6381 | | 57 | | 69 | | 46 | | 47 | |
| 43.6586 | | 44 | | 58 | | 48 | | 65 | |
| 43.679 | | 51 | | 49 | | 45 | | 46 | |
| 43.6995 | | 45 | | 51 | | 50 | | 40 | |
| 43.7199 | | 50 | | 62 | | 55 | | 42 | |
| 43.7404 | | 45 | | 65 | | 37 | | 51 | |
| 43.7608 | | 48 | | 59 | | 57 | | 63 | |
| 43.7813 | | 41 | | 63 | | 61 | | 46 | |
| 43.8017 | | 65 | | 48 | | 51 | | 50 | |
| 43.8222 | | 57 | | 40 | | 38 | | 42 | |
| 43.8426 | | 44 | | 52 | | 48 | | 64 | |
| 43.8631 | | 36 | | 59 | | 43 | | 39 | |
| 43.8835 | | 55 | | 56 | | 52 | | 51 | |
| 43.904 | | 43 | | 39 | | 48 | | 61 | |
| 43.9244 | | 45 | | 55 | | 60 | | 36 | |
| 43.9449 | | 50 | | 67 | | 47 | | 55 | |
| 43.9653 | | 52 | | 44 | | 42 | | 50 | |
| 43.9858 | | 56 | | 49 | | 57 | | 58 | |
| 44.0062 | | 43 | | 43 | | 43 | | 41 | |
| 44.0267 | | 49 | | 49 | | 40 | | 39 | |
| 44.0472 | | 51 | | 55 | | 38 | | 64 | |
| 44.0676 | | 47 | | 42 | | 52 | | 51 | |
| 44.0881 | | 59 | | 43 | | 51 | | 47 | |
| 44.1085 | | 59 | | 58 | | 39 | | 39 | |
| 44.129 | | 51 | | 44 | | 61 | | 38 | |
| 44.1494 | | 49 | | 53 | | 50 | | 38 | |
| 44.1699 | | 60 | | 54 | | 50 | | 43 | |
| 44.1903 | | 54 | | 45 | | 61 | | 45 | |
| 44.2108 | | 45 | | 55 | | 60 | | 60 | |
| 44.2312 | | 40 | | 48 | | 46 | | 43 | |
| 44.2517 | | 49 | | 48 | | 54 | | 54 | |
| 44.2721 | | 54 | | 55 | | 45 | | 43 | |
| 44.2926 | | 46 | | 48 | | 46 | | 57 | |
| 44.313 | | 37 | | 47 | | 41 | | 47 | |
| 44.3335 | | 52 | | 54 | | 52 | | 72 | |
| 44.3539 | | 46 | | 40 | | 52 | | 58 | |
| 44.3744 | | 49 | | 39 | | 58 | | 53 | |
| 44.3948 | | 49 | | 57 | | 44 | | 42 | |
| 44.4153 | | 41 | | 55 | | 45 | | 50 | |
| 44.4357 | | 46 | | 45 | | 50 | | 45 | |
| 44.4562 | | 49 | | 59 | | 42 | | 56 | |
| 44.4766 | | 48 | | 54 | | 44 | | 47 | |
| 44.4971 | | 51 | | 50 | | 36 | | 45 | |
| 44.5175 | | 41 | | 57 | | 55 | | 53 | |
| 44.538 | | 49 | | 49 | | 44 | | 56 | |
| 44.5585 | | 47 | | 48 | | 58 | | 44 | |
| 44.5789 | | 30 | | 49 | | 63 | | 39 | |
| 44.5994 | | 44 | | 72 | | 51 | | 50 | |
| 44.6198 | | 48 | | 44 | | 54 | | 54 | |
| 44.6403 | | 48 | | 60 | | 71 | | 57 | |
| 44.6607 | | 48 | | 58 | | 67 | | 53 | |
| 44.6812 | | 61 | | 64 | | 55 | | 50 | |
| 44.7016 | | 48 | | 61 | | 42 | | 40 | |
| 44.7221 | | 55 | | 64 | | 54 | | 51 | |
| 44.7425 | | 53 | | 55 | | 53 | | 56 | |
| 44.763 | | 55 | | 33 | | 48 | | 56 | |
| 44.7834 | | 60 | | 56 | | 49 | | 46 | |
| 44.8039 | | 56 | | 67 | | 56 | | 57 | |
| 44.8243 | | 62 | | 55 | | 53 | | 43 | |
| 44.8448 | | 50 | | 59 | | 43 | | 47 | |
| 44.8652 | | 48 | | 71 | | 57 | | 46 | |
| 44.8857 | | 60 | | 62 | | 53 | | 62 | |
| 44.9061 | | 71 | | 79 | | 60 | | 53 | |
| 44.9266 | | 55 | | 76 | | 58 | | 65 | |
| 44.947 | | 68 | | 88 | | 89 | | 90 | |
| 44.9675 | | 91 | | 108 | | 104 | | 112 | |
| 44.9879 | | 150 | | 154 | | 165 | | 138 | |
| 45.0084 | | 163 | | 191 | | 186 | | 175 | |
| 45.0288 | | 157 | | 181 | | 177 | | 154 | |
| 45.0493 | | 101 | | 135 | | 117 | | 114 | |
| 45.0697 | | 65 | | 91 | | 96 | | 83 | |
| 45.0902 | | 68 | | 83 | | 68 | | 64 | |
| 45.1107 | | 53 | | 64 | | 55 | | 59 | |
| 45.1311 | | 64 | | 68 | | 63 | | 58 | |
| 45.1516 | | 65 | | 62 | | 58 | | 50 | |
| 45.172 | | 51 | | 59 | | 58 | | 53 | |
| 45.1925 | | 51 | | 64 | | 54 | | 60 | |
| 45.2129 | | 55 | | 50 | | 48 | | 56 | |
| 45.2334 | | 48 | | 52 | | 55 | | 59 | |
| 45.2538 | | 53 | | 65 | | 65 | | 49 | |
| 45.2743 | | 57 | | 52 | | 58 | | 55 | |
| 45.2947 | | 56 | | 52 | | 61 | | 76 | |
| 45.3152 | | 47 | | 52 | | 62 | | 62 | |
| 45.3356 | | 61 | | 63 | | 69 | | 48 | |
| 45.3561 | | 66 | | 48 | | 59 | | 65 | |
| 45.3765 | | 58 | | 52 | | 53 | | 47 | |
| 45.397 | | 57 | | 61 | | 60 | | 50 | |
| 45.4174 | | 54 | | 66 | | 55 | | 61 | |
| 45.4379 | | 54 | | 61 | | 82 | | 75 | |
| 45.4583 | | 51 | | 59 | | 75 | | 65 | |
| 45.4788 | | 62 | | 58 | | 66 | | 72 | |
| 45.4992 | | 61 | | 57 | | 62 | | 52 | |
| 45.5197 | | 51 | | 79 | | 64 | | 73 | |
| 45.5401 | | 76 | | 103 | | 71 | | 79 | |
| 45.5606 | | 68 | | 94 | | 72 | | 84 | |
| 45.581 | | 80 | | 103 | | 80 | | 84 | |
| 45.6015 | | 76 | | 101 | | 75 | | 86 | |
| 45.622 | | 107 | | 97 | | 87 | | 81 | |
| 45.6424 | | 91 | | 104 | | 105 | | 116 | |
| 45.6629 | | 117 | | 130 | | 125 | | 132 | |
| 45.6833 | | 166 | | 163 | | 141 | | 197 | |
| 45.7038 | | 245 | | 209 | | 221 | | 264 | |
| 45.7242 | | 406 | | 361 | | 372 | | 466 | |
| 45.7447 | | 791 | | 585 | | 679 | | 773 | |
| 45.7651 | | 1561 | | 1093 | | 1217 | | 1294 | |
| 45.7856 | | 1758 | | 1545 | | 1599 | | 1512 | |
| 45.806 | | 777 | | 1039 | | 952 | | 943 | |
| 45.8265 | | 339 | | 468 | | 448 | | 409 | |
| 45.8469 | | 368 | | 369 | | 347 | | 395 | |
| 45.8674 | | 483 | | 400 | | 475 | | 532 | |
| 45.8878 | | 863 | | 670 | | 706 | | 756 | |
| 45.9083 | | 803 | | 842 | | 786 | | 835 | |
| 45.9287 | | 407 | | 524 | | 513 | | 467 | |
| 45.9492 | | 194 | | 262 | | 214 | | 199 | |
| 45.9696 | | 115 | | 126 | | 144 | | 138 | |
| 45.9901 | | 99 | | 109 | | 111 | | 121 | |
| 46.0105 | | 78 | | 96 | | 85 | | 102 | |
| 46.031 | | 84 | | 77 | | 76 | | 79 | |
| 46.0514 | | 51 | | 79 | | 68 | | 78 | |
| 46.0719 | | 65 | | 69 | | 69 | | 79 | |
| 46.0923 | | 52 | | 68 | | 64 | | 72 | |
| 46.1128 | | 67 | | 70 | | 65 | | 66 | |
| 46.1333 | | 54 | | 71 | | 49 | | 52 | |
| 46.1537 | | 70 | | 58 | | 61 | | 52 | |
| 46.1742 | | 54 | | 62 | | 58 | | 64 | |
| 46.1946 | | 41 | | 62 | | 48 | | 58 | |
| 46.2151 | | 45 | | 61 | | 47 | | 63 | |
| 46.2355 | | 59 | | 49 | | 42 | | 43 | |
| 46.256 | | 49 | | 49 | | 57 | | 66 | |
| 46.2764 | | 47 | | 54 | | 56 | | 56 | |
| 46.2969 | | 48 | | 56 | | 66 | | 63 | |
| 46.3173 | | 51 | | 58 | | 58 | | 45 | |
| 46.3378 | | 48 | | 59 | | 57 | | 50 | |
| 46.3582 | | 39 | | 50 | | 42 | | 49 | |
| 46.3787 | | 45 | | 55 | | 55 | | 60 | |
| 46.3991 | | 53 | | 66 | | 56 | | 53 | |
| 46.4196 | | 55 | | 59 | | 52 | | 52 | |
| 46.44 | | 37 | | 43 | | 55 | | 47 | |
| 46.4605 | | 49 | | 51 | | 62 | | 53 | |
| 46.4809 | | 46 | | 44 | | 59 | | 62 | |
| 46.5014 | | 43 | | 57 | | 49 | | 51 | |
| 46.5218 | | 42 | | 51 | | 49 | | 47 | |
| 46.5423 | | 50 | | 46 | | 58 | | 58 | |
| 46.5627 | | 49 | | 50 | | 60 | | 50 | |
| 46.5832 | | 44 | | 65 | | 42 | | 54 | |
| 46.6036 | | 49 | | 51 | | 45 | | 37 | |
| 46.6241 | | 47 | | 41 | | 51 | | 51 | |
| 46.6445 | | 57 | | 48 | | 54 | | 58 | |
| 46.665 | | 50 | | 58 | | 53 | | 51 | |
| 46.6855 | | 42 | | 64 | | 43 | | 47 | |
| 46.7059 | | 43 | | 48 | | 52 | | 49 | |
| 46.7264 | | 49 | | 61 | | 63 | | 51 | |
| 46.7468 | | 39 | | 46 | | 42 | | 54 | |
| 46.7673 | | 52 | | 65 | | 47 | | 42 | |
| 46.7877 | | 43 | | 52 | | 33 | | 47 | |
| 46.8082 | | 39 | | 54 | | 44 | | 54 | |
| 46.8286 | | 44 | | 36 | | 52 | | 60 | |
| 46.8491 | | 48 | | 55 | | 58 | | 36 | |
| 46.8695 | | 47 | | 51 | | 43 | | 56 | |
| 46.89 | | 64 | | 48 | | 41 | | 60 | |
| 46.9104 | | 39 | | 40 | | 50 | | 48 | |
| 46.9309 | | 39 | | 53 | | 44 | | 40 | |
| 46.9513 | | 47 | | 56 | | 58 | | 48 | |
| 46.9718 | | 38 | | 64 | | 46 | | 57 | |
| 46.9922 | | 48 | | 54 | | 60 | | 60 | |
| 47.0127 | | 47 | | 54 | | 37 | | 48 | |
| 47.0331 | | 52 | | 55 | | 45 | | 39 | |
| 47.0536 | | 53 | | 46 | | 44 | | 46 | |
| 47.074 | | 44 | | 39 | | 54 | | 53 | |
| 47.0945 | | 47 | | 55 | | 49 | | 46 | |
| 47.1149 | | 41 | | 44 | | 41 | | 48 | |
| 47.1354 | | 36 | | 46 | | 54 | | 44 | |
| 47.1558 | | 45 | | 44 | | 54 | | 54 | |
| 47.1763 | | 33 | | 54 | | 45 | | 53 | |
| 47.1968 | | 54 | | 46 | | 50 | | 55 | |
| 47.2172 | | 45 | | 47 | | 50 | | 46 | |
| 47.2377 | | 41 | | 71 | | 56 | | 57 | |
| 47.2581 | | 36 | | 40 | | 53 | | 44 | |
| 47.2786 | | 47 | | 55 | | 49 | | 44 | |
| 47.299 | | 41 | | 48 | | 49 | | 41 | |
| 47.3195 | | 50 | | 61 | | 49 | | 70 | |
| 47.3399 | | 65 | | 62 | | 51 | | 35 | |
| 47.3604 | | 39 | | 47 | | 45 | | 50 | |
| 47.3808 | | 44 | | 49 | | 46 | | 38 | |
| 47.4013 | | 48 | | 49 | | 55 | | 53 | |
| 47.4217 | | 49 | | 67 | | 43 | | 47 | |
| 47.4422 | | 34 | | 49 | | 57 | | 49 | |
| 47.4626 | | 41 | | 53 | | 41 | | 61 | |
| 47.4831 | | 36 | | 64 | | 44 | | 57 | |
| 47.5035 | | 57 | | 51 | | 43 | | 51 | |
| 47.524 | | 44 | | 47 | | 62 | | 59 | |
| 47.5444 | | 50 | | 52 | | 65 | | 52 | |
| 47.5649 | | 59 | | 55 | | 54 | | 33 | |
| 47.5853 | | 44 | | 42 | | 58 | | 34 | |
| 47.6058 | | 46 | | 56 | | 50 | | 59 | |
| 47.6262 | | 54 | | 53 | | 57 | | 47 | |
| 47.6467 | | 53 | | 47 | | 56 | | 58 | |
| 47.6671 | | 42 | | 46 | | 53 | | 58 | |
| 47.6876 | | 38 | | 64 | | 48 | | 46 | |
| 47.7081 | | 57 | | 60 | | 48 | | 54 | |
| 47.7285 | | 59 | | 57 | | 50 | | 49 | |
| 47.749 | | 42 | | 46 | | 55 | | 46 | |
| 47.7694 | | 50 | | 61 | | 53 | | 43 | |
| 47.7899 | | 50 | | 44 | | 47 | | 55 | |
| 47.8103 | | 63 | | 49 | | 63 | | 56 | |
| 47.8308 | | 41 | | 47 | | 61 | | 65 | |
| 47.8512 | | 66 | | 59 | | 66 | | 60 | |
| 47.8717 | | 57 | | 40 | | 57 | | 50 | |
| 47.8921 | | 69 | | 77 | | 68 | | 77 | |
| 47.9126 | | 57 | | 68 | | 78 | | 62 | |
| 47.933 | | 57 | | 61 | | 56 | | 55 | |
| 47.9535 | | 82 | | 66 | | 56 | | 60 | |
| 47.9739 | | 58 | | 49 | | 56 | | 45 | |
| 47.9944 | | 54 | | 41 | | 62 | | 46 | |
| 48.0148 | | 47 | | 62 | | 57 | | 43 | |
| 48.0353 | | 46 | | 55 | | 57 | | 55 | |
| 48.0557 | | 44 | | 45 | | 60 | | 40 | |
| 48.0762 | | 48 | | 59 | | 58 | | 46 | |
| 48.0966 | | 43 | | 57 | | 40 | | 49 | |
| 48.1171 | | 49 | | 55 | | 52 | | 49 | |
| 48.1375 | | 44 | | 42 | | 74 | | 54 | |
| 48.158 | | 38 | | 50 | | 42 | | 52 | |
| 48.1784 | | 52 | | 67 | | 42 | | 49 | |
| 48.1989 | | 56 | | 59 | | 60 | | 60 | |
| 48.2193 | | 64 | | 53 | | 55 | | 53 | |
| 48.2398 | | 45 | | 64 | | 47 | | 45 | |
| 48.2603 | | 55 | | 62 | | 54 | | 57 | |
| 48.2807 | | 49 | | 49 | | 53 | | 57 | |
| 48.3012 | | 38 | | 62 | | 63 | | 53 | |
| 48.3216 | | 41 | | 75 | | 45 | | 52 | |
| 48.3421 | | 44 | | 53 | | 55 | | 54 | |
| 48.3625 | | 54 | | 54 | | 60 | | 61 | |
| 48.383 | | 62 | | 59 | | 52 | | 39 | |
| 48.4034 | | 53 | | 60 | | 46 | | 64 | |
| 48.4239 | | 56 | | 57 | | 52 | | 55 | |
| 48.4443 | | 47 | | 65 | | 49 | | 54 | |
| 48.4648 | | 43 | | 51 | | 45 | | 55 | |
| 48.4852 | | 53 | | 54 | | 61 | | 46 | |
| 48.5057 | | 47 | | 61 | | 53 | | 49 | |
| 48.5261 | | 48 | | 58 | | 41 | | 52 | |
| 48.5466 | | 53 | | 68 | | 59 | | 48 | |
| 48.567 | | 45 | | 58 | | 55 | | 46 | |
| 48.5875 | | 45 | | 62 | | 51 | | 47 | |
| 48.6079 | | 59 | | 53 | | 54 | | 62 | |
| 48.6284 | | 49 | | 46 | | 49 | | 46 | |
| 48.6488 | | 59 | | 41 | | 55 | | 57 | |
| 48.6693 | | 59 | | 47 | | 46 | | 47 | |
| 48.6897 | | 50 | | 61 | | 39 | | 51 | |
| 48.7102 | | 58 | | 57 | | 48 | | 63 | |
| 48.7306 | | 49 | | 70 | | 46 | | 61 | |
| 48.7511 | | 50 | | 47 | | 64 | | 42 | |
| 48.7716 | | 34 | | 49 | | 51 | | 54 | |
| 48.792 | | 61 | | 59 | | 46 | | 48 | |
| 48.8125 | | 58 | | 48 | | 51 | | 55 | |
| 48.8329 | | 51 | | 52 | | 52 | | 50 | |
| 48.8534 | | 46 | | 53 | | 58 | | 54 | |
| 48.8738 | | 47 | | 57 | | 62 | | 49 | |
| 48.8943 | | 47 | | 48 | | 48 | | 49 | |
| 48.9147 | | 48 | | 55 | | 51 | | 49 | |
| 48.9352 | | 48 | | 49 | | 45 | | 54 | |
| 48.9556 | | 56 | | 54 | | 66 | | 68 | |
| 48.9761 | | 61 | | 50 | | 63 | | 53 | |
| 48.9965 | | 56 | | 53 | | 63 | | 53 | |
| 49.017 | | 43 | | 62 | | 55 | | 58 | |
| 49.0374 | | 56 | | 56 | | 62 | | 57 | |
| 49.0579 | | 54 | | 53 | | 53 | | 52 | |
| 49.0783 | | 56 | | 49 | | 56 | | 38 | |
| 49.0988 | | 50 | | 65 | | 69 | | 61 | |
| 49.1192 | | 56 | | 75 | | 64 | | 54 | |
| 49.1397 | | 70 | | 65 | | 67 | | 78 | |
| 49.1601 | | 69 | | 78 | | 76 | | 91 | |
| 49.1806 | | 77 | | 90 | | 91 | | 96 | |
| 49.201 | | 98 | | 86 | | 99 | | 83 | |
| 49.2215 | | 75 | | 104 | | 81 | | 87 | |
| 49.2419 | | 77 | | 76 | | 76 | | 67 | |
| 49.2624 | | 48 | | 53 | | 65 | | 60 | |
| 49.2829 | | 64 | | 64 | | 50 | | 70 | |
| 49.3033 | | 52 | | 67 | | 56 | | 57 | |
| 49.3238 | | 46 | | 55 | | 60 | | 58 | |
| 49.3442 | | 60 | | 64 | | 62 | | 59 | |
| 49.3647 | | 57 | | 61 | | 61 | | 68 | |
| 49.3851 | | 50 | | 60 | | 73 | | 72 | |
| 49.4056 | | 67 | | 72 | | 57 | | 70 | |
| 49.426 | | 48 | | 55 | | 76 | | 71 | |
| 49.4465 | | 58 | | 77 | | 74 | | 65 | |
| 49.4669 | | 63 | | 79 | | 76 | | 52 | |
| 49.4874 | | 70 | | 71 | | 59 | | 69 | |
| 49.5078 | | 71 | | 77 | | 64 | | 64 | |
| 49.5283 | | 73 | | 65 | | 63 | | 77 | |
| 49.5487 | | 76 | | 83 | | 77 | | 79 | |
| 49.5692 | | 75 | | 84 | | 69 | | 62 | |
| 49.5896 | | 84 | | 72 | | 89 | | 83 | |
| 49.6101 | | 86 | | 82 | | 74 | | 85 | |
| 49.6305 | | 66 | | 91 | | 71 | | 80 | |
| 49.651 | | 68 | | 83 | | 68 | | 80 | |
| 49.6714 | | 68 | | 113 | | 80 | | 84 | |
| 49.6919 | | 73 | | 83 | | 69 | | 82 | |
| 49.7123 | | 64 | | 88 | | 83 | | 61 | |
| 49.7328 | | 75 | | 82 | | 101 | | 79 | |
| 49.7532 | | 73 | | 92 | | 84 | | 84 | |
| 49.7737 | | 89 | | 90 | | 82 | | 104 | |
| 49.7941 | | 79 | | 88 | | 93 | | 100 | |
| 49.8146 | | 110 | | 112 | | 107 | | 96 | |
| 49.8351 | | 121 | | 114 | | 93 | | 131 | |
| 49.8555 | | 128 | | 132 | | 132 | | 117 | |
| 49.876 | | 141 | | 163 | | 167 | | 165 | |
| 49.8964 | | 166 | | 168 | | 137 | | 190 | |
| 49.9169 | | 164 | | 182 | | 174 | | 175 | |
| 49.9373 | | 185 | | 184 | | 190 | | 174 | |
| 49.9578 | | 195 | | 249 | | 212 | | 205 | |
| 49.9782 | | 221 | | 273 | | 258 | | 274 | |
| 49.9987 | | 279 | | 336 | | 324 | | 325 | |
| 50.0191 | | 385 | | 417 | | 437 | | 460 | |
| 50.0396 | | 619 | | 677 | | 591 | | 645 | |
| 50.06 | | 1017 | | 957 | | 1002 | | 1161 | |
| 50.0805 | | 1968 | | 1604 | | 1785 | | 2126 | |
| 50.1009 | | 3727 | | 3074 | | 3328 | | 3859 | |
| 50.1214 | | 5770 | | 4952 | | 5488 | | 5523 | |
| 50.1418 | | 4393 | | 4946 | | 4960 | | 4423 | |
| 50.1623 | | 1772 | | 2376 | | 2140 | | 2196 | |
| 50.1827 | | 979 | | 1205 | | 1136 | | 1278 | |
| 50.2032 | | 1090 | | 1025 | | 1058 | | 1184 | |
| 50.2236 | | 1613 | | 1328 | | 1414 | | 1728 | |
| 50.2441 | | 2509 | | 2051 | | 2432 | | 2547 | |
| 50.2645 | | 3016 | | 2800 | | 2996 | | 2759 | |
| 50.285 | | 1499 | | 1952 | | 1801 | | 1707 | |
| 50.3054 | | 594 | | 815 | | 738 | | 791 | |
| 50.3259 | | 332 | | 447 | | 370 | | 493 | |
| 50.3464 | | 236 | | 288 | | 293 | | 293 | |
| 50.3668 | | 207 | | 220 | | 211 | | 246 | |
| 50.3873 | | 152 | | 206 | | 201 | | 209 | |
| 50.4077 | | 132 | | 144 | | 142 | | 168 | |
| 50.4282 | | 139 | | 135 | | 128 | | 137 | |
| 50.4486 | | 94 | | 132 | | 127 | | 121 | |
| 50.4691 | | 83 | | 107 | | 120 | | 125 | |
| 50.4895 | | 84 | | 119 | | 115 | | 125 | |
| 50.51 | | 74 | | 99 | | 99 | | 91 | |
| 50.5304 | | 88 | | 116 | | 97 | | 97 | |
| 50.5509 | | 103 | | 119 | | 105 | | 102 | |
| 50.5713 | | 134 | | 128 | | 136 | | 126 | |
| 50.5918 | | 169 | | 159 | | 177 | | 150 | |
| 50.6122 | | 138 | | 170 | | 185 | | 147 | |
| 50.6327 | | 107 | | 152 | | 123 | | 119 | |
| 50.6531 | | 85 | | 113 | | 77 | | 114 | |
| 50.6736 | | 84 | | 88 | | 84 | | 86 | |
| 50.694 | | 87 | | 84 | | 107 | | 99 | |
| 50.7145 | | 92 | | 101 | | 97 | | 98 | |
| 50.7349 | | 96 | | 116 | | 111 | | 95 | |
| 50.7554 | | 90 | | 121 | | 110 | | 115 | |
| 50.7758 | | 67 | | 96 | | 101 | | 80 | |
| 50.7963 | | 66 | | 79 | | 76 | | 83 | |
| 50.8167 | | 57 | | 72 | | 82 | | 59 | |
| 50.8372 | | 65 | | 65 | | 67 | | 52 | |
| 50.8577 | | 54 | | 66 | | 55 | | 55 | |
| 50.8781 | | 76 | | 76 | | 65 | | 51 | |
| 50.8986 | | 65 | | 63 | | 60 | | 52 | |
| 50.919 | | 68 | | 66 | | 66 | | 76 | |
| 50.9395 | | 60 | | 67 | | 70 | | 62 | |
| 50.9599 | | 54 | | 71 | | 70 | | 60 | |
| 50.9804 | | 53 | | 59 | | 71 | | 49 | |
| 51.0008 | | 60 | | 61 | | 59 | | 60 | |
| 51.0213 | | 57 | | 61 | | 63 | | 62 | |
| 51.0417 | | 48 | | 61 | | 62 | | 61 | |
| 51.0622 | | 58 | | 58 | | 61 | | 61 | |
| 51.0826 | | 56 | | 58 | | 49 | | 65 | |
| 51.1031 | | 58 | | 53 | | 51 | | 65 | |
| 51.1235 | | 64 | | 51 | | 47 | | 64 | |
| 51.144 | | 55 | | 51 | | 57 | | 60 | |
| 51.1644 | | 55 | | 58 | | 56 | | 80 | |
| 51.1849 | | 50 | | 56 | | 48 | | 55 | |
| 51.2053 | | 64 | | 48 | | 41 | | 71 | |
| 51.2258 | | 53 | | 56 | | 64 | | 52 | |
| 51.2462 | | 60 | | 56 | | 42 | | 53 | |
| 51.2667 | | 66 | | 56 | | 49 | | 40 | |
| 51.2871 | | 41 | | 46 | | 60 | | 63 | |
| 51.3076 | | 33 | | 64 | | 63 | | 63 | |
| 51.328 | | 39 | | 67 | | 54 | | 49 | |
| 51.3485 | | 46 | | 56 | | 45 | | 65 | |
| 51.3689 | | 47 | | 55 | | 58 | | 51 | |
| 51.3894 | | 66 | | 56 | | 49 | | 51 | |
| 51.4099 | | 50 | | 43 | | 41 | | 50 | |
| 51.4303 | | 52 | | 52 | | 61 | | 46 | |
| 51.4508 | | 41 | | 48 | | 43 | | 44 | |
| 51.4712 | | 51 | | 61 | | 57 | | 37 | |
| 51.4917 | | 54 | | 64 | | 57 | | 44 | |
| 51.5121 | | 55 | | 50 | | 65 | | 40 | |
| 51.5326 | | 52 | | 48 | | 53 | | 60 | |
| 51.553 | | 47 | | 58 | | 57 | | 43 | |
| 51.5735 | | 41 | | 62 | | 55 | | 50 | |
| 51.5939 | | 55 | | 49 | | 63 | | 47 | |
| 51.6144 | | 51 | | 56 | | 44 | | 58 | |
| 51.6348 | | 40 | | 45 | | 39 | | 62 | |
| 51.6553 | | 51 | | 59 | | 55 | | 62 | |
| 51.6757 | | 42 | | 51 | | 62 | | 47 | |
| 51.6962 | | 49 | | 55 | | 56 | | 59 | |
| 51.7166 | | 51 | | 71 | | 59 | | 48 | |
| 51.7371 | | 51 | | 55 | | 51 | | 45 | |
| 51.7575 | | 51 | | 52 | | 56 | | 49 | |
| 51.778 | | 42 | | 51 | | 45 | | 54 | |
| 51.7984 | | 38 | | 65 | | 45 | | 59 | |
| 51.8189 | | 47 | | 42 | | 49 | | 55 | |
| 51.8393 | | 49 | | 57 | | 54 | | 49 | |
| 51.8598 | | 62 | | 45 | | 51 | | 59 | |
| 51.8802 | | 60 | | 55 | | 60 | | 50 | |
| 51.9007 | | 54 | | 60 | | 58 | | 66 | |
| 51.9212 | | 43 | | 67 | | 41 | | 50 | |
| 51.9416 | | 52 | | 52 | | 41 | | 51 | |
| 51.9621 | | 62 | | 59 | | 44 | | 48 | |
| 51.9825 | | 50 | | 57 | | 54 | | 52 | |
| 52.003 | | 51 | | 51 | | 42 | | 41 | |
| 52.0234 | | 49 | | 64 | | 56 | | 38 | |
| 52.0439 | | 57 | | 49 | | 52 | | 42 | |
| 52.0643 | | 46 | | 49 | | 53 | | 47 | |
| 52.0848 | | 38 | | 51 | | 62 | | 54 | |
| 52.1052 | | 49 | | 62 | | 53 | | 61 | |
| 52.1257 | | 40 | | 40 | | 49 | | 46 | |
| 52.1461 | | 40 | | 46 | | 56 | | 41 | |
| 52.1666 | | 49 | | 47 | | 49 | | 56 | |
| 52.187 | | 42 | | 56 | | 52 | | 46 | |
| 52.2075 | | 43 | | 43 | | 48 | | 51 | |
| 52.2279 | | 45 | | 57 | | 56 | | 52 | |
| 52.2484 | | 53 | | 62 | | 51 | | 51 | |
| 52.2688 | | 44 | | 57 | | 52 | | 56 | |
| 52.2893 | | 49 | | 50 | | 47 | | 42 | |
| 52.3097 | | 61 | | 52 | | 50 | | 52 | |
| 52.3302 | | 49 | | 49 | | 51 | | 54 | |
| 52.3506 | | 58 | | 52 | | 56 | | 42 | |
| 52.3711 | | 64 | | 66 | | 43 | | 60 | |
| 52.3915 | | 47 | | 64 | | 64 | | 48 | |
| 52.412 | | 52 | | 56 | | 51 | | 54 | |
| 52.4324 | | 55 | | 38 | | 48 | | 44 | |
| 52.4529 | | 48 | | 55 | | 57 | | 53 | |
| 52.4734 | | 40 | | 73 | | 51 | | 55 | |
| 52.4938 | | 46 | | 62 | | 49 | | 53 | |
| 52.5143 | | 43 | | 41 | | 39 | | 39 | |
| 52.5347 | | 41 | | 52 | | 48 | | 42 | |
| 52.5552 | | 50 | | 54 | | 58 | | 52 | |
| 52.5756 | | 51 | | 57 | | 56 | | 52 | |
| 52.5961 | | 41 | | 52 | | 48 | | 46 | |
| 52.6165 | | 54 | | 34 | | 58 | | 55 | |
| 52.637 | | 51 | | 62 | | 59 | | 56 | |
| 52.6574 | | 43 | | 46 | | 55 | | 49 | |
| 52.6779 | | 43 | | 43 | | 57 | | 47 | |
| 52.6983 | | 29 | | 42 | | 50 | | 44 | |
| 52.7188 | | 36 | | 53 | | 46 | | 43 | |
| 52.7392 | | 47 | | 52 | | 37 | | 39 | |
| 52.7597 | | 36 | | 48 | | 44 | | 48 | |
| 52.7801 | | 56 | | 41 | | 36 | | 52 | |
| 52.8006 | | 50 | | 48 | | 52 | | 45 | |
| 52.821 | | 52 | | 50 | | 45 | | 51 | |
| 52.8415 | | 51 | | 55 | | 40 | | 52 | |
| 52.8619 | | 45 | | 60 | | 64 | | 58 | |
| 52.8824 | | 56 | | 58 | | 48 | | 41 | |
| 52.9028 | | 49 | | 60 | | 50 | | 62 | |
| 52.9233 | | 49 | | 42 | | 55 | | 46 | |
| 52.9437 | | 51 | | 62 | | 59 | | 47 | |
| 52.9642 | | 55 | | 42 | | 53 | | 48 | |
| 52.9847 | | 36 | | 56 | | 41 | | 45 | |
| 53.0051 | | 54 | | 66 | | 53 | | 48 | |
| 53.0256 | | 49 | | 36 | | 60 | | 38 | |
| 53.046 | | 47 | | 62 | | 62 | | 53 | |
| 53.0665 | | 39 | | 39 | | 40 | | 60 | |
| 53.0869 | | 55 | | 49 | | 52 | | 46 | |
| 53.1074 | | 43 | | 57 | | 49 | | 56 | |
| 53.1278 | | 48 | | 62 | | 45 | | 52 | |
| 53.1483 | | 50 | | 55 | | 36 | | 50 | |
| 53.1687 | | 48 | | 55 | | 57 | | 40 | |
| 53.1892 | | 52 | | 61 | | 62 | | 53 | |
| 53.2096 | | 44 | | 56 | | 53 | | 52 | |
| 53.2301 | | 56 | | 60 | | 65 | | 53 | |
| 53.2505 | | 55 | | 54 | | 47 | | 43 | |
| 53.271 | | 53 | | 52 | | 56 | | 46 | |
| 53.2914 | | 41 | | 49 | | 39 | | 52 | |
| 53.3119 | | 43 | | 65 | | 45 | | 49 | |
| 53.3323 | | 45 | | 47 | | 47 | | 52 | |
| 53.3528 | | 48 | | 53 | | 59 | | 59 | |
| 53.3732 | | 56 | | 59 | | 60 | | 52 | |
| 53.3937 | | 40 | | 45 | | 41 | | 61 | |
| 53.4141 | | 54 | | 58 | | 49 | | 58 | |
| 53.4346 | | 59 | | 40 | | 46 | | 45 | |
| 53.455 | | 50 | | 56 | | 59 | | 45 | |
| 53.4755 | | 46 | | 53 | | 51 | | 64 | |
| 53.496 | | 51 | | 44 | | 45 | | 52 | |
| 53.5164 | | 42 | | 49 | | 50 | | 61 | |
| 53.5369 | | 67 | | 59 | | 53 | | 50 | |
| 53.5573 | | 64 | | 61 | | 58 | | 63 | |
| 53.5778 | | 56 | | 51 | | 69 | | 58 | |
| 53.5982 | | 77 | | 64 | | 66 | | 81 | |
| 53.6187 | | 72 | | 88 | | 73 | | 97 | |
| 53.6391 | | 107 | | 95 | | 95 | | 99 | |
| 53.6596 | | 126 | | 118 | | 117 | | 116 | |
| 53.68 | | 136 | | 134 | | 131 | | 117 | |
| 53.7005 | | 91 | | 100 | | 83 | | 94 | |
| 53.7209 | | 80 | | 63 | | 72 | | 79 | |
| 53.7414 | | 69 | | 69 | | 82 | | 56 | |
| 53.7618 | | 68 | | 60 | | 65 | | 60 | |
| 53.7823 | | 53 | | 64 | | 61 | | 44 | |
| 53.8027 | | 55 | | 55 | | 42 | | 47 | |
| 53.8232 | | 46 | | 59 | | 49 | | 56 | |
| 53.8436 | | 47 | | 60 | | 49 | | 54 | |
| 53.8641 | | 44 | | 51 | | 53 | | 48 | |
| 53.8845 | | 52 | | 41 | | 43 | | 47 | |
| 53.905 | | 54 | | 60 | | 62 | | 58 | |
| 53.9254 | | 52 | | 59 | | 65 | | 50 | |
| 53.9459 | | 45 | | 57 | | 52 | | 52 | |
| 53.9663 | | 34 | | 56 | | 61 | | 59 | |
| 53.9868 | | 60 | | 52 | | 46 | | 41 | |
| 54.0072 | | 56 | | 56 | | 50 | | 46 | |
| 54.0277 | | 56 | | 47 | | 48 | | 53 | |
| 54.0482 | | 43 | | 51 | | 71 | | 47 | |
| 54.0686 | | 51 | | 54 | | 43 | | 53 | |
| 54.0891 | | 39 | | 64 | | 59 | | 59 | |
| 54.1095 | | 50 | | 56 | | 37 | | 38 | |
| 54.13 | | 54 | | 62 | | 35 | | 46 | |
| 54.1504 | | 50 | | 51 | | 51 | | 53 | |
| 54.1709 | | 48 | | 49 | | 42 | | 41 | |
| 54.1913 | | 46 | | 46 | | 52 | | 51 | |
| 54.2118 | | 50 | | 55 | | 52 | | 57 | |
| 54.2322 | | 52 | | 62 | | 39 | | 41 | |
| 54.2527 | | 50 | | 51 | | 61 | | 41 | |
| 54.2731 | | 52 | | 48 | | 63 | | 61 | |
| 54.2936 | | 66 | | 56 | | 52 | | 49 | |
| 54.314 | | 61 | | 60 | | 51 | | 44 | |
| 54.3345 | | 40 | | 56 | | 45 | | 68 | |
| 54.3549 | | 55 | | 52 | | 55 | | 53 | |
| 54.3754 | | 45 | | 57 | | 54 | | 52 | |
| 54.3958 | | 52 | | 57 | | 47 | | 56 | |
| 54.4163 | | 70 | | 45 | | 47 | | 62 | |
| 54.4367 | | 51 | | 53 | | 60 | | 44 | |
| 54.4572 | | 56 | | 62 | | 54 | | 60 | |
| 54.4776 | | 53 | | 59 | | 60 | | 58 | |
| 54.4981 | | 59 | | 76 | | 64 | | 62 | |
| 54.5185 | | 73 | | 64 | | 65 | | 58 | |
| 54.539 | | 60 | | 73 | | 53 | | 70 | |
| 54.5595 | | 64 | | 66 | | 66 | | 65 | |
| 54.5799 | | 48 | | 66 | | 69 | | 79 | |
| 54.6004 | | 85 | | 76 | | 66 | | 88 | |
| 54.6208 | | 78 | | 70 | | 86 | | 67 | |
| 54.6413 | | 70 | | 77 | | 82 | | 103 | |
| 54.6617 | | 79 | | 98 | | 78 | | 91 | |
| 54.6822 | | 84 | | 113 | | 73 | | 92 | |
| 54.7026 | | 100 | | 100 | | 103 | | 90 | |
| 54.7231 | | 110 | | 127 | | 117 | | 130 | |
| 54.7435 | | 147 | | 172 | | 157 | | 153 | |
| 54.764 | | 192 | | 221 | | 193 | | 210 | |
| 54.7844 | | 285 | | 296 | | 288 | | 328 | |
| 54.8049 | | 457 | | 464 | | 465 | | 473 | |
| 54.8253 | | 871 | | 760 | | 859 | | 893 | |
| 54.8458 | | 1519 | | 1344 | | 1399 | | 1338 | |
| 54.8662 | | 1655 | | 1812 | | 1797 | | 1447 | |
| 54.8867 | | 825 | | 1164 | | 1102 | | 918 | |
| 54.9071 | | 393 | | 524 | | 471 | | 511 | |
| 54.9276 | | 264 | | 375 | | 340 | | 337 | |
| 54.948 | | 326 | | 286 | | 358 | | 384 | |
| 54.9685 | | 477 | | 448 | | 415 | | 495 | |
| 54.9889 | | 770 | | 669 | | 640 | | 695 | |
| 55.0094 | | 882 | | 967 | | 947 | | 746 | |
| 55.0298 | | 534 | | 738 | | 726 | | 615 | |
| 55.0503 | | 291 | | 364 | | 359 | | 295 | |
| 55.0708 | | 171 | | 204 | | 188 | | 170 | |
| 55.0912 | | 115 | | 147 | | 128 | | 126 | |
| 55.1117 | | 120 | | 120 | | 111 | | 120 | |
| 55.1321 | | 84 | | 121 | | 103 | | 115 | |
| 55.1526 | | 88 | | 110 | | 105 | | 88 | |
| 55.173 | | 102 | | 111 | | 111 | | 110 | |
| 55.1935 | | 108 | | 115 | | 117 | | 109 | |
| 55.2139 | | 119 | | 113 | | 124 | | 112 | |
| 55.2344 | | 164 | | 153 | | 145 | | 165 | |
| 55.2548 | | 217 | | 212 | | 203 | | 208 | |
| 55.2753 | | 326 | | 302 | | 325 | | 319 | |
| 55.2957 | | 508 | | 458 | | 533 | | 506 | |
| 55.3162 | | 515 | | 610 | | 563 | | 498 | |
| 55.3366 | | 304 | | 419 | | 370 | | 366 | |
| 55.3571 | | 175 | | 220 | | 193 | | 205 | |
| 55.3775 | | 154 | | 199 | | 173 | | 170 | |
| 55.398 | | 136 | | 164 | | 160 | | 167 | |
| 55.4184 | | 194 | | 196 | | 219 | | 210 | |
| 55.4389 | | 286 | | 280 | | 264 | | 269 | |
| 55.4593 | | 291 | | 336 | | 313 | | 318 | |
| 55.4798 | | 234 | | 258 | | 242 | | 240 | |
| 55.5002 | | 124 | | 151 | | 143 | | 178 | |
| 55.5207 | | 95 | | 102 | | 92 | | 105 | |
| 55.5411 | | 72 | | 90 | | 100 | | 63 | |
| 55.5616 | | 77 | | 71 | | 82 | | 78 | |
| 55.582 | | 69 | | 60 | | 72 | | 75 | |
| 55.6025 | | 57 | | 71 | | 64 | | 60 | |
| 55.623 | | 59 | | 71 | | 64 | | 50 | |
| 55.6434 | | 55 | | 77 | | 58 | | 66 | |
| 55.6639 | | 58 | | 64 | | 48 | | 65 | |
| 55.6843 | | 61 | | 71 | | 48 | | 51 | |
| 55.7048 | | 55 | | 49 | | 57 | | 57 | |
| 55.7252 | | 46 | | 51 | | 49 | | 64 | |
| 55.7457 | | 44 | | 57 | | 52 | | 50 | |
| 55.7661 | | 62 | | 58 | | 62 | | 54 | |
| 55.7866 | | 48 | | 51 | | 63 | | 37 | |
| 55.807 | | 54 | | 77 | | 44 | | 56 | |
| 55.8275 | | 48 | | 43 | | 45 | | 49 | |
| 55.8479 | | 52 | | 70 | | 49 | | 54 | |
| 55.8684 | | 51 | | 64 | | 45 | | 58 | |
| 55.8888 | | 67 | | 53 | | 57 | | 55 | |
| 55.9093 | | 46 | | 59 | | 67 | | 55 | |
| 55.9297 | | 54 | | 43 | | 56 | | 42 | |
| 55.9502 | | 52 | | 50 | | 47 | | 55 | |
| 55.9706 | | 56 | | 59 | | 57 | | 51 | |
| 55.9911 | | 52 | | 53 | | 52 | | 64 | |
| 56.0115 | | 47 | | 61 | | 56 | | 46 | |
| 56.032 | | 48 | | 50 | | 51 | | 54 | |
| 56.0524 | | 57 | | 50 | | 48 | | 43 | |
| 56.0729 | | 56 | | 53 | | 45 | | 51 | |
| 56.0933 | | 48 | | 56 | | 52 | | 39 | |
| 56.1138 | | 60 | | 51 | | 54 | | 52 | |
| 56.1343 | | 38 | | 50 | | 55 | | 53 | |
| 56.1547 | | 46 | | 58 | | 69 | | 50 | |
| 56.1752 | | 49 | | 45 | | 49 | | 35 | |
| 56.1956 | | 46 | | 56 | | 61 | | 47 | |
| 56.2161 | | 57 | | 69 | | 61 | | 49 | |
| 56.2365 | | 50 | | 43 | | 53 | | 54 | |
| 56.257 | | 50 | | 59 | | 64 | | 52 | |
| 56.2774 | | 53 | | 50 | | 37 | | 55 | |
| 56.2979 | | 47 | | 42 | | 49 | | 49 | |
| 56.3183 | | 42 | | 62 | | 51 | | 50 | |
| 56.3388 | | 45 | | 50 | | 53 | | 46 | |
| 56.3592 | | 46 | | 51 | | 49 | | 58 | |
| 56.3797 | | 44 | | 56 | | 54 | | 57 | |
| 56.4001 | | 48 | | 54 | | 52 | | 42 | |
| 56.4206 | | 46 | | 49 | | 55 | | 40 | |
| 56.441 | | 50 | | 65 | | 50 | | 43 | |
| 56.4615 | | 57 | | 38 | | 56 | | 42 | |
| 56.4819 | | 54 | | 45 | | 43 | | 41 | |
| 56.5024 | | 50 | | 53 | | 46 | | 44 | |
| 56.5228 | | 49 | | 57 | | 48 | | 57 | |
| 56.5433 | | 43 | | 43 | | 36 | | 50 | |
| 56.5637 | | 44 | | 52 | | 46 | | 39 | |
| 56.5842 | | 43 | | 55 | | 51 | | 57 | |
| 56.6046 | | 53 | | 62 | | 57 | | 35 | |
| 56.6251 | | 55 | | 61 | | 54 | | 42 | |
| 56.6456 | | 46 | | 43 | | 50 | | 43 | |
| 56.666 | | 38 | | 48 | | 50 | | 46 | |
| 56.6865 | | 41 | | 45 | | 43 | | 37 | |
| 56.7069 | | 42 | | 57 | | 49 | | 42 | |
| 56.7274 | | 43 | | 49 | | 49 | | 43 | |
| 56.7478 | | 48 | | 47 | | 44 | | 45 | |
| 56.7683 | | 33 | | 46 | | 48 | | 51 | |
| 56.7887 | | 52 | | 54 | | 39 | | 47 | |
| 56.8092 | | 49 | | 52 | | 58 | | 56 | |
| 56.8296 | | 52 | | 58 | | 54 | | 46 | |
| 56.8501 | | 41 | | 47 | | 50 | | 51 | |
| 56.8705 | | 46 | | 60 | | 50 | | 43 | |
| 56.891 | | 39 | | 44 | | 37 | | 62 | |
| 56.9114 | | 42 | | 51 | | 42 | | 62 | |
| 56.9319 | | 51 | | 52 | | 50 | | 61 | |
| 56.9523 | | 56 | | 36 | | 41 | | 62 | |
| 56.9728 | | 62 | | 47 | | 50 | | 52 | |
| 56.9932 | | 46 | | 44 | | 52 | | 56 | |
| 57.0137 | | 56 | | 62 | | 53 | | 58 | |
| 57.0341 | | 52 | | 61 | | 53 | | 56 | |
| 57.0546 | | 48 | | 44 | | 47 | | 57 | |
| 57.075 | | 49 | | 67 | | 47 | | 53 | |
| 57.0955 | | 43 | | 61 | | 53 | | 58 | |
| 57.1159 | | 57 | | 63 | | 63 | | 59 | |
| 57.1364 | | 58 | | 69 | | 69 | | 64 | |
| 57.1568 | | 80 | | 90 | | 80 | | 84 | |
| 57.1773 | | 101 | | 92 | | 82 | | 98 | |
| 57.1978 | | 142 | | 120 | | 125 | | 130 | |
| 57.2182 | | 148 | | 165 | | 165 | | 155 | |
| 57.2387 | | 121 | | 147 | | 130 | | 131 | |
| 57.2591 | | 91 | | 107 | | 103 | | 82 | |
| 57.2796 | | 84 | | 80 | | 71 | | 76 | |
| 57.3 | | 60 | | 87 | | 61 | | 72 | |
| 57.3205 | | 55 | | 76 | | 62 | | 64 | |
| 57.3409 | | 86 | | 53 | | 72 | | 75 | |
| 57.3614 | | 79 | | 89 | | 87 | | 84 | |
| 57.3818 | | 88 | | 106 | | 95 | | 87 | |
| 57.4023 | | 59 | | 68 | | 70 | | 81 | |
| 57.4227 | | 54 | | 58 | | 48 | | 58 | |
| 57.4432 | | 60 | | 76 | | 47 | | 54 | |
| 57.4636 | | 53 | | 55 | | 53 | | 63 | |
| 57.4841 | | 44 | | 68 | | 54 | | 50 | |
| 57.5045 | | 33 | | 42 | | 41 | | 45 | |
| 57.525 | | 64 | | 48 | | 41 | | 54 | |
| 57.5454 | | 50 | | 57 | | 47 | | 65 | |
| 57.5659 | | 59 | | 50 | | 60 | | 34 | |
| 57.5863 | | 48 | | 49 | | 56 | | 51 | |
| 57.6068 | | 54 | | 52 | | 53 | | 54 | |
| 57.6272 | | 54 | | 55 | | 60 | | 49 | |
| 57.6477 | | 52 | | 54 | | 44 | | 32 | |
| 57.6681 | | 55 | | 51 | | 60 | | 63 | |
| 57.6886 | | 46 | | 46 | | 53 | | 71 | |
| 57.7091 | | 49 | | 49 | | 51 | | 55 | |
| 57.7295 | | 36 | | 55 | | 52 | | 43 | |
| 57.75 | | 41 | | 57 | | 52 | | 43 | |
| 57.7704 | | 57 | | 38 | | 44 | | 43 | |
| 57.7909 | | 65 | | 50 | | 58 | | 49 | |
| 57.8113 | | 47 | | 57 | | 60 | | 37 | |
| 57.8318 | | 55 | | 53 | | 43 | | 45 | |
| 57.8522 | | 37 | | 58 | | 44 | | 50 | |
| 57.8727 | | 54 | | 41 | | 48 | | 46 | |
| 57.8931 | | 48 | | 49 | | 50 | | 58 | |
| 57.9136 | | 45 | | 49 | | 61 | | 48 | |
| 57.934 | | 53 | | 57 | | 43 | | 45 | |
| 57.9545 | | 52 | | 63 | | 54 | | 37 | |
| 57.9749 | | 45 | | 49 | | 37 | | 53 | |
| 57.9954 | | 52 | | 59 | | 48 | | 44 | |
| 58.0158 | | 44 | | 44 | | 41 | | 51 | |
| 58.0363 | | 45 | | 55 | | 49 | | 52 | |
| 58.0567 | | 53 | | 49 | | 51 | | 55 | |
| 58.0772 | | 48 | | 48 | | 46 | | 46 | |
| 58.0976 | | 60 | | 42 | | 42 | | 48 | |
| 58.1181 | | 55 | | 42 | | 50 | | 51 | |
| 58.1385 | | 47 | | 48 | | 54 | | 52 | |
| 58.159 | | 47 | | 59 | | 57 | | 57 | |
| 58.1794 | | 60 | | 50 | | 48 | | 47 | |
| 58.1999 | | 49 | | 47 | | 42 | | 55 | |
| 58.2204 | | 46 | | 42 | | 41 | | 52 | |
| 58.2408 | | 45 | | 55 | | 41 | | 47 | |
| 58.2613 | | 43 | | 46 | | 44 | | 47 | |
| 58.2817 | | 43 | | 42 | | 41 | | 54 | |
| 58.3022 | | 41 | | 39 | | 46 | | 69 | |
| 58.3226 | | 48 | | 57 | | 57 | | 45 | |
| 58.3431 | | 33 | | 59 | | 40 | | 55 | |
| 58.3635 | | 41 | | 53 | | 40 | | 47 | |
| 58.384 | | 55 | | 62 | | 52 | | 51 | |
| 58.4044 | | 39 | | 36 | | 61 | | 58 | |
| 58.4249 | | 60 | | 40 | | 41 | | 39 | |
| 58.4453 | | 55 | | 61 | | 47 | | 42 | |
| 58.4658 | | 47 | | 57 | | 56 | | 56 | |
| 58.4862 | | 50 | | 56 | | 45 | | 43 | |
| 58.5067 | | 51 | | 57 | | 52 | | 51 | |
| 58.5271 | | 55 | | 48 | | 44 | | 49 | |
| 58.5476 | | 53 | | 52 | | 50 | | 52 | |
| 58.568 | | 42 | | 45 | | 38 | | 46 | |
| 58.5885 | | 50 | | 50 | | 53 | | 51 | |
| 58.6089 | | 45 | | 55 | | 44 | | 41 | |
| 58.6294 | | 57 | | 52 | | 55 | | 60 | |
| 58.6498 | | 58 | | 79 | | 57 | | 60 | |
| 58.6703 | | 43 | | 49 | | 49 | | 53 | |
| 58.6907 | | 45 | | 60 | | 61 | | 59 | |
| 58.7112 | | 57 | | 41 | | 55 | | 52 | |
| 58.7316 | | 51 | | 69 | | 55 | | 62 | |
| 58.7521 | | 59 | | 53 | | 55 | | 62 | |
| 58.7726 | | 54 | | 44 | | 57 | | 46 | |
| 58.793 | | 55 | | 59 | | 55 | | 41 | |
| 58.8135 | | 62 | | 60 | | 72 | | 52 | |
| 58.8339 | | 40 | | 58 | | 74 | | 63 | |
| 58.8544 | | 56 | | 51 | | 62 | | 41 | |
| 58.8748 | | 39 | | 50 | | 56 | | 55 | |
| 58.8953 | | 49 | | 69 | | 40 | | 48 | |
| 58.9157 | | 51 | | 57 | | 51 | | 47 | |
| 58.9362 | | 41 | | 51 | | 43 | | 52 | |
| 58.9566 | | 41 | | 42 | | 50 | | 52 | |
| 58.9771 | | 42 | | 54 | | 62 | | 56 | |
| 58.9975 | | 42 | | 55 | | 55 | | 43 | |
| 59.018 | | 47 | | 46 | | 67 | | 70 | |
| 59.0384 | | 62 | | 57 | | 47 | | 53 | |
| 59.0589 | | 58 | | 51 | | 49 | | 50 | |
| 59.0793 | | 51 | | 48 | | 61 | | 49 | |
| 59.0998 | | 43 | | 48 | | 57 | | 56 | |
| 59.1202 | | 49 | | 47 | | 56 | | 53 | |
| 59.1407 | | 45 | | 52 | | 59 | | 49 | |
| 59.1611 | | 44 | | 60 | | 44 | | 56 | |
| 59.1816 | | 52 | | 57 | | 66 | | 61 | |
| 59.202 | | 39 | | 66 | | 47 | | 51 | |
| 59.2225 | | 51 | | 62 | | 35 | | 57 | |
| 59.2429 | | 57 | | 67 | | 56 | | 52 | |
| 59.2634 | | 46 | | 57 | | 64 | | 55 | |
| 59.2839 | | 43 | | 46 | | 51 | | 60 | |
| 59.3043 | | 58 | | 55 | | 50 | | 43 | |
| 59.3248 | | 63 | | 62 | | 68 | | 42 | |
| 59.3452 | | 56 | | 58 | | 66 | | 53 | |
| 59.3657 | | 52 | | 54 | | 72 | | 57 | |
| 59.3861 | | 53 | | 76 | | 66 | | 58 | |
| 59.4066 | | 73 | | 70 | | 63 | | 62 | |
| 59.427 | | 57 | | 68 | | 79 | | 54 | |
| 59.4475 | | 50 | | 55 | | 71 | | 62 | |
| 59.4679 | | 42 | | 71 | | 69 | | 72 | |
| 59.4884 | | 68 | | 75 | | 72 | | 77 | |
| 59.5088 | | 74 | | 88 | | 76 | | 66 | |
| 59.5293 | | 78 | | 67 | | 62 | | 71 | |
| 59.5497 | | 75 | | 72 | | 77 | | 92 | |
| 59.5702 | | 78 | | 82 | | 81 | | 83 | |
| 59.5906 | | 79 | | 94 | | 80 | | 79 | |
| 59.6111 | | 89 | | 98 | | 71 | | 100 | |
| 59.6315 | | 80 | | 111 | | 118 | | 116 | |
| 59.652 | | 81 | | 97 | | 114 | | 102 | |
| 59.6724 | | 105 | | 93 | | 133 | | 124 | |
| 59.6929 | | 110 | | 111 | | 128 | | 134 | |
| 59.7133 | | 139 | | 145 | | 116 | | 138 | |
| 59.7338 | | 109 | | 144 | | 129 | | 143 | |
| 59.7542 | | 129 | | 142 | | 139 | | 153 | |
| 59.7747 | | 163 | | 200 | | 176 | | 197 | |
| 59.7951 | | 192 | | 198 | | 203 | | 233 | |
| 59.8156 | | 270 | | 301 | | 262 | | 296 | |
| 59.8361 | | 308 | | 352 | | 344 | | 403 | |
| 59.8565 | | 458 | | 528 | | 504 | | 542 | |
| 59.877 | | 768 | | 778 | | 721 | | 816 | |
| 59.8974 | | 1499 | | 1168 | | 1222 | | 1449 | |
| 59.9179 | | 2560 | | 2009 | | 2198 | | 2309 | |
| 59.9383 | | 3562 | | 3311 | | 3486 | | 3313 | |
| 59.9588 | | 2779 | | 3481 | | 3131 | | 2881 | |
| 59.9792 | | 1265 | | 1794 | | 1569 | | 1688 | |
| 59.9997 | | 637 | | 955 | | 816 | | 999 | |
| 60.0201 | | 523 | | 644 | | 600 | | 691 | |
| 60.0406 | | 578 | | 591 | | 556 | | 695 | |
| 60.061 | | 847 | | 816 | | 747 | | 844 | |
| 60.0815 | | 1339 | | 1190 | | 1237 | | 1309 | |
| 60.1019 | | 1810 | | 1601 | | 1662 | | 1703 | |
| 60.1224 | | 1508 | | 1777 | | 1600 | | 1586 | |
| 60.1428 | | 709 | | 1013 | | 919 | | 922 | |
| 60.1633 | | 396 | | 503 | | 394 | | 533 | |
| 60.1837 | | 236 | | 307 | | 264 | | 326 | |
| 60.2042 | | 212 | | 213 | | 199 | | 250 | |
| 60.2246 | | 133 | | 184 | | 162 | | 182 | |
| 60.2451 | | 118 | | 147 | | 159 | | 143 | |
| 60.2655 | | 123 | | 132 | | 131 | | 135 | |
| 60.286 | | 114 | | 111 | | 127 | | 107 | |
| 60.3064 | | 100 | | 106 | | 104 | | 108 | |
| 60.3269 | | 79 | | 86 | | 118 | | 103 | |
| 60.3474 | | 86 | | 100 | | 101 | | 100 | |
| 60.3678 | | 77 | | 103 | | 106 | | 89 | |
| 60.3883 | | 75 | | 82 | | 88 | | 84 | |
| 60.4087 | | 89 | | 117 | | 95 | | 101 | |
| 60.4292 | | 127 | | 97 | | 112 | | 101 | |
| 60.4496 | | 127 | | 127 | | 113 | | 108 | |
| 60.4701 | | 106 | | 129 | | 136 | | 105 | |
| 60.4905 | | 94 | | 138 | | 116 | | 105 | |
| 60.511 | | 94 | | 87 | | 91 | | 83 | |
| 60.5314 | | 57 | | 73 | | 62 | | 85 | |
| 60.5519 | | 80 | | 74 | | 77 | | 78 | |
| 60.5723 | | 60 | | 78 | | 68 | | 69 | |
| 60.5928 | | 65 | | 63 | | 67 | | 52 | |
| 60.6132 | | 59 | | 59 | | 54 | | 76 | |
| 60.6337 | | 78 | | 73 | | 54 | | 64 | |
| 60.6541 | | 67 | | 79 | | 55 | | 48 | |
| 60.6746 | | 73 | | 71 | | 69 | | 71 | |
| 60.695 | | 59 | | 60 | | 65 | | 74 | |
| 60.7155 | | 64 | | 71 | | 63 | | 61 | |
| 60.7359 | | 72 | | 83 | | 90 | | 84 | |
| 60.7564 | | 81 | | 83 | | 81 | | 84 | |
| 60.7768 | | 97 | | 108 | | 111 | | 94 | |
| 60.7973 | | 108 | | 122 | | 105 | | 101 | |
| 60.8177 | | 105 | | 131 | | 105 | | 108 | |
| 60.8382 | | 94 | | 113 | | 104 | | 90 | |
| 60.8587 | | 81 | | 91 | | 83 | | 74 | |
| 60.8791 | | 64 | | 83 | | 59 | | 76 | |
| 60.8996 | | 70 | | 83 | | 92 | | 82 | |
| 60.92 | | 87 | | 73 | | 102 | | 84 | |
| 60.9405 | | 87 | | 95 | | 92 | | 84 | |
| 60.9609 | | 80 | | 80 | | 113 | | 83 | |
| 60.9814 | | 81 | | 81 | | 90 | | 89 | |
| 61.0018 | | 75 | | 78 | | 69 | | 64 | |
| 61.0223 | | 57 | | 73 | | 77 | | 71 | |
| 61.0427 | | 71 | | 62 | | 70 | | 55 | |
| 61.0632 | | 60 | | 49 | | 57 | | 59 | |
| 61.0836 | | 58 | | 61 | | 72 | | 67 | |
| 61.1041 | | 42 | | 62 | | 66 | | 53 | |
| 61.1245 | | 57 | | 64 | | 49 | | 59 | |
| 61.145 | | 54 | | 60 | | 53 | | 68 | |
| 61.1654 | | 57 | | 55 | | 54 | | 49 | |
| 61.1859 | | 61 | | 64 | | 71 | | 57 | |
| 61.2063 | | 57 | | 52 | | 58 | | 62 | |
| 61.2268 | | 66 | | 63 | | 51 | | 53 | |
| 61.2472 | | 56 | | 58 | | 60 | | 60 | |
| 61.2677 | | 41 | | 58 | | 63 | | 50 | |
| 61.2881 | | 54 | | 70 | | 62 | | 54 | |
| 61.3086 | | 50 | | 54 | | 55 | | 61 | |
| 61.329 | | 54 | | 57 | | 58 | | 59 | |
| 61.3495 | | 55 | | 51 | | 52 | | 59 | |
| 61.3699 | | 64 | | 45 | | 48 | | 60 | |
| 61.3904 | | 46 | | 43 | | 39 | | 57 | |
| 61.4109 | | 47 | | 53 | | 53 | | 54 | |
| 61.4313 | | 50 | | 57 | | 51 | | 48 | |
| 61.4518 | | 48 | | 35 | | 42 | | 42 | |
| 61.4722 | | 54 | | 46 | | 57 | | 59 | |
| 61.4927 | | 51 | | 71 | | 56 | | 48 | |
| 61.5131 | | 36 | | 54 | | 47 | | 50 | |
| 61.5336 | | 59 | | 59 | | 57 | | 44 | |
| 61.554 | | 42 | | 50 | | 52 | | 61 | |
| 61.5745 | | 44 | | 55 | | 47 | | 51 | |
| 61.5949 | | 52 | | 50 | | 37 | | 48 | |
| 61.6154 | | 44 | | 49 | | 50 | | 56 | |
| 61.6358 | | 45 | | 60 | | 51 | | 50 | |
| 61.6563 | | 57 | | 52 | | 45 | | 60 | |
| 61.6767 | | 57 | | 53 | | 48 | | 60 | |
| 61.6972 | | 49 | | 49 | | 47 | | 52 | |
| 61.7176 | | 53 | | 46 | | 50 | | 50 | |
| 61.7381 | | 42 | | 60 | | 53 | | 42 | |
| 61.7585 | | 45 | | 47 | | 51 | | 45 | |
| 61.779 | | 45 | | 37 | | 58 | | 50 | |
| 61.7994 | | 42 | | 52 | | 38 | | 53 | |
| 61.8199 | | 53 | | 45 | | 63 | | 46 | |
| 61.8403 | | 46 | | 60 | | 46 | | 51 | |
| 61.8608 | | 44 | | 55 | | 54 | | 36 | |
| 61.8812 | | 52 | | 48 | | 54 | | 56 | |
| 61.9017 | | 43 | | 41 | | 57 | | 46 | |
| 61.9222 | | 56 | | 59 | | 42 | | 56 | |
| 61.9426 | | 48 | | 43 | | 48 | | 61 | |
| 61.9631 | | 41 | | 46 | | 46 | | 42 | |
| 61.9835 | | 40 | | 46 | | 44 | | 52 | |
| 62.004 | | 52 | | 44 | | 56 | | 48 | |
| 62.0244 | | 45 | | 53 | | 50 | | 55 | |
| 62.0449 | | 55 | | 41 | | 50 | | 45 | |
| 62.0653 | | 55 | | 74 | | 46 | | 67 | |
| 62.0858 | | 66 | | 48 | | 43 | | 51 | |
| 62.1062 | | 43 | | 55 | | 53 | | 53 | |
| 62.1267 | | 61 | | 59 | | 52 | | 38 | |
| 62.1471 | | 42 | | 49 | | 56 | | 51 | |
| 62.1676 | | 46 | | 42 | | 46 | | 40 | |
| 62.188 | | 60 | | 44 | | 54 | | 63 | |
| 62.2085 | | 49 | | 61 | | 43 | | 63 | |
| 62.2289 | | 55 | | 54 | | 59 | | 44 | |
| 62.2494 | | 59 | | 47 | | 53 | | 35 | |
| 62.2698 | | 49 | | 47 | | 38 | | 50 | |
| 62.2903 | | 41 | | 58 | | 42 | | 47 | |
| 62.3107 | | 60 | | 48 | | 47 | | 39 | |
| 62.3312 | | 58 | | 47 | | 49 | | 58 | |
| 62.3516 | | 61 | | 61 | | 47 | | 44 | |
| 62.3721 | | 55 | | 56 | | 48 | | 69 | |
| 62.3925 | | 51 | | 54 | | 55 | | 48 | |
| 62.413 | | 40 | | 56 | | 41 | | 43 | |
| 62.4335 | | 40 | | 59 | | 48 | | 61 | |
| 62.4539 | | 52 | | 50 | | 59 | | 55 | |
| 62.4744 | | 48 | | 49 | | 54 | | 50 | |
| 62.4948 | | 48 | | 52 | | 57 | | 39 | |
| 62.5153 | | 52 | | 53 | | 63 | | 49 | |
| 62.5357 | | 46 | | 45 | | 47 | | 59 | |
| 62.5562 | | 51 | | 66 | | 41 | | 52 | |
| 62.5766 | | 46 | | 50 | | 49 | | 56 | |
| 62.5971 | | 60 | | 48 | | 51 | | 61 | |
| 62.6175 | | 46 | | 46 | | 33 | | 42 | |
| 62.638 | | 80 | | 59 | | 53 | | 49 | |
| 62.6584 | | 49 | | 67 | | 57 | | 52 | |
| 62.6789 | | 66 | | 59 | | 58 | | 59 | |
| 62.6993 | | 47 | | 65 | | 52 | | 40 | |
| 62.7198 | | 42 | | 44 | | 40 | | 41 | |
| 62.7402 | | 41 | | 54 | | 50 | | 55 | |
| 62.7607 | | 48 | | 51 | | 38 | | 47 | |
| 62.7811 | | 51 | | 52 | | 60 | | 48 | |
| 62.8016 | | 52 | | 59 | | 53 | | 48 | |
| 62.822 | | 60 | | 50 | | 37 | | 53 | |
| 62.8425 | | 49 | | 65 | | 55 | | 47 | |
| 62.8629 | | 34 | | 46 | | 49 | | 52 | |
| 62.8834 | | 35 | | 50 | | 49 | | 60 | |
| 62.9038 | | 52 | | 38 | | 62 | | 51 | |
| 62.9243 | | 63 | | 48 | | 49 | | 29 | |
| 62.9447 | | 52 | | 44 | | 50 | | 42 | |
| 62.9652 | | 57 | | 54 | | 40 | | 42 | |
| 62.9857 | | 60 | | 43 | | 49 | | 55 | |
| 63.0061 | | 49 | | 52 | | 46 | | 39 | |
| 63.0266 | | 51 | | 46 | | 60 | | 58 | |
| 63.047 | | 47 | | 47 | | 50 | | 59 | |
| 63.0675 | | 58 | | 58 | | 43 | | 50 | |
| 63.0879 | | 44 | | 52 | | 47 | | 51 | |
| 63.1084 | | 46 | | 63 | | 46 | | 54 | |
| 63.1288 | | 43 | | 46 | | 56 | | 41 | |
| 63.1493 | | 49 | | 46 | | 43 | | 59 | |
| 63.1697 | | 47 | | 57 | | 57 | | 49 | |
| 63.1902 | | 52 | | 54 | | 52 | | 50 | |
| 63.2106 | | 39 | | 55 | | 53 | | 45 | |
| 63.2311 | | 43 | | 55 | | 46 | | 58 | |
| 63.2515 | | 45 | | 58 | | 65 | | 67 | |
| 63.272 | | 43 | | 53 | | 58 | | 55 | |
| 63.2924 | | 52 | | 41 | | 49 | | 38 | |
| 63.3129 | | 51 | | 65 | | 63 | | 56 | |
| 63.3333 | | 38 | | 47 | | 65 | | 52 | |
| 63.3538 | | 64 | | 77 | | 57 | | 44 | |
| 63.3742 | | 55 | | 54 | | 55 | | 48 | |
| 63.3947 | | 61 | | 44 | | 55 | | 42 | |
| 63.4151 | | 54 | | 45 | | 52 | | 54 | |
| 63.4356 | | 47 | | 49 | | 63 | | 49 | |
| 63.456 | | 47 | | 51 | | 45 | | 47 | |
| 63.4765 | | 60 | | 59 | | 45 | | 54 | |
| 63.497 | | 38 | | 42 | | 63 | | 50 | |
| 63.5174 | | 51 | | 60 | | 59 | | 52 | |
| 63.5379 | | 44 | | 58 | | 45 | | 61 | |
| 63.5583 | | 57 | | 61 | | 54 | | 52 | |
| 63.5788 | | 44 | | 52 | | 59 | | 47 | |
| 63.5992 | | 49 | | 48 | | 52 | | 45 | |
| 63.6197 | | 56 | | 61 | | 64 | | 56 | |
| 63.6401 | | 61 | | 51 | | 46 | | 65 | |
| 63.6606 | | 50 | | 50 | | 63 | | 55 | |
| 63.681 | | 51 | | 61 | | 67 | | 61 | |
| 63.7015 | | 59 | | 47 | | 66 | | 54 | |
| 63.7219 | | 57 | | 60 | | 53 | | 61 | |
| 63.7424 | | 63 | | 68 | | 67 | | 56 | |
| 63.7628 | | 59 | | 57 | | 53 | | 65 | |
| 63.7833 | | 61 | | 61 | | 63 | | 49 | |
| 63.8037 | | 63 | | 68 | | 82 | | 82 | |
| 63.8242 | | 64 | | 80 | | 69 | | 69 | |
| 63.8446 | | 74 | | 74 | | 67 | | 62 | |
| 63.8651 | | 76 | | 78 | | 61 | | 77 | |
| 63.8855 | | 93 | | 93 | | 100 | | 86 | |
| 63.906 | | 89 | | 104 | | 91 | | 105 | |
| 63.9264 | | 127 | | 128 | | 123 | | 123 | |
| 63.9469 | | 160 | | 171 | | 163 | | 171 | |
| 63.9673 | | 231 | | 255 | | 238 | | 285 | |
| 63.9878 | | 426 | | 417 | | 384 | | 450 | |
| 64.0083 | | 762 | | 581 | | 663 | | 689 | |
| 64.0287 | | 671 | | 640 | | 654 | | 655 | |
| 64.0492 | | 361 | | 435 | | 416 | | 408 | |
| 64.0696 | | 225 | | 227 | | 203 | | 246 | |
| 64.0901 | | 129 | | 151 | | 144 | | 163 | |
| 64.1105 | | 124 | | 159 | | 120 | | 166 | |
| 64.131 | | 140 | | 154 | | 152 | | 168 | |
| 64.1514 | | 182 | | 178 | | 196 | | 249 | |
| 64.1719 | | 314 | | 286 | | 240 | | 309 | |
| 64.1923 | | 437 | | 340 | | 388 | | 381 | |
| 64.2128 | | 351 | | 340 | | 288 | | 338 | |
| 64.2332 | | 178 | | 220 | | 220 | | 195 | |
| 64.2537 | | 106 | | 107 | | 105 | | 130 | |
| 64.2741 | | 85 | | 84 | | 96 | | 117 | |
| 64.2946 | | 58 | | 91 | | 81 | | 80 | |
| 64.315 | | 73 | | 69 | | 79 | | 74 | |
| 64.3355 | | 72 | | 81 | | 69 | | 56 | |
| 64.3559 | | 80 | | 61 | | 57 | | 69 | |
| 64.3764 | | 65 | | 76 | | 66 | | 62 | |
| 64.3968 | | 58 | | 68 | | 58 | | 54 | |
| 64.4173 | | 61 | | 53 | | 58 | | 59 | |
| 64.4377 | | 52 | | 70 | | 61 | | 70 | |
| 64.4582 | | 60 | | 72 | | 53 | | 56 | |
| 64.4786 | | 49 | | 64 | | 52 | | 65 | |
| 64.4991 | | 52 | | 68 | | 48 | | 72 | |
| 64.5195 | | 58 | | 62 | | 53 | | 59 | |
| 64.54 | | 60 | | 64 | | 59 | | 68 | |
| 64.5605 | | 62 | | 60 | | 55 | | 72 | |
| 64.5809 | | 57 | | 50 | | 57 | | 45 | |
| 64.6014 | | 63 | | 50 | | 58 | | 58 | |
| 64.6218 | | 56 | | 65 | | 61 | | 55 | |
| 64.6423 | | 60 | | 65 | | 55 | | 60 | |
| 64.6627 | | 44 | | 76 | | 62 | | 62 | |
| 64.6832 | | 62 | | 49 | | 50 | | 45 | |
| 64.7036 | | 44 | | 63 | | 57 | | 54 | |
| 64.7241 | | 45 | | 65 | | 39 | | 52 | |
| 64.7445 | | 43 | | 57 | | 57 | | 61 | |
| 64.765 | | 57 | | 46 | | 46 | | 65 | |
| 64.7854 | | 44 | | 58 | | 60 | | 42 | |
| 64.8059 | | 55 | | 59 | | 66 | | 55 | |
| 64.8263 | | 54 | | 63 | | 56 | | 51 | |
| 64.8468 | | 53 | | 54 | | 48 | | 55 | |
| 64.8672 | | 60 | | 60 | | 53 | | 62 | |
| 64.8877 | | 64 | | 74 | | 56 | | 60 | |
| 64.9081 | | 74 | | 65 | | 60 | | 57 | |
| 64.9286 | | 53 | | 56 | | 65 | | 73 | |
| 64.949 | | 61 | | 73 | | 59 | | 51 | |
| 64.9695 | | 41 | | 72 | | 45 | | 62 | |
| 64.9899 | | 51 | | 61 | | 48 | | 51 | |
| 65.0104 | | 45 | | 60 | | 52 | | 65 | |
| 65.0308 | | 57 | | 47 | | 48 | | 65 | |
| 65.0513 | | 55 | | 73 | | 60 | | 58 | |
| 65.0718 | | 55 | | 76 | | 65 | | 46 | |
| 65.0922 | | 53 | | 56 | | 55 | | 58 | |
| 65.1127 | | 57 | | 47 | | 56 | | 65 | |
| 65.1331 | | 45 | | 54 | | 49 | | 58 | |
| 65.1536 | | 41 | | 61 | | 60 | | 61 | |
| 65.174 | | 54 | | 55 | | 44 | | 52 | |
| 65.1945 | | 51 | | 53 | | 44 | | 50 | |
| 65.2149 | | 50 | | 48 | | 62 | | 55 | |
| 65.2354 | | 55 | | 55 | | 53 | | 55 | |
| 65.2558 | | 56 | | 64 | | 65 | | 45 | |
| 65.2763 | | 45 | | 53 | | 57 | | 57 | |
| 65.2967 | | 56 | | 54 | | 55 | | 64 | |
| 65.3172 | | 60 | | 58 | | 62 | | 48 | |
| 65.3376 | | 57 | | 59 | | 54 | | 56 | |
| 65.3581 | | 49 | | 52 | | 70 | | 56 | |
| 65.3785 | | 76 | | 71 | | 58 | | 68 | |
| 65.399 | | 66 | | 75 | | 67 | | 74 | |
| 65.4194 | | 60 | | 77 | | 61 | | 63 | |
| 65.4399 | | 75 | | 84 | | 58 | | 69 | |
| 65.4603 | | 68 | | 61 | | 63 | | 58 | |
| 65.4808 | | 55 | | 53 | | 52 | | 63 | |
| 65.5012 | | 41 | | 55 | | 54 | | 63 | |
| 65.5217 | | 58 | | 82 | | 70 | | 69 | |
| 65.5421 | | 54 | | 58 | | 48 | | 49 | |
| 65.5626 | | 60 | | 72 | | 62 | | 52 | |
| 65.5831 | | 73 | | 71 | | 55 | | 62 | |
| 65.6035 | | 69 | | 73 | | 61 | | 67 | |
| 65.624 | | 66 | | 57 | | 66 | | 64 | |
| 65.6444 | | 65 | | 55 | | 71 | | 63 | |
| 65.6649 | | 98 | | 78 | | 65 | | 68 | |
| 65.6853 | | 98 | | 96 | | 72 | | 91 | |
| 65.7058 | | 127 | | 84 | | 77 | | 108 | |
| 65.7262 | | 154 | | 133 | | 103 | | 119 | |
| 65.7467 | | 226 | | 143 | | 164 | | 157 | |
| 65.7671 | | 246 | | 224 | | 180 | | 157 | |
| 65.7876 | | 194 | | 197 | | 168 | | 173 | |
| 65.808 | | 114 | | 129 | | 125 | | 112 | |
| 65.8285 | | 99 | | 95 | | 80 | | 91 | |
| 65.8489 | | 81 | | 90 | | 79 | | 76 | |
| 65.8694 | | 87 | | 76 | | 67 | | 87 | |
| 65.8898 | | 95 | | 92 | | 102 | | 83 | |
| 65.9103 | | 115 | | 100 | | 98 | | 93 | |
| 65.9307 | | 146 | | 113 | | 120 | | 117 | |
| 65.9512 | | 134 | | 126 | | 125 | | 108 | |
| 65.9716 | | 134 | | 115 | | 96 | | 100 | |
| 65.9921 | | 82 | | 90 | | 96 | | 99 | |
| 66.0125 | | 68 | | 91 | | 54 | | 68 | |
| 66.033 | | 59 | | 59 | | 77 | | 71 | |
| 66.0534 | | 53 | | 66 | | 66 | | 55 | |
| 66.0739 | | 53 | | 57 | | 67 | | 56 | |
| 66.0943 | | 65 | | 52 | | 60 | | 52 | |
| 66.1148 | | 59 | | 44 | | 56 | | 52 | |
| 66.1353 | | 59 | | 47 | | 47 | | 40 | |
| 66.1557 | | 40 | | 61 | | 54 | | 67 | |
| 66.1762 | | 57 | | 66 | | 56 | | 54 | |
| 66.1966 | | 55 | | 59 | | 54 | | 58 | |
| 66.2171 | | 49 | | 64 | | 66 | | 53 | |
| 66.2375 | | 54 | | 55 | | 67 | | 63 | |
| 66.258 | | 51 | | 62 | | 50 | | 57 | |
| 66.2784 | | 56 | | 56 | | 52 | | 65 | |
| 66.2989 | | 53 | | 51 | | 43 | | 64 | |
| 66.3193 | | 54 | | 58 | | 52 | | 60 | |
| 66.3398 | | 50 | | 53 | | 57 | | 59 | |
| 66.3602 | | 54 | | 61 | | 54 | | 56 | |
| 66.3807 | | 45 | | 43 | | 47 | | 59 | |
| 66.4011 | | 42 | | 60 | | 51 | | 43 | |
| 66.4216 | | 58 | | 61 | | 70 | | 61 | |
| 66.442 | | 50 | | 60 | | 51 | | 53 | |
| 66.4625 | | 56 | | 71 | | 72 | | 56 | |
| 66.4829 | | 42 | | 51 | | 47 | | 54 | |
| 66.5034 | | 54 | | 64 | | 65 | | 59 | |
| 66.5238 | | 55 | | 40 | | 62 | | 50 | |
| 66.5443 | | 67 | | 61 | | 49 | | 52 | |
| 66.5647 | | 51 | | 60 | | 62 | | 55 | |
| 66.5852 | | 47 | | 60 | | 54 | | 65 | |
| 66.6056 | | 55 | | 57 | | 69 | | 63 | |
| 66.6261 | | 51 | | 41 | | 50 | | 46 | |
| 66.6466 | | 57 | | 41 | | 61 | | 64 | |
| 66.667 | | 58 | | 60 | | 45 | | 56 | |
| 66.6875 | | 46 | | 53 | | 52 | | 61 | |
| 66.7079 | | 57 | | 60 | | 69 | | 67 | |
| 66.7284 | | 47 | | 68 | | 55 | | 40 | |
| 66.7488 | | 62 | | 50 | | 57 | | 56 | |
| 66.7693 | | 58 | | 72 | | 52 | | 58 | |
| 66.7897 | | 54 | | 57 | | 64 | | 48 | |
| 66.8102 | | 58 | | 60 | | 58 | | 47 | |
| 66.8306 | | 54 | | 70 | | 45 | | 52 | |
| 66.8511 | | 62 | | 55 | | 67 | | 52 | |
| 66.8715 | | 57 | | 63 | | 70 | | 59 | |
| 66.892 | | 63 | | 60 | | 54 | | 59 | |
| 66.9124 | | 59 | | 57 | | 54 | | 65 | |
| 66.9329 | | 48 | | 66 | | 56 | | 65 | |
| 66.9533 | | 47 | | 75 | | 52 | | 57 | |
| 66.9738 | | 56 | | 58 | | 67 | | 58 | |
| 66.9942 | | 55 | | 70 | | 59 | | 59 | |
| 67.0147 | | 53 | | 58 | | 77 | | 57 | |
| 67.0351 | | 52 | | 51 | | 64 | | 55 | |
| 67.0556 | | 52 | | 60 | | 62 | | 81 | |
| 67.076 | | 69 | | 66 | | 63 | | 53 | |
| 67.0965 | | 60 | | 63 | | 73 | | 68 | |
| 67.1169 | | 54 | | 70 | | 52 | | 57 | |
| 67.1374 | | 58 | | 65 | | 70 | | 69 | |
| 67.1579 | | 75 | | 66 | | 68 | | 64 | |
| 67.1783 | | 67 | | 76 | | 66 | | 84 | |
| 67.1988 | | 59 | | 72 | | 66 | | 80 | |
| 67.2192 | | 88 | | 100 | | 71 | | 70 | |
| 67.2397 | | 76 | | 63 | | 73 | | 86 | |
| 67.2601 | | 78 | | 92 | | 88 | | 99 | |
| 67.2806 | | 97 | | 98 | | 99 | | 83 | |
| 67.301 | | 85 | | 96 | | 99 | | 91 | |
| 67.3215 | | 83 | | 102 | | 99 | | 98 | |
| 67.3419 | | 80 | | 73 | | 108 | | 95 | |
| 67.3624 | | 94 | | 105 | | 101 | | 83 | |
| 67.3828 | | 97 | | 97 | | 86 | | 96 | |
| 67.4033 | | 90 | | 104 | | 97 | | 113 | |
| 67.4237 | | 107 | | 115 | | 95 | | 94 | |
| 67.4442 | | 100 | | 113 | | 104 | | 105 | |
| 67.4646 | | 103 | | 100 | | 97 | | 107 | |
| 67.4851 | | 103 | | 112 | | 108 | | 119 | |
| 67.5055 | | 116 | | 138 | | 114 | | 120 | |
| 67.526 | | 115 | | 139 | | 125 | | 131 | |
| 67.5464 | | 135 | | 133 | | 141 | | 128 | |
| 67.5669 | | 118 | | 174 | | 159 | | 184 | |
| 67.5873 | | 180 | | 201 | | 174 | | 202 | |
| 67.6078 | | 237 | | 257 | | 192 | | 265 | |
| 67.6282 | | 309 | | 326 | | 261 | | 325 | |
| 67.6487 | | 419 | | 470 | | 419 | | 456 | |
| 67.6691 | | 725 | | 651 | | 626 | | 720 | |
| 67.6896 | | 1198 | | 1006 | | 996 | | 1230 | |
| 67.7101 | | 1948 | | 1667 | | 1711 | | 1819 | |
| 67.7305 | | 2211 | | 2398 | | 2240 | | 2084 | |
| 67.751 | | 1397 | | 1949 | | 1596 | | 1412 | |
| 67.7714 | | 778 | | 951 | | 778 | | 849 | |
| 67.7919 | | 421 | | 561 | | 522 | | 540 | |
| 67.8123 | | 339 | | 419 | | 339 | | 461 | |
| 67.8328 | | 342 | | 372 | | 382 | | 409 | |
| 67.8532 | | 441 | | 437 | | 455 | | 469 | |
| 67.8737 | | 653 | | 591 | | 604 | | 615 | |
| 67.8941 | | 914 | | 835 | | 818 | | 935 | |
| 67.9146 | | 1224 | | 1283 | | 1137 | | 1165 | |
| 67.935 | | 990 | | 1264 | | 1099 | | 998 | |
| 67.9555 | | 587 | | 744 | | 706 | | 635 | |
| 67.9759 | | 390 | | 494 | | 438 | | 474 | |
| 67.9964 | | 358 | | 377 | | 391 | | 426 | |
| 68.0168 | | 366 | | 386 | | 370 | | 397 | |
| 68.0373 | | 430 | | 486 | | 421 | | 551 | |
| 68.0577 | | 658 | | 641 | | 559 | | 708 | |
| 68.0782 | | 1058 | | 926 | | 851 | | 1064 | |
| 68.0986 | | 1653 | | 1416 | | 1418 | | 1644 | |
| 68.1191 | | 2360 | | 2332 | | 2120 | | 2408 | |
| 68.1395 | | 2155 | | 2610 | | 2124 | | 2174 | |
| 68.16 | | 1105 | | 1498 | | 1244 | | 1336 | |
| 68.1804 | | 613 | | 849 | | 673 | | 896 | |
| 68.2009 | | 576 | | 667 | | 560 | | 686 | |
| 68.2214 | | 596 | | 671 | | 595 | | 695 | |
| 68.2418 | | 822 | | 808 | | 789 | | 986 | |
| 68.2623 | | 1331 | | 1158 | | 1156 | | 1370 | |
| 68.2827 | | 2143 | | 1827 | | 2018 | | 2100 | |
| 68.3032 | | 2602 | | 2478 | | 2617 | | 2660 | |
| 68.3236 | | 2056 | | 2331 | | 2191 | | 2230 | |
| 68.3441 | | 1169 | | 1437 | | 1315 | | 1350 | |
| 68.3645 | | 570 | | 860 | | 700 | | 850 | |
| 68.385 | | 408 | | 539 | | 460 | | 524 | |
| 68.4054 | | 336 | | 382 | | 409 | | 443 | |
| 68.4259 | | 360 | | 419 | | 388 | | 408 | |
| 68.4463 | | 485 | | 458 | | 493 | | 545 | |
| 68.4668 | | 702 | | 652 | | 753 | | 695 | |
| 68.4872 | | 847 | | 774 | | 947 | | 912 | |
| 68.5077 | | 677 | | 774 | | 789 | | 781 | |
| 68.5281 | | 369 | | 493 | | 481 | | 496 | |
| 68.5486 | | 224 | | 269 | | 281 | | 336 | |
| 68.569 | | 160 | | 217 | | 190 | | 209 | |
| 68.5895 | | 123 | | 145 | | 118 | | 140 | |
| 68.6099 | | 114 | | 118 | | 128 | | 137 | |
| 68.6304 | | 112 | | 136 | | 131 | | 131 | |
| 68.6508 | | 94 | | 103 | | 111 | | 115 | |
| 68.6713 | | 73 | | 126 | | 96 | | 111 | |
| 68.6917 | | 95 | | 103 | | 94 | | 96 | |
| 68.7122 | | 89 | | 108 | | 64 | | 71 | |
| 68.7326 | | 72 | | 79 | | 84 | | 87 | |
| 68.7531 | | 75 | | 90 | | 82 | | 69 | |
| 68.7736 | | 70 | | 99 | | 88 | | 83 | |
| 68.794 | | 73 | | 77 | | 83 | | 77 | |
| 68.8145 | | 61 | | 94 | | 77 | | 59 | |
| 68.8349 | | 78 | | 79 | | 83 | | 67 | |
| 68.8554 | | 81 | | 77 | | 63 | | 75 | |
| 68.8758 | | 77 | | 94 | | 78 | | 74 | |
| 68.8963 | | 71 | | 79 | | 70 | | 82 | |
| 68.9167 | | 85 | | 91 | | 70 | | 74 | |
| 68.9372 | | 64 | | 70 | | 66 | | 46 | |
| 68.9576 | | 61 | | 77 | | 83 | | 84 | |
| 68.9781 | | 71 | | 76 | | 95 | | 82 | |
| 68.9985 | | 74 | | 75 | | 88 | | 84 | |
| 69.019 | | 84 | | 86 | | 67 | | 68 | |
| 69.0394 | | 86 | | 79 | | 72 | | 72 | |
| 69.0599 | | 62 | | 84 | | 68 | | 63 | |
| 69.0803 | | 59 | | 67 | | 68 | | 72 | |
| 69.1008 | | 79 | | 48 | | 64 | | 63 | |
| 69.1212 | | 56 | | 64 | | 74 | | 54 | |
| 69.1417 | | 63 | | 65 | | 64 | | 59 | |
| 69.1621 | | 80 | | 72 | | 61 | | 52 | |
| 69.1826 | | 62 | | 66 | | 62 | | 64 | |
| 69.203 | | 71 | | 52 | | 66 | | 71 | |
| 69.2235 | | 47 | | 65 | | 58 | | 76 | |
| 69.2439 | | 65 | | 74 | | 65 | | 72 | |
| 69.2644 | | 65 | | 60 | | 45 | | 64 | |
| 69.2849 | | 62 | | 61 | | 50 | | 75 | |
| 69.3053 | | 63 | | 47 | | 71 | | 51 | |
| 69.3258 | | 49 | | 50 | | 61 | | 68 | |
| 69.3462 | | 54 | | 67 | | 59 | | 58 | |
| 69.3667 | | 51 | | 61 | | 59 | | 62 | |
| 69.3871 | | 55 | | 53 | | 63 | | 60 | |
| 69.4076 | | 56 | | 41 | | 53 | | 73 | |
| 69.428 | | 55 | | 57 | | 50 | | 52 | |
| 69.4485 | | 48 | | 55 | | 62 | | 57 | |
| 69.4689 | | 57 | | 60 | | 70 | | 47 | |
| 69.4894 | | 61 | | 52 | | 49 | | 66 | |
| 69.5098 | | 49 | | 56 | | 56 | | 58 | |
| 69.5303 | | 50 | | 48 | | 66 | | 62 | |
| 69.5507 | | 50 | | 54 | | 50 | | 63 | |
| 69.5712 | | 58 | | 61 | | 71 | | 57 | |
| 69.5916 | | 56 | | 70 | | 63 | | 53 | |
| 69.6121 | | 58 | | 52 | | 50 | | 44 | |
| 69.6325 | | 51 | | 40 | | 58 | | 64 | |
| 69.653 | | 52 | | 57 | | 60 | | 59 | |
| 69.6734 | | 47 | | 59 | | 47 | | 47 | |
| 69.6939 | | 50 | | 49 | | 54 | | 75 | |
| 69.7143 | | 50 | | 49 | | 55 | | 37 | |
| 69.7348 | | 59 | | 77 | | 47 | | 50 | |
| 69.7552 | | 59 | | 66 | | 62 | | 53 | |
| 69.7757 | | 48 | | 63 | | 54 | | 47 | |
| 69.7962 | | 60 | | 51 | | 54 | | 47 | |
| 69.8166 | | 41 | | 46 | | 52 | | 40 | |
| 69.8371 | | 46 | | 45 | | 55 | | 69 | |
| 69.8575 | | 66 | | 67 | | 56 | | 61 | |
| 69.878 | | 39 | | 51 | | 60 | | 54 | |
| 69.8984 | | 62 | | 60 | | 63 | | 55 | |
| 69.9189 | | 50 | | 67 | | 46 | | 60 | |
| 69.9393 | | 54 | | 56 | | 57 | | 51 | |
| 69.9598 | | 45 | | 52 | | 58 | | 61 | |
| 69.9802 | | 46 | | 55 | | 64 | | 55 | |
| 70.0007 | | 56 | | 53 | | 50 | | 47 | |
| 70.0211 | | 53 | | 52 | | 50 | | 43 | |
| 70.0416 | | 44 | | 46 | | 70 | | 51 | |
| 70.062 | | 41 | | 59 | | 50 | | 59 | |
| 70.0825 | | 57 | | 61 | | 50 | | 52 | |
| 70.1029 | | 60 | | 79 | | 61 | | 47 | |
| 70.1234 | | 62 | | 53 | | 64 | | 47 | |
| 70.1438 | | 44 | | 50 | | 59 | | 50 | |
| 70.1643 | | 57 | | 60 | | 47 | | 48 | |
| 70.1847 | | 63 | | 46 | | 47 | | 56 | |
| 70.2052 | | 48 | | 59 | | 53 | | 54 | |
| 70.2256 | | 47 | | 41 | | 56 | | 57 | |
| 70.2461 | | 57 | | 46 | | 47 | | 54 | |
| 70.2665 | | 54 | | 45 | | 51 | | 51 | |
| 70.287 | | 59 | | 50 | | 55 | | 51 | |
| 70.3074 | | 46 | | 59 | | 54 | | 52 | |
| 70.3279 | | 39 | | 54 | | 57 | | 62 | |
| 70.3484 | | 55 | | 59 | | 43 | | 56 | |
| 70.3688 | | 58 | | 54 | | 53 | | 61 | |
| 70.3893 | | 42 | | 58 | | 56 | | 44 | |
| 70.4097 | | 49 | | 52 | | 43 | | 50 | |
| 70.4302 | | 50 | | 67 | | 55 | | 55 | |
| 70.4506 | | 41 | | 58 | | 59 | | 58 | |
| 70.4711 | | 52 | | 47 | | 54 | | 47 | |
| 70.4915 | | 53 | | 51 | | 46 | | 58 | |
| 70.512 | | 44 | | 52 | | 51 | | 57 | |
| 70.5324 | | 53 | | 56 | | 53 | | 62 | |
| 70.5529 | | 51 | | 69 | | 48 | | 44 | |
| 70.5733 | | 61 | | 76 | | 56 | | 68 | |
| 70.5938 | | 51 | | 54 | | 62 | | 45 | |
| 70.6142 | | 63 | | 44 | | 49 | | 61 | |
| 70.6347 | | 59 | | 57 | | 63 | | 61 | |
| 70.6551 | | 51 | | 53 | | 49 | | 49 | |
| 70.6756 | | 44 | | 42 | | 67 | | 53 | |
| 70.696 | | 50 | | 46 | | 46 | | 46 | |
| 70.7165 | | 48 | | 38 | | 48 | | 49 | |
| 70.7369 | | 53 | | 52 | | 49 | | 51 | |
| 70.7574 | | 56 | | 50 | | 61 | | 50 | |
| 70.7778 | | 60 | | 59 | | 52 | | 49 | |
| 70.7983 | | 45 | | 50 | | 48 | | 64 | |
| 70.8187 | | 60 | | 58 | | 48 | | 61 | |
| 70.8392 | | 64 | | 65 | | 68 | | 69 | |
| 70.8597 | | 67 | | 64 | | 59 | | 68 | |
| 70.8801 | | 57 | | 74 | | 57 | | 70 | |
| 70.9006 | | 69 | | 77 | | 61 | | 86 | |
| 70.921 | | 60 | | 65 | | 84 | | 52 | |
| 70.9415 | | 66 | | 62 | | 60 | | 69 | |
| 70.9619 | | 68 | | 64 | | 60 | | 60 | |
| 70.9824 | | 55 | | 71 | | 66 | | 65 | |
| 71.0028 | | 46 | | 63 | | 60 | | 76 | |
| 71.0233 | | 57 | | 56 | | 62 | | 55 | |
| 71.0437 | | 47 | | 43 | | 65 | | 75 | |
| 71.0642 | | 47 | | 49 | | 49 | | 38 | |
| 71.0846 | | 59 | | 65 | | 46 | | 64 | |
| 71.1051 | | 32 | | 55 | | 62 | | 49 | |
| 71.1255 | | 57 | | 41 | | 55 | | 45 | |
| 71.146 | | 46 | | 55 | | 40 | | 47 | |
| 71.1664 | | 56 | | 50 | | 54 | | 48 | |
| 71.1869 | | 50 | | 67 | | 45 | | 70 | |
| 71.2073 | | 49 | | 48 | | 62 | | 56 | |
| 71.2278 | | 51 | | 54 | | 42 | | 56 | |
| 71.2482 | | 51 | | 49 | | 53 | | 55 | |
| 71.2687 | | 44 | | 51 | | 47 | | 57 | |
| 71.2891 | | 49 | | 43 | | 54 | | 51 | |
| 71.3096 | | 51 | | 55 | | 47 | | 53 | |
| 71.33 | | 43 | | 48 | | 51 | | 67 | |
| 71.3505 | | 50 | | 50 | | 44 | | 60 | |
| 71.371 | | 51 | | 46 | | 39 | | 51 | |
| 71.3914 | | 52 | | 51 | | 47 | | 51 | |
| 71.4119 | | 50 | | 56 | | 47 | | 62 | |
| 71.4323 | | 41 | | 56 | | 67 | | 42 | |
| 71.4528 | | 52 | | 53 | | 47 | | 38 | |
| 71.4732 | | 41 | | 62 | | 31 | | 48 | |
| 71.4937 | | 54 | | 55 | | 57 | | 48 | |
| 71.5141 | | 57 | | 47 | | 50 | | 48 | |
| 71.5346 | | 47 | | 48 | | 41 | | 49 | |
| 71.555 | | 49 | | 50 | | 51 | | 45 | |
| 71.5755 | | 34 | | 46 | | 48 | | 48 | |
| 71.5959 | | 48 | | 48 | | 55 | | 47 | |
| 71.6164 | | 38 | | 56 | | 54 | | 65 | |
| 71.6368 | | 56 | | 46 | | 61 | | 53 | |
| 71.6573 | | 49 | | 55 | | 62 | | 51 | |
| 71.6777 | | 39 | | 54 | | 56 | | 45 | |
| 71.6982 | | 65 | | 53 | | 62 | | 55 | |
| 71.7186 | | 52 | | 58 | | 43 | | 47 | |
| 71.7391 | | 56 | | 63 | | 50 | | 42 | |
| 71.7595 | | 36 | | 53 | | 49 | | 45 | |
| 71.78 | | 56 | | 58 | | 43 | | 39 | |
| 71.8004 | | 45 | | 57 | | 57 | | 47 | |
| 71.8209 | | 46 | | 53 | | 46 | | 58 | |
| 71.8413 | | 50 | | 40 | | 65 | | 48 | |
| 71.8618 | | 41 | | 63 | | 52 | | 58 | |
| 71.8822 | | 61 | | 55 | | 61 | | 40 | |
| 71.9027 | | 48 | | 55 | | 67 | | 52 | |
| 71.9232 | | 52 | | 53 | | 61 | | 60 | |
| 71.9436 | | 59 | | 61 | | 52 | | 52 | |
| 71.9641 | | 60 | | 55 | | 51 | | 57 | |
| 71.9845 | | 74 | | 61 | | 63 | | 60 | |
| 72.005 | | 82 | | 72 | | 68 | | 70 | |
| 72.0254 | | 57 | | 61 | | 72 | | 58 | |
| 72.0459 | | 50 | | 72 | | 76 | | 47 | |
| 72.0663 | | 60 | | 46 | | 48 | | 64 | |
| 72.0868 | | 51 | | 56 | | 52 | | 52 | |
| 72.1072 | | 45 | | 58 | | 38 | | 53 | |
| 72.1277 | | 52 | | 45 | | 64 | | 50 | |
| 72.1481 | | 56 | | 63 | | 53 | | 59 | |
| 72.1686 | | 50 | | 50 | | 54 | | 45 | |
| 72.189 | | 61 | | 61 | | 63 | | 62 | |
| 72.2095 | | 60 | | 61 | | 48 | | 42 | |
| 72.2299 | | 73 | | 46 | | 74 | | 75 | |
| 72.2504 | | 81 | | 64 | | 54 | | 67 | |
| 72.2708 | | 60 | | 73 | | 74 | | 54 | |
| 72.2913 | | 62 | | 74 | | 63 | | 64 | |
| 72.3117 | | 58 | | 59 | | 60 | | 61 | |
| 72.3322 | | 51 | | 63 | | 52 | | 50 | |
| 72.3526 | | 41 | | 55 | | 67 | | 60 | |
| 72.3731 | | 54 | | 48 | | 59 | | 40 | |
| 72.3935 | | 61 | | 66 | | 60 | | 61 | |
| 72.414 | | 41 | | 52 | | 50 | | 40 | |
| 72.4345 | | 53 | | 47 | | 56 | | 44 | |
| 72.4549 | | 57 | | 52 | | 61 | | 57 | |
| 72.4754 | | 56 | | 57 | | 54 | | 61 | |
| 72.4958 | | 48 | | 58 | | 43 | | 43 | |
| 72.5163 | | 48 | | 59 | | 36 | | 57 | |
| 72.5367 | | 47 | | 52 | | 63 | | 54 | |
| 72.5572 | | 56 | | 45 | | 48 | | 59 | |
| 72.5776 | | 41 | | 45 | | 49 | | 58 | |
| 72.5981 | | 55 | | 51 | | 62 | | 51 | |
| 72.6185 | | 50 | | 47 | | 43 | | 55 | |
| 72.639 | | 45 | | 60 | | 56 | | 48 | |
| 72.6594 | | 48 | | 38 | | 54 | | 43 | |
| 72.6799 | | 48 | | 48 | | 51 | | 51 | |
| 72.7003 | | 37 | | 65 | | 49 | | 49 | |
| 72.7208 | | 52 | | 51 | | 54 | | 45 | |
| 72.7412 | | 38 | | 47 | | 53 | | 41 | |
| 72.7617 | | 41 | | 42 | | 65 | | 56 | |
| 72.7821 | | 52 | | 57 | | 60 | | 48 | |
| 72.8026 | | 63 | | 56 | | 56 | | 53 | |
| 72.823 | | 48 | | 71 | | 63 | | 46 | |
| 72.8435 | | 47 | | 43 | | 58 | | 45 | |
| 72.8639 | | 51 | | 58 | | 50 | | 49 | |
| 72.8844 | | 52 | | 42 | | 68 | | 45 | |
| 72.9048 | | 69 | | 46 | | 52 | | 46 | |
| 72.9253 | | 40 | | 55 | | 50 | | 48 | |
| 72.9458 | | 45 | | 57 | | 51 | | 45 | |
| 72.9662 | | 53 | | 57 | | 54 | | 51 | |
| 72.9867 | | 46 | | 47 | | 62 | | 56 | |
| 73.0071 | | 53 | | 56 | | 51 | | 39 | |
| 73.0276 | | 49 | | 42 | | 42 | | 50 | |
| 73.048 | | 56 | | 68 | | 57 | | 61 | |
| 73.0685 | | 62 | | 49 | | 41 | | 70 | |
| 73.0889 | | 73 | | 61 | | 60 | | 56 | |
| 73.1094 | | 71 | | 55 | | 67 | | 49 | |
| 73.1298 | | 37 | | 63 | | 64 | | 70 | |
| 73.1503 | | 37 | | 49 | | 65 | | 54 | |
| 73.1707 | | 67 | | 57 | | 65 | | 55 | |
| 73.1912 | | 63 | | 60 | | 50 | | 57 | |
| 73.2116 | | 60 | | 89 | | 72 | | 56 | |
| 73.2321 | | 70 | | 69 | | 69 | | 74 | |
| 73.2525 | | 62 | | 68 | | 63 | | 73 | |
| 73.273 | | 75 | | 79 | | 79 | | 75 | |
| 73.2934 | | 84 | | 88 | | 72 | | 76 | |
| 73.3139 | | 94 | | 96 | | 106 | | 86 | |
| 73.3343 | | 106 | | 125 | | 103 | | 111 | |
| 73.3548 | | 135 | | 154 | | 130 | | 136 | |
| 73.3752 | | 178 | | 178 | | 195 | | 220 | |
| 73.3957 | | 298 | | 289 | | 269 | | 314 | |
| 73.4161 | | 399 | | 382 | | 410 | | 412 | |
| 73.4366 | | 584 | | 561 | | 608 | | 566 | |
| 73.457 | | 526 | | 688 | | 641 | | 580 | |
| 73.4775 | | 336 | | 501 | | 420 | | 412 | |
| 73.498 | | 216 | | 264 | | 228 | | 244 | |
| 73.5184 | | 132 | | 170 | | 179 | | 167 | |
| 73.5389 | | 123 | | 161 | | 127 | | 120 | |
| 73.5593 | | 98 | | 128 | | 118 | | 123 | |
| 73.5798 | | 124 | | 151 | | 144 | | 116 | |
| 73.6002 | | 156 | | 173 | | 153 | | 165 | |
| 73.6207 | | 206 | | 207 | | 200 | | 203 | |
| 73.6411 | | 297 | | 294 | | 286 | | 273 | |
| 73.6616 | | 315 | | 374 | | 343 | | 326 | |
| 73.682 | | 236 | | 285 | | 293 | | 262 | |
| 73.7025 | | 152 | | 227 | | 189 | | 176 | |
| 73.7229 | | 90 | | 133 | | 130 | | 123 | |
| 73.7434 | | 65 | | 75 | | 99 | | 96 | |
| 73.7638 | | 79 | | 92 | | 83 | | 83 | |
| 73.7843 | | 65 | | 78 | | 72 | | 54 | |
| 73.8047 | | 67 | | 63 | | 71 | | 58 | |
| 73.8252 | | 57 | | 81 | | 62 | | 65 | |
| 73.8456 | | 53 | | 77 | | 53 | | 60 | |
| 73.8661 | | 46 | | 65 | | 62 | | 61 | |
| 73.8865 | | 45 | | 57 | | 67 | | 54 | |
| 73.907 | | 58 | | 73 | | 58 | | 64 | |
| 73.9274 | | 48 | | 56 | | 44 | | 67 | |
| 73.9479 | | 49 | | 62 | | 52 | | 53 | |
| 73.9683 | | 58 | | 63 | | 58 | | 49 | |
| 73.9888 | | 46 | | 61 | | 62 | | 59 | |
| 74.0093 | | 70 | | 67 | | 59 | | 53 | |
| 74.0297 | | 40 | | 48 | | 43 | | 46 | |
| 74.0502 | | 58 | | 53 | | 36 | | 53 | |
| 74.0706 | | 58 | | 55 | | 54 | | 49 | |
| 74.0911 | | 45 | | 62 | | 59 | | 52 | |
| 74.1115 | | 52 | | 55 | | 52 | | 63 | |
| 74.132 | | 50 | | 45 | | 50 | | 49 | |
| 74.1524 | | 55 | | 61 | | 47 | | 71 | |
| 74.1729 | | 57 | | 65 | | 67 | | 81 | |
| 74.1933 | | 69 | | 58 | | 60 | | 59 | |
| 74.2138 | | 56 | | 66 | | 62 | | 57 | |
| 74.2342 | | 71 | | 67 | | 58 | | 59 | |
| 74.2547 | | 69 | | 71 | | 80 | | 54 | |
| 74.2751 | | 67 | | 78 | | 55 | | 50 | |
| 74.2956 | | 55 | | 60 | | 78 | | 57 | |
| 74.316 | | 54 | | 52 | | 46 | | 48 | |
| 74.3365 | | 49 | | 47 | | 48 | | 51 | |
| 74.3569 | | 52 | | 40 | | 64 | | 46 | |
| 74.3774 | | 41 | | 63 | | 47 | | 53 | |
| 74.3978 | | 48 | | 48 | | 62 | | 59 | |
| 74.4183 | | 63 | | 57 | | 49 | | 57 | |
| 74.4387 | | 44 | | 45 | | 63 | | 60 | |
| 74.4592 | | 47 | | 51 | | 50 | | 50 | |
| 74.4796 | | 47 | | 62 | | 56 | | 60 | |
| 74.5001 | | 41 | | 44 | | 50 | | 43 | |
| 74.5206 | | 49 | | 40 | | 43 | | 39 | |
| 74.541 | | 39 | | 50 | | 45 | | 41 | |
| 74.5615 | | 48 | | 50 | | 66 | | 52 | |
| 74.5819 | | 45 | | 60 | | 58 | | 52 | |
| 74.6024 | | 51 | | 52 | | 65 | | 48 | |
| 74.6228 | | 58 | | 38 | | 46 | | 45 | |
| 74.6433 | | 48 | | 50 | | 55 | | 47 | |
| 74.6637 | | 46 | | 50 | | 57 | | 46 | |
| 74.6842 | | 48 | | 50 | | 43 | | 51 | |
| 74.7046 | | 41 | | 61 | | 39 | | 39 | |
| 74.7251 | | 46 | | 55 | | 52 | | 42 | |
| 74.7455 | | 61 | | 49 | | 58 | | 48 | |
| 74.766 | | 56 | | 48 | | 47 | | 60 | |
| 74.7864 | | 41 | | 59 | | 69 | | 44 | |
| 74.8069 | | 49 | | 48 | | 58 | | 59 | |
| 74.8273 | | 56 | | 54 | | 67 | | 63 | |
| 74.8478 | | 33 | | 63 | | 54 | | 61 | |
| 74.8682 | | 56 | | 44 | | 63 | | 54 | |
| 74.8887 | | 57 | | 54 | | 72 | | 56 | |
| 74.9091 | | 64 | | 41 | | 39 | | 51 | |
| 74.9296 | | 49 | | 55 | | 48 | | 47 | |
| 74.95 | | 51 | | 60 | | 48 | | 54 | |
| 74.9705 | | 46 | | 41 | | 54 | | 46 | |
| 74.9909 | | 52 | | 55 | | 34 | | 45 | |
| 75.0114 | | 46 | | 55 | | 60 | | 50 | |
| 75.0318 | | 53 | | 58 | | 53 | | 51 | |
| 75.0523 | | 61 | | 38 | | 46 | | 61 | |
| 75.0728 | | 51 | | 59 | | 61 | | 45 | |
| 75.0932 | | 45 | | 61 | | 52 | | 55 | |
| 75.1137 | | 57 | | 55 | | 53 | | 51 | |
| 75.1341 | | 50 | | 66 | | 61 | | 56 | |
| 75.1546 | | 66 | | 70 | | 54 | | 62 | |
| 75.175 | | 50 | | 55 | | 54 | | 63 | |
| 75.1955 | | 76 | | 71 | | 58 | | 52 | |
| 75.2159 | | 59 | | 69 | | 59 | | 68 | |
| 75.2364 | | 64 | | 68 | | 48 | | 55 | |
| 75.2568 | | 45 | | 48 | | 59 | | 62 | |
| 75.2773 | | 71 | | 72 | | 58 | | 60 | |
| 75.2977 | | 63 | | 60 | | 65 | | 69 | |
| 75.3182 | | 58 | | 61 | | 56 | | 50 | |
| 75.3386 | | 68 | | 57 | | 62 | | 68 | |
| 75.3591 | | 67 | | 69 | | 74 | | 64 | |
| 75.3795 | | 82 | | 53 | | 71 | | 58 | |
| 75.4 | | 72 | | 94 | | 66 | | 69 | |
| 75.4204 | | 79 | | 85 | | 71 | | 78 | |
| 75.4409 | | 74 | | 63 | | 75 | | 109 | |
| 75.4613 | | 81 | | 85 | | 72 | | 90 | |
| 75.4818 | | 103 | | 88 | | 95 | | 92 | |
| 75.5022 | | 123 | | 121 | | 110 | | 117 | |
| 75.5227 | | 127 | | 127 | | 124 | | 142 | |
| 75.5431 | | 160 | | 189 | | 175 | | 209 | |
| 75.5636 | | 256 | | 219 | | 232 | | 267 | |
| 75.5841 | | 350 | | 314 | | 376 | | 390 | |
| 75.6045 | | 583 | | 510 | | 584 | | 570 | |
| 75.625 | | 930 | | 769 | | 992 | | 809 | |
| 75.6454 | | 1129 | | 1037 | | 1154 | | 959 | |
| 75.6659 | | 724 | | 788 | | 874 | | 698 | |
| 75.6863 | | 400 | | 400 | | 443 | | 442 | |
| 75.7068 | | 216 | | 261 | | 257 | | 284 | |
| 75.7272 | | 163 | | 184 | | 197 | | 197 | |
| 75.7477 | | 147 | | 172 | | 180 | | 181 | |
| 75.7681 | | 179 | | 143 | | 147 | | 163 | |
| 75.7886 | | 146 | | 180 | | 172 | | 184 | |
| 75.809 | | 257 | | 229 | | 233 | | 251 | |
| 75.8295 | | 374 | | 292 | | 355 | | 374 | |
| 75.8499 | | 489 | | 403 | | 532 | | 480 | |
| 75.8704 | | 553 | | 480 | | 609 | | 506 | |
| 75.8908 | | 392 | | 384 | | 478 | | 381 | |
| 75.9113 | | 230 | | 233 | | 264 | | 253 | |
| 75.9317 | | 151 | | 160 | | 145 | | 150 | |
| 75.9522 | | 95 | | 119 | | 103 | | 120 | |
| 75.9726 | | 84 | | 83 | | 88 | | 106 | |
| 75.9931 | | 91 | | 89 | | 73 | | 102 | |
| 76.0135 | | 68 | | 74 | | 71 | | 75 | |
| 76.034 | | 61 | | 81 | | 60 | | 71 | |
| 76.0544 | | 81 | | 65 | | 62 | | 66 | |
| 76.0749 | | 46 | | 66 | | 65 | | 63 | |
| 76.0953 | | 53 | | 59 | | 47 | | 64 | |
| 76.1158 | | 52 | | 69 | | 69 | | 54 | |
| 76.1363 | | 56 | | 54 | | 51 | | 69 | |
| 76.1567 | | 48 | | 62 | | 64 | | 46 | |
| 76.1772 | | 47 | | 67 | | 51 | | 73 | |
| 76.1976 | | 63 | | 60 | | 54 | | 54 | |
| 76.2181 | | 44 | | 67 | | 56 | | 65 | |
| 76.2385 | | 49 | | 53 | | 44 | | 49 | |
| 76.259 | | 51 | | 54 | | 55 | | 64 | |
| 76.2794 | | 54 | | 54 | | 53 | | 46 | |
| 76.2999 | | 45 | | 52 | | 55 | | 45 | |
| 76.3203 | | 52 | | 60 | | 48 | | 54 | |
| 76.3408 | | 48 | | 61 | | 64 | | 44 | |
| 76.3612 | | 49 | | 43 | | 58 | | 42 | |
| 76.3817 | | 49 | | 46 | | 50 | | 56 | |
| 76.4021 | | 45 | | 53 | | 57 | | 44 | |
| 76.4226 | | 61 | | 49 | | 62 | | 34 | |
| 76.443 | | 51 | | 44 | | 54 | | 51 | |
| 76.4635 | | 34 | | 53 | | 56 | | 52 | |
| 76.4839 | | 64 | | 64 | | 60 | | 41 | |
| 76.5044 | | 45 | | 47 | | 45 | | 60 | |
| 76.5248 | | 56 | | 74 | | 54 | | 48 | |
| 76.5453 | | 61 | | 55 | | 47 | | 48 | |
| 76.5657 | | 43 | | 53 | | 46 | | 61 | |
| 76.5862 | | 54 | | 49 | | 53 | | 54 | |
| 76.6066 | | 42 | | 48 | | 44 | | 41 | |
| 76.6271 | | 51 | | 50 | | 45 | | 44 | |
| 76.6476 | | 39 | | 57 | | 53 | | 38 | |
| 76.668 | | 57 | | 60 | | 41 | | 51 | |
| 76.6885 | | 45 | | 59 | | 53 | | 45 | |
| 76.7089 | | 51 | | 43 | | 50 | | 43 | |
| 76.7294 | | 47 | | 52 | | 35 | | 50 | |
| 76.7498 | | 46 | | 50 | | 47 | | 53 | |
| 76.7703 | | 44 | | 45 | | 62 | | 62 | |
| 76.7907 | | 53 | | 47 | | 45 | | 52 | |
| 76.8112 | | 56 | | 59 | | 41 | | 54 | |
| 76.8316 | | 43 | | 66 | | 50 | | 44 | |
| 76.8521 | | 56 | | 53 | | 49 | | 54 | |
| 76.8725 | | 54 | | 57 | | 53 | | 41 | |
| 76.893 | | 53 | | 52 | | 43 | | 43 | |
| 76.9134 | | 24 | | 52 | | 54 | | 56 | |
| 76.9339 | | 62 | | 53 | | 47 | | 47 | |
| 76.9543 | | 42 | | 50 | | 54 | | 55 | |
| 76.9748 | | 54 | | 55 | | 54 | | 55 | |
| 76.9952 | | 46 | | 52 | | 55 | | 58 | |
| 77.0157 | | 46 | | 54 | | 58 | | 56 | |
| 77.0361 | | 40 | | 60 | | 53 | | 47 | |
| 77.0566 | | 41 | | 68 | | 53 | | 37 | |
| 77.077 | | 46 | | 62 | | 51 | | 59 | |
| 77.0975 | | 43 | | 65 | | 51 | | 44 | |
| 77.1179 | | 58 | | 71 | | 57 | | 51 | |
| 77.1384 | | 55 | | 56 | | 57 | | 58 | |
| 77.1589 | | 42 | | 62 | | 39 | | 50 | |
| 77.1793 | | 35 | | 55 | | 59 | | 48 | |
| 77.1998 | | 66 | | 62 | | 57 | | 67 | |
| 77.2202 | | 60 | | 43 | | 51 | | 56 | |
| 77.2407 | | 50 | | 63 | | 64 | | 69 | |
| 77.2611 | | 51 | | 54 | | 59 | | 36 | |
| 77.2816 | | 41 | | 65 | | 51 | | 54 | |
| 77.302 | | 62 | | 55 | | 49 | | 53 | |
| 77.3225 | | 56 | | 56 | | 70 | | 61 | |
| 77.3429 | | 54 | | 64 | | 58 | | 61 | |
| 77.3634 | | 59 | | 54 | | 72 | | 65 | |
| 77.3838 | | 69 | | 52 | | 75 | | 52 | |
| 77.4043 | | 58 | | 68 | | 58 | | 60 | |
| 77.4247 | | 43 | | 76 | | 66 | | 67 | |
| 77.4452 | | 62 | | 66 | | 73 | | 76 | |
| 77.4656 | | 71 | | 82 | | 70 | | 78 | |
| 77.4861 | | 71 | | 79 | | 86 | | 79 | |
| 77.5065 | | 66 | | 97 | | 74 | | 83 | |
| 77.527 | | 99 | | 87 | | 75 | | 99 | |
| 77.5474 | | 107 | | 109 | | 116 | | 114 | |
| 77.5679 | | 135 | | 124 | | 121 | | 131 | |
| 77.5883 | | 196 | | 177 | | 187 | | 200 | |
| 77.6088 | | 251 | | 265 | | 261 | | 267 | |
| 77.6292 | | 413 | | 404 | | 389 | | 340 | |
| 77.6497 | | 535 | | 532 | | 531 | | 415 | |
| 77.6701 | | 486 | | 491 | | 418 | | 408 | |
| 77.6906 | | 262 | | 304 | | 232 | | 259 | |
| 77.7111 | | 170 | | 205 | | 160 | | 187 | |
| 77.7315 | | 110 | | 138 | | 124 | | 128 | |
| 77.752 | | 132 | | 96 | | 97 | | 111 | |
| 77.7724 | | 107 | | 94 | | 97 | | 109 | |
| 77.7929 | | 89 | | 100 | | 104 | | 111 | |
| 77.8133 | | 127 | | 122 | | 133 | | 158 | |
| 77.8338 | | 175 | | 150 | | 157 | | 184 | |
| 77.8542 | | 263 | | 219 | | 194 | | 213 | |
| 77.8747 | | 253 | | 306 | | 255 | | 250 | |
| 77.8951 | | 276 | | 271 | | 251 | | 222 | |
| 77.9156 | | 185 | | 190 | | 182 | | 178 | |
| 77.936 | | 127 | | 124 | | 120 | | 124 | |
| 77.9565 | | 69 | | 103 | | 95 | | 88 | |
| 77.9769 | | 72 | | 67 | | 79 | | 89 | |
| 77.9974 | | 60 | | 76 | | 70 | | 84 | |
| 78.0178 | | 59 | | 58 | | 51 | | 60 | |
| 78.0383 | | 43 | | 55 | | 58 | | 77 | |
| 78.0587 | | 51 | | 55 | | 48 | | 50 | |
| 78.0792 | | 64 | | 44 | | 60 | | 49 | |
| 78.0996 | | 54 | | 54 | | 58 | | 52 | |
| 78.1201 | | 50 | | 64 | | 58 | | 54 | |
| 78.1405 | | 52 | | 56 | | 56 | | 49 | |
| 78.161 | | 55 | | 52 | | 44 | | 55 | |
| 78.1814 | | 52 | | 53 | | 53 | | 52 | |
| 78.2019 | | 45 | | 50 | | 50 | | 55 | |
| 78.2224 | | 52 | | 65 | | 56 | | 49 | |
| 78.2428 | | 49 | | 55 | | 62 | | 46 | |
| 78.2633 | | 60 | | 50 | | 53 | | 57 | |
| 78.2837 | | 60 | | 56 | | 44 | | 58 | |
| 78.3042 | | 49 | | 61 | | 39 | | 41 | |
| 78.3246 | | 35 | | 62 | | 46 | | 49 | |
| 78.3451 | | 44 | | 46 | | 58 | | 51 | |
| 78.3655 | | 47 | | 56 | | 54 | | 56 | |
| 78.386 | | 51 | | 43 | | 58 | | 71 | |
| 78.4064 | | 46 | | 55 | | 53 | | 57 | |
| 78.4269 | | 51 | | 46 | | 51 | | 47 | |
| 78.4473 | | 49 | | 44 | | 47 | | 45 | |
| 78.4678 | | 38 | | 65 | | 62 | | 59 | |
| 78.4882 | | 47 | | 47 | | 51 | | 41 | |
| 78.5087 | | 41 | | 46 | | 51 | | 45 | |
| 78.5291 | | 44 | | 54 | | 51 | | 55 | |
| 78.5496 | | 61 | | 32 | | 52 | | 55 | |
| 78.57 | | 34 | | 46 | | 50 | | 50 | |
| 78.5905 | | 43 | | 39 | | 46 | | 69 | |
| 78.6109 | | 37 | | 43 | | 47 | | 46 | |
| 78.6314 | | 47 | | 43 | | 48 | | 53 | |
| 78.6518 | | 53 | | 47 | | 43 | | 42 | |
| 78.6723 | | 54 | | 49 | | 41 | | 40 | |
| 78.6927 | | 57 | | 52 | | 36 | | 45 | |
| 78.7132 | | 47 | | 43 | | 43 | | 37 | |
| 78.7337 | | 52 | | 60 | | 49 | | 42 | |
| 78.7541 | | 39 | | 50 | | 51 | | 48 | |
| 78.7746 | | 47 | | 44 | | 52 | | 47 | |
| 78.795 | | 45 | | 55 | | 49 | | 34 | |
| 78.8155 | | 44 | | 43 | | 51 | | 56 | |
| 78.8359 | | 41 | | 60 | | 59 | | 53 | |
| 78.8564 | | 51 | | 57 | | 73 | | 39 | |
| 78.8768 | | 34 | | 54 | | 52 | | 47 | |
| 78.8973 | | 57 | | 55 | | 46 | | 45 | |
| 78.9177 | | 44 | | 50 | | 49 | | 45 | |
| 78.9382 | | 50 | | 48 | | 58 | | 54 | |
| 78.9586 | | 43 | | 55 | | 55 | | 52 | |
| 78.9791 | | 46 | | 37 | | 55 | | 54 | |
| 78.9995 | | 42 | | 47 | | 45 | | 33 | |
| 79.02 | | 56 | | 49 | | 64 | | 47 | |
| 79.0404 | | 47 | | 48 | | 53 | | 59 | |
| 79.0609 | | 52 | | 49 | | 32 | | 54 | |
| 79.0813 | | 57 | | 52 | | 49 | | 50 | |
| 79.1018 | | 37 | | 46 | | 60 | | 46 | |
| 79.1222 | | 49 | | 55 | | 54 | | 57 | |
| 79.1427 | | 50 | | 51 | | 46 | | 45 | |
| 79.1631 | | 52 | | 49 | | 62 | | 47 | |
| 79.1836 | | 43 | | 57 | | 52 | | 39 | |
| 79.204 | | 43 | | 58 | | 68 | | 61 | |
| 79.2245 | | 63 | | 47 | | 42 | | 54 | |
| 79.2449 | | 45 | | 55 | | 66 | | 62 | |
| 79.2654 | | 43 | | 60 | | 48 | | 44 | |
| 79.2859 | | 43 | | 60 | | 58 | | 43 | |
| 79.3063 | | 54 | | 49 | | 57 | | 55 | |
| 79.3268 | | 42 | | 42 | | 64 | | 52 | |
| 79.3472 | | 57 | | 66 | | 52 | | 40 | |
| 79.3677 | | 53 | | 73 | | 52 | | 56 | |
| 79.3881 | | 46 | | 46 | | 58 | | 58 | |
| 79.4086 | | 55 | | 72 | | 63 | | 60 | |
| 79.429 | | 59 | | 50 | | 63 | | 68 | |
| 79.4495 | | 74 | | 52 | | 64 | | 57 | |
| 79.4699 | | 71 | | 65 | | 79 | | 70 | |
| 79.4904 | | 52 | | 59 | | 62 | | 83 | |
| 79.5108 | | 59 | | 66 | | 49 | | 70 | |
| 79.5313 | | 67 | | 72 | | 59 | | 60 | |
| 79.5517 | | 63 | | 66 | | 55 | | 57 | |
| 79.5722 | | 70 | | 65 | | 73 | | 74 | |
| 79.5926 | | 58 | | 62 | | 63 | | 69 | |
| 79.6131 | | 77 | | 84 | | 62 | | 71 | |
| 79.6335 | | 77 | | 94 | | 74 | | 87 | |
| 79.654 | | 72 | | 81 | | 82 | | 76 | |
| 79.6744 | | 82 | | 84 | | 85 | | 79 | |
| 79.6949 | | 89 | | 94 | | 92 | | 126 | |
| 79.7153 | | 99 | | 116 | | 96 | | 107 | |
| 79.7358 | | 116 | | 112 | | 123 | | 141 | |
| 79.7562 | | 143 | | 141 | | 142 | | 160 | |
| 79.7767 | | 199 | | 177 | | 195 | | 204 | |
| 79.7972 | | 311 | | 288 | | 275 | | 335 | |
| 79.8176 | | 446 | | 387 | | 429 | | 493 | |
| 79.8381 | | 714 | | 634 | | 644 | | 743 | |
| 79.8585 | | 954 | | 892 | | 833 | | 918 | |
| 79.879 | | 860 | | 1018 | | 851 | | 960 | |
| 79.8994 | | 506 | | 636 | | 499 | | 593 | |
| 79.9199 | | 293 | | 375 | | 308 | | 396 | |
| 79.9403 | | 239 | | 281 | | 269 | | 312 | |
| 79.9608 | | 220 | | 235 | | 221 | | 266 | |
| 79.9812 | | 294 | | 242 | | 254 | | 294 | |
| 80.0017 | | 375 | | 324 | | 363 | | 350 | |
| 80.0221 | | 489 | | 363 | | 445 | | 424 | |
| 80.0426 | | 459 | | 417 | | 455 | | 417 | |
| 80.063 | | 456 | | 362 | | 470 | | 444 | |
| 80.0835 | | 496 | | 489 | | 488 | | 521 | |
| 80.1039 | | 587 | | 563 | | 530 | | 567 | |
| 80.1244 | | 441 | | 552 | | 423 | | 471 | |
| 80.1448 | | 264 | | 345 | | 262 | | 342 | |
| 80.1653 | | 194 | | 212 | | 166 | | 241 | |
| 80.1857 | | 157 | | 160 | | 141 | | 175 | |
| 80.2062 | | 150 | | 140 | | 172 | | 162 | |
| 80.2266 | | 157 | | 153 | | 180 | | 169 | |
| 80.2471 | | 220 | | 181 | | 206 | | 179 | |
| 80.2675 | | 216 | | 166 | | 204 | | 187 | |
| 80.288 | | 188 | | 139 | | 169 | | 157 | |
| 80.3085 | | 97 | | 140 | | 113 | | 137 | |
| 80.3289 | | 102 | | 88 | | 104 | | 123 | |
| 80.3494 | | 76 | | 73 | | 87 | | 85 | |
| 80.3698 | | 62 | | 60 | | 86 | | 78 | |
| 80.3903 | | 67 | | 66 | | 77 | | 88 | |
| 80.4107 | | 70 | | 69 | | 51 | | 61 | |
| 80.4312 | | 66 | | 62 | | 57 | | 55 | |
| 80.4516 | | 53 | | 81 | | 79 | | 55 | |
| 80.4721 | | 40 | | 66 | | 72 | | 57 | |
| 80.4925 | | 55 | | 60 | | 64 | | 57 | |
| 80.513 | | 53 | | 67 | | 72 | | 61 | |
| 80.5334 | | 49 | | 54 | | 74 | | 54 | |
| 80.5539 | | 54 | | 65 | | 52 | | 56 | |
| 80.5743 | | 44 | | 51 | | 62 | | 41 | |
| 80.5948 | | 55 | | 62 | | 62 | | 52 | |
| 80.6152 | | 56 | | 70 | | 58 | | 68 | |
| 80.6357 | | 60 | | 51 | | 57 | | 48 | |
| 80.6561 | | 57 | | 63 | | 57 | | 60 | |
| 80.6766 | | 50 | | 62 | | 65 | | 58 | |
| 80.697 | | 59 | | 63 | | 77 | | 55 | |
| 80.7175 | | 56 | | 70 | | 50 | | 56 | |
| 80.7379 | | 60 | | 65 | | 55 | | 68 | |
| 80.7584 | | 63 | | 79 | | 58 | | 55 | |
| 80.7788 | | 56 | | 68 | | 62 | | 66 | |
| 80.7993 | | 52 | | 62 | | 57 | | 74 | |
| 80.8197 | | 57 | | 67 | | 56 | | 52 | |
| 80.8402 | | 61 | | 60 | | 55 | | 60 | |
| 80.8607 | | 48 | | 59 | | 71 | | 75 | |
| 80.8811 | | 64 | | 57 | | 59 | | 55 | |
| 80.9016 | | 56 | | 79 | | 69 | | 59 | |
| 80.922 | | 67 | | 77 | | 68 | | 82 | |
| 80.9425 | | 76 | | 89 | | 52 | | 74 | |
| 80.9629 | | 71 | | 101 | | 73 | | 94 | |
| 80.9834 | | 80 | | 89 | | 64 | | 95 | |
| 81.0038 | | 83 | | 111 | | 89 | | 112 | |
| 81.0243 | | 119 | | 110 | | 107 | | 138 | |
| 81.0447 | | 124 | | 145 | | 130 | | 139 | |
| 81.0652 | | 175 | | 191 | | 182 | | 192 | |
| 81.0856 | | 267 | | 237 | | 247 | | 264 | |
| 81.1061 | | 362 | | 308 | | 370 | | 373 | |
| 81.1265 | | 601 | | 450 | | 530 | | 530 | |
| 81.147 | | 763 | | 589 | | 674 | | 679 | |
| 81.1674 | | 587 | | 605 | | 638 | | 635 | |
| 81.1879 | | 330 | | 388 | | 402 | | 412 | |
| 81.2083 | | 196 | | 250 | | 236 | | 263 | |
| 81.2288 | | 165 | | 163 | | 182 | | 201 | |
| 81.2492 | | 142 | | 163 | | 162 | | 202 | |
| 81.2697 | | 123 | | 152 | | 142 | | 177 | |
| 81.2901 | | 125 | | 155 | | 161 | | 184 | |
| 81.3106 | | 193 | | 175 | | 164 | | 185 | |
| 81.331 | | 199 | | 216 | | 211 | | 267 | |
| 81.3515 | | 304 | | 285 | | 304 | | 327 | |
| 81.372 | | 457 | | 419 | | 391 | | 417 | |
| 81.3924 | | 571 | | 511 | | 539 | | 575 | |
| 81.4129 | | 660 | | 561 | | 573 | | 662 | |
| 81.4333 | | 700 | | 634 | | 637 | | 663 | |
| 81.4538 | | 868 | | 774 | | 842 | | 747 | |
| 81.4742 | | 929 | | 844 | | 962 | | 741 | |
| 81.4947 | | 629 | | 727 | | 701 | | 578 | |
| 81.5151 | | 349 | | 419 | | 374 | | 390 | |
| 81.5356 | | 231 | | 239 | | 264 | | 248 | |
| 81.556 | | 152 | | 185 | | 178 | | 210 | |
| 81.5765 | | 149 | | 161 | | 141 | | 157 | |
| 81.5969 | | 119 | | 145 | | 109 | | 140 | |
| 81.6174 | | 158 | | 151 | | 167 | | 149 | |
| 81.6378 | | 158 | | 151 | | 176 | | 221 | |
| 81.6583 | | 236 | | 192 | | 201 | | 203 | |
| 81.6787 | | 264 | | 309 | | 305 | | 275 | |
| 81.6992 | | 454 | | 391 | | 385 | | 358 | |
| 81.7196 | | 487 | | 445 | | 400 | | 374 | |
| 81.7401 | | 364 | | 372 | | 379 | | 332 | |
| 81.7605 | | 182 | | 254 | | 251 | | 221 | |
| 81.781 | | 136 | | 145 | | 164 | | 136 | |
| 81.8014 | | 121 | | 108 | | 116 | | 132 | |
| 81.8219 | | 102 | | 82 | | 83 | | 104 | |
| 81.8423 | | 62 | | 71 | | 72 | | 71 | |
| 81.8628 | | 62 | | 77 | | 67 | | 76 | |
| 81.8833 | | 49 | | 69 | | 69 | | 61 | |
| 81.9037 | | 66 | | 67 | | 54 | | 53 | |
| 81.9242 | | 58 | | 74 | | 53 | | 70 | |
| 81.9446 | | 50 | | 75 | | 56 | | 56 | |
| 81.9651 | | 54 | | 49 | | 62 | | 59 | |
| 81.9855 | | 52 | | 73 | | 68 | | 43 | |
| 82.006 | | 58 | | 49 | | 56 | | 54 | |
| 82.0264 | | 48 | | 53 | | 63 | | 56 | |
| 82.0469 | | 53 | | 59 | | 58 | | 55 | |
| 82.0673 | | 42 | | 55 | | 55 | | 56 | |
| 82.0878 | | 45 | | 60 | | 51 | | 45 | |
| 82.1082 | | 46 | | 40 | | 37 | | 61 | |
| 82.1287 | | 47 | | 57 | | 63 | | 50 | |
| 82.1491 | | 51 | | 56 | | 41 | | 50 | |
| 82.1696 | | 50 | | 46 | | 48 | | 50 | |
| 82.19 | | 43 | | 47 | | 44 | | 46 | |
| 82.2105 | | 49 | | 45 | | 55 | | 48 | |
| 82.2309 | | 50 | | 60 | | 56 | | 47 | |
| 82.2514 | | 41 | | 50 | | 48 | | 40 | |
| 82.2718 | | 42 | | 45 | | 43 | | 47 | |
| 82.2923 | | 48 | | 55 | | 43 | | 36 | |
| 82.3127 | | 43 | | 46 | | 51 | | 43 | |
| 82.3332 | | 38 | | 50 | | 44 | | 45 | |
| 82.3536 | | 29 | | 47 | | 40 | | 45 | |
| 82.3741 | | 45 | | 48 | | 34 | | 45 | |
| 82.3945 | | 52 | | 42 | | 43 | | 48 | |
| 82.415 | | 48 | | 44 | | 47 | | 53 | |
| 82.4355 | | 39 | | 46 | | 40 | | 34 | |
| 82.4559 | | 46 | | 40 | | 40 | | 38 | |
| 82.4764 | | 49 | | 42 | | 48 | | 46 | |
| 82.4968 | | 31 | | 47 | | 46 | | 49 | |
| 82.5173 | | 37 | | 43 | | 41 | | 41 | |
| 82.5377 | | 43 | | 54 | | 51 | | 58 | |
| 82.5582 | | 49 | | 65 | | 56 | | 42 | |
| 82.5786 | | 48 | | 43 | | 50 | | 49 | |
| 82.5991 | | 39 | | 41 | | 45 | | 52 | |
| 82.6195 | | 48 | | 45 | | 45 | | 41 | |
| 82.64 | | 47 | | 52 | | 45 | | 44 | |
| 82.6604 | | 43 | | 37 | | 33 | | 55 | |
| 82.6809 | | 37 | | 33 | | 38 | | 51 | |
| 82.7013 | | 42 | | 55 | | 44 | | 38 | |
| 82.7218 | | 36 | | 41 | | 59 | | 48 | |
| 82.7422 | | 46 | | 48 | | 52 | | 43 | |
| 82.7627 | | 56 | | 49 | | 52 | | 43 | |
| 82.7831 | | 39 | | 41 | | 28 | | 37 | |
| 82.8036 | | 42 | | 33 | | 41 | | 35 | |
| 82.824 | | 41 | | 45 | | 41 | | 41 | |
| 82.8445 | | 44 | | 45 | | 47 | | 54 | |
| 82.8649 | | 40 | | 40 | | 49 | | 41 | |
| 82.8854 | | 35 | | 35 | | 53 | | 50 | |
| 82.9058 | | 43 | | 51 | | 48 | | 41 | |
| 82.9263 | | 47 | | 42 | | 36 | | 41 | |
| 82.9468 | | 51 | | 58 | | 31 | | 56 | |
| 82.9672 | | 38 | | 33 | | 33 | | 40 | |
| 82.9877 | | 49 | | 54 | | 39 | | 40 | |
| 83.0081 | | 43 | | 41 | | 38 | | 41 | |
| 83.0286 | | 40 | | 49 | | 40 | | 44 | |
| 83.049 | | 45 | | 58 | | 41 | | 47 | |
| 83.0695 | | 40 | | 35 | | 39 | | 47 | |
| 83.0899 | | 43 | | 50 | | 51 | | 47 | |
| 83.1104 | | 41 | | 54 | | 48 | | 52 | |
| 83.1308 | | 45 | | 38 | | 39 | | 44 | |
| 83.1513 | | 35 | | 47 | | 58 | | 41 | |
| 83.1717 | | 47 | | 46 | | 52 | | 50 | |
| 83.1922 | | 46 | | 43 | | 47 | | 57 | |
| 83.2126 | | 42 | | 54 | | 41 | | 47 | |
| 83.2331 | | 47 | | 49 | | 50 | | 39 | |
| 83.2535 | | 50 | | 52 | | 52 | | 53 | |
| 83.274 | | 55 | | 55 | | 52 | | 35 | |
| 83.2944 | | 51 | | 60 | | 61 | | 40 | |
| 83.3149 | | 52 | | 45 | | 53 | | 60 | |
| 83.3353 | | 37 | | 55 | | 39 | | 56 | |
| 83.3558 | | 43 | | 54 | | 59 | | 43 | |
| 83.3762 | | 43 | | 54 | | 48 | | 38 | |
| 83.3967 | | 38 | | 56 | | 58 | | 42 | |
| 83.4171 | | 43 | | 55 | | 47 | | 51 | |
| 83.4376 | | 50 | | 43 | | 48 | | 41 | |
| 83.458 | | 42 | | 61 | | 50 | | 49 | |
| 83.4785 | | 43 | | 49 | | 51 | | 49 | |
| 83.499 | | 40 | | 59 | | 49 | | 56 | |
| 83.5194 | | 54 | | 62 | | 42 | | 46 | |
| 83.5399 | | 51 | | 63 | | 55 | | 46 | |
| 83.5603 | | 50 | | 56 | | 52 | | 56 | |
| 83.5808 | | 66 | | 86 | | 64 | | 51 | |
| 83.6012 | | 66 | | 66 | | 53 | | 61 | |
| 83.6217 | | 63 | | 60 | | 66 | | 67 | |
| 83.6421 | | 68 | | 62 | | 69 | | 92 | |
| 83.6626 | | 71 | | 73 | | 93 | | 94 | |
| 83.683 | | 78 | | 86 | | 79 | | 90 | |
| 83.7035 | | 112 | | 124 | | 96 | | 98 | |
| 83.7239 | | 120 | | 121 | | 142 | | 159 | |
| 83.7444 | | 178 | | 165 | | 167 | | 190 | |
| 83.7648 | | 244 | | 273 | | 225 | | 268 | |
| 83.7853 | | 420 | | 337 | | 373 | | 357 | |
| 83.8057 | | 518 | | 486 | | 546 | | 477 | |
| 83.8262 | | 603 | | 642 | | 582 | | 475 | |
| 83.8466 | | 367 | | 477 | | 437 | | 407 | |
| 83.8671 | | 217 | | 255 | | 259 | | 248 | |
| 83.8875 | | 134 | | 180 | | 174 | | 190 | |
| 83.908 | | 111 | | 124 | | 118 | | 129 | |
| 83.9284 | | 79 | | 117 | | 93 | | 116 | |
| 83.9489 | | 81 | | 85 | | 97 | | 111 | |
| 83.9693 | | 85 | | 96 | | 107 | | 111 | |
| 83.9898 | | 91 | | 123 | | 109 | | 125 | |
| 84.0103 | | 135 | | 141 | | 131 | | 150 | |
| 84.0307 | | 175 | | 194 | | 196 | | 192 | |
| 84.0512 | | 269 | | 234 | | 265 | | 268 | |
| 84.0716 | | 298 | | 314 | | 332 | | 258 | |
| 84.0921 | | 210 | | 316 | | 302 | | 253 | |
| 84.1125 | | 168 | | 211 | | 186 | | 218 | |
| 84.133 | | 94 | | 155 | | 115 | | 144 | |
| 84.1534 | | 97 | | 91 | | 98 | | 106 | |
| 84.1739 | | 73 | | 87 | | 68 | | 75 | |
| 84.1943 | | 62 | | 68 | | 55 | | 60 | |
| 84.2148 | | 49 | | 58 | | 59 | | 48 | |
| 84.2352 | | 47 | | 53 | | 46 | | 61 | |
| 84.2557 | | 48 | | 53 | | 44 | | 46 | |
| 84.2761 | | 44 | | 64 | | 63 | | 54 | |
| 84.2966 | | 32 | | 49 | | 48 | | 48 | |
| 84.317 | | 50 | | 40 | | 37 | | 41 | |
| 84.3375 | | 34 | | 43 | | 48 | | 46 | |
| 84.3579 | | 60 | | 42 | | 37 | | 52 | |
| 84.3784 | | 48 | | 48 | | 41 | | 45 | |
| 84.3988 | | 43 | | 48 | | 42 | | 39 | |
| 84.4193 | | 43 | | 45 | | 58 | | 46 | |
| 84.4397 | | 35 | | 49 | | 38 | | 36 | |
| 84.4602 | | 53 | | 52 | | 48 | | 50 | |
| 84.4806 | | 45 | | 59 | | 54 | | 40 | |
| 84.5011 | | 44 | | 48 | | 38 | | 41 | |
| 84.5216 | | 46 | | 48 | | 60 | | 46 | |
| 84.542 | | 41 | | 49 | | 46 | | 52 | |
| 84.5625 | | 50 | | 43 | | 42 | | 48 | |
| 84.5829 | | 47 | | 42 | | 42 | | 31 | |
| 84.6034 | | 48 | | 46 | | 40 | | 50 | |
| 84.6238 | | 40 | | 31 | | 40 | | 45 | |
| 84.6443 | | 39 | | 38 | | 34 | | 27 | |
| 84.6647 | | 37 | | 48 | | 35 | | 48 | |
| 84.6852 | | 35 | | 32 | | 48 | | 38 | |
| 84.7056 | | 41 | | 41 | | 51 | | 41 | |
| 84.7261 | | 48 | | 44 | | 47 | | 42 | |
| 84.7465 | | 42 | | 45 | | 43 | | 45 | |
| 84.767 | | 34 | | 46 | | 52 | | 43 | |
| 84.7874 | | 40 | | 51 | | 41 | | 48 | |
| 84.8079 | | 35 | | 45 | | 44 | | 44 | |
| 84.8283 | | 45 | | 55 | | 47 | | 55 | |
| 84.8488 | | 56 | | 32 | | 52 | | 61 | |
| 84.8692 | | 58 | | 55 | | 53 | | 56 | |
| 84.8897 | | 71 | | 68 | | 71 | | 67 | |
| 84.9101 | | 93 | | 91 | | 83 | | 87 | |
| 84.9306 | | 107 | | 105 | | 96 | | 107 | |
| 84.951 | | 97 | | 102 | | 85 | | 103 | |
| 84.9715 | | 72 | | 86 | | 74 | | 69 | |
| 84.9919 | | 44 | | 59 | | 52 | | 52 | |
| 85.0124 | | 44 | | 46 | | 42 | | 50 | |
| 85.0328 | | 44 | | 45 | | 49 | | 40 | |
| 85.0533 | | 50 | | 55 | | 60 | | 39 | |
| 85.0738 | | 35 | | 43 | | 41 | | 50 | |
| 85.0942 | | 43 | | 55 | | 48 | | 48 | |
| 85.1147 | | 44 | | 52 | | 45 | | 47 | |
| 85.1351 | | 42 | | 62 | | 57 | | 35 | |
| 85.1556 | | 51 | | 54 | | 56 | | 60 | |
| 85.176 | | 74 | | 60 | | 45 | | 70 | |
| 85.1965 | | 60 | | 76 | | 73 | | 61 | |
| 85.2169 | | 62 | | 75 | | 56 | | 51 | |
| 85.2374 | | 37 | | 53 | | 43 | | 49 | |
| 85.2578 | | 56 | | 61 | | 53 | | 47 | |
| 85.2783 | | 47 | | 38 | | 52 | | 42 | |
| 85.2987 | | 36 | | 43 | | 45 | | 44 | |
| 85.3192 | | 30 | | 38 | | 37 | | 41 | |
| 85.3396 | | 41 | | 38 | | 38 | | 31 | |
| 85.3601 | | 41 | | 43 | | 47 | | 31 | |
| 85.3805 | | 34 | | 32 | | 29 | | 41 | |
| 85.401 | | 29 | | 33 | | 33 | | 45 | |
| 85.4214 | | 33 | | 55 | | 50 | | 50 | |
| 85.4419 | | 40 | | 40 | | 51 | | 25 | |
| 85.4623 | | 44 | | 28 | | 32 | | 36 | |
| 85.4828 | | 40 | | 48 | | 37 | | 39 | |
| 85.5032 | | 24 | | 27 | | 37 | | 35 | |
| 85.5237 | | 33 | | 41 | | 44 | | 36 | |
| 85.5441 | | 31 | | 30 | | 32 | | 40 | |
| 85.5646 | | 32 | | 39 | | 40 | | 45 | |
| 85.5851 | | 38 | | 44 | | 36 | | 34 | |
| 85.6055 | | 40 | | 36 | | 39 | | 33 | |
| 85.626 | | 22 | | 31 | | 41 | | 42 | |
| 85.6464 | | 27 | | 30 | | 33 | | 37 | |
| 85.6669 | | 44 | | 40 | | 33 | | 39 | |
| 85.6873 | | 37 | | 38 | | 22 | | 35 | |
| 85.7078 | | 38 | | 37 | | 54 | | 44 | |
| 85.7282 | | 33 | | 44 | | 31 | | 30 | |
| 85.7487 | | 36 | | 34 | | 35 | | 28 | |
| 85.7691 | | 40 | | 43 | | 25 | | 45 | |
| 85.7896 | | 31 | | 37 | | 31 | | 35 | |
| 85.81 | | 30 | | 46 | | 41 | | 39 | |
| 85.8305 | | 25 | | 41 | | 42 | | 27 | |
| 85.8509 | | 31 | | 39 | | 31 | | 38 | |
| 85.8714 | | 28 | | 36 | | 44 | | 35 | |
| 85.8918 | | 41 | | 27 | | 42 | | 39 | |
| 85.9123 | | 37 | | 46 | | 39 | | 38 | |
| 85.9327 | | 31 | | 44 | | 39 | | 31 | |
| 85.9532 | | 43 | | 39 | | 52 | | 35 | |
| 85.9736 | | 43 | | 42 | | 24 | | 30 | |
| 85.9941 | | 37 | | 30 | | 26 | | 25 | |
| 86.0145 | | 32 | | 34 | | 55 | | 30 | |
| 86.035 | | 41 | | 30 | | 39 | | 36 | |
| 86.0554 | | 35 | | 39 | | 31 | | 27 | |
| 86.0759 | | 38 | | 43 | | 36 | | 32 | |
| 86.0964 | | 32 | | 36 | | 30 | | 40 | |
| 86.1168 | | 42 | | 43 | | 42 | | 37 | |
| 86.1373 | | 31 | | 36 | | 31 | | 37 | |
| 86.1577 | | 34 | | 33 | | 24 | | 29 | |
| 86.1782 | | 26 | | 36 | | 37 | | 29 | |
| 86.1986 | | 30 | | 33 | | 29 | | 35 | |
| 86.2191 | | 37 | | 34 | | 27 | | 41 | |
| 86.2395 | | 36 | | 37 | | 28 | | 35 | |
| 86.26 | | 25 | | 38 | | 31 | | 44 | |
| 86.2804 | | 42 | | 33 | | 41 | | 33 | |
| 86.3009 | | 39 | | 40 | | 34 | | 30 | |
| 86.3213 | | 44 | | 35 | | 29 | | 36 | |
| 86.3418 | | 35 | | 43 | | 27 | | 43 | |
| 86.3622 | | 32 | | 35 | | 26 | | 35 | |
| 86.3827 | | 27 | | 37 | | 34 | | 41 | |
| 86.4031 | | 29 | | 33 | | 26 | | 47 | |
| 86.4236 | | 32 | | 24 | | 27 | | 35 | |
| 86.444 | | 32 | | 40 | | 38 | | 46 | |
| 86.4645 | | 31 | | 39 | | 38 | | 39 | |
| 86.4849 | | 30 | | 49 | | 43 | | 45 | |
| 86.5054 | | 29 | | 30 | | 29 | | 37 | |
| 86.5258 | | 31 | | 42 | | 29 | | 42 | |
| 86.5463 | | 51 | | 50 | | 37 | | 40 | |
| 86.5667 | | 39 | | 37 | | 52 | | 37 | |
| 86.5872 | | 44 | | 42 | | 39 | | 33 | |
| 86.6076 | | 31 | | 47 | | 34 | | 45 | |
| 86.6281 | | 46 | | 34 | | 37 | | 48 | |
| 86.6486 | | 42 | | 29 | | 40 | | 41 | |
| 86.669 | | 35 | | 30 | | 39 | | 44 | |
| 86.6895 | | 34 | | 55 | | 44 | | 41 | |
| 86.7099 | | 40 | | 29 | | 39 | | 35 | |
| 86.7304 | | 35 | | 46 | | 34 | | 32 | |
| 86.7508 | | 27 | | 35 | | 32 | | 28 | |
| 86.7713 | | 35 | | 38 | | 25 | | 37 | |
| 86.7917 | | 38 | | 35 | | 40 | | 31 | |
| 86.8122 | | 36 | | 34 | | 42 | | 27 | |
| 86.8326 | | 30 | | 41 | | 34 | | 27 | |
| 86.8531 | | 33 | | 44 | | 35 | | 45 | |
| 86.8735 | | 36 | | 29 | | 37 | | 33 | |
| 86.894 | | 37 | | 32 | | 35 | | 33 | |
| 86.9144 | | 37 | | 36 | | 30 | | 28 | |
| 86.9349 | | 37 | | 29 | | 25 | | 39 | |
| 86.9553 | | 39 | | 37 | | 44 | | 40 | |
| 86.9758 | | 38 | | 40 | | 46 | | 34 | |
| 86.9962 | | 38 | | 46 | | 35 | | 35 | |
| 87.0167 | | 41 | | 42 | | 33 | | 35 | |
| 87.0371 | | 31 | | 47 | | 35 | | 39 | |
| 87.0576 | | 45 | | 41 | | 37 | | 48 | |
| 87.078 | | 33 | | 32 | | 29 | | 45 | |
| 87.0985 | | 34 | | 44 | | 49 | | 33 | |
| 87.1189 | | 33 | | 31 | | 37 | | 45 | |
| 87.1394 | | 34 | | 40 | | 39 | | 39 | |
| 87.1599 | | 33 | | 27 | | 49 | | 33 | |
| 87.1803 | | 33 | | 39 | | 46 | | 25 | |
| 87.2008 | | 34 | | 40 | | 42 | | 28 | |
| 87.2212 | | 41 | | 31 | | 39 | | 47 | |
| 87.2417 | | 44 | | 29 | | 45 | | 29 | |
| 87.2621 | | 29 | | 48 | | 40 | | 42 | |
| 87.2826 | | 31 | | 45 | | 35 | | 43 | |
| 87.303 | | 65 | | 42 | | 46 | | 42 | |
| 87.3235 | | 49 | | 46 | | 42 | | 61 | |
| 87.3439 | | 52 | | 48 | | 56 | | 66 | |
| 87.3644 | | 54 | | 67 | | 76 | | 57 | |
| 87.3848 | | 94 | | 56 | | 78 | | 93 | |
| 87.4053 | | 114 | | 81 | | 88 | | 89 | |
| 87.4257 | | 124 | | 112 | | 109 | | 71 | |
| 87.4462 | | 112 | | 102 | | 97 | | 79 | |
| 87.4666 | | 63 | | 73 | | 65 | | 70 | |
| 87.4871 | | 51 | | 53 | | 52 | | 59 | |
| 87.5075 | | 36 | | 46 | | 45 | | 47 | |
| 87.528 | | 58 | | 45 | | 42 | | 43 | |
| 87.5484 | | 44 | | 22 | | 38 | | 48 | |
| 87.5689 | | 37 | | 32 | | 43 | | 34 | |
| 87.5893 | | 33 | | 52 | | 37 | | 39 | |
| 87.6098 | | 53 | | 38 | | 47 | | 42 | |
| 87.6302 | | 44 | | 49 | | 45 | | 33 | |
| 87.6507 | | 54 | | 68 | | 47 | | 54 | |
| 87.6712 | | 65 | | 52 | | 46 | | 42 | |
| 87.6916 | | 74 | | 71 | | 61 | | 69 | |
| 87.7121 | | 75 | | 80 | | 77 | | 61 | |
| 87.7325 | | 54 | | 62 | | 52 | | 39 | |
| 87.753 | | 61 | | 54 | | 56 | | 39 | |
| 87.7734 | | 39 | | 34 | | 48 | | 36 | |
| 87.7939 | | 32 | | 40 | | 36 | | 35 | |
| 87.8143 | | 33 | | 36 | | 35 | | 36 | |
| 87.8348 | | 37 | | 38 | | 40 | | 37 | |
| 87.8552 | | 36 | | 30 | | 42 | | 31 | |
| 87.8757 | | 27 | | 41 | | 34 | | 35 | |
| 87.8961 | | 33 | | 41 | | 40 | | 40 | |
| 87.9166 | | 45 | | 38 | | 46 | | 33 | |
| 87.937 | | 31 | | 35 | | 33 | | 34 | |
| 87.9575 | | 39 | | 28 | | 31 | | 31 | |
| 87.9779 | | 24 | | 38 | | 34 | | 39 | |
| 87.9984 | | 28 | | 28 | | 25 | | 44 | |
| 88.0188 | | 31 | | 29 | | 28 | | 34 | |
| 88.0393 | | 43 | | 30 | | 42 | | 41 | |
| 88.0597 | | 23 | | 30 | | 31 | | 31 | |
| 88.0802 | | 34 | | 39 | | 41 | | 40 | |
| 88.1006 | | 42 | | 41 | | 35 | | 33 | |
| 88.1211 | | 38 | | 27 | | 41 | | 37 | |
| 88.1415 | | 38 | | 39 | | 44 | | 38 | |
| 88.162 | | 31 | | 25 | | 40 | | 24 | |
| 88.1824 | | 32 | | 30 | | 29 | | 31 | |
| 88.2029 | | 33 | | 33 | | 32 | | 27 | |
| 88.2234 | | 35 | | 34 | | 43 | | 39 | |
| 88.2438 | | 25 | | 34 | | 36 | | 35 | |
| 88.2643 | | 37 | | 37 | | 26 | | 50 | |
| 88.2847 | | 40 | | 39 | | 36 | | 28 | |
| 88.3052 | | 26 | | 36 | | 43 | | 38 | |
| 88.3256 | | 41 | | 41 | | 30 | | 34 | |
| 88.3461 | | 34 | | 40 | | 51 | | 27 | |
| 88.3665 | | 24 | | 36 | | 37 | | 29 | |
| 88.387 | | 35 | | 23 | | 29 | | 40 | |
| 88.4074 | | 27 | | 34 | | 39 | | 35 | |
| 88.4279 | | 35 | | 42 | | 29 | | 34 | |
| 88.4483 | | 40 | | 44 | | 28 | | 27 | |
| 88.4688 | | 30 | | 23 | | 33 | | 27 | |
| 88.4892 | | 38 | | 27 | | 33 | | 31 | |
| 88.5097 | | 42 | | 28 | | 40 | | 38 | |
| 88.5301 | | 36 | | 46 | | 31 | | 34 | |
| 88.5506 | | 41 | | 36 | | 37 | | 41 | |
| 88.571 | | 26 | | 26 | | 46 | | 33 | |
| 88.5915 | | 36 | | 31 | | 40 | | 27 | |
| 88.6119 | | 39 | | 30 | | 41 | | 31 | |
| 88.6324 | | 27 | | 22 | | 37 | | 23 | |
| 88.6528 | | 31 | | 42 | | 37 | | 35 | |
| 88.6733 | | 30 | | 42 | | 33 | | 41 | |
| 88.6937 | | 40 | | 35 | | 29 | | 33 | |
| 88.7142 | | 24 | | 35 | | 34 | | 34 | |
| 88.7347 | | 24 | | 33 | | 30 | | 34 | |
| 88.7551 | | 30 | | 39 | | 30 | | 35 | |
| 88.7756 | | 26 | | 38 | | 32 | | 34 | |
| 88.796 | | 29 | | 28 | | 44 | | 23 | |
| 88.8165 | | 35 | | 40 | | 29 | | 42 | |
| 88.8369 | | 28 | | 43 | | 32 | | 31 | |
| 88.8574 | | 38 | | 42 | | 27 | | 28 | |
| 88.8778 | | 34 | | 36 | | 32 | | 42 | |
| 88.8983 | | 37 | | 31 | | 28 | | 40 | |
| 88.9187 | | 34 | | 25 | | 29 | | 36 | |
| 88.9392 | | 37 | | 35 | | 36 | | 34 | |
| 88.9596 | | 25 | | 35 | | 40 | | 37 | |
| 88.9801 | | 38 | | 34 | | 32 | | 43 | |
| 89.0005 | | 34 | | 34 | | 30 | | 29 | |
| 89.021 | | 35 | | 44 | | 28 | | 36 | |
| 89.0414 | | 29 | | 32 | | 36 | | 34 | |
| 89.0619 | | 37 | | 34 | | 28 | | 27 | |
| 89.0823 | | 34 | | 35 | | 38 | | 32 | |
| 89.1028 | | 32 | | 32 | | 41 | | 30 | |
| 89.1232 | | 23 | | 31 | | 38 | | 31 | |
| 89.1437 | | 33 | | 31 | | 25 | | 25 | |
| 89.1641 | | 44 | | 39 | | 45 | | 35 | |
| 89.1846 | | 34 | | 25 | | 32 | | 28 | |
| 89.205 | | 35 | | 34 | | 50 | | 31 | |
| 89.2255 | | 36 | | 35 | | 25 | | 19 | |
| 89.246 | | 38 | | 43 | | 45 | | 34 | |
| 89.2664 | | 36 | | 40 | | 26 | | 29 | |
| 89.2869 | | 25 | | 25 | | 43 | | 47 | |
| 89.3073 | | 35 | | 34 | | 34 | | 38 | |
| 89.3278 | | 33 | | 35 | | 29 | | 30 | |
| 89.3482 | | 36 | | 38 | | 45 | | 34 | |
| 89.3687 | | 42 | | 42 | | 44 | | 38 | |
| 89.3891 | | 37 | | 44 | | 43 | | 28 | |
| 89.4096 | | 37 | | 42 | | 31 | | 32 | |
| 89.43 | | 33 | | 33 | | 37 | | 31 | |
| 89.4505 | | 33 | | 42 | | 34 | | 41 | |
| 89.4709 | | 32 | | 39 | | 37 | | 33 | |
| 89.4914 | | 33 | | 49 | | 43 | | 33 | |
| 89.5118 | | 37 | | 30 | | 42 | | 25 | |
| 89.5323 | | 33 | | 34 | | 37 | | 38 | |
| 89.5527 | | 34 | | 35 | | 37 | | 21 | |
| 89.5732 | | 33 | | 42 | | 35 | | 38 | |
| 89.5936 | | 34 | | 47 | | 35 | | 43 | |
| 89.6141 | | 35 | | 44 | | 32 | | 47 | |
| 89.6345 | | 32 | | 41 | | 37 | | 42 | |
| 89.655 | | 42 | | 47 | | 38 | | 28 | |
| 89.6754 | | 44 | | 38 | | 34 | | 43 | |
| 89.6959 | | 42 | | 47 | | 38 | | 42 | |
| 89.7163 | | 24 | | 39 | | 36 | | 35 | |
| 89.7368 | | 32 | | 31 | | 38 | | 29 | |
| 89.7572 | | 36 | | 39 | | 34 | | 37 | |
| 89.7777 | | 25 | | 33 | | 33 | | 27 | |
| 89.7982 | | 29 | | 40 | | 34 | | 40 | |
| 89.8186 | | 38 | | 31 | | 44 | | 40 | |
| 89.8391 | | 22 | | 35 | | 36 | | 34 | |
| 89.8595 | | 31 | | 39 | | 36 | | 28 | |
| 89.88 | | 43 | | 44 | | 30 | | 30 | |
| 89.9004 | | 34 | | 47 | | 38 | | 39 | |
| 89.9209 | | 36 | | 35 | | 41 | | 30 | |
| 89.9413 | | 34 | | 31 | | 49 | | 35 | |
| 89.9618 | | 37 | | 46 | | 37 | | 41 | |
| 89.9822 | | 44 | | 40 | | 43 | | 28 | |
| 90.0027 | | 34 | | 46 | | 31 | | 46 | |
| **UV-Vis Spectrum data of fig.3b** | | | | | | | | |  |
| color parameters | untreated | | 380℃ | | 400℃ | | 420℃ | |  |
| L^*^ | 52.04 | | 53.09 | | 65.56 | | 83.38 | |  |
| a^*^ | 28.89 | | 26.69 | | 16.5 | | 0.05 | |  |
| b^*^ | -26.7 | | -23.53 | | -14.33 | | 2.01 | |  |
| wavelength(nm) | untreated | | 380℃ | | 400℃ | | 420℃ | |  |
| 200 | 0.99295 | | 1.08525 | | 1.19012 | | 0.83642 | |  |
| 200.5 | 0.99678 | | 1.10077 | | 1.1947 | | 0.84011 | |  |
| 201 | 1.00062 | | 1.11503 | | 1.19882 | | 0.84393 | |  |
| 201.5 | 1.00446 | | 1.12803 | | 1.20249 | | 0.84789 | |  |
| 202 | 1.00832 | | 1.13977 | | 1.20571 | | 0.85196 | |  |
| 202.5 | 1.01218 | | 1.15025 | | 1.20847 | | 0.85617 | |  |
| 203 | 1.01606 | | 1.15948 | | 1.21078 | | 0.8605 | |  |
| 203.5 | 1.01994 | | 1.16744 | | 1.21263 | | 0.86496 | |  |
| 204 | 1.02384 | | 1.17415 | | 1.21403 | | 0.86954 | |  |
| 204.5 | 1.02774 | | 1.1796 | | 1.21498 | | 0.87426 | |  |
| 205 | 1.03165 | | 1.18378 | | 1.21547 | | 0.8791 | |  |
| 205.5 | 1.03693 | | 1.18244 | | 1.20835 | | 0.88495 | |  |
| 206 | 1.04223 | | 1.18034 | | 1.20575 | | 0.89048 | |  |
| 206.5 | 1.04493 | | 1.1807 | | 1.20394 | | 0.89453 | |  |
| 207 | 1.05034 | | 1.18047 | | 1.21116 | | 0.90004 | |  |
| 207.5 | 1.05434 | | 1.18276 | | 1.21059 | | 0.90388 | |  |
| 208 | 1.05693 | | 1.18169 | | 1.2072 | | 0.90904 | |  |
| 208.5 | 1.05723 | | 1.17728 | | 1.20393 | | 0.91422 | |  |
| 209 | 1.05782 | | 1.17233 | | 1.20064 | | 0.92162 | |  |
| 209.5 | 1.05803 | | 1.17022 | | 1.19989 | | 0.92769 | |  |
| 210 | 1.06089 | | 1.17329 | | 1.20001 | | 0.93259 | |  |
| 210.5 | 1.06313 | | 1.17515 | | 1.20051 | | 0.93524 | |  |
| 211 | 1.06364 | | 1.1785 | | 1.19997 | | 0.93787 | |  |
| 211.5 | 1.0636 | | 1.17869 | | 1.20111 | | 0.94022 | |  |
| 212 | 1.06423 | | 1.17983 | | 1.20479 | | 0.9433 | |  |
| 212.5 | 1.06554 | | 1.17931 | | 1.20821 | | 0.94611 | |  |
| 213 | 1.06378 | | 1.18182 | | 1.20901 | | 0.94945 | |  |
| 213.5 | 1.06053 | | 1.18332 | | 1.20838 | | 0.9527 | |  |
| 214 | 1.05771 | | 1.18547 | | 1.20681 | | 0.95722 | |  |
| 214.5 | 1.05752 | | 1.18606 | | 1.20706 | | 0.96144 | |  |
| 215 | 1.05826 | | 1.18735 | | 1.20785 | | 0.96526 | |  |
| 215.5 | 1.0583 | | 1.1889 | | 1.2102 | | 0.96807 | |  |
| 216 | 1.05925 | | 1.19062 | | 1.21292 | | 0.97095 | |  |
| 216.5 | 1.05983 | | 1.19206 | | 1.21334 | | 0.97372 | |  |
| 217 | 1.06315 | | 1.19183 | | 1.21293 | | 0.97694 | |  |
| 217.5 | | 1.06436 | | 1.19048 | | 1.20835 | | 0.97994 |  |
| 218 | | 1.0649 | | 1.18828 | | 1.20623 | | 0.98337 |  |
| 218.5 | | 1.06465 | | 1.18677 | | 1.20408 | | 0.98674 |  |
| 219 | | 1.0659 | | 1.18827 | | 1.20478 | | 0.98963 |  |
| 219.5 | | 1.06806 | | 1.18986 | | 1.20579 | | 0.99201 |  |
| 220 | | 1.07127 | | 1.19056 | | 1.20711 | | 0.99355 |  |
| 220.5 | | 1.07373 | | 1.18929 | | 1.20783 | | 0.99517 |  |
| 221 | | 1.07453 | | 1.18903 | | 1.20986 | | 0.99699 |  |
| 221.5 | | 1.07515 | | 1.18934 | | 1.21133 | | 0.9987 |  |
| 222 | | 1.07629 | | 1.19166 | | 1.2136 | | 1.00094 |  |
| 222.5 | | 1.07902 | | 1.19355 | | 1.21272 | | 1.00348 |  |
| 223 | | 1.08017 | | 1.19643 | | 1.21268 | | 1.00689 |  |
| 223.5 | | 1.07973 | | 1.19773 | | 1.2126 | | 1.01092 |  |
| 224 | | 1.07905 | | 1.19982 | | 1.21332 | | 1.01553 |  |
| 224.5 | | 1.08005 | | 1.20116 | | 1.21433 | | 1.01936 |  |
| 225 | | 1.0827 | | 1.2018 | | 1.21446 | | 1.02233 |  |
| 225.5 | | 1.08483 | | 1.20129 | | 1.21602 | | 1.024 |  |
| 226 | | 1.08555 | | 1.19999 | | 1.2172 | | 1.02613 |  |
| 226.5 | | 1.08489 | | 1.19953 | | 1.2177 | | 1.02833 |  |
| 227 | | 1.08497 | | 1.19942 | | 1.21804 | | 1.03064 |  |
| 227.5 | | 1.08539 | | 1.19966 | | 1.21773 | | 1.03259 |  |
| 228 | | 1.08619 | | 1.1996 | | 1.21928 | | 1.03388 |  |
| 228.5 | | 1.0875 | | 1.19894 | | 1.22063 | | 1.03482 |  |
| 229 | | 1.08871 | | 1.1985 | | 1.22286 | | 1.03475 |  |
| 229.5 | | 1.08996 | | 1.19743 | | 1.22359 | | 1.03474 |  |
| 230 | | 1.09004 | | 1.19789 | | 1.2245 | | 1.03451 |  |
| 230.5 | | 1.09082 | | 1.19886 | | 1.2252 | | 1.03489 |  |
| 231 | | 1.09191 | | 1.20099 | | 1.22675 | | 1.03453 |  |
| 231.5 | | 1.09384 | | 1.20279 | | 1.22579 | | 1.03399 |  |
| 232 | | 1.09525 | | 1.20419 | | 1.22598 | | 1.03438 |  |
| 232.5 | | 1.09677 | | 1.20438 | | 1.2258 | | 1.03595 |  |
| 233 | | 1.09777 | | 1.20533 | | 1.22779 | | 1.03926 |  |
| 233.5 | | 1.09922 | | 1.20603 | | 1.2285 | | 1.04233 |  |
| 234 | | 1.10063 | | 1.20694 | | 1.22989 | | 1.0454 |  |
| 234.5 | | 1.10204 | | 1.20601 | | 1.2305 | | 1.04708 |  |
| 235 | | 1.10177 | | 1.2049 | | 1.23172 | | 1.049 |  |
| 235.5 | | 1.10053 | | 1.20424 | | 1.23282 | | 1.05029 |  |
| 236 | | 1.09882 | | 1.20521 | | 1.2337 | | 1.05263 |  |
| 236.5 | | 1.09743 | | 1.20709 | | 1.23392 | | 1.05486 |  |
| 237 | | 1.09678 | | 1.20833 | | 1.23378 | | 1.05733 |  |
| 237.5 | | 1.09605 | | 1.2093 | | 1.23335 | | 1.05991 |  |
| 238 | | 1.09572 | | 1.21129 | | 1.2331 | | 1.06228 |  |
| 238.5 | | 1.09502 | | 1.21419 | | 1.23276 | | 1.06441 |  |
| 239 | | 1.09419 | | 1.21665 | | 1.2326 | | 1.06555 |  |
| 239.5 | | 1.09424 | | 1.21796 | | 1.2312 | | 1.06664 |  |
| 240 | | 1.09502 | | 1.21965 | | 1.22885 | | 1.06736 |  |
| 240.5 | | 1.09694 | | 1.2205 | | 1.22561 | | 1.06808 |  |
| 241 | | 1.09817 | | 1.22113 | | 1.22349 | | 1.06795 |  |
| 241.5 | | 1.09945 | | 1.22033 | | 1.22159 | | 1.06762 |  |
| 242 | | 1.10059 | | 1.21918 | | 1.22222 | | 1.06789 |  |
| 242.5 | | 1.10213 | | 1.21831 | | 1.22343 | | 1.0684 |  |
| 243 | | 1.10378 | | 1.21855 | | 1.22528 | | 1.06929 |  |
| 243.5 | | 1.10523 | | 1.21922 | | 1.22567 | | 1.06919 |  |
| 244 | | 1.10606 | | 1.21939 | | 1.22572 | | 1.06904 |  |
| 244.5 | | 1.10672 | | 1.21817 | | 1.2256 | | 1.06832 |  |
| 245 | | 1.10723 | | 1.21702 | | 1.22558 | | 1.06754 |  |
| 245.5 | | 1.10796 | | 1.21648 | | 1.22554 | | 1.06672 |  |
| 246 | | 1.10822 | | 1.21715 | | 1.22584 | | 1.06586 |  |
| 246.5 | | 1.10743 | | 1.21775 | | 1.22594 | | 1.0651 |  |
| 247 | | 1.10646 | | 1.21782 | | 1.22607 | | 1.06423 |  |
| 247.5 | | 1.10538 | | 1.21941 | | 1.22604 | | 1.06362 |  |
| 248 | | 1.10556 | | 1.2209 | | 1.22717 | | 1.06224 |  |
| 248.5 | | 1.10597 | | 1.22233 | | 1.22835 | | 1.06068 |  |
| 249 | | 1.10663 | | 1.22117 | | 1.22859 | | 1.05882 |  |
| 249.5 | | 1.10752 | | 1.21957 | | 1.22735 | | 1.05779 |  |
| 250 | | 1.1084 | | 1.21729 | | 1.22556 | | 1.05712 |  |
| 250.5 | | 1.10922 | | 1.21524 | | 1.22426 | | 1.05673 |  |
| 251 | | 1.1096 | | 1.21377 | | 1.22392 | | 1.05634 |  |
| 251.5 | | 1.11001 | | 1.21275 | | 1.22372 | | 1.05589 |  |
| 252 | | 1.1103 | | 1.21231 | | 1.22444 | | 1.05566 |  |
| 252.5 | | 1.11126 | | 1.21243 | | 1.225 | | 1.05563 |  |
| 253 | | 1.11193 | | 1.21259 | | 1.22572 | | 1.05557 |  |
| 253.5 | | 1.11174 | | 1.21244 | | 1.22559 | | 1.05477 |  |
| 254 | | 1.11053 | | 1.21097 | | 1.22526 | | 1.05376 |  |
| 254.5 | | 1.10883 | | 1.20874 | | 1.22447 | | 1.05227 |  |
| 255 | | 1.10753 | | 1.20725 | | 1.22358 | | 1.05105 |  |
| 255.5 | | 1.10618 | | 1.20678 | | 1.22342 | | 1.04972 |  |
| 256 | | 1.10507 | | 1.2069 | | 1.22314 | | 1.04871 |  |
| 256.5 | | 1.1038 | | 1.20712 | | 1.22279 | | 1.04769 |  |
| 257 | | 1.10287 | | 1.20777 | | 1.22206 | | 1.04687 |  |
| 257.5 | | 1.10174 | | 1.20886 | | 1.22228 | | 1.04553 |  |
| 258 | | 1.10123 | | 1.20821 | | 1.22311 | | 1.04392 |  |
| 258.5 | | 1.10074 | | 1.20702 | | 1.22346 | | 1.04244 |  |
| 259 | | 1.10013 | | 1.20501 | | 1.2232 | | 1.04149 |  |
| 259.5 | | 1.10044 | | 1.20399 | | 1.22401 | | 1.04103 |  |
| 260 | | 1.10109 | | 1.20343 | | 1.22478 | | 1.04031 |  |
| 260.5 | | 1.10252 | | 1.20415 | | 1.22638 | | 1.03958 |  |
| 261 | | 1.10377 | | 1.20565 | | 1.22756 | | 1.03877 |  |
| 261.5 | | 1.10542 | | 1.20777 | | 1.23025 | | 1.03799 |  |
| 262 | | 1.10701 | | 1.21004 | | 1.23356 | | 1.03668 |  |
| 262.5 | | 1.10866 | | 1.21202 | | 1.23704 | | 1.03567 |  |
| 263 | | 1.109 | | 1.21261 | | 1.24006 | | 1.03429 |  |
| 263.5 | | 1.10938 | | 1.21279 | | 1.24157 | | 1.03336 |  |
| 264 | | 1.10959 | | 1.21205 | | 1.24194 | | 1.03237 |  |
| 264.5 | | 1.11049 | | 1.21145 | | 1.24161 | | 1.03131 |  |
| 265 | | 1.11119 | | 1.21145 | | 1.2417 | | 1.03051 |  |
| 265.5 | | 1.11141 | | 1.21168 | | 1.24136 | | 1.03002 |  |
| 266 | | 1.11166 | | 1.21147 | | 1.24049 | | 1.0304 |  |
| 266.5 | | 1.11203 | | 1.21055 | | 1.23942 | | 1.03013 |  |
| 267 | | 1.11194 | | 1.20951 | | 1.23839 | | 1.02958 |  |
| 267.5 | | 1.11127 | | 1.2082 | | 1.23735 | | 1.02829 |  |
| 268 | | 1.11129 | | 1.20622 | | 1.23579 | | 1.02742 |  |
| 268.5 | | 1.11199 | | 1.2052 | | 1.235 | | 1.02651 |  |
| 269 | | 1.11333 | | 1.20566 | | 1.23428 | | 1.02576 |  |
| 269.5 | | 1.11448 | | 1.20775 | | 1.23403 | | 1.02488 |  |
| 270 | | 1.11505 | | 1.20889 | | 1.23279 | | 1.02374 |  |
| 270.5 | | 1.11551 | | 1.21005 | | 1.23223 | | 1.0228 |  |
| 271 | | 1.11599 | | 1.21163 | | 1.23245 | | 1.02192 |  |
| 271.5 | | 1.11685 | | 1.2144 | | 1.23408 | | 1.02077 |  |
| 272 | | 1.11728 | | 1.21649 | | 1.23613 | | 1.01941 |  |
| 272.5 | | 1.1171 | | 1.21721 | | 1.2375 | | 1.01763 |  |
| 273 | | 1.11667 | | 1.21712 | | 1.23841 | | 1.01598 |  |
| 273.5 | | 1.11709 | | 1.21732 | | 1.23817 | | 1.014 |  |
| 274 | | 1.118 | | 1.21689 | | 1.23797 | | 1.01205 |  |
| 274.5 | | 1.11865 | | 1.21643 | | 1.23801 | | 1.01022 |  |
| 275 | | 1.11838 | | 1.21571 | | 1.23797 | | 1.00848 |  |
| 275.5 | | 1.11816 | | 1.21533 | | 1.23819 | | 1.00634 |  |
| 276 | | 1.11858 | | 1.21444 | | 1.23788 | | 1.00352 |  |
| 276.5 | | 1.11926 | | 1.21362 | | 1.23809 | | 1.0002 |  |
| 277 | | 1.11977 | | 1.21296 | | 1.23729 | | 0.99758 |  |
| 277.5 | | 1.12047 | | 1.21266 | | 1.23616 | | 0.99555 |  |
| 278 | | 1.12261 | | 1.21219 | | 1.23567 | | 0.99411 |  |
| 278.5 | | 1.1244 | | 1.21258 | | 1.23603 | | 0.9927 |  |
| 279 | | 1.12591 | | 1.214 | | 1.23648 | | 0.99139 |  |
| 279.5 | | 1.12606 | | 1.21532 | | 1.23662 | | 0.98965 |  |
| 280 | | 1.12679 | | 1.21531 | | 1.2365 | | 0.9875 |  |
| 280.5 | | 1.1277 | | 1.21508 | | 1.23653 | | 0.98599 |  |
| 281 | | 1.12887 | | 1.21565 | | 1.23669 | | 0.98469 |  |
| 281.5 | | 1.12953 | | 1.21663 | | 1.23754 | | 0.98355 |  |
| 282 | | 1.12927 | | 1.21657 | | 1.23875 | | 0.98187 |  |
| 282.5 | | 1.12809 | | 1.21613 | | 1.23895 | | 0.97985 |  |
| 283 | | 1.12741 | | 1.21504 | | 1.23919 | | 0.9782 |  |
| 283.5 | | 1.12751 | | 1.2142 | | 1.23854 | | 0.97647 |  |
| 284 | | 1.1281 | | 1.21188 | | 1.23867 | | 0.97525 |  |
| 284.5 | | 1.12855 | | 1.20956 | | 1.23868 | | 0.97324 |  |
| 285 | | 1.12904 | | 1.20685 | | 1.23918 | | 0.97032 |  |
| 285.5 | | 1.13026 | | 1.20558 | | 1.2395 | | 0.96687 |  |
| 286 | | 1.13087 | | 1.2054 | | 1.23832 | | 0.96394 |  |
| 286.5 | | 1.13115 | | 1.20591 | | 1.23692 | | 0.96181 |  |
| 287 | | 1.13096 | | 1.20601 | | 1.23469 | | 0.96022 |  |
| 287.5 | | 1.13123 | | 1.20555 | | 1.23299 | | 0.95862 |  |
| 288 | | 1.13204 | | 1.20568 | | 1.23134 | | 0.95644 |  |
| 288.5 | | 1.13196 | | 1.20658 | | 1.22993 | | 0.95382 |  |
| 289 | | 1.13158 | | 1.208 | | 1.2284 | | 0.95048 |  |
| 289.5 | | 1.13124 | | 1.2085 | | 1.22597 | | 0.94723 |  |
| 290 | | 1.13206 | | 1.2085 | | 1.22368 | | 0.94378 |  |
| 290.5 | | 1.1323 | | 1.20841 | | 1.22158 | | 0.94076 |  |
| 291 | | 1.13173 | | 1.20898 | | 1.22066 | | 0.93749 |  |
| 291.5 | | 1.13 | | 1.209 | | 1.21965 | | 0.93477 |  |
| 292 | | 1.12787 | | 1.20775 | | 1.2188 | | 0.93157 |  |
| 292.5 | | 1.12565 | | 1.20582 | | 1.21681 | | 0.92858 |  |
| 293 | | 1.12472 | | 1.20341 | | 1.21505 | | 0.92483 |  |
| 293.5 | | 1.12462 | | 1.20168 | | 1.21301 | | 0.92108 |  |
| 294 | | 1.12579 | | 1.1995 | | 1.21235 | | 0.91701 |  |
| 294.5 | | 1.12713 | | 1.19787 | | 1.21203 | | 0.9124 |  |
| 295 | | 1.12845 | | 1.19627 | | 1.21194 | | 0.90809 |  |
| 295.5 | | 1.12962 | | 1.19596 | | 1.21106 | | 0.90398 |  |
| 296 | | 1.12998 | | 1.1965 | | 1.21013 | | 0.90063 |  |
| 296.5 | | 1.13039 | | 1.19679 | | 1.20922 | | 0.897 |  |
| 297 | | 1.13042 | | 1.19592 | | 1.20886 | | 0.89314 |  |
| 297.5 | | 1.13157 | | 1.19398 | | 1.20838 | | 0.8892 |  |
| 298 | | 1.1327 | | 1.19256 | | 1.20826 | | 0.88491 |  |
| 298.5 | | 1.13321 | | 1.19131 | | 1.20795 | | 0.88173 |  |
| 299 | | 1.13315 | | 1.19059 | | 1.20779 | | 0.87839 |  |
| 299.5 | | 1.13334 | | 1.18994 | | 1.20727 | | 0.87583 |  |
| 300 | | 1.13328 | | 1.18938 | | 1.20751 | | 0.87278 |  |
| 300.5 | | 1.13234 | | 1.18867 | | 1.20715 | | 0.87003 |  |
| 301 | | 1.13119 | | 1.18727 | | 1.20634 | | 0.86771 |  |
| 301.5 | | 1.1299 | | 1.18579 | | 1.20455 | | 0.86542 |  |
| 302 | | 1.12923 | | 1.18433 | | 1.2026 | | 0.86323 |  |
| 302.5 | | 1.12842 | | 1.18335 | | 1.1995 | | 0.86064 |  |
| 303 | | 1.1283 | | 1.18335 | | 1.19649 | | 0.85812 |  |
| 303.5 | | 1.12774 | | 1.18393 | | 1.19305 | | 0.85569 |  |
| 304 | | 1.12818 | | 1.18437 | | 1.18972 | | 0.85345 |  |
| 304.5 | | 1.12811 | | 1.18389 | | 1.18616 | | 0.85103 |  |
| 305 | | 1.12859 | | 1.18329 | | 1.18233 | | 0.84876 |  |
| 305.5 | | 1.12909 | | 1.18346 | | 1.17899 | | 0.84618 |  |
| 306 | | 1.1297 | | 1.1839 | | 1.17554 | | 0.84423 |  |
| 306.5 | | 1.12981 | | 1.18297 | | 1.17265 | | 0.84155 |  |
| 307 | | 1.12965 | | 1.18087 | | 1.17039 | | 0.83881 |  |
| 307.5 | | 1.12975 | | 1.17849 | | 1.16729 | | 0.8359 |  |
| 308 | | 1.12934 | | 1.1773 | | 1.1641 | | 0.83346 |  |
| 308.5 | | 1.12827 | | 1.17611 | | 1.15991 | | 0.83125 |  |
| 309 | | 1.12708 | | 1.17512 | | 1.1571 | | 0.82809 |  |
| 309.5 | | 1.12646 | | 1.17376 | | 1.15362 | | 0.82511 |  |
| 310 | | 1.12593 | | 1.17233 | | 1.15113 | | 0.82186 |  |
| 310.5 | | 1.12555 | | 1.17041 | | 1.14836 | | 0.8184 |  |
| 311 | | 1.12534 | | 1.16832 | | 1.14623 | | 0.81445 |  |
| 311.5 | | 1.12534 | | 1.16674 | | 1.14432 | | 0.81029 |  |
| 312 | | 1.12539 | | 1.16637 | | 1.14197 | | 0.80694 |  |
| 312.5 | | 1.12517 | | 1.16676 | | 1.13928 | | 0.80324 |  |
| 313 | | 1.12462 | | 1.16772 | | 1.13685 | | 0.79967 |  |
| 313.5 | | 1.12497 | | 1.16783 | | 1.13478 | | 0.79561 |  |
| 314 | | 1.12567 | | 1.16765 | | 1.13268 | | 0.79218 |  |
| 314.5 | | 1.12619 | | 1.16686 | | 1.13055 | | 0.78884 |  |
| 315 | | 1.12592 | | 1.16618 | | 1.12806 | | 0.78607 |  |
| 315.5 | | 1.12573 | | 1.16549 | | 1.127 | | 0.78334 |  |
| 316 | | 1.12566 | | 1.16437 | | 1.12577 | | 0.78073 |  |
| 316.5 | | 1.12573 | | 1.16318 | | 1.12553 | | 0.7776 |  |
| 317 | | 1.12584 | | 1.16222 | | 1.12443 | | 0.77486 |  |
| 317.5 | | 1.1261 | | 1.16127 | | 1.12236 | | 0.77233 |  |
| 318 | | 1.12638 | | 1.16035 | | 1.11981 | | 0.77018 |  |
| 318.5 | | 1.12613 | | 1.15824 | | 1.11693 | | 0.76767 |  |
| 319 | | 1.12537 | | 1.15642 | | 1.11543 | | 0.76518 |  |
| 319.5 | | 1.12447 | | 1.154 | | 1.11301 | | 0.76278 |  |
| 320 | | 1.12391 | | 1.15212 | | 1.11098 | | 0.76025 |  |
| 320.5 | | 1.12453 | | 1.15009 | | 1.1083 | | 0.75736 |  |
| 321 | | 1.1249 | | 1.14825 | | 1.10579 | | 0.75419 |  |
| 321.5 | | 1.12439 | | 1.14726 | | 1.10264 | | 0.75133 |  |
| 322 | | 1.12259 | | 1.14681 | | 1.09875 | | 0.74847 |  |
| 322.5 | | 1.12022 | | 1.14593 | | 1.09439 | | 0.74541 |  |
| 323 | | 1.11756 | | 1.14332 | | 1.09025 | | 0.74246 |  |
| 323.5 | | 1.11551 | | 1.13947 | | 1.08532 | | 0.73954 |  |
| 324 | | 1.113 | | 1.13631 | | 1.0795 | | 0.73676 |  |
| 324.5 | | 1.11073 | | 1.13311 | | 1.07351 | | 0.73325 |  |
| 325 | | 1.10841 | | 1.12982 | | 1.06888 | | 0.72958 |  |
| 325.5 | | 1.10693 | | 1.12624 | | 1.06533 | | 0.72571 |  |
| 326 | | 1.10607 | | 1.12295 | | 1.06151 | | 0.72223 |  |
| 326.5 | | 1.10509 | | 1.12031 | | 1.05811 | | 0.71897 |  |
| 327 | | 1.10419 | | 1.11801 | | 1.05589 | | 0.71618 |  |
| 327.5 | | 1.10317 | | 1.11614 | | 1.05417 | | 0.71286 |  |
| 328 | | 1.1027 | | 1.11444 | | 1.05246 | | 0.70947 |  |
| 328.5 | | 1.10202 | | 1.1119 | | 1.05084 | | 0.70577 |  |
| 329 | | 1.10186 | | 1.10947 | | 1.05013 | | 0.70235 |  |
| 329.5 | | 1.10204 | | 1.10706 | | 1.04934 | | 0.69867 |  |
| 330 | | 1.10341 | | 1.10646 | | 1.04839 | | 0.69481 |  |
| 330.5 | | 1.10488 | | 1.10567 | | 1.04754 | | 0.6912 |  |
| 331 | | 1.10518 | | 1.10519 | | 1.04711 | | 0.68748 |  |
| 331.5 | | 1.10418 | | 1.10418 | | 1.04629 | | 0.68427 |  |
| 332 | | 1.10247 | | 1.10293 | | 1.04518 | | 0.6812 |  |
| 332.5 | | 1.10137 | | 1.10113 | | 1.04333 | | 0.67857 |  |
| 333 | | 1.10008 | | 1.09855 | | 1.04063 | | 0.67589 |  |
| 333.5 | | 1.09932 | | 1.09656 | | 1.03771 | | 0.67271 |  |
| 334 | | 1.09791 | | 1.09546 | | 1.0348 | | 0.66962 |  |
| 334.5 | | 1.09651 | | 1.09469 | | 1.0333 | | 0.66668 |  |
| 335 | | 1.09525 | | 1.09382 | | 1.03178 | | 0.66439 |  |
| 335.5 | | 1.09436 | | 1.09284 | | 1.03052 | | 0.66243 |  |
| 336 | | 1.09412 | | 1.09224 | | 1.02954 | | 0.66039 |  |
| 336.5 | | 1.09307 | | 1.09185 | | 1.02909 | | 0.65798 |  |
| 337 | | 1.09248 | | 1.09164 | | 1.02921 | | 0.65546 |  |
| 337.5 | | 1.09192 | | 1.0909 | | 1.02892 | | 0.65272 |  |
| 338 | | 1.09265 | | 1.08954 | | 1.02919 | | 0.65024 |  |
| 338.5 | | 1.09301 | | 1.08768 | | 1.02948 | | 0.64759 |  |
| 339 | | 1.09386 | | 1.08633 | | 1.02991 | | 0.64498 |  |
| 339.5 | | 1.09377 | | 1.08593 | | 1.0295 | | 0.64193 |  |
| 340 | | 1.09444 | | 1.08619 | | 1.02886 | | 0.63885 |  |
| 340.5 | | 1.09465 | | 1.08562 | | 1.02838 | | 0.63622 |  |
| 341 | | 1.09554 | | 1.0849 | | 1.0281 | | 0.63378 |  |
| 341.5 | | 1.09517 | | 1.08345 | | 1.02737 | | 0.63152 |  |
| 342 | | 1.09502 | | 1.08272 | | 1.02664 | | 0.62884 |  |
| 342.5 | | 1.09473 | | 1.08188 | | 1.02543 | | 0.62603 |  |
| 343 | | 1.09444 | | 1.08139 | | 1.02448 | | 0.62296 |  |
| 343.5 | | 1.09338 | | 1.08074 | | 1.02371 | | 0.62017 |  |
| 344 | | 1.09133 | | 1.08044 | | 1.02267 | | 0.61792 |  |
| 344.5 | | 1.09019 | | 1.08041 | | 1.02198 | | 0.61591 |  |
| 345 | | 1.08947 | | 1.08036 | | 1.02057 | | 0.61384 |  |
| 345.5 | | 1.08942 | | 1.07999 | | 1.01949 | | 0.61178 |  |
| 346 | | 1.08914 | | 1.07937 | | 1.01799 | | 0.60983 |  |
| 346.5 | | 1.08869 | | 1.07858 | | 1.01633 | | 0.60833 |  |
| 347 | | 1.08843 | | 1.07793 | | 1.01508 | | 0.6071 |  |
| 347.5 | | 1.08807 | | 1.07696 | | 1.01428 | | 0.60566 |  |
| 348 | | 1.08817 | | 1.07619 | | 1.0138 | | 0.60419 |  |
| 348.5 | | 1.08794 | | 1.07544 | | 1.01253 | | 0.60272 |  |
| 349 | | 1.08811 | | 1.07497 | | 1.01045 | | 0.60126 |  |
| 349.5 | | 1.08819 | | 1.07443 | | 1.00775 | | 0.59952 |  |
| 350 | | 1.08867 | | 1.07424 | | 1.00518 | | 0.5976 |  |
| 350.5 | | 1.08875 | | 1.07384 | | 1.00267 | | 0.59564 |  |
| 351 | | 1.08874 | | 1.07322 | | 1.0003 | | 0.59353 |  |
| 351.5 | | 1.08815 | | 1.07165 | | 0.99787 | | 0.59121 |  |
| 352 | | 1.0882 | | 1.07021 | | 0.99604 | | 0.58856 |  |
| 352.5 | | 1.08782 | | 1.06861 | | 0.9944 | | 0.58572 |  |
| 353 | | 1.0875 | | 1.06744 | | 0.99289 | | 0.58262 |  |
| 353.5 | | 1.08611 | | 1.06584 | | 0.99058 | | 0.57979 |  |
| 354 | | 1.08519 | | 1.06468 | | 0.9881 | | 0.57726 |  |
| 354.5 | | 1.08447 | | 1.06307 | | 0.98563 | | 0.57509 |  |
| 355 | | 1.0837 | | 1.06118 | | 0.98279 | | 0.57273 |  |
| 355.5 | | 1.08258 | | 1.05871 | | 0.98002 | | 0.57057 |  |
| 356 | | 1.08163 | | 1.05642 | | 0.97736 | | 0.56836 |  |
| 356.5 | | 1.08095 | | 1.05415 | | 0.9752 | | 0.56638 |  |
| 357 | | 1.08019 | | 1.05173 | | 0.97345 | | 0.56419 |  |
| 357.5 | | 1.07918 | | 1.04878 | | 0.97147 | | 0.56209 |  |
| 358 | | 1.07855 | | 1.04603 | | 0.96922 | | 0.56014 |  |
| 358.5 | | 1.0778 | | 1.04303 | | 0.96635 | | 0.55811 |  |
| 359 | | 1.0775 | | 1.04013 | | 0.96339 | | 0.55586 |  |
| 359.5 | | 1.07726 | | 1.03688 | | 0.96033 | | 0.55356 |  |
| 360 | | 1.07689 | | 1.03428 | | 0.95749 | | 0.55141 |  |
| 360.5 | | 1.07595 | | 1.03123 | | 0.95487 | | 0.5493 |  |
| 361 | | 1.07471 | | 1.02852 | | 0.95203 | | 0.54736 |  |
| 361.5 | | 1.07385 | | 1.02561 | | 0.94915 | | 0.54544 |  |
| 362 | | 1.07311 | | 1.02383 | | 0.94618 | | 0.54329 |  |
| 362.5 | | 1.07242 | | 1.02226 | | 0.94335 | | 0.54099 |  |
| 363 | | 1.07146 | | 1.02102 | | 0.94041 | | 0.53886 |  |
| 363.5 | | 1.07032 | | 1.01939 | | 0.93701 | | 0.53727 |  |
| 364 | | 1.06888 | | 1.0176 | | 0.93339 | | 0.53593 |  |
| 364.5 | | 1.06763 | | 1.0153 | | 0.92959 | | 0.53437 |  |
| 365 | | 1.06635 | | 1.0128 | | 0.92577 | | 0.53246 |  |
| 365.5 | | 1.06491 | | 1.01021 | | 0.92226 | | 0.53063 |  |
| 366 | | 1.06353 | | 1.0076 | | 0.91873 | | 0.52869 |  |
| 366.5 | | 1.06165 | | 1.00432 | | 0.91512 | | 0.52691 |  |
| 367 | | 1.05994 | | 1.00102 | | 0.91136 | | 0.52522 |  |
| 367.5 | | 1.05793 | | 0.99775 | | 0.9072 | | 0.52359 |  |
| 368 | | 1.05627 | | 0.99508 | | 0.90298 | | 0.52194 |  |
| 368.5 | | 1.05471 | | 0.99196 | | 0.89875 | | 0.52022 |  |
| 369 | | 1.05351 | | 0.9885 | | 0.89457 | | 0.51873 |  |
| 369.5 | | 1.05229 | | 0.98499 | | 0.89065 | | 0.5174 |  |
| 370 | | 1.05064 | | 0.98195 | | 0.88689 | | 0.51598 |  |
| 370.5 | | 1.04852 | | 0.97877 | | 0.88305 | | 0.51461 |  |
| 371 | | 1.04643 | | 0.9755 | | 0.87884 | | 0.51344 |  |
| 371.5 | | 1.04424 | | 0.97216 | | 0.87454 | | 0.5124 |  |
| 372 | | 1.042 | | 0.96888 | | 0.87017 | | 0.51128 |  |
| 372.5 | | 1.03969 | | 0.96585 | | 0.86607 | | 0.51013 |  |
| 373 | | 1.03725 | | 0.96236 | | 0.86181 | | 0.50903 |  |
| 373.5 | | 1.03507 | | 0.95873 | | 0.85734 | | 0.50791 |  |
| 374 | | 1.0329 | | 0.95434 | | 0.85247 | | 0.50651 |  |
| 374.5 | | 1.03087 | | 0.9496 | | 0.84778 | | 0.50479 |  |
| 375 | | 1.02831 | | 0.9446 | | 0.84289 | | 0.50304 |  |
| 375.5 | | 1.02534 | | 0.93938 | | 0.83814 | | 0.50126 |  |
| 376 | | 1.02252 | | 0.93427 | | 0.83278 | | 0.4997 |  |
| 376.5 | | 1.01985 | | 0.92931 | | 0.82757 | | 0.49811 |  |
| 377 | | 1.01739 | | 0.9249 | | 0.82233 | | 0.49678 |  |
| 377.5 | | 1.01452 | | 0.92061 | | 0.81738 | | 0.49539 |  |
| 378 | | 1.01162 | | 0.9162 | | 0.81265 | | 0.49434 |  |
| 378.5 | | 1.00874 | | 0.91147 | | 0.8077 | | 0.49335 |  |
| 379 | | 1.00588 | | 0.90697 | | 0.80286 | | 0.49235 |  |
| 379.5 | | 1.00288 | | 0.90301 | | 0.79797 | | 0.49111 |  |
| 380 | | 0.99951 | | 0.89914 | | 0.79319 | | 0.48995 |  |
| 380.5 | | 0.99614 | | 0.89476 | | 0.78854 | | 0.48902 |  |
| 381 | | 0.99245 | | 0.8902 | | 0.78416 | | 0.4882 |  |
| 381.5 | | 0.98858 | | 0.88608 | | 0.77969 | | 0.4872 |  |
| 382 | | 0.98455 | | 0.88211 | | 0.77523 | | 0.48615 |  |
| 382.5 | | 0.98031 | | 0.87821 | | 0.77078 | | 0.48489 |  |
| 383 | | 0.97617 | | 0.87331 | | 0.76651 | | 0.48349 |  |
| 383.5 | | 0.97194 | | 0.86832 | | 0.762 | | 0.48204 |  |
| 384 | | 0.96763 | | 0.86275 | | 0.75765 | | 0.48061 |  |
| 384.5 | | 0.96307 | | 0.85744 | | 0.75332 | | 0.47934 |  |
| 385 | | 0.95856 | | 0.85207 | | 0.74898 | | 0.47816 |  |
| 385.5 | | 0.95432 | | 0.84695 | | 0.74479 | | 0.47695 |  |
| 386 | | 0.95046 | | 0.84221 | | 0.74048 | | 0.4759 |  |
| 386.5 | | 0.94634 | | 0.83749 | | 0.73657 | | 0.47491 |  |
| 387 | | 0.9419 | | 0.83274 | | 0.7326 | | 0.47431 |  |
| 387.5 | | 0.9375 | | 0.8277 | | 0.72885 | | 0.47377 |  |
| 388 | | 0.93339 | | 0.82268 | | 0.72485 | | 0.47321 |  |
| 388.5 | | 0.92967 | | 0.81802 | | 0.72063 | | 0.47235 |  |
| 389 | | 0.92624 | | 0.81379 | | 0.71667 | | 0.47134 |  |
| 389.5 | | 0.92286 | | 0.80975 | | 0.71281 | | 0.47025 |  |
| 390 | | 0.91986 | | 0.80559 | | 0.70928 | | 0.46944 |  |
| 390.5 | | 0.91686 | | 0.80109 | | 0.70584 | | 0.46863 |  |
| 391 | | 0.914 | | 0.79685 | | 0.70243 | | 0.46786 |  |
| 391.5 | | 0.91107 | | 0.79277 | | 0.69895 | | 0.46709 |  |
| 392 | | 0.90798 | | 0.78893 | | 0.6956 | | 0.46639 |  |
| 392.5 | | 0.90476 | | 0.78478 | | 0.69261 | | 0.46562 |  |
| 393 | | 0.90162 | | 0.78053 | | 0.68978 | | 0.46474 |  |
| 393.5 | | 0.89846 | | 0.77628 | | 0.68662 | | 0.46388 |  |
| 394 | | 0.89522 | | 0.77227 | | 0.68347 | | 0.46305 |  |
| 394.5 | | 0.89181 | | 0.76835 | | 0.68006 | | 0.46218 |  |
| 395 | | 0.88835 | | 0.7645 | | 0.67683 | | 0.46128 |  |
| 395.5 | | 0.88498 | | 0.76099 | | 0.67386 | | 0.46051 |  |
| 396 | | 0.88151 | | 0.75759 | | 0.6711 | | 0.46001 |  |
| 396.5 | | 0.87788 | | 0.75412 | | 0.66852 | | 0.45965 |  |
| 397 | | 0.87421 | | 0.75065 | | 0.66591 | | 0.45922 |  |
| 397.5 | | 0.87085 | | 0.74721 | | 0.66336 | | 0.45857 |  |
| 398 | | 0.86762 | | 0.74417 | | 0.6608 | | 0.45756 |  |
| 398.5 | | 0.86482 | | 0.74119 | | 0.65841 | | 0.4565 |  |
| 399 | | 0.862 | | 0.73826 | | 0.65647 | | 0.45559 |  |
| 399.5 | | 0.85945 | | 0.73538 | | 0.65447 | | 0.45482 |  |
| 400 | | 0.85684 | | 0.73248 | | 0.6522 | | 0.45405 |  |
| 400.5 | | 0.8541 | | 0.72963 | | 0.64976 | | 0.45314 |  |
| 401 | | 0.85123 | | 0.72691 | | 0.6475 | | 0.45246 |  |
| 401.5 | | 0.84803 | | 0.72429 | | 0.6455 | | 0.45174 |  |
| 402 | | 0.84479 | | 0.72166 | | 0.64338 | | 0.45102 |  |
| 402.5 | | 0.84174 | | 0.71867 | | 0.64118 | | 0.45017 |  |
| 403 | | 0.83893 | | 0.71574 | | 0.63858 | | 0.4493 |  |
| 403.5 | | 0.83617 | | 0.71274 | | 0.63585 | | 0.44854 |  |
| 404 | | 0.83308 | | 0.71007 | | 0.63299 | | 0.44792 |  |
| 404.5 | | 0.82983 | | 0.7071 | | 0.63038 | | 0.44734 |  |
| 405 | | 0.82627 | | 0.70418 | | 0.62811 | | 0.44694 |  |
| 405.5 | | 0.82283 | | 0.70107 | | 0.62615 | | 0.44648 |  |
| 406 | | 0.81938 | | 0.69787 | | 0.62407 | | 0.44608 |  |
| 406.5 | | 0.81604 | | 0.69458 | | 0.62203 | | 0.44553 |  |
| 407 | | 0.81261 | | 0.69148 | | 0.62 | | 0.44492 |  |
| 407.5 | | 0.80911 | | 0.68826 | | 0.61822 | | 0.44436 |  |
| 408 | | 0.80554 | | 0.68508 | | 0.61615 | | 0.44376 |  |
| 408.5 | | 0.80195 | | 0.68199 | | 0.61392 | | 0.44346 |  |
| 409 | | 0.79817 | | 0.67909 | | 0.61177 | | 0.44328 |  |
| 409.5 | | 0.79439 | | 0.67629 | | 0.60951 | | 0.44306 |  |
| 410 | | 0.79021 | | 0.67341 | | 0.60723 | | 0.44263 |  |
| 410.5 | | 0.78606 | | 0.67051 | | 0.60484 | | 0.44232 |  |
| 411 | | 0.78174 | | 0.66766 | | 0.60263 | | 0.44216 |  |
| 411.5 | | 0.77778 | | 0.66469 | | 0.60029 | | 0.44187 |  |
| 412 | | 0.77406 | | 0.66176 | | 0.59779 | | 0.44148 |  |
| 412.5 | | 0.77061 | | 0.65879 | | 0.59514 | | 0.441 |  |
| 413 | | 0.76698 | | 0.65604 | | 0.59266 | | 0.44059 |  |
| 413.5 | | 0.76315 | | 0.65319 | | 0.59017 | | 0.44027 |  |
| 414 | | 0.75898 | | 0.65034 | | 0.58764 | | 0.43991 |  |
| 414.5 | | 0.75486 | | 0.64721 | | 0.58523 | | 0.43954 |  |
| 415 | | 0.75073 | | 0.64395 | | 0.58283 | | 0.43918 |  |
| 415.5 | | 0.74685 | | 0.64065 | | 0.58046 | | 0.43855 |  |
| 416 | | 0.74302 | | 0.63747 | | 0.57794 | | 0.43797 |  |
| 416.5 | | 0.73923 | | 0.63431 | | 0.57551 | | 0.43735 |  |
| 417 | | 0.73527 | | 0.6311 | | 0.57316 | | 0.43682 |  |
| 417.5 | | 0.73122 | | 0.62764 | | 0.57066 | | 0.43636 |  |
| 418 | | 0.72696 | | 0.62425 | | 0.56797 | | 0.43595 |  |
| 418.5 | | 0.72269 | | 0.62097 | | 0.56523 | | 0.43554 |  |
| 419 | | 0.71835 | | 0.61773 | | 0.56278 | | 0.43508 |  |
| 419.5 | | 0.71391 | | 0.61449 | | 0.56027 | | 0.43461 |  |
| 420 | | 0.70933 | | 0.61132 | | 0.55777 | | 0.43425 |  |
| 420.5 | | 0.70482 | | 0.60807 | | 0.55512 | | 0.434 |  |
| 421 | | 0.7004 | | 0.60471 | | 0.55268 | | 0.43364 |  |
| 421.5 | | 0.6961 | | 0.6011 | | 0.55028 | | 0.43326 |  |
| 422 | | 0.69141 | | 0.59746 | | 0.54794 | | 0.43292 |  |
| 422.5 | | 0.6865 | | 0.59377 | | 0.54561 | | 0.4327 |  |
| 423 | | 0.68161 | | 0.59009 | | 0.54352 | | 0.43247 |  |
| 423.5 | | 0.6768 | | 0.5865 | | 0.54121 | | 0.43228 |  |
| 424 | | 0.67192 | | 0.58301 | | 0.53905 | | 0.4322 |  |
| 424.5 | | 0.66707 | | 0.57949 | | 0.53676 | | 0.4322 |  |
| 425 | | 0.66218 | | 0.57596 | | 0.53465 | | 0.43239 |  |
| 425.5 | | 0.6575 | | 0.57261 | | 0.53239 | | 0.43243 |  |
| 426 | | 0.65298 | | 0.56924 | | 0.52998 | | 0.43243 |  |
| 426.5 | | 0.64847 | | 0.56589 | | 0.5275 | | 0.43225 |  |
| 427 | | 0.64396 | | 0.56252 | | 0.52512 | | 0.43201 |  |
| 427.5 | | 0.6394 | | 0.55932 | | 0.52275 | | 0.43166 |  |
| 428 | | 0.63499 | | 0.5562 | | 0.52049 | | 0.43131 |  |
| 428.5 | | 0.63072 | | 0.55321 | | 0.51823 | | 0.43082 |  |
| 429 | | 0.62635 | | 0.55039 | | 0.51605 | | 0.43038 |  |
| 429.5 | | 0.62186 | | 0.54761 | | 0.51377 | | 0.43008 |  |
| 430 | | 0.61747 | | 0.54486 | | 0.51159 | | 0.42997 |  |
| 430.5 | | 0.61313 | | 0.54203 | | 0.50936 | | 0.42984 |  |
| 431 | | 0.609 | | 0.53911 | | 0.50736 | | 0.42958 |  |
| 431.5 | | 0.60488 | | 0.53613 | | 0.50541 | | 0.42935 |  |
| 432 | | 0.60084 | | 0.53322 | | 0.50351 | | 0.42917 |  |
| 432.5 | | 0.59699 | | 0.53055 | | 0.50175 | | 0.42902 |  |
| 433 | | 0.59338 | | 0.52789 | | 0.50018 | | 0.42875 |  |
| 433.5 | | 0.58979 | | 0.52541 | | 0.49852 | | 0.42852 |  |
| 434 | | 0.58635 | | 0.52296 | | 0.49693 | | 0.42838 |  |
| 434.5 | | 0.58296 | | 0.52064 | | 0.49542 | | 0.42817 |  |
| 435 | | 0.57978 | | 0.51843 | | 0.49416 | | 0.428 |  |
| 435.5 | | 0.57662 | | 0.51628 | | 0.49274 | | 0.4278 |  |
| 436 | | 0.57366 | | 0.51421 | | 0.49139 | | 0.42749 |  |
| 436.5 | | 0.57079 | | 0.51236 | | 0.49015 | | 0.42705 |  |
| 437 | | 0.56824 | | 0.51066 | | 0.4892 | | 0.42662 |  |
| 437.5 | | 0.56584 | | 0.50902 | | 0.48836 | | 0.42612 |  |
| 438 | | 0.56357 | | 0.50736 | | 0.4876 | | 0.42577 |  |
| 438.5 | | 0.56135 | | 0.50579 | | 0.48697 | | 0.42544 |  |
| 439 | | 0.55925 | | 0.50444 | | 0.48626 | | 0.4252 |  |
| 439.5 | | 0.55738 | | 0.50306 | | 0.48568 | | 0.42516 |  |
| 440 | | 0.55591 | | 0.50169 | | 0.4851 | | 0.4252 |  |
| 440.5 | | 0.55451 | | 0.50038 | | 0.48466 | | 0.42527 |  |
| 441 | | 0.55325 | | 0.49912 | | 0.48429 | | 0.42531 |  |
| 441.5 | | 0.55198 | | 0.49792 | | 0.48375 | | 0.42537 |  |
| 442 | | 0.55104 | | 0.49701 | | 0.48309 | | 0.42553 |  |
| 442.5 | | 0.55007 | | 0.49619 | | 0.4824 | | 0.42561 |  |
| 443 | | 0.54925 | | 0.49554 | | 0.48182 | | 0.4257 |  |
| 443.5 | | 0.54851 | | 0.49518 | | 0.48127 | | 0.42567 |  |
| 444 | | 0.54799 | | 0.49501 | | 0.48088 | | 0.42555 |  |
| 444.5 | | 0.54739 | | 0.49502 | | 0.48049 | | 0.42527 |  |
| 445 | | 0.54669 | | 0.49495 | | 0.47995 | | 0.42505 |  |
| 445.5 | | 0.54598 | | 0.49499 | | 0.47937 | | 0.42496 |  |
| 446 | | 0.54564 | | 0.49508 | | 0.47889 | | 0.4247 |  |
| 446.5 | | 0.5458 | | 0.49527 | | 0.47859 | | 0.4244 |  |
| 447 | | 0.54637 | | 0.49546 | | 0.47838 | | 0.42405 |  |
| 447.5 | | 0.54709 | | 0.4956 | | 0.47815 | | 0.42368 |  |
| 448 | | 0.54782 | | 0.49583 | | 0.47806 | | 0.42344 |  |
| 448.5 | | 0.54881 | | 0.49604 | | 0.47791 | | 0.42305 |  |
| 449 | | 0.54986 | | 0.49625 | | 0.47782 | | 0.42267 |  |
| 449.5 | | 0.55098 | | 0.49624 | | 0.47784 | | 0.4224 |  |
| 450 | | 0.55199 | | 0.49635 | | 0.4781 | | 0.42219 |  |
| 450.5 | | 0.55303 | | 0.49657 | | 0.47841 | | 0.42212 |  |
| 451 | | 0.55403 | | 0.49701 | | 0.47863 | | 0.42201 |  |
| 451.5 | | 0.55516 | | 0.49752 | | 0.47867 | | 0.422 |  |
| 452 | | 0.5566 | | 0.49812 | | 0.47872 | | 0.42207 |  |
| 452.5 | | 0.5579 | | 0.49875 | | 0.47898 | | 0.42206 |  |
| 453 | | 0.55928 | | 0.49965 | | 0.47924 | | 0.42209 |  |
| 453.5 | | 0.56076 | | 0.50055 | | 0.47946 | | 0.42191 |  |
| 454 | | 0.56229 | | 0.50151 | | 0.47954 | | 0.42165 |  |
| 454.5 | | 0.56385 | | 0.50246 | | 0.47956 | | 0.42132 |  |
| 455 | | 0.56528 | | 0.50357 | | 0.47966 | | 0.42103 |  |
| 455.5 | | 0.56712 | | 0.5048 | | 0.48003 | | 0.42073 |  |
| 456 | | 0.56925 | | 0.506 | | 0.48053 | | 0.42036 |  |
| 456.5 | | 0.57163 | | 0.50737 | | 0.48107 | | 0.41997 |  |
| 457 | | 0.57406 | | 0.50867 | | 0.48163 | | 0.41959 |  |
| 457.5 | | 0.57647 | | 0.50995 | | 0.48217 | | 0.41928 |  |
| 458 | | 0.57884 | | 0.5112 | | 0.48284 | | 0.41897 |  |
| 458.5 | | 0.58132 | | 0.51228 | | 0.48343 | | 0.41858 |  |
| 459 | | 0.58378 | | 0.51344 | | 0.48428 | | 0.41828 |  |
| 459.5 | | 0.58616 | | 0.51468 | | 0.48514 | | 0.41796 |  |
| 460 | | 0.58853 | | 0.51617 | | 0.48606 | | 0.41774 |  |
| 460.5 | | 0.59099 | | 0.51763 | | 0.48668 | | 0.41762 |  |
| 461 | | 0.59354 | | 0.51917 | | 0.4873 | | 0.41758 |  |
| 461.5 | | 0.59626 | | 0.52062 | | 0.48787 | | 0.41766 |  |
| 462 | | 0.59905 | | 0.5221 | | 0.48865 | | 0.41758 |  |
| 462.5 | | 0.60177 | | 0.52366 | | 0.48932 | | 0.4175 |  |
| 463 | | 0.60451 | | 0.52517 | | 0.48989 | | 0.41748 |  |
| 463.5 | | 0.6073 | | 0.52662 | | 0.49054 | | 0.41753 |  |
| 464 | | 0.60994 | | 0.5281 | | 0.49114 | | 0.41748 |  |
| 464.5 | | 0.6126 | | 0.5297 | | 0.49193 | | 0.41744 |  |
| 465 | | 0.6152 | | 0.53137 | | 0.49272 | | 0.41734 |  |
| 465.5 | | 0.61793 | | 0.53297 | | 0.49363 | | 0.41733 |  |
| 466 | | 0.62056 | | 0.53448 | | 0.49452 | | 0.41733 |  |
| 466.5 | | 0.62307 | | 0.53607 | | 0.49539 | | 0.41733 |  |
| 467 | | 0.62566 | | 0.53765 | | 0.49651 | | 0.41729 |  |
| 467.5 | | 0.62825 | | 0.53921 | | 0.49766 | | 0.41741 |  |
| 468 | | 0.63109 | | 0.54086 | | 0.49887 | | 0.41742 |  |
| 468.5 | | 0.63401 | | 0.54244 | | 0.49997 | | 0.41739 |  |
| 469 | | 0.63708 | | 0.54422 | | 0.50107 | | 0.41725 |  |
| 469.5 | | 0.64015 | | 0.54596 | | 0.50204 | | 0.41711 |  |
| 470 | | 0.64328 | | 0.54786 | | 0.5029 | | 0.41699 |  |
| 470.5 | | 0.64638 | | 0.54975 | | 0.50378 | | 0.41692 |  |
| 471 | | 0.64948 | | 0.55174 | | 0.50471 | | 0.41692 |  |
| 471.5 | | 0.65253 | | 0.55362 | | 0.50578 | | 0.417 |  |
| 472 | | 0.65573 | | 0.55555 | | 0.50683 | | 0.41694 |  |
| 472.5 | | 0.65899 | | 0.5574 | | 0.50777 | | 0.41694 |  |
| 473 | | 0.66216 | | 0.55928 | | 0.50888 | | 0.41691 |  |
| 473.5 | | 0.66524 | | 0.56123 | | 0.51009 | | 0.417 |  |
| 474 | | 0.66821 | | 0.56321 | | 0.51128 | | 0.417 |  |
| 474.5 | | 0.6714 | | 0.5651 | | 0.5125 | | 0.41703 |  |
| 475 | | 0.67457 | | 0.56684 | | 0.51355 | | 0.41696 |  |
| 475.5 | | 0.6777 | | 0.56854 | | 0.51465 | | 0.41699 |  |
| 476 | | 0.68079 | | 0.57028 | | 0.51575 | | 0.41697 |  |
| 476.5 | | 0.68371 | | 0.57215 | | 0.51694 | | 0.41696 |  |
| 477 | | 0.68672 | | 0.5741 | | 0.51825 | | 0.41695 |  |
| 477.5 | | 0.68984 | | 0.57599 | | 0.51944 | | 0.4171 |  |
| 478 | | 0.69308 | | 0.578 | | 0.52049 | | 0.41708 |  |
| 478.5 | | 0.69645 | | 0.57997 | | 0.52137 | | 0.417 |  |
| 479 | | 0.69992 | | 0.58204 | | 0.52233 | | 0.41687 |  |
| 479.5 | | 0.70334 | | 0.5842 | | 0.52333 | | 0.41683 |  |
| 480 | | 0.70647 | | 0.58654 | | 0.52437 | | 0.41679 |  |
| 480.5 | | 0.70961 | | 0.58889 | | 0.52527 | | 0.41672 |  |
| 481 | | 0.71289 | | 0.59116 | | 0.52611 | | 0.41654 |  |
| 481.5 | | 0.71633 | | 0.59348 | | 0.52701 | | 0.41643 |  |
| 482 | | 0.71977 | | 0.59595 | | 0.5279 | | 0.41628 |  |
| 482.5 | | 0.72314 | | 0.59835 | | 0.52888 | | 0.41606 |  |
| 483 | | 0.72632 | | 0.60085 | | 0.52998 | | 0.41593 |  |
| 483.5 | | 0.72948 | | 0.60324 | | 0.53117 | | 0.41593 |  |
| 484 | | 0.73261 | | 0.60561 | | 0.53238 | | 0.41592 |  |
| 484.5 | | 0.73581 | | 0.60782 | | 0.53344 | | 0.41587 |  |
| 485 | | 0.73891 | | 0.61006 | | 0.53456 | | 0.41577 |  |
| 485.5 | | 0.74197 | | 0.61226 | | 0.53583 | | 0.41591 |  |
| 486 | | 0.74484 | | 0.61438 | | 0.53721 | | 0.41611 |  |
| 486.5 | | 0.74761 | | 0.61645 | | 0.53862 | | 0.41629 |  |
| 487 | | 0.75035 | | 0.6184 | | 0.53998 | | 0.41634 |  |
| 487.5 | | 0.75316 | | 0.62031 | | 0.54131 | | 0.41639 |  |
| 488 | | 0.75602 | | 0.62226 | | 0.54262 | | 0.41642 |  |
| 488.5 | | 0.75911 | | 0.62428 | | 0.54401 | | 0.41656 |  |
| 489 | | 0.76209 | | 0.62635 | | 0.54559 | | 0.4167 |  |
| 489.5 | | 0.76511 | | 0.62853 | | 0.5471 | | 0.4169 |  |
| 490 | | 0.76793 | | 0.63064 | | 0.54865 | | 0.41692 |  |
| 490.5 | | 0.771 | | 0.63268 | | 0.55001 | | 0.41681 |  |
| 491 | | 0.77394 | | 0.63461 | | 0.55131 | | 0.41666 |  |
| 491.5 | | 0.77684 | | 0.63665 | | 0.55256 | | 0.41665 |  |
| 492 | | 0.77945 | | 0.63868 | | 0.55374 | | 0.41669 |  |
| 492.5 | | 0.7821 | | 0.64062 | | 0.55502 | | 0.41662 |  |
| 493 | | 0.78443 | | 0.64259 | | 0.55635 | | 0.41673 |  |
| 493.5 | | 0.78684 | | 0.64452 | | 0.55779 | | 0.41686 |  |
| 494 | | 0.78926 | | 0.64637 | | 0.55903 | | 0.41695 |  |
| 494.5 | | 0.79193 | | 0.64816 | | 0.56017 | | 0.41704 |  |
| 495 | | 0.79464 | | 0.64981 | | 0.56134 | | 0.4172 |  |
| 495.5 | | 0.79703 | | 0.65144 | | 0.56272 | | 0.41757 |  |
| 496 | | 0.79925 | | 0.65295 | | 0.56409 | | 0.41782 |  |
| 496.5 | | 0.8014 | | 0.65439 | | 0.5654 | | 0.41791 |  |
| 497 | | 0.80352 | | 0.6558 | | 0.56656 | | 0.41796 |  |
| 497.5 | | 0.80576 | | 0.65731 | | 0.56766 | | 0.41809 |  |
| 498 | | 0.80796 | | 0.65897 | | 0.56876 | | 0.41834 |  |
| 498.5 | | 0.81032 | | 0.66064 | | 0.56996 | | 0.4186 |  |
| 499 | | 0.81243 | | 0.66239 | | 0.57122 | | 0.41892 |  |
| 499.5 | | 0.81461 | | 0.66403 | | 0.57257 | | 0.41924 |  |
| 500 | | 0.81665 | | 0.66563 | | 0.57403 | | 0.41945 |  |
| 500.5 | | 0.8188 | | 0.66718 | | 0.57527 | | 0.41964 |  |
| 501 | | 0.82087 | | 0.66889 | | 0.57641 | | 0.41983 |  |
| 501.5 | | 0.82294 | | 0.67058 | | 0.57756 | | 0.42002 |  |
| 502 | | 0.82496 | | 0.67243 | | 0.57881 | | 0.4203 |  |
| 502.5 | | 0.82712 | | 0.67438 | | 0.58031 | | 0.42055 |  |
| 503 | | 0.8292 | | 0.67636 | | 0.58171 | | 0.42081 |  |
| 503.5 | | 0.83151 | | 0.67829 | | 0.58319 | | 0.42095 |  |
| 504 | | 0.83395 | | 0.67988 | | 0.58435 | | 0.42109 |  |
| 504.5 | | 0.83642 | | 0.68149 | | 0.58563 | | 0.42136 |  |
| 505 | | 0.83872 | | 0.68298 | | 0.58693 | | 0.42168 |  |
| 505.5 | | 0.84074 | | 0.68465 | | 0.58851 | | 0.42195 |  |
| 506 | | 0.84276 | | 0.6861 | | 0.58991 | | 0.42209 |  |
| 506.5 | | 0.84473 | | 0.68748 | | 0.59141 | | 0.42228 |  |
| 507 | | 0.84664 | | 0.68878 | | 0.59268 | | 0.42251 |  |
| 507.5 | | 0.84849 | | 0.69016 | | 0.59405 | | 0.4227 |  |
| 508 | | 0.85024 | | 0.69181 | | 0.59523 | | 0.42289 |  |
| 508.5 | | 0.85202 | | 0.69343 | | 0.59644 | | 0.42308 |  |
| 509 | | 0.85361 | | 0.69513 | | 0.5976 | | 0.42331 |  |
| 509.5 | | 0.85529 | | 0.69666 | | 0.59878 | | 0.42344 |  |
| 510 | | 0.85702 | | 0.69829 | | 0.59999 | | 0.4236 |  |
| 510.5 | | 0.85881 | | 0.69975 | | 0.60097 | | 0.42378 |  |
| 511 | | 0.86059 | | 0.70114 | | 0.60194 | | 0.42404 |  |
| 511.5 | | 0.86232 | | 0.70229 | | 0.60294 | | 0.42431 |  |
| 512 | | 0.86416 | | 0.70349 | | 0.60411 | | 0.42466 |  |
| 512.5 | | 0.86597 | | 0.70464 | | 0.60515 | | 0.42489 |  |
| 513 | | 0.86769 | | 0.70574 | | 0.60606 | | 0.42511 |  |
| 513.5 | | 0.8694 | | 0.70678 | | 0.60698 | | 0.4254 |  |
| 514 | | 0.87099 | | 0.70761 | | 0.60773 | | 0.42578 |  |
| 514.5 | | 0.8726 | | 0.70867 | | 0.60855 | | 0.42612 |  |
| 515 | | 0.87419 | | 0.70961 | | 0.60942 | | 0.4264 |  |
| 515.5 | | 0.87574 | | 0.71051 | | 0.61039 | | 0.42666 |  |
| 516 | | 0.87715 | | 0.71125 | | 0.61148 | | 0.42692 |  |
| 516.5 | | 0.87858 | | 0.71205 | | 0.61268 | | 0.42722 |  |
| 517 | | 0.8798 | | 0.71294 | | 0.61381 | | 0.42754 |  |
| 517.5 | | 0.88091 | | 0.71406 | | 0.61503 | | 0.42778 |  |
| 518 | | 0.88191 | | 0.71543 | | 0.61608 | | 0.42806 |  |
| 518.5 | | 0.88303 | | 0.71682 | | 0.61711 | | 0.42841 |  |
| 519 | | 0.88413 | | 0.7183 | | 0.618 | | 0.42868 |  |
| 519.5 | | 0.88512 | | 0.71972 | | 0.61897 | | 0.42894 |  |
| 520 | | 0.88636 | | 0.72107 | | 0.61994 | | 0.4291 |  |
| 520.5 | | 0.88742 | | 0.72213 | | 0.62094 | | 0.42928 |  |
| 521 | | 0.88861 | | 0.72311 | | 0.62194 | | 0.42941 |  |
| 521.5 | | 0.88973 | | 0.72408 | | 0.62289 | | 0.42961 |  |
| 522 | | 0.89103 | | 0.72524 | | 0.62386 | | 0.42979 |  |
| 522.5 | | 0.89208 | | 0.72638 | | 0.62459 | | 0.42993 |  |
| 523 | | 0.89305 | | 0.72754 | | 0.6254 | | 0.43005 |  |
| 523.5 | | 0.89407 | | 0.72845 | | 0.62596 | | 0.43012 |  |
| 524 | | 0.8951 | | 0.72942 | | 0.62651 | | 0.43021 |  |
| 524.5 | | 0.89605 | | 0.73043 | | 0.62702 | | 0.4303 |  |
| 525 | | 0.89679 | | 0.73143 | | 0.62773 | | 0.43053 |  |
| 525.5 | | 0.89751 | | 0.73243 | | 0.62843 | | 0.43069 |  |
| 526 | | 0.89825 | | 0.73331 | | 0.62923 | | 0.43084 |  |
| 526.5 | | 0.89913 | | 0.7342 | | 0.62992 | | 0.43101 |  |
| 527 | | 0.89997 | | 0.73517 | | 0.63069 | | 0.43126 |  |
| 527.5 | | 0.90087 | | 0.73598 | | 0.63143 | | 0.43146 |  |
| 528 | | 0.90181 | | 0.73694 | | 0.63211 | | 0.43184 |  |
| 528.5 | | 0.90278 | | 0.73779 | | 0.63257 | | 0.43216 |  |
| 529 | | 0.90369 | | 0.73867 | | 0.63304 | | 0.43252 |  |
| 529.5 | | 0.90451 | | 0.73938 | | 0.63369 | | 0.43286 |  |
| 530 | | 0.90538 | | 0.73998 | | 0.63445 | | 0.43327 |  |
| 530.5 | | 0.90603 | | 0.74066 | | 0.63522 | | 0.43369 |  |
| 531 | | 0.90683 | | 0.74138 | | 0.6359 | | 0.43401 |  |
| 531.5 | | 0.90769 | | 0.74224 | | 0.63661 | | 0.43434 |  |
| 532 | | 0.90854 | | 0.74288 | | 0.63738 | | 0.43457 |  |
| 532.5 | | 0.90917 | | 0.74329 | | 0.63805 | | 0.4348 |  |
| 533 | | 0.90985 | | 0.74356 | | 0.6385 | | 0.43503 |  |
| 533.5 | | 0.91059 | | 0.74377 | | 0.63881 | | 0.43537 |  |
| 534 | | 0.91117 | | 0.74427 | | 0.63922 | | 0.43574 |  |
| 534.5 | | 0.91162 | | 0.74496 | | 0.63956 | | 0.43615 |  |
| 535 | | 0.91226 | | 0.7458 | | 0.64006 | | 0.43642 |  |
| 535.5 | | 0.91311 | | 0.74652 | | 0.64048 | | 0.43649 |  |
| 536 | | 0.91411 | | 0.74713 | | 0.64098 | | 0.43653 |  |
| 536.5 | | 0.91492 | | 0.74786 | | 0.64138 | | 0.43654 |  |
| 537 | | 0.91559 | | 0.74865 | | 0.6418 | | 0.43655 |  |
| 537.5 | | 0.91619 | | 0.74932 | | 0.64223 | | 0.43671 |  |
| 538 | | 0.91688 | | 0.75017 | | 0.64271 | | 0.43695 |  |
| 538.5 | | 0.91749 | | 0.75073 | | 0.64323 | | 0.43727 |  |
| 539 | | 0.91794 | | 0.75132 | | 0.64375 | | 0.4376 |  |
| 539.5 | | 0.91825 | | 0.75166 | | 0.64414 | | 0.43787 |  |
| 540 | | 0.91849 | | 0.75205 | | 0.64459 | | 0.43815 |  |
| 540.5 | | 0.91889 | | 0.75245 | | 0.64482 | | 0.43817 |  |
| 541 | | 0.9193 | | 0.75284 | | 0.64519 | | 0.43815 |  |
| 541.5 | | 0.91979 | | 0.7531 | | 0.64542 | | 0.43815 |  |
| 542 | | 0.92004 | | 0.75315 | | 0.64572 | | 0.43828 |  |
| 542.5 | | 0.92024 | | 0.75315 | | 0.64584 | | 0.43853 |  |
| 543 | | 0.92057 | | 0.75329 | | 0.646 | | 0.43886 |  |
| 543.5 | | 0.921 | | 0.75361 | | 0.64621 | | 0.43924 |  |
| 544 | | 0.92144 | | 0.75411 | | 0.64638 | | 0.43958 |  |
| 544.5 | | 0.9219 | | 0.75453 | | 0.64647 | | 0.43991 |  |
| 545 | | 0.92225 | | 0.755 | | 0.64668 | | 0.44012 |  |
| 545.5 | | 0.92254 | | 0.75539 | | 0.64698 | | 0.44029 |  |
| 546 | | 0.92263 | | 0.75579 | | 0.64734 | | 0.44052 |  |
| 546.5 | | 0.92273 | | 0.75621 | | 0.64768 | | 0.44082 |  |
| 547 | | 0.92287 | | 0.75666 | | 0.64804 | | 0.44108 |  |
| 547.5 | | 0.92312 | | 0.75707 | | 0.6484 | | 0.44146 |  |
| 548 | | 0.92336 | | 0.75745 | | 0.64859 | | 0.44186 |  |
| 548.5 | | 0.92346 | | 0.75768 | | 0.64877 | | 0.44228 |  |
| 549 | | 0.92358 | | 0.75787 | | 0.64871 | | 0.44241 |  |
| 549.5 | | 0.9238 | | 0.75803 | | 0.64863 | | 0.44253 |  |
| 550 | | 0.92395 | | 0.75825 | | 0.64841 | | 0.44262 |  |
| 550.5 | | 0.92422 | | 0.75863 | | 0.64822 | | 0.4428 |  |
| 551 | | 0.92463 | | 0.75902 | | 0.64808 | | 0.44295 |  |
| 551.5 | | 0.92491 | | 0.75947 | | 0.6479 | | 0.44316 |  |
| 552 | | 0.92504 | | 0.75992 | | 0.64763 | | 0.44337 |  |
| 552.5 | | 0.92506 | | 0.76038 | | 0.64722 | | 0.44346 |  |
| 553 | | 0.9253 | | 0.76092 | | 0.64697 | | 0.44352 |  |
| 553.5 | | 0.92559 | | 0.76146 | | 0.6466 | | 0.4435 |  |
| 554 | | 0.92566 | | 0.76198 | | 0.64626 | | 0.44347 |  |
| 554.5 | | 0.92566 | | 0.76218 | | 0.64583 | | 0.44338 |  |
| 555 | | 0.92562 | | 0.76242 | | 0.64566 | | 0.4433 |  |
| 555.5 | | 0.92568 | | 0.7625 | | 0.6455 | | 0.44323 |  |
| 556 | | 0.92551 | | 0.76246 | | 0.64543 | | 0.44324 |  |
| 556.5 | | 0.92546 | | 0.76232 | | 0.64529 | | 0.44322 |  |
| 557 | | 0.92505 | | 0.76212 | | 0.6451 | | 0.44319 |  |
| 557.5 | | 0.9248 | | 0.76179 | | 0.64489 | | 0.44332 |  |
| 558 | | 0.92444 | | 0.7614 | | 0.64468 | | 0.44354 |  |
| 558.5 | | 0.92416 | | 0.76097 | | 0.6447 | | 0.44376 |  |
| 559 | | 0.92366 | | 0.76042 | | 0.64467 | | 0.44387 |  |
| 559.5 | | 0.923 | | 0.75986 | | 0.64462 | | 0.44411 |  |
| 560 | | 0.92212 | | 0.75916 | | 0.64434 | | 0.4445 |  |
| 560.5 | | 0.92117 | | 0.75851 | | 0.64437 | | 0.44509 |  |
| 561 | | 0.92033 | | 0.75778 | | 0.64438 | | 0.44561 |  |
| 561.5 | | 0.91926 | | 0.7571 | | 0.64445 | | 0.44616 |  |
| 562 | | 0.91843 | | 0.75628 | | 0.64427 | | 0.44661 |  |
| 562.5 | | 0.91755 | | 0.75553 | | 0.64438 | | 0.44695 |  |
| 563 | | 0.91684 | | 0.75465 | | 0.64456 | | 0.44727 |  |
| 563.5 | | 0.9162 | | 0.75378 | | 0.64458 | | 0.44753 |  |
| 564 | | 0.91559 | | 0.75283 | | 0.64439 | | 0.44794 |  |
| 564.5 | | 0.91517 | | 0.75168 | | 0.64413 | | 0.44838 |  |
| 565 | | 0.91455 | | 0.75057 | | 0.6439 | | 0.44883 |  |
| 565.5 | | 0.91388 | | 0.74962 | | 0.64374 | | 0.44916 |  |
| 566 | | 0.91314 | | 0.74879 | | 0.64348 | | 0.44945 |  |
| 566.5 | | 0.91259 | | 0.74812 | | 0.64315 | | 0.44967 |  |
| 567 | | 0.91196 | | 0.74742 | | 0.64277 | | 0.4499 |  |
| 567.5 | | 0.91159 | | 0.74669 | | 0.64236 | | 0.45015 |  |
| 568 | | 0.91098 | | 0.74595 | | 0.64195 | | 0.45035 |  |
| 568.5 | | 0.91036 | | 0.74522 | | 0.64153 | | 0.4505 |  |
| 569 | | 0.90953 | | 0.74458 | | 0.64101 | | 0.45052 |  |
| 569.5 | | 0.90866 | | 0.74398 | | 0.64033 | | 0.45057 |  |
| 570 | | 0.9077 | | 0.7433 | | 0.63985 | | 0.45063 |  |
| 570.5 | | 0.90693 | | 0.74268 | | 0.63952 | | 0.45085 |  |
| 571 | | 0.90615 | | 0.74208 | | 0.63909 | | 0.45111 |  |
| 571.5 | | 0.90543 | | 0.74159 | | 0.63863 | | 0.45134 |  |
| 572 | | 0.90448 | | 0.74127 | | 0.63806 | | 0.45134 |  |
| 572.5 | | 0.90344 | | 0.74101 | | 0.63761 | | 0.45121 |  |
| 573 | | 0.90252 | | 0.74057 | | 0.63692 | | 0.45119 |  |
| 573.5 | | 0.90181 | | 0.74021 | | 0.63615 | | 0.45123 |  |
| 574 | | 0.90123 | | 0.74 | | 0.63542 | | 0.45134 |  |
| 574.5 | | 0.90061 | | 0.7398 | | 0.63468 | | 0.4514 |  |
| 575 | | 0.89997 | | 0.73987 | | 0.63403 | | 0.45131 |  |
| 575.5 | | 0.89952 | | 0.74012 | | 0.63335 | | 0.45115 |  |
| 576 | | 0.89925 | | 0.74046 | | 0.63252 | | 0.45115 |  |
| 576.5 | | 0.89889 | | 0.74058 | | 0.63174 | | 0.45128 |  |
| 577 | | 0.89844 | | 0.74064 | | 0.63081 | | 0.45135 |  |
| 577.5 | | 0.8981 | | 0.7408 | | 0.62994 | | 0.45132 |  |
| 578 | | 0.89778 | | 0.74098 | | 0.6291 | | 0.45121 |  |
| 578.5 | | 0.89766 | | 0.74108 | | 0.62832 | | 0.45117 |  |
| 579 | | 0.89741 | | 0.74092 | | 0.62745 | | 0.45117 |  |
| 579.5 | | 0.89716 | | 0.74066 | | 0.62662 | | 0.45127 |  |
| 580 | | 0.89679 | | 0.74018 | | 0.62586 | | 0.45127 |  |
| 580.5 | | 0.89632 | | 0.73986 | | 0.62496 | | 0.4513 |  |
| 581 | | 0.89554 | | 0.73942 | | 0.62396 | | 0.45127 |  |
| 581.5 | | 0.89468 | | 0.73906 | | 0.62291 | | 0.45112 |  |
| 582 | | 0.89358 | | 0.73847 | | 0.62181 | | 0.45083 |  |
| 582.5 | | 0.89261 | | 0.73772 | | 0.6209 | | 0.45077 |  |
| 583 | | 0.8916 | | 0.73683 | | 0.62022 | | 0.45075 |  |
| 583.5 | | 0.89063 | | 0.7361 | | 0.61982 | | 0.45073 |  |
| 584 | | 0.88955 | | 0.7353 | | 0.61922 | | 0.45081 |  |
| 584.5 | | 0.88842 | | 0.73437 | | 0.61845 | | 0.4509 |  |
| 585 | | 0.88725 | | 0.73335 | | 0.61772 | | 0.45098 |  |
| 585.5 | | 0.88605 | | 0.73232 | | 0.61715 | | 0.4511 |  |
| 586 | | 0.88474 | | 0.7313 | | 0.61656 | | 0.45137 |  |
| 586.5 | | 0.88325 | | 0.73032 | | 0.61597 | | 0.45154 |  |
| 587 | | 0.88182 | | 0.72951 | | 0.61528 | | 0.45165 |  |
| 587.5 | | 0.88044 | | 0.72857 | | 0.6148 | | 0.45179 |  |
| 588 | | 0.87914 | | 0.72745 | | 0.61443 | | 0.452 |  |
| 588.5 | | 0.87785 | | 0.72621 | | 0.6141 | | 0.45242 |  |
| 589 | | 0.87641 | | 0.72501 | | 0.61368 | | 0.45276 |  |
| 589.5 | | 0.87504 | | 0.72389 | | 0.61323 | | 0.45317 |  |
| 590 | | 0.87372 | | 0.72278 | | 0.61268 | | 0.45336 |  |
| 590.5 | | 0.87246 | | 0.72167 | | 0.61203 | | 0.45361 |  |
| 591 | | 0.87121 | | 0.72054 | | 0.61141 | | 0.45374 |  |
| 591.5 | | 0.86969 | | 0.71951 | | 0.61079 | | 0.45395 |  |
| 592 | | 0.86817 | | 0.71833 | | 0.61034 | | 0.4541 |  |
| 592.5 | | 0.86669 | | 0.71715 | | 0.60994 | | 0.45443 |  |
| 593 | | 0.86547 | | 0.71584 | | 0.6095 | | 0.45468 |  |
| 593.5 | | 0.86432 | | 0.71469 | | 0.60887 | | 0.45499 |  |
| 594 | | 0.86309 | | 0.71357 | | 0.60799 | | 0.45534 |  |
| 594.5 | | 0.86192 | | 0.71242 | | 0.60719 | | 0.4555 |  |
| 595 | | 0.86065 | | 0.7113 | | 0.60644 | | 0.4556 |  |
| 595.5 | | 0.85941 | | 0.71021 | | 0.60568 | | 0.45551 |  |
| 596 | | 0.85811 | | 0.70927 | | 0.60479 | | 0.45554 |  |
| 596.5 | | 0.85685 | | 0.70832 | | 0.60396 | | 0.45554 |  |
| 597 | | 0.85542 | | 0.70729 | | 0.60317 | | 0.45566 |  |
| 597.5 | | 0.85395 | | 0.7062 | | 0.60256 | | 0.45584 |  |
| 598 | | 0.85247 | | 0.70508 | | 0.60192 | | 0.456 |  |
| 598.5 | | 0.85097 | | 0.70394 | | 0.60133 | | 0.45622 |  |
| 599 | | 0.84944 | | 0.70271 | | 0.60065 | | 0.45635 |  |
| 599.5 | | 0.84803 | | 0.70133 | | 0.59987 | | 0.45653 |  |
| 600 | | 0.84664 | | 0.70009 | | 0.59917 | | 0.45648 |  |
| 600.5 | | 0.84511 | | 0.69898 | | 0.5984 | | 0.45642 |  |
| 601 | | 0.8432 | | 0.69816 | | 0.59781 | | 0.4564 |  |
| 601.5 | | 0.84116 | | 0.6973 | | 0.59714 | | 0.45652 |  |
| 602 | | 0.83944 | | 0.69641 | | 0.59645 | | 0.45658 |  |
| 602.5 | | 0.83795 | | 0.69529 | | 0.59576 | | 0.45662 |  |
| 603 | | 0.83676 | | 0.69426 | | 0.59512 | | 0.45653 |  |
| 603.5 | | 0.83542 | | 0.69329 | | 0.59457 | | 0.45646 |  |
| 604 | | 0.83409 | | 0.69236 | | 0.59401 | | 0.4564 |  |
| 604.5 | | 0.83268 | | 0.69132 | | 0.59346 | | 0.45631 |  |
| 605 | | 0.83109 | | 0.69022 | | 0.59268 | | 0.45625 |  |
| 605.5 | | 0.82961 | | 0.68917 | | 0.59182 | | 0.45611 |  |
| 606 | | 0.82804 | | 0.68822 | | 0.591 | | 0.45609 |  |
| 606.5 | | 0.82636 | | 0.68726 | | 0.59024 | | 0.45603 |  |
| 607 | | 0.82438 | | 0.68618 | | 0.58962 | | 0.45606 |  |
| 607.5 | | 0.82264 | | 0.68501 | | 0.58885 | | 0.45625 |  |
| 608 | | 0.82091 | | 0.68376 | | 0.58802 | | 0.4564 |  |
| 608.5 | | 0.81942 | | 0.6824 | | 0.58702 | | 0.45656 |  |
| 609 | | 0.81778 | | 0.68098 | | 0.58585 | | 0.45673 |  |
| 609.5 | | 0.81629 | | 0.67965 | | 0.58485 | | 0.45689 |  |
| 610 | | 0.81459 | | 0.6786 | | 0.58381 | | 0.457 |  |
| 610.5 | | 0.81305 | | 0.67761 | | 0.58286 | | 0.45727 |  |
| 611 | | 0.81131 | | 0.6766 | | 0.58186 | | 0.45759 |  |
| 611.5 | | 0.80976 | | 0.67551 | | 0.58089 | | 0.45786 |  |
| 612 | | 0.80832 | | 0.67444 | | 0.57995 | | 0.45801 |  |
| 612.5 | | 0.80681 | | 0.6734 | | 0.57897 | | 0.45815 |  |
| 613 | | 0.8052 | | 0.67235 | | 0.57792 | | 0.45826 |  |
| 613.5 | | 0.80342 | | 0.67111 | | 0.5769 | | 0.45833 |  |
| 614 | | 0.80159 | | 0.66998 | | 0.57593 | | 0.45837 |  |
| 614.5 | | 0.79967 | | 0.66882 | | 0.57514 | | 0.4584 |  |
| 615 | | 0.79778 | | 0.66765 | | 0.57413 | | 0.45836 |  |
| 615.5 | | 0.79608 | | 0.66651 | | 0.57315 | | 0.45823 |  |
| 616 | | 0.79425 | | 0.66527 | | 0.57222 | | 0.45809 |  |
| 616.5 | | 0.79247 | | 0.66405 | | 0.57154 | | 0.45803 |  |
| 617 | | 0.79066 | | 0.66277 | | 0.57088 | | 0.45806 |  |
| 617.5 | | 0.78909 | | 0.66152 | | 0.57004 | | 0.45793 |  |
| 618 | | 0.7873 | | 0.66045 | | 0.56939 | | 0.45774 |  |
| 618.5 | | 0.78565 | | 0.65935 | | 0.5687 | | 0.45754 |  |
| 619 | | 0.78376 | | 0.6582 | | 0.56824 | | 0.45735 |  |
| 619.5 | | 0.78201 | | 0.65705 | | 0.56781 | | 0.45722 |  |
| 620 | | 0.78018 | | 0.65607 | | 0.56711 | | 0.45701 |  |
| 620.5 | | 0.77855 | | 0.65497 | | 0.56633 | | 0.45697 |  |
| 621 | | 0.77666 | | 0.65393 | | 0.56528 | | 0.4568 |  |
| 621.5 | | 0.77464 | | 0.65285 | | 0.56439 | | 0.45675 |  |
| 622 | | 0.77245 | | 0.65186 | | 0.56345 | | 0.45667 |  |
| 622.5 | | 0.77022 | | 0.65081 | | 0.56241 | | 0.45664 |  |
| 623 | | 0.76802 | | 0.64959 | | 0.56133 | | 0.45661 |  |
| 623.5 | | 0.76596 | | 0.64833 | | 0.5604 | | 0.45655 |  |
| 624 | | 0.76395 | | 0.64718 | | 0.55962 | | 0.45657 |  |
| 624.5 | | 0.76223 | | 0.64597 | | 0.55879 | | 0.4566 |  |
| 625 | | 0.76049 | | 0.64473 | | 0.55775 | | 0.45666 |  |
| 625.5 | | 0.75893 | | 0.6435 | | 0.55665 | | 0.45667 |  |
| 626 | | 0.75724 | | 0.64222 | | 0.55576 | | 0.45667 |  |
| 626.5 | | 0.75555 | | 0.64086 | | 0.55491 | | 0.45668 |  |
| 627 | | 0.75372 | | 0.63937 | | 0.55411 | | 0.45661 |  |
| 627.5 | | 0.75193 | | 0.63795 | | 0.55325 | | 0.45648 |  |
| 628 | | 0.75001 | | 0.63666 | | 0.55241 | | 0.45643 |  |
| 628.5 | | 0.74823 | | 0.63521 | | 0.55152 | | 0.45631 |  |
| 629 | | 0.7463 | | 0.63359 | | 0.55063 | | 0.45615 |  |
| 629.5 | | 0.74431 | | 0.63203 | | 0.54974 | | 0.45604 |  |
| 630 | | 0.74223 | | 0.63061 | | 0.54883 | | 0.45601 |  |
| 630.5 | | 0.7402 | | 0.62906 | | 0.54781 | | 0.45602 |  |
| 631 | | 0.7381 | | 0.6276 | | 0.54685 | | 0.45593 |  |
| 631.5 | | 0.73583 | | 0.62615 | | 0.54603 | | 0.45584 |  |
| 632 | | 0.73365 | | 0.62479 | | 0.54535 | | 0.45584 |  |
| 632.5 | | 0.73155 | | 0.62328 | | 0.54458 | | 0.4559 |  |
| 633 | | 0.72947 | | 0.62176 | | 0.54383 | | 0.456 |  |
| 633.5 | | 0.72742 | | 0.62035 | | 0.54315 | | 0.45591 |  |
| 634 | | 0.72533 | | 0.61912 | | 0.54234 | | 0.45585 |  |
| 634.5 | | 0.72346 | | 0.61798 | | 0.54142 | | 0.45579 |  |
| 635 | | 0.72158 | | 0.61677 | | 0.54042 | | 0.45564 |  |
| 635.5 | | 0.71974 | | 0.61568 | | 0.53953 | | 0.45543 |  |
| 636 | | 0.71775 | | 0.61443 | | 0.53868 | | 0.45512 |  |
| 636.5 | | 0.71562 | | 0.61326 | | 0.53772 | | 0.45474 |  |
| 637 | | 0.71343 | | 0.61195 | | 0.53673 | | 0.45439 |  |
| 637.5 | | 0.71136 | | 0.61064 | | 0.53559 | | 0.45408 |  |
| 638 | | 0.70926 | | 0.60926 | | 0.53452 | | 0.45379 |  |
| 638.5 | | 0.70719 | | 0.60786 | | 0.53375 | | 0.45353 |  |
| 639 | | 0.70506 | | 0.60662 | | 0.53291 | | 0.45338 |  |
| 639.5 | | 0.70297 | | 0.60552 | | 0.5322 | | 0.45323 |  |
| 640 | | 0.701 | | 0.60436 | | 0.53143 | | 0.4531 |  |
| 640.5 | | 0.69901 | | 0.60317 | | 0.53058 | | 0.45289 |  |
| 641 | | 0.69693 | | 0.60203 | | 0.52966 | | 0.45278 |  |
| 641.5 | | 0.69473 | | 0.60086 | | 0.52872 | | 0.45269 |  |
| 642 | | 0.69266 | | 0.59963 | | 0.52783 | | 0.45272 |  |
| 642.5 | | 0.69065 | | 0.59815 | | 0.52691 | | 0.45275 |  |
| 643 | | 0.6887 | | 0.59672 | | 0.52601 | | 0.45273 |  |
| 643.5 | | 0.68681 | | 0.59533 | | 0.52501 | | 0.45271 |  |
| 644 | | 0.68492 | | 0.59403 | | 0.52386 | | 0.45274 |  |
| 644.5 | | 0.6832 | | 0.59266 | | 0.52265 | | 0.45275 |  |
| 645 | | 0.6815 | | 0.59114 | | 0.52152 | | 0.45256 |  |
| 645.5 | | 0.6798 | | 0.58964 | | 0.52051 | | 0.45232 |  |
| 646 | | 0.67809 | | 0.58816 | | 0.51962 | | 0.45209 |  |
| 646.5 | | 0.67607 | | 0.5868 | | 0.51862 | | 0.45188 |  |
| 647 | | 0.6742 | | 0.58535 | | 0.51771 | | 0.45168 |  |
| 647.5 | | 0.67219 | | 0.58389 | | 0.51685 | | 0.45159 |  |
| 648 | | 0.67042 | | 0.58255 | | 0.51614 | | 0.45152 |  |
| 648.5 | | 0.66856 | | 0.58138 | | 0.51547 | | 0.45146 |  |
| 649 | | 0.66681 | | 0.58046 | | 0.51479 | | 0.45125 |  |
| 649.5 | | 0.66493 | | 0.57952 | | 0.5142 | | 0.45099 |  |
| 650 | | 0.66294 | | 0.57855 | | 0.5138 | | 0.45075 |  |
| 650.5 | | 0.66084 | | 0.57761 | | 0.51318 | | 0.45064 |  |
| 651 | | 0.65891 | | 0.57654 | | 0.51238 | | 0.45063 |  |
| 651.5 | | 0.6569 | | 0.57547 | | 0.51149 | | 0.45047 |  |
| 652 | | 0.65476 | | 0.57426 | | 0.51061 | | 0.45025 |  |
| 652.5 | | 0.6523 | | 0.57294 | | 0.50984 | | 0.44987 |  |
| 653 | | 0.64986 | | 0.57139 | | 0.50889 | | 0.44951 |  |
| 653.5 | | 0.64724 | | 0.56987 | | 0.50773 | | 0.44921 |  |
| 654 | | 0.64465 | | 0.5683 | | 0.50643 | | 0.44887 |  |
| 654.5 | | 0.64218 | | 0.56672 | | 0.50519 | | 0.44862 |  |
| 655 | | 0.63979 | | 0.56499 | | 0.50402 | | 0.44836 |  |
| 655.5 | | 0.63749 | | 0.56335 | | 0.50293 | | 0.44819 |  |
| 656 | | 0.63506 | | 0.56186 | | 0.502 | | 0.44795 |  |
| 656.5 | | 0.63267 | | 0.5603 | | 0.50096 | | 0.44764 |  |
| 657 | | 0.63041 | | 0.55875 | | 0.50014 | | 0.44734 |  |
| 657.5 | | 0.62818 | | 0.55721 | | 0.4992 | | 0.44721 |  |
| 658 | | 0.62631 | | 0.55588 | | 0.49842 | | 0.44708 |  |
| 658.5 | | 0.62442 | | 0.55471 | | 0.4976 | | 0.44694 |  |
| 659 | | 0.62269 | | 0.5537 | | 0.49686 | | 0.44667 |  |
| 659.5 | | 0.62091 | | 0.55259 | | 0.49615 | | 0.4465 |  |
| 660 | | 0.6192 | | 0.55151 | | 0.4955 | | 0.44638 |  |
| 660.5 | | 0.61751 | | 0.55007 | | 0.49454 | | 0.44634 |  |
| 661 | | 0.61597 | | 0.54874 | | 0.49368 | | 0.44621 |  |
| 661.5 | | 0.6143 | | 0.54745 | | 0.49279 | | 0.44589 |  |
| 662 | | 0.61249 | | 0.54643 | | 0.49219 | | 0.44549 |  |
| 662.5 | | 0.61051 | | 0.54537 | | 0.4916 | | 0.44504 |  |
| 663 | | 0.60855 | | 0.54426 | | 0.49083 | | 0.44472 |  |
| 663.5 | | 0.60645 | | 0.54332 | | 0.49008 | | 0.44449 |  |
| 664 | | 0.60432 | | 0.54227 | | 0.48924 | | 0.44428 |  |
| 664.5 | | 0.60223 | | 0.54114 | | 0.48821 | | 0.44427 |  |
| 665 | | 0.60016 | | 0.53991 | | 0.48718 | | 0.44409 |  |
| 665.5 | | 0.59807 | | 0.5388 | | 0.4861 | | 0.44385 |  |
| 666 | | 0.59596 | | 0.53759 | | 0.48525 | | 0.44336 |  |
| 666.5 | | 0.59402 | | 0.5363 | | 0.48426 | | 0.44301 |  |
| 667 | | 0.59207 | | 0.53508 | | 0.48346 | | 0.44255 |  |
| 667.5 | | 0.59032 | | 0.53362 | | 0.48252 | | 0.44224 |  |
| 668 | | 0.58856 | | 0.53218 | | 0.48172 | | 0.44186 |  |
| 668.5 | | 0.58685 | | 0.5305 | | 0.4808 | | 0.44159 |  |
| 669 | | 0.58506 | | 0.52892 | | 0.47991 | | 0.44136 |  |
| 669.5 | | 0.58324 | | 0.5271 | | 0.47912 | | 0.44131 |  |
| 670 | | 0.58154 | | 0.52523 | | 0.47828 | | 0.44128 |  |
| 670.5 | | 0.57998 | | 0.52322 | | 0.47734 | | 0.44113 |  |
| 671 | | 0.57859 | | 0.52166 | | 0.47649 | | 0.44073 |  |
| 671.5 | | 0.57701 | | 0.52019 | | 0.47578 | | 0.44033 |  |
| 672 | | 0.57541 | | 0.51896 | | 0.47515 | | 0.43995 |  |
| 672.5 | | 0.57365 | | 0.51745 | | 0.47424 | | 0.43973 |  |
| 673 | | 0.57185 | | 0.51611 | | 0.47325 | | 0.4396 |  |
| 673.5 | | 0.57013 | | 0.51477 | | 0.47241 | | 0.43949 |  |
| 674 | | 0.56832 | | 0.51345 | | 0.47149 | | 0.43918 |  |
| 674.5 | | 0.56665 | | 0.51207 | | 0.47058 | | 0.43885 |  |
| 675 | | 0.56495 | | 0.51069 | | 0.46982 | | 0.43819 |  |
| 675.5 | | 0.56325 | | 0.50919 | | 0.46903 | | 0.43769 |  |
| 676 | | 0.56144 | | 0.50766 | | 0.46812 | | 0.43703 |  |
| 676.5 | | 0.55952 | | 0.50614 | | 0.46689 | | 0.43647 |  |
| 677 | | 0.5574 | | 0.50472 | | 0.46581 | | 0.43584 |  |
| 677.5 | | 0.55522 | | 0.50313 | | 0.46472 | | 0.43544 |  |
| 678 | | 0.55301 | | 0.5015 | | 0.46389 | | 0.43515 |  |
| 678.5 | | 0.55083 | | 0.49998 | | 0.46288 | | 0.435 |  |
| 679 | | 0.54862 | | 0.49883 | | 0.4617 | | 0.43472 |  |
| 679.5 | | 0.54647 | | 0.49763 | | 0.46039 | | 0.43437 |  |
| 680 | | 0.54435 | | 0.49648 | | 0.45914 | | 0.4339 |  |
| 680.5 | | 0.54221 | | 0.49524 | | 0.45804 | | 0.43328 |  |
| 681 | | 0.54001 | | 0.49421 | | 0.45693 | | 0.43254 |  |
| 681.5 | | 0.5378 | | 0.49322 | | 0.45598 | | 0.43203 |  |
| 682 | | 0.53579 | | 0.49216 | | 0.45496 | | 0.43157 |  |
| 682.5 | | 0.53381 | | 0.49097 | | 0.45401 | | 0.4313 |  |
| 683 | | 0.532 | | 0.49 | | 0.45319 | | 0.43098 |  |
| 683.5 | | 0.53042 | | 0.48893 | | 0.45258 | | 0.43057 |  |
| 684 | | 0.52859 | | 0.48771 | | 0.45195 | | 0.42997 |  |
| 684.5 | | 0.5269 | | 0.48612 | | 0.45144 | | 0.4294 |  |
| 685 | | 0.52515 | | 0.48447 | | 0.45083 | | 0.42876 |  |
| 685.5 | | 0.52358 | | 0.48295 | | 0.44999 | | 0.42818 |  |
| 686 | | 0.52191 | | 0.48159 | | 0.44864 | | 0.42741 |  |
| 686.5 | | 0.52016 | | 0.48027 | | 0.44749 | | 0.42667 |  |
| 687 | | 0.51833 | | 0.47897 | | 0.44654 | | 0.42613 |  |
| 687.5 | | 0.51657 | | 0.47759 | | 0.44574 | | 0.42568 |  |
| 688 | | 0.51487 | | 0.47596 | | 0.44475 | | 0.4252 |  |
| 688.5 | | 0.51316 | | 0.47456 | | 0.44348 | | 0.42455 |  |
| 689 | | 0.51142 | | 0.47318 | | 0.44231 | | 0.4238 |  |
| 689.5 | | 0.50961 | | 0.47177 | | 0.44114 | | 0.42324 |  |
| 690 | | 0.50787 | | 0.4701 | | 0.44032 | | 0.42271 |  |
| 690.5 | | 0.50633 | | 0.46865 | | 0.43957 | | 0.42222 |  |
| 691 | | 0.50481 | | 0.46739 | | 0.43893 | | 0.42182 |  |
| 691.5 | | 0.50335 | | 0.46599 | | 0.43827 | | 0.42158 |  |
| 692 | | 0.50178 | | 0.46439 | | 0.43749 | | 0.42143 |  |
| 692.5 | | 0.50032 | | 0.46268 | | 0.43671 | | 0.42129 |  |
| 693 | | 0.49868 | | 0.46138 | | 0.43592 | | 0.42096 |  |
| 693.5 | | 0.49698 | | 0.45997 | | 0.43515 | | 0.42053 |  |
| 694 | | 0.4951 | | 0.45874 | | 0.43432 | | 0.42004 |  |
| 694.5 | | 0.49341 | | 0.45737 | | 0.43358 | | 0.4196 |  |
| 695 | | 0.49161 | | 0.45625 | | 0.43274 | | 0.41906 |  |
| 695.5 | | 0.49004 | | 0.45516 | | 0.43183 | | 0.4186 |  |
| 696 | | 0.48853 | | 0.45424 | | 0.43081 | | 0.41802 |  |
| 696.5 | | 0.48699 | | 0.45333 | | 0.43013 | | 0.41763 |  |
| 697 | | 0.48538 | | 0.45248 | | 0.42945 | | 0.41718 |  |
| 697.5 | | 0.48366 | | 0.45156 | | 0.42896 | | 0.41662 |  |
| 698 | | 0.48227 | | 0.45057 | | 0.42826 | | 0.41609 |  |
| 698.5 | | 0.48088 | | 0.44968 | | 0.42785 | | 0.41557 |  |
| 699 | | 0.47955 | | 0.44853 | | 0.42761 | | 0.41514 |  |
| 699.5 | | 0.47814 | | 0.44747 | | 0.4275 | | 0.41467 |  |
| 700 | | 0.47686 | | 0.44621 | | 0.42712 | | 0.41408 |  |
| 700.5 | | 0.47572 | | 0.44517 | | 0.42653 | | 0.41367 |  |
| 701 | | 0.47446 | | 0.44386 | | 0.4259 | | 0.4135 |  |
| 701.5 | | 0.47319 | | 0.44252 | | 0.42501 | | 0.41321 |  |
| 702 | | 0.47168 | | 0.44126 | | 0.42396 | | 0.41276 |  |
| 702.5 | | 0.47003 | | 0.4402 | | 0.42269 | | 0.41203 |  |
| 703 | | 0.46834 | | 0.43943 | | 0.42139 | | 0.41137 |  |
| 703.5 | | 0.46666 | | 0.43857 | | 0.42006 | | 0.41087 |  |
| 704 | | 0.46511 | | 0.43773 | | 0.41875 | | 0.41044 |  |
| 704.5 | | 0.46358 | | 0.43679 | | 0.41752 | | 0.41017 |  |
| 705 | | 0.46217 | | 0.43598 | | 0.41645 | | 0.40986 |  |
| 705.5 | | 0.46096 | | 0.4351 | | 0.4153 | | 0.40982 |  |
| 706 | | 0.45978 | | 0.43429 | | 0.41423 | | 0.40967 |  |
| 706.5 | | 0.45855 | | 0.43336 | | 0.41336 | | 0.40945 |  |
| 707 | | 0.45734 | | 0.43242 | | 0.41274 | | 0.40894 |  |
| 707.5 | | 0.45607 | | 0.43134 | | 0.41221 | | 0.40832 |  |
| 708 | | 0.45494 | | 0.43026 | | 0.41149 | | 0.40783 |  |
| 708.5 | | 0.45348 | | 0.42906 | | 0.41094 | | 0.40716 |  |
| 709 | | 0.45207 | | 0.42773 | | 0.41043 | | 0.40655 |  |
| 709.5 | | 0.45037 | | 0.42643 | | 0.4099 | | 0.40583 |  |
| 710 | | 0.44878 | | 0.42496 | | 0.40891 | | 0.40541 |  |
| 710.5 | | 0.44726 | | 0.42351 | | 0.40808 | | 0.40505 |  |
| 711 | | 0.44581 | | 0.42222 | | 0.40733 | | 0.40464 |  |
| 711.5 | | 0.44435 | | 0.42123 | | 0.40667 | | 0.40391 |  |
| 712 | | 0.44274 | | 0.42028 | | 0.40584 | | 0.40307 |  |
| 712.5 | | 0.4412 | | 0.41928 | | 0.40498 | | 0.40241 |  |
| 713 | | 0.43984 | | 0.41826 | | 0.40439 | | 0.40198 |  |
| 713.5 | | 0.43846 | | 0.41744 | | 0.40386 | | 0.4017 |  |
| 714 | | 0.43718 | | 0.41665 | | 0.40338 | | 0.40125 |  |
| 714.5 | | 0.43592 | | 0.41618 | | 0.40299 | | 0.40091 |  |
| 715 | | 0.43462 | | 0.41569 | | 0.40261 | | 0.40044 |  |
| 715.5 | | 0.43325 | | 0.41517 | | 0.40202 | | 0.40007 |  |
| 716 | | 0.43171 | | 0.41465 | | 0.40138 | | 0.39953 |  |
| 716.5 | | 0.43024 | | 0.41421 | | 0.40085 | | 0.39875 |  |
| 717 | | 0.42869 | | 0.41378 | | 0.40029 | | 0.398 |  |
| 717.5 | | 0.42708 | | 0.41326 | | 0.39961 | | 0.39725 |  |
| 718 | | 0.42547 | | 0.41283 | | 0.39882 | | 0.39684 |  |
| 718.5 | | 0.42373 | | 0.41246 | | 0.39796 | | 0.39647 |  |
| 719 | | 0.42198 | | 0.4119 | | 0.39731 | | 0.39625 |  |
| 719.5 | | 0.42011 | | 0.41119 | | 0.39659 | | 0.39595 |  |
| 720 | | 0.41845 | | 0.41038 | | 0.39575 | | 0.39579 |  |
| 720.5 | | 0.4168 | | 0.40957 | | 0.39499 | | 0.39549 |  |
| 721 | | 0.41527 | | 0.40876 | | 0.39431 | | 0.39521 |  |
| 721.5 | | 0.4136 | | 0.4079 | | 0.39376 | | 0.39489 |  |
| 722 | | 0.41202 | | 0.40699 | | 0.39312 | | 0.39462 |  |
| 722.5 | | 0.41055 | | 0.40605 | | 0.3926 | | 0.39447 |  |
| 723 | | 0.4091 | | 0.40514 | | 0.39219 | | 0.39418 |  |
| 723.5 | | 0.40775 | | 0.40433 | | 0.39177 | | 0.39372 |  |
| 724 | | 0.40637 | | 0.40358 | | 0.39138 | | 0.39301 |  |
| 724.5 | | 0.40506 | | 0.40287 | | 0.39101 | | 0.39238 |  |
| 725 | | 0.40375 | | 0.40214 | | 0.39053 | | 0.39182 |  |
| 725.5 | | 0.40256 | | 0.40153 | | 0.39 | | 0.39125 |  |
| 726 | | 0.40129 | | 0.40093 | | 0.38945 | | 0.39072 |  |
| 726.5 | | 0.40013 | | 0.40043 | | 0.38896 | | 0.39003 |  |
| 727 | | 0.39904 | | 0.39998 | | 0.38847 | | 0.38955 |  |
| 727.5 | | 0.39809 | | 0.39967 | | 0.38794 | | 0.38895 |  |
| 728 | | 0.39706 | | 0.39939 | | 0.38749 | | 0.38848 |  |
| 728.5 | | 0.39592 | | 0.39894 | | 0.38687 | | 0.38789 |  |
| 729 | | 0.39486 | | 0.39846 | | 0.38624 | | 0.38738 |  |
| 729.5 | | 0.39387 | | 0.39791 | | 0.3855 | | 0.38705 |  |
| 730 | | 0.39301 | | 0.39741 | | 0.38487 | | 0.38681 |  |
| 730.5 | | 0.39197 | | 0.39674 | | 0.38463 | | 0.38646 |  |
| 731 | | 0.39108 | | 0.39601 | | 0.38447 | | 0.38614 |  |
| 731.5 | | 0.39015 | | 0.39525 | | 0.38443 | | 0.3857 |  |
| 732 | | 0.38939 | | 0.39441 | | 0.3844 | | 0.38524 |  |
| 732.5 | | 0.38866 | | 0.39348 | | 0.38431 | | 0.38475 |  |
| 733 | | 0.38809 | | 0.39264 | | 0.38412 | | 0.38433 |  |
| 733.5 | | 0.38766 | | 0.39185 | | 0.38392 | | 0.38403 |  |
| 734 | | 0.38725 | | 0.39112 | | 0.38378 | | 0.38391 |  |
| 734.5 | | 0.38689 | | 0.39024 | | 0.38373 | | 0.38392 |  |
| 735 | | 0.38661 | | 0.38951 | | 0.38359 | | 0.38394 |  |
| 735.5 | | 0.38631 | | 0.38889 | | 0.38332 | | 0.38374 |  |
| 736 | | 0.38574 | | 0.38839 | | 0.38295 | | 0.38348 |  |
| 736.5 | | 0.38511 | | 0.38802 | | 0.38263 | | 0.38312 |  |
| 737 | | 0.38446 | | 0.38772 | | 0.38243 | | 0.38296 |  |
| 737.5 | | 0.38384 | | 0.38762 | | 0.38214 | | 0.38278 |  |
| 738 | | 0.38329 | | 0.38741 | | 0.38187 | | 0.38264 |  |
| 738.5 | | 0.38297 | | 0.38716 | | 0.38139 | | 0.38248 |  |
| 739 | | 0.38264 | | 0.38691 | | 0.3809 | | 0.38236 |  |
| 739.5 | | 0.3822 | | 0.38665 | | 0.38055 | | 0.38213 |  |
| 740 | | 0.38167 | | 0.38636 | | 0.38033 | | 0.38196 |  |
| 740.5 | | 0.38105 | | 0.38585 | | 0.38017 | | 0.38171 |  |
| 741 | | 0.3806 | | 0.38525 | | 0.37996 | | 0.38162 |  |
| 741.5 | | 0.38012 | | 0.3846 | | 0.37979 | | 0.38145 |  |
| 742 | | 0.37987 | | 0.38397 | | 0.37961 | | 0.38131 |  |
| 742.5 | | 0.37967 | | 0.38342 | | 0.37923 | | 0.38112 |  |
| 743 | | 0.37963 | | 0.38295 | | 0.37877 | | 0.38091 |  |
| 743.5 | | 0.3796 | | 0.3825 | | 0.37841 | | 0.38073 |  |
| 744 | | 0.37947 | | 0.38196 | | 0.37818 | | 0.38072 |  |
| 744.5 | | 0.37919 | | 0.38131 | | 0.37815 | | 0.38072 |  |
| 745 | | 0.37891 | | 0.38068 | | 0.37811 | | 0.38047 |  |
| 745.5 | | 0.37853 | | 0.38019 | | 0.37809 | | 0.3801 |  |
| 746 | | 0.37782 | | 0.37976 | | 0.37815 | | 0.37963 |  |
| 746.5 | | 0.37712 | | 0.37934 | | 0.37831 | | 0.3792 |  |
| 747 | | 0.37643 | | 0.37895 | | 0.37842 | | 0.37881 |  |
| 747.5 | | 0.37595 | | 0.37872 | | 0.37846 | | 0.37853 |  |
| 748 | | 0.37544 | | 0.37848 | | 0.37845 | | 0.37803 |  |
| 748.5 | | 0.37508 | | 0.37837 | | 0.37838 | | 0.37757 |  |
| 749 | | 0.37462 | | 0.37827 | | 0.37831 | | 0.37703 |  |
| 749.5 | | 0.37423 | | 0.37831 | | 0.3783 | | 0.37665 |  |
| 750 | | 0.37397 | | 0.37811 | | 0.37837 | | 0.37644 |  |
| 750.5 | | 0.37375 | | 0.37775 | | 0.37829 | | 0.37632 |  |
| 751 | | 0.37363 | | 0.3774 | | 0.3782 | | 0.37632 |  |
| 751.5 | | 0.3736 | | 0.37733 | | 0.37804 | | 0.37617 |  |
| 752 | | 0.37371 | | 0.37727 | | 0.37794 | | 0.37581 |  |
| 752.5 | | 0.3737 | | 0.37727 | | 0.37767 | | 0.37541 |  |
| 753 | | 0.37354 | | 0.37719 | | 0.37737 | | 0.3751 |  |
| 753.5 | | 0.37339 | | 0.37724 | | 0.37715 | | 0.37502 |  |
| 754 | | 0.37319 | | 0.37704 | | 0.37702 | | 0.37509 |  |
| 754.5 | | 0.37299 | | 0.37674 | | 0.37698 | | 0.37504 |  |
| 755 | | 0.37294 | | 0.37643 | | 0.37688 | | 0.37498 |  |
| 755.5 | | 0.37288 | | 0.37632 | | 0.37663 | | 0.37499 |  |
| 756 | | 0.3726 | | 0.37616 | | 0.37647 | | 0.3748 |  |
| 756.5 | | 0.37233 | | 0.37592 | | 0.37621 | | 0.37471 |  |
| 757 | | 0.37202 | | 0.37564 | | 0.37597 | | 0.37463 |  |
| 757.5 | | 0.37189 | | 0.37546 | | 0.37575 | | 0.37458 |  |
| 758 | | 0.37172 | | 0.37532 | | 0.3754 | | 0.37429 |  |
| 758.5 | | 0.37175 | | 0.37511 | | 0.37512 | | 0.37401 |  |
| 759 | | 0.3718 | | 0.37469 | | 0.37488 | | 0.37381 |  |
| 759.5 | | 0.37184 | | 0.37408 | | 0.37473 | | 0.37365 |  |
| 760 | | 0.37189 | | 0.37349 | | 0.37448 | | 0.37341 |  |
| 760.5 | | 0.37174 | | 0.37304 | | 0.37408 | | 0.37331 |  |
| 761 | | 0.37175 | | 0.37279 | | 0.37377 | | 0.37304 |  |
| 761.5 | | 0.37173 | | 0.37274 | | 0.37368 | | 0.37258 |  |
| 762 | | 0.37174 | | 0.37271 | | 0.37355 | | 0.37214 |  |
| 762.5 | | 0.3717 | | 0.37263 | | 0.37339 | | 0.37175 |  |
| 763 | | 0.37158 | | 0.37249 | | 0.37313 | | 0.37153 |  |
| 763.5 | | 0.37165 | | 0.37244 | | 0.37297 | | 0.37126 |  |
| 764 | | 0.37167 | | 0.37226 | | 0.37279 | | 0.3711 |  |
| 764.5 | | 0.37175 | | 0.37205 | | 0.37261 | | 0.37103 |  |
| 765 | | 0.37182 | | 0.37201 | | 0.3725 | | 0.37099 |  |
| 765.5 | | 0.37173 | | 0.37206 | | 0.37237 | | 0.37076 |  |
| 766 | | 0.37143 | | 0.3721 | | 0.37252 | | 0.37042 |  |
| 766.5 | | 0.37121 | | 0.37214 | | 0.37257 | | 0.3702 |  |
| 767 | | 0.37106 | | 0.37225 | | 0.37278 | | 0.37003 |  |
| 767.5 | | 0.37103 | | 0.37252 | | 0.37289 | | 0.3699 |  |
| 768 | | 0.37096 | | 0.37279 | | 0.37293 | | 0.36976 |  |
| 768.5 | | 0.37086 | | 0.37292 | | 0.37291 | | 0.36963 |  |
| 769 | | 0.37082 | | 0.37295 | | 0.37292 | | 0.36949 |  |
| 769.5 | | 0.37082 | | 0.37282 | | 0.37288 | | 0.36929 |  |
| 770 | | 0.37087 | | 0.37278 | | 0.37266 | | 0.36911 |  |
| 770.5 | | 0.37088 | | 0.37279 | | 0.37238 | | 0.36888 |  |
| 771 | | 0.37107 | | 0.37282 | | 0.37223 | | 0.36843 |  |
| 771.5 | | 0.3713 | | 0.37282 | | 0.37223 | | 0.36788 |  |
| 772 | | 0.37156 | | 0.37277 | | 0.37197 | | 0.36763 |  |
| 772.5 | | 0.37181 | | 0.37258 | | 0.3718 | | 0.36735 |  |
| 773 | | 0.37194 | | 0.37235 | | 0.37153 | | 0.36737 |  |
| 773.5 | | 0.37216 | | 0.37195 | | 0.37133 | | 0.36722 |  |
| 774 | | 0.37236 | | 0.37143 | | 0.37107 | | 0.3671 |  |
| 774.5 | | 0.37259 | | 0.37092 | | 0.37085 | | 0.36689 |  |
| 775 | | 0.37272 | | 0.3706 | | 0.37084 | | 0.36651 |  |
| 775.5 | | 0.37279 | | 0.37026 | | 0.37077 | | 0.36618 |  |
| 776 | | 0.37277 | | 0.36995 | | 0.37098 | | 0.36581 |  |
| 776.5 | | 0.37269 | | 0.36969 | | 0.37108 | | 0.36566 |  |
| 777 | | 0.37248 | | 0.3695 | | 0.37121 | | 0.36561 |  |
| 777.5 | | 0.37217 | | 0.36944 | | 0.37123 | | 0.36564 |  |
| 778 | | 0.37186 | | 0.36937 | | 0.37126 | | 0.36556 |  |
| 778.5 | | 0.37165 | | 0.3693 | | 0.37131 | | 0.36533 |  |
| 779 | | 0.37165 | | 0.3693 | | 0.37137 | | 0.36511 |  |
| 779.5 | | 0.37178 | | 0.36933 | | 0.37123 | | 0.365 |  |
| 780 | | 0.372 | | 0.36962 | | 0.37103 | | 0.36496 |  |
| 780.5 | | 0.3721 | | 0.37004 | | 0.37082 | | 0.36484 |  |
| 781 | | 0.37221 | | 0.37047 | | 0.37074 | | 0.36462 |  |
| 781.5 | | 0.3724 | | 0.37077 | | 0.37076 | | 0.36455 |  |
| 782 | | 0.37263 | | 0.37109 | | 0.37065 | | 0.36457 |  |
| 782.5 | | 0.37294 | | 0.37141 | | 0.37062 | | 0.36456 |  |
| 783 | | 0.37311 | | 0.3717 | | 0.37051 | | 0.36444 |  |
| 783.5 | | 0.37342 | | 0.37192 | | 0.37054 | | 0.36419 |  |
| 784 | | 0.37386 | | 0.37212 | | 0.37049 | | 0.36379 |  |
| 784.5 | | 0.37438 | | 0.37232 | | 0.37041 | | 0.36347 |  |
| 785 | | 0.37492 | | 0.37238 | | 0.37052 | | 0.36314 |  |
| 785.5 | | 0.37535 | | 0.37221 | | 0.37071 | | 0.3629 |  |
| 786 | | 0.37566 | | 0.37194 | | 0.3709 | | 0.36262 |  |
| 786.5 | | 0.3757 | | 0.3716 | | 0.37088 | | 0.36257 |  |
| 787 | | 0.37585 | | 0.37125 | | 0.37085 | | 0.36255 |  |
| 787.5 | | 0.37592 | | 0.37094 | | 0.37096 | | 0.36259 |  |
| 788 | | 0.37618 | | 0.3708 | | 0.37101 | | 0.36257 |  |
| 788.5 | | 0.37639 | | 0.37084 | | 0.37098 | | 0.36248 |  |
| 789 | | 0.37673 | | 0.37107 | | 0.3708 | | 0.36232 |  |
| 789.5 | | 0.37694 | | 0.37135 | | 0.37061 | | 0.36216 |  |
| 790 | | 0.37714 | | 0.37172 | | 0.37068 | | 0.36211 |  |
| 790.5 | | 0.37711 | | 0.37194 | | 0.37087 | | 0.36194 |  |
| 791 | | 0.37719 | | 0.37215 | | 0.37114 | | 0.36181 |  |
| 791.5 | | 0.37726 | | 0.3723 | | 0.37128 | | 0.36176 |  |
| 792 | | 0.37735 | | 0.37229 | | 0.3713 | | 0.3617 |  |
| 792.5 | | 0.37737 | | 0.37247 | | 0.37147 | | 0.36163 |  |
| 793 | | 0.37734 | | 0.37269 | | 0.37174 | | 0.36145 |  |
| 793.5 | | 0.37747 | | 0.37304 | | 0.37209 | | 0.36132 |  |
| 794 | | 0.37771 | | 0.37327 | | 0.37233 | | 0.36111 |  |
| 794.5 | | 0.37804 | | 0.3735 | | 0.37264 | | 0.36099 |  |
| 795 | | 0.37842 | | 0.37378 | | 0.37298 | | 0.36071 |  |
| 795.5 | | 0.37881 | | 0.374 | | 0.37325 | | 0.36034 |  |
| 796 | | 0.3791 | | 0.37422 | | 0.37309 | | 0.36005 |  |
| 796.5 | | 0.37918 | | 0.37439 | | 0.37289 | | 0.35992 |  |
| 797 | | 0.37943 | | 0.37468 | | 0.3727 | | 0.35984 |  |
| 797.5 | | 0.37958 | | 0.3749 | | 0.37261 | | 0.35963 |  |
| 798 | | 0.38002 | | 0.37506 | | 0.37254 | | 0.35931 |  |
| 798.5 | | 0.3802 | | 0.37521 | | 0.37244 | | 0.35897 |  |
| 799 | | 0.38052 | | 0.37543 | | 0.37244 | | 0.35861 |  |
| 799.5 | | 0.3806 | | 0.3757 | | 0.37252 | | 0.35832 |  |
| 800 | | 0.38083 | | 0.3758 | | 0.37267 | | 0.35814 |  |
| 800.5 | | 0.38109 | | 0.37578 | | 0.37268 | | 0.35809 |  |
| 801 | | 0.38137 | | 0.37583 | | 0.37258 | | 0.35834 |  |
| 801.5 | | 0.38167 | | 0.37579 | | 0.37232 | | 0.35876 |  |
| 802 | | 0.38201 | | 0.37572 | | 0.37241 | | 0.35909 |  |
| 802.5 | | 0.38245 | | 0.37585 | | 0.37264 | | 0.35937 |  |
| 803 | | 0.38277 | | 0.37599 | | 0.37288 | | 0.35956 |  |
| 803.5 | | 0.38307 | | 0.37621 | | 0.37318 | | 0.35978 |  |
| 804 | | 0.38345 | | 0.37633 | | 0.37339 | | 0.35984 |  |
| 804.5 | | 0.38411 | | 0.37644 | | 0.37363 | | 0.35981 |  |
| 805 | | 0.38465 | | 0.37651 | | 0.37366 | | 0.35964 |  |
| 805.5 | | 0.38514 | | 0.37643 | | 0.37357 | | 0.3595 |  |
| 806 | | 0.38554 | | 0.37649 | | 0.37334 | | 0.35935 |  |
| 806.5 | | 0.38591 | | 0.37647 | | 0.37335 | | 0.35913 |  |
| 807 | | 0.38638 | | 0.37652 | | 0.37343 | | 0.35897 |  |
| 807.5 | | 0.38689 | | 0.37647 | | 0.37361 | | 0.3586 |  |
| 808 | | 0.38752 | | 0.37654 | | 0.37375 | | 0.35831 |  |
| 808.5 | | 0.3879 | | 0.37669 | | 0.37369 | | 0.35795 |  |
| 809 | | 0.38829 | | 0.37692 | | 0.37355 | | 0.35755 |  |
| 809.5 | | 0.38858 | | 0.37732 | | 0.37347 | | 0.35708 |  |
| 810 | | 0.38916 | | 0.37768 | | 0.37331 | | 0.35684 |  |
| 810.5 | | 0.38974 | | 0.37815 | | 0.37323 | | 0.35685 |  |
| 811 | | 0.39026 | | 0.37849 | | 0.37315 | | 0.35707 |  |
| 811.5 | | 0.39065 | | 0.37891 | | 0.37318 | | 0.3572 |  |
| 812 | | 0.39089 | | 0.37931 | | 0.37335 | | 0.35722 |  |
| 812.5 | | 0.39116 | | 0.37981 | | 0.37361 | | 0.35727 |  |
| 813 | | 0.39144 | | 0.38019 | | 0.37397 | | 0.35735 |  |
| 813.5 | | 0.39183 | | 0.38066 | | 0.37449 | | 0.35753 |  |
| 814 | | 0.39235 | | 0.38107 | | 0.37484 | | 0.35763 |  |
| 814.5 | | 0.39274 | | 0.38148 | | 0.37515 | | 0.35765 |  |
| 815 | | 0.39302 | | 0.38172 | | 0.37534 | | 0.35764 |  |
| 815.5 | | 0.39336 | | 0.38184 | | 0.37553 | | 0.35763 |  |
| 816 | | 0.39382 | | 0.38205 | | 0.3757 | | 0.35754 |  |
| 816.5 | | 0.39429 | | 0.38202 | | 0.37588 | | 0.35757 |  |
| 817 | | 0.39468 | | 0.38214 | | 0.37591 | | 0.35765 |  |
| 817.5 | | 0.39511 | | 0.38219 | | 0.37598 | | 0.35756 |  |
| 818 | | 0.39539 | | 0.38238 | | 0.376 | | 0.35749 |  |
| 818.5 | | 0.39567 | | 0.3825 | | 0.37595 | | 0.3573 |  |
| 819 | | 0.39589 | | 0.38275 | | 0.37592 | | 0.35716 |  |
| 819.5 | | 0.39613 | | 0.383 | | 0.37586 | | 0.35706 |  |
| 820 | | 0.3965 | | 0.38329 | | 0.37568 | | 0.35728 |  |
| 820.5 | | 0.39689 | | 0.38331 | | 0.37559 | | 0.35751 |  |
| 821 | | 0.39712 | | 0.38333 | | 0.37552 | | 0.35764 |  |
| 821.5 | | 0.39719 | | 0.38353 | | 0.3757 | | 0.35756 |  |
| 822 | | 0.39735 | | 0.3838 | | 0.37582 | | 0.35752 |  |
| 822.5 | | 0.39789 | | 0.38416 | | 0.37614 | | 0.35745 |  |
| 823 | | 0.3985 | | 0.38442 | | 0.37642 | | 0.35731 |  |
| 823.5 | | 0.39896 | | 0.38488 | | 0.37688 | | 0.35693 |  |
| 824 | | 0.39923 | | 0.38528 | | 0.37714 | | 0.3565 |  |
| 824.5 | | 0.39947 | | 0.38559 | | 0.37736 | | 0.35608 |  |
| 825 | | 0.39996 | | 0.3857 | | 0.37755 | | 0.35587 |  |
| 825.5 | | 0.40047 | | 0.38588 | | 0.3777 | | 0.35563 |  |
| 826 | | 0.40099 | | 0.38613 | | 0.3778 | | 0.35538 |  |
| 826.5 | | 0.4014 | | 0.38642 | | 0.37783 | | 0.35529 |  |
| 827 | | 0.40178 | | 0.38688 | | 0.37794 | | 0.35518 |  |
| 827.5 | | 0.40221 | | 0.38735 | | 0.37808 | | 0.35516 |  |
| 828 | | 0.40271 | | 0.3879 | | 0.37828 | | 0.35515 |  |
| 828.5 | | 0.40339 | | 0.38833 | | 0.37831 | | 0.35516 |  |
| 829 | | 0.40409 | | 0.3887 | | 0.37828 | | 0.35519 |  |
| 829.5 | | 0.40472 | | 0.3889 | | 0.37826 | | 0.35528 |  |
| 830 | | 0.40527 | | 0.38901 | | 0.37839 | | 0.35554 |  |
| 830.5 | | 0.40577 | | 0.38872 | | 0.37858 | | 0.35569 |  |
| 831 | | 0.4062 | | 0.38859 | | 0.37868 | | 0.35577 |  |
| 831.5 | | 0.40669 | | 0.38858 | | 0.37885 | | 0.35583 |  |
| 832 | | 0.40728 | | 0.38867 | | 0.37909 | | 0.35587 |  |
| 832.5 | | 0.40799 | | 0.38872 | | 0.37952 | | 0.3558 |  |
| 833 | | 0.40839 | | 0.38874 | | 0.37992 | | 0.35569 |  |
| 833.5 | | 0.40877 | | 0.38899 | | 0.38024 | | 0.35552 |  |
| 834 | | 0.40897 | | 0.38914 | | 0.38033 | | 0.35551 |  |
| 834.5 | | 0.40936 | | 0.38911 | | 0.38039 | | 0.35538 |  |
| 835 | | 0.40951 | | 0.38902 | | 0.38059 | | 0.35516 |  |
| 835.5 | | 0.40968 | | 0.38928 | | 0.3807 | | 0.3549 |  |
| 836 | | 0.41018 | | 0.3898 | | 0.38067 | | 0.35471 |  |
| 836.5 | | 0.41101 | | 0.39027 | | 0.38051 | | 0.35449 |  |
| 837 | | 0.4119 | | 0.3904 | | 0.38044 | | 0.3541 |  |
| 837.5 | | 0.41247 | | 0.39043 | | 0.38033 | | 0.35373 |  |
| 838 | | 0.4129 | | 0.39062 | | 0.38022 | | 0.35331 |  |
| 838.5 | | 0.41329 | | 0.39095 | | 0.37996 | | 0.353 |  |
| 839 | | 0.41354 | | 0.39131 | | 0.37994 | | 0.35284 |  |
| 839.5 | | 0.41361 | | 0.3916 | | 0.38004 | | 0.35275 |  |
| 840 | | 0.41365 | | 0.39195 | | 0.3804 | | 0.35287 |  |
| 840.5 | | 0.41378 | | 0.39254 | | 0.38058 | | 0.35292 |  |
| 841 | | 0.41392 | | 0.3935 | | 0.38093 | | 0.35298 |  |
| 841.5 | | 0.41422 | | 0.39431 | | 0.38152 | | 0.3532 |  |
| 842 | | 0.41476 | | 0.39468 | | 0.38217 | | 0.35349 |  |
| 842.5 | | 0.41522 | | 0.39471 | | 0.38278 | | 0.3539 |  |
| 843 | | 0.41561 | | 0.39458 | | 0.38328 | | 0.35435 |  |
| 843.5 | | 0.41597 | | 0.39426 | | 0.38397 | | 0.35461 |  |
| 844 | | 0.41625 | | 0.39413 | | 0.38458 | | 0.35498 |  |
| 844.5 | | 0.41657 | | 0.3939 | | 0.38517 | | 0.3551 |  |
| 845 | | 0.41687 | | 0.39407 | | 0.38581 | | 0.35531 |  |
| 845.5 | | 0.4179 | | 0.39444 | | 0.38626 | | 0.35547 |  |
| 846 | | 0.41939 | | 0.39512 | | 0.38653 | | 0.3558 |  |
| 846.5 | | 0.42085 | | 0.39507 | | 0.38663 | | 0.35571 |  |
| 847 | | 0.42174 | | 0.39436 | | 0.38672 | | 0.355 |  |
| 847.5 | | 0.42234 | | 0.39396 | | 0.38668 | | 0.35388 |  |
| 848 | | 0.42286 | | 0.39415 | | 0.38653 | | 0.35301 |  |
| 848.5 | | 0.42332 | | 0.39442 | | 0.38632 | | 0.3525 |  |
| 849 | | 0.42361 | | 0.39493 | | 0.3862 | | 0.35187 |  |
| 849.5 | | 0.42386 | | 0.39578 | | 0.38589 | | 0.35131 |  |
| 850 | | 0.42405 | | 0.39665 | | 0.3858 | | 0.35104 |  |
| 850.5 | | 0.4239 | | 0.3972 | | 0.38574 | | 0.35084 |  |
| 851 | | 0.42366 | | 0.3974 | | 0.38609 | | 0.35062 |  |
| 851.5 | | 0.4235 | | 0.39759 | | 0.38636 | | 0.35037 |  |
| 852 | | 0.42339 | | 0.39754 | | 0.38644 | | 0.35021 |  |
| 852.5 | | 0.42312 | | 0.39717 | | 0.38648 | | 0.35054 |  |
| 853 | | 0.42294 | | 0.39718 | | 0.38713 | | 0.35076 |  |
| 853.5 | | 0.42257 | | 0.3971 | | 0.38793 | | 0.35105 |  |
| 854 | | 0.42232 | | 0.39773 | | 0.38875 | | 0.35108 |  |
| 854.5 | | 0.42237 | | 0.39784 | | 0.38946 | | 0.35097 |  |
| 855 | | 0.42344 | | 0.39805 | | 0.39029 | | 0.35085 |  |
| 855.5 | | 0.4251 | | 0.39874 | | 0.39181 | | 0.35077 |  |
| 856 | | 0.42678 | | 0.39978 | | 0.39275 | | 0.35109 |  |
| 856.5 | | 0.42787 | | 0.40029 | | 0.39347 | | 0.35098 |  |
| 857 | | 0.42885 | | 0.40008 | | 0.39352 | | 0.35075 |  |
| 857.5 | | 0.43043 | | 0.3996 | | 0.39346 | | 0.35033 |  |
| 858 | | 0.43184 | | 0.39945 | | 0.39411 | | 0.35041 |  |
| 858.5 | | 0.43325 | | 0.39902 | | 0.39404 | | 0.3503 |  |
| 859 | | 0.43393 | | 0.39935 | | 0.39385 | | 0.35026 |  |
| 859.5 | | 0.43449 | | 0.39936 | | 0.39273 | | 0.35042 |  |
| 860 | | 0.43429 | | 0.39973 | | 0.39239 | | 0.35096 |  |
| 860.5 | | 0.43388 | | 0.39945 | | 0.39211 | | 0.35146 |  |
| 861 | | 0.43424 | | 0.39962 | | 0.39163 | | 0.35201 |  |
| 861.5 | | 0.43516 | | 0.39991 | | 0.39073 | | 0.35237 |  |
| 862 | | 0.43586 | | 0.40063 | | 0.38962 | | 0.35272 |  |
| 862.5 | | 0.43567 | | 0.40123 | | 0.38932 | | 0.35289 |  |
| 863 | | 0.43487 | | 0.40227 | | 0.38925 | | 0.35248 |  |
| 863.5 | | 0.43463 | | 0.40295 | | 0.38874 | | 0.35191 |  |
| 864 | | 0.43514 | | 0.40422 | | 0.38756 | | 0.35077 |  |
| 864.5 | | 0.43651 | | 0.40496 | | 0.38709 | | 0.34995 |  |
| 865 | | 0.43782 | | 0.40639 | | 0.38756 | | 0.34941 |  |
| 865.5 | | 0.43878 | | 0.40793 | | 0.38909 | | 0.34916 |  |
| 866 | | 0.43982 | | 0.409 | | 0.38955 | | 0.34931 |  |
| 866.5 | | 0.44087 | | 0.409 | | 0.39051 | | 0.34933 |  |
| 867 | | 0.44256 | | 0.40851 | | 0.3905 | | 0.34953 |  |
| 867.5 | | 0.44404 | | 0.40766 | | 0.39105 | | 0.34923 |  |
| 868 | | 0.44453 | | 0.40753 | | 0.39091 | | 0.34896 |  |
| 868.5 | | 0.4446 | | 0.40714 | | 0.39077 | | 0.349 |  |
| 869 | | 0.44379 | | 0.40766 | | 0.39122 | | 0.34993 |  |
| 869.5 | | 0.44313 | | 0.40762 | | 0.39196 | | 0.35113 |  |
| 870 | | 0.44208 | | 0.40817 | | 0.39313 | | 0.35256 |  |
| 870.5 | | 0.44209 | | 0.40816 | | 0.39335 | | 0.35305 |  |
| 871 | | 0.44306 | | 0.40909 | | 0.39314 | | 0.35359 |  |
| 871.5 | | 0.44363 | | 0.40971 | | 0.39265 | | 0.35331 |  |
| 872 | | 0.44324 | | 0.41155 | | 0.39253 | | 0.35363 |  |
| 872.5 | | 0.44248 | | 0.41265 | | 0.39246 | | 0.35369 |  |
| 873 | | 0.44288 | | 0.41386 | | 0.39274 | | 0.35419 |  |
| 873.5 | | 0.4448 | | 0.41388 | | 0.39218 | | 0.35459 |  |
| 874 | | 0.44689 | | 0.41404 | | 0.39236 | | 0.35354 |  |
| 874.5 | | 0.44864 | | 0.41382 | | 0.39354 | | 0.35185 |  |
| 875 | | 0.44973 | | 0.41377 | | 0.39535 | | 0.34924 |  |
| 875.5 | | 0.45108 | | 0.41374 | | 0.39678 | | 0.34772 |  |
| 876 | | 0.45232 | | 0.41382 | | 0.39738 | | 0.34655 |  |
| 876.5 | | 0.45264 | | 0.41407 | | 0.39882 | | 0.34541 |  |
| 877 | | 0.45302 | | 0.41376 | | 0.39905 | | 0.34371 |  |
| 877.5 | | 0.45442 | | 0.41335 | | 0.39986 | | 0.34244 |  |
| 878 | | 0.4562 | | 0.41308 | | 0.39968 | | 0.34166 |  |
| 878.5 | | 0.45836 | | 0.41321 | | 0.40129 | | 0.34222 |  |
| 879 | | 0.45799 | | 0.41376 | | 0.40246 | | 0.34313 |  |
| 879.5 | | 0.45739 | | 0.4139 | | 0.4047 | | 0.34418 |  |
| 880 | | 0.45535 | | 0.41454 | | 0.4059 | | 0.34409 |  |
| 880.5 | | 0.45587 | | 0.4147 | | 0.40695 | | 0.34367 |  |
| 881 | | 0.45627 | | 0.41559 | | 0.40779 | | 0.34477 |  |
| 881.5 | | 0.45686 | | 0.41661 | | 0.40903 | | 0.3466 |  |
| 882 | | 0.45594 | | 0.41839 | | 0.41015 | | 0.3487 |  |
| 882.5 | | 0.45527 | | 0.42008 | | 0.41019 | | 0.35017 |  |
| 883 | | 0.45458 | | 0.4205 | | 0.41063 | | 0.35211 |  |
| 883.5 | | 0.45392 | | 0.42009 | | 0.40989 | | 0.3545 |  |
| 884 | | 0.45427 | | 0.41992 | | 0.40902 | | 0.35617 |  |
| 884.5 | | 0.45458 | | 0.41979 | | 0.40623 | | 0.35833 |  |
| 885 | | 0.45418 | | 0.42001 | | 0.40333 | | 0.36011 |  |
| 885.5 | | 0.45274 | | 0.41977 | | 0.40072 | | 0.36222 |  |
| 886 | | 0.4513 | | 0.42003 | | 0.39859 | | 0.36146 |  |
| 886.5 | | 0.45075 | | 0.41919 | | 0.39607 | | 0.35881 |  |
| 887 | | 0.45196 | | 0.41789 | | 0.39241 | | 0.35545 |  |
| 887.5 | | 0.45447 | | 0.41712 | | 0.38993 | | 0.35323 |  |
| 888 | | 0.45672 | | 0.41652 | | 0.38738 | | 0.35144 |  |
| 888.5 | | 0.45999 | | 0.41618 | | 0.3865 | | 0.3501 |  |
| 889 | | 0.46057 | | 0.41566 | | 0.38586 | | 0.34795 |  |
| 889.5 | | 0.46175 | | 0.4148 | | 0.3871 | | 0.34548 |  |
| 890 | | 0.45993 | | 0.41413 | | 0.38771 | | 0.34117 |  |
| 890.5 | | 0.46085 | | 0.4125 | | 0.39025 | | 0.33754 |  |
| 891 | | 0.46083 | | 0.41322 | | 0.3936 | | 0.33473 |  |
| 891.5 | | 0.46148 | | 0.41399 | | 0.39771 | | 0.33302 |  |
| 892 | | 0.46163 | | 0.41385 | | 0.40022 | | 0.33256 |  |
| 892.5 | | 0.46238 | | 0.41226 | | 0.40328 | | 0.33231 |  |
| 893 | | 0.46338 | | 0.40889 | | 0.40633 | | 0.33249 |  |
| 893.5 | | 0.46426 | | 0.40846 | | 0.40812 | | 0.33351 |  |
| 894 | | 0.4653 | | 0.40917 | | 0.40777 | | 0.33596 |  |
| 894.5 | | 0.46417 | | 0.41036 | | 0.40725 | | 0.33952 |  |
| 895 | | 0.46169 | | 0.41082 | | 0.40765 | | 0.34176 |  |
| 895.5 | | 0.46224 | | 0.41194 | | 0.40839 | | 0.34228 |  |
| 896 | | 0.46296 | | 0.41341 | | 0.409 | | 0.34256 |  |
| 896.5 | | 0.46386 | | 0.41522 | | 0.40948 | | 0.3426 |  |
| 897 | | 0.46494 | | 0.41738 | | 0.40981 | | 0.34239 |  |
| 897.5 | | 0.46619 | | 0.41989 | | 0.41 | | 0.34195 |  |
| 898 | | 0.46762 | | 0.42274 | | 0.41005 | | 0.34126 |  |
| 898.5 | | 0.46922 | | 0.42594 | | 0.40996 | | 0.34034 |  |
| 899 | | 0.471 | | 0.42949 | | 0.40973 | | 0.33917 |  |
| 899.5 | | 0.47296 | | 0.43338 | | 0.40936 | | 0.33775 |  |
| 900 | | 0.47509 | | 0.43762 | | 0.40886 | | 0.3361 |  |

| **The average peak area (X) at 545nm and average color parameters of 7 categories(fig.3c)** | | | | | | |
| --- | --- | --- | --- | --- | --- | --- |
| categories | $\overline{X}$ | $\overline{L^{\text{*}}}$ | $\overline{a^{*}}$ | $\overline{b^{*}}$ | $\overline{C^{*}}$ | $\overline{h^{o}}$ |
| L_1_^*^(＜35) | 91.983 | 33.83 | 25.5 | -28.85 | 38.5 | 311.5 |
|  | 73.665 | 31.32 | 28.08 | -29.56 | 40.77 | 313.5 |
|  | 48.971 | 34.51 | 29.91 | -31.06 | 43.12 | 313.9 |
|  | 61.008 | 30.37 | 29.74 | -29.36 | 41.79 | 315.4 |
|  | 54.386 | 31.27 | 28.54 | -27.7 | 39.77 | 315.9 |
|  | 47.497 | 31.02 | 21.46 | -20.7 | 29.82 | 316 |
| MEAN VALUE | 66.0026 | 32.26 | 28.354 | -29.306 | 40.79 | 314.04 |
| L_2_^*^(35~40) | 62.118 | 36.33 | 24.81 | -25.17 | 35.34 | 314.6 |
|  | 71.041 | 39.77 | 34.28 | -31.5 | 46.56 | 317.4 |
|  | 75.807 | 36.22 | 27.76 | -28.93 | 40.09 | 313.8 |
|  | 72.561 | 39.11 | 34.08 | -31.65 | 46.51 | 317.1 |
|  | 88.918 | 38 | 33.5 | -32.89 | 46.95 | 315.5 |
|  | 55.309 | 38.78 | 20.56 | -17.85 | 27.23 | 319.0 |
|  | 42.877 | 39.37 | 16.95 | -20.05 | 26.25 | 310.2 |
|  | 67.706 | 38.27 | 32.07 | -28.05 | 42.60 | 318.8 |
|  | 33.362 | 36.53 | 20.2 | -20.78 | 28.98 | 314.2 |
|  | 60.462 | 35.74 | 29.76 | -29.79 | 42.11 | 315.0 |
| MEAN VALUE | 63.016 | 37.81 | 27.40 | -26.67 | 38.26 | 315.56 |
| L_3_^*^(40~45) | 57.599 | 42.81 | 32.9 | -28.99 | 43.84 | 318.6 |
|  | 71.802 | 41.39 | 30.05 | -31.99 | 43.89 | 313.2 |
|  | 70 | 40.14 | 33.47 | -31.27 | 45.8 | 316.9 |
|  | 58.485 | 43.67 | 29.92 | -27.62 | 40.72 | 317.3 |
|  | 43.816 | 44.77 | 19.12 | -20.25 | 27.85 | 313.4 |
|  | 50.544 | 40.17 | 20.21 | -20.74 | 28.96 | 314.2 |
|  | 61.533 | 43.33 | 29.75 | -28.19 | 40.99 | 316.5 |
|  | 68.724 | 41.37 | 31.46 | -28.99 | 42.78 | 317.3 |
| MEAN VALUE | 60.312875 | 42.20583333 | 28.35958333 | -27.25458333 | 39.3531933 | 315.9194583 |
| L_4_^*^(45~50) | 67.248 | 45.29 | 31.27 | -28.77 | 42.49 | 317.4 |
|  | 67.056 | 48.46 | 25.86 | -27.79 | 37.96 | 312.9 |
|  | 68.717 | 45.04 | 28.61 | -24.81 | 37.87 | 319.1 |
|  | 48.045 | 49.47 | 27.26 | -24.22 | 36.47 | 318.4 |
|  | 49.737 | 49.24 | 27.24 | -24.56 | 36.96 | 318.3 |
|  | 53.281 | 46.94 | 26.78 | -22.97 | 35.28 | 319.4 |
| MEAN VALUE | 59.014 | 47.40666667 | 27.83611111 | -25.52 | 37.83776245 | 317.5775905 |
| L_5_^*^(50~55) | 51.562 | 53.15 | 26.35 | -26.42 | 37.21 | 314.9 |
|  | 62.256 | 51.68 | 31.58 | -24.67 | 40.07 | 322.0 |
|  | 48.92 | 51.19 | 18.97 | -24.85 | 31.27 | 307.4 |
|  | 30.91 | 54.98 | 7.05 | -7.73 | 10.47 | 312.4 |
|  | 35.077 | 50.31 | 9.11 | -9.24 | 12.97 | 314.6 |
|  | 49.359 | 50.86 | 17.29 | -15.03 | 22.91 | 319.0 |
|  | 42.626 | 51.03 | 18.42 | -17.39 | 25.33 | 316.6 |
|  | 49.65 | 54.79 | 23.87 | -23.12 | 33.23 | 315.9 |
|  | 62.616 | 52.09 | 30.26 | -22.95 | 37.98 | 322.8 |
|  | 60.238 | 53.58 | 17.15 | -21.94 | 27.85 | 308.0 |
|  | 63.39 | 50.7 | 23.46 | -24.49 | 33.91 | 313.8 |
| MEAN VALUE | 50.60036364 | 52.21484848 | 20.31939394 | -19.8030303 | 28.4724366 | 315.2151793 |
| L_6_^*^(55~60) | 34.523 | 58.23 | 8.94 | -8.11 | 12.2 | 317.1 |
|  | 52.466 | 56.33 | 21.67 | -20.65 | 29.93 | 316.4 |
|  | 45.015 | 59.04 | 17.95 | -13.97 | 22.74 | 322.1 |
|  | 46.265 | 59.18 | 25.05 | -18.84 | 31.34 | 323.0 |
|  | 55.598 | 56.87 | 15.47 | -19.67 | 25.02 | 308.2 |
|  | 54.751 | 56.75 | 20.19 | -21.1 | 29.2 | 313.7 |
| MEAN VALUE | 48.103 | 57.73333333 | 18.21166667 | -17.05666667 | 25.07166667 | 316.75 |
| L_7_^*^(＞60) | 31.113 | 68.79 | 11.49 | -9.58 | 14.96 | 320.2 |
|  | 37 | 65.62 | 14.63 | -12.04 | 18.95 | 320.5 |
|  | 39.987 | 65.3 | 15.96 | -14.9 | 21.84 | 317.0 |
| MEAN VALUE | 36.03333333 | 66.57 | 14.02666667 | -12.17333333 | 18.58333333 | 319.2333333 |

**Data of Table 3**

| **Table 3.** Mean value of color parameters, R_a_, R_b_ and color difference (ΔE_00_) at different temperatures | | | | | | | | |
| --- | --- | --- | --- | --- | --- | --- | --- | --- |
| Temperatures(°C) | $\overline{L^{*}}$ | $\overline{a^{*}}$ | $\overline{b^{*}}$ | $\overline{C^{*}}$ | $\overline{h^{_{0}}}$ | Ra | R_b_ | ΔE_00_ |
| 20 | 39.25 (δ=8.50) | 30.59 (δ=4.59) | -29.51 (δ=2.86) | 42.50 | 316.02 | 19.18 | 8.99 | - |
| 300 | 39.76 (δ=7.99) | 29.30 (δ=4.25) | -28.78 (δ=2.45) | 41.07 | 315.51 | 17.23 | 8.57 | 0.72 |
| 320 | 39.96 (δ=8.07) | 29.33 (δ=4.29) | -28.73 (δ=2.55) | 41.06 | 315.59 | 17.83 | 8.79 | 0.18 |
| 340 | 38.77 (δ=8.54) | 28.81 (δ=4.33) | -28.03 (δ=2.86) | 40.20 | 315.78 | 18.78 | 9.15 | 1.09 |
| 360 | 39.30 (δ=8.27) | 27.73 (δ=4.90) | -26.73 (δ=3.25) | 38.51 | 316.05 | 21.60 | 15.37 | 0.77 |
| 380 | 43.59 (δ=7.11) | 24.96 (δ=5.11) | -23.80 (δ=3.31) | 34.49 | 316.36 | 17.29 | 12.96 | 4.15 |
| 400 | 55.53 (δ=7.77) | 16.82 (δ=6.69) | -15.59 (δ=6.02) | 22.93 | 317.17 | 25.51 | 21.22 | 12.99 |
| 420 | 70.49 (δ=8.69) | 4.07 (δ=7.56) | -1.72 (δ=8.21) | 4.42 | 337.11 | 26.92 | 28.53 | 18.22 |
| 440 | 76.52 (δ=8.03) | -0.62 (δ=3.27) | 3.63 (δ=4.26) | 3.68 | 99.76 | 12.46 | 15.98 | 9.09 |
| 460 | 78.16 (δ=7.85) | -2.17 (δ=2.47) | 6.37 (δ=3.83) | 6.73 | 108.79 | 10.60 | 14.87 | 3.15 |
| 480 | 78.94 (δ=7.48) | -3.16 (δ=1.70) | 10.32 (δ=4.36) | 10.79 | 107.01 | 6.86 | 13.00 | 3.03 |
| 500 | 78.81 (δ=7.25) | -2.93 (δ=1.17) | 13.26 (δ=4.96) | 13.58 | 102.44 | 4.97 | 14.97 | 2.01 |
| 520 | 78.79 (δ=6.84) | -2.69 (δ=1.04) | 14.79 (δ=4.85) | 15.03 | 100.32 | 4.14 | 14.98 | 1.03 |
| 540 | 79.11 (δ=6.48) | -2.34 (δ=0.88) | 16.09 (δ=5.30) | 16.25 | 98.27 | 3.08 | 17.02 | 0.95 |
| 560 | 78.90 (δ=6.53) | -1.74 (δ=0.83) | 17.17 (δ=5.50) | 17.26 | 95.79 | 2.68 | 18.25 | 0.98 |
| 580 | 76.15 (δ=6.91) | -0.60 (δ=0.94) | 19.29 (δ=5.94) | 19.30 | 91.79 | 3.47 | 20.32 | 2.65 |
| 600 | 76.15 (δ=6.50) | -0.08 (δ=0.84) | 19.77 (δ=6.09) | 19.77 | 90.23 | 3.28 | 18.79 | 0.63 |

## **Data of figure 5c ( The relationship between the degree of color change of amethyst and the initial color)**

| Temperature | C_1_^*^(30-40) | C_2_^*^(40-45) | C_3_^*^(45-55) |
| --- | --- | --- | --- |
| 280 | 0 | 0 | 0 |
| 300 | 0.46 | 1.488461538 | 2.135833333 |
| 320 | 0.65 | 1.388461538 | 2.173333333 |
| 340 | 0.78220467 | 2.242022667 | 3.601273739 |
| 360 | 3.474209149 | 3.655910473 | 4.756734767 |
| 380 | 6.149571587 | 8.688271788 | 8.561351225 |
| 400 | 15.93109404 | 20.64486134 | 21.54847416 |
| 420 | 27.48123095 | 33.0760006 | 38.96622343 |
| 440 | 30.69875 | 36.107 | 41.91875 |
| 460 | 28.43642899 | 36.03176106 | 39.56735972 |
| 480 | 25.97176747 | 32.14709677 | 35.19982921 |
| 500 | 23.40453517 | 29.29143228 | 32.43055196 |
| 520 | 21.72143753 | 27.89752247 | 31.22071565 |
| 540 | 20.44945927 | 26.67693614 | 30.04101502 |
| 560 | 19.68536572 | 25.56290157 | 28.9357787 |
| 580 | 19.23787378 | 24.63637094 | 28.27564671 |
| 600 | 19.02141518 | 24.71638502 | 27.61367492 |

| Temperature | L1*(20-35) | L_2_^*^(35-45) | L_3_^*^(45-55) |
| --- | --- | --- | --- |
| 20 | 0 | 0 | 0 |
| 300 | 1.2125 | 0.404615385 | -0.284444444 |
| 320 | 1.3275 | 0.679230769 | -0.102222222 |
| 340 | -0.619722222 | -0.151538462 | -0.771111111 |
| 360 | 0.135694444 | 0.47025641 | -0.675555556 |
| 380 | 5.770833333 | 5.042051282 | 1.381111111 |
| 400 | 19.12055556 | 18.38846154 | 9.426666667 |
| 420 | 35.26888889 | 32.26076923 | 24.35777778 |
| 440 | 40.76571429 | 36.22363636 | 33.17 |
| 460 | 41.31904762 | 38.63787879 | 34.6975 |
| 480 | 42.41333333 | 39.44969697 | 35.16083333 |
| 500 | 41.76571429 | 39.74151515 | 34.91166667 |
| 520 | 41.88571429 | 40.18454545 | 34.12791667 |
| 540 | 42.40142857 | 40.81727273 | 33.86333333 |
| 560 | 42.34380952 | 40.24348485 | 34.00041667 |
| 580 | 40.32466667 | 39.09333333 | 34.335 |
| 600 | 40.312 | 38.73333333 | 33.88666667 |

| **Color Grading of heat-treated amethyst (Data of figure 7)** | | | | | | |
| --- | --- | --- | --- | --- | --- | --- |
| Temperature | L^*^ | a^*^ | b^*^ | c^*^ | h^*^ | Classification |
| 460 | 81.23 | -5.99 | 9.89 | 11.56 | 121.19 | 5 |
| 460 | 81.64 | -5.92 | 9.71 | 11.38 | 121.38 | 5 |
| 460 | 75.73 | -6.20 | 11.40 | 12.97 | 118.55 | 5 |
| 460 | 75.72 | -6.03 | 11.27 | 12.78 | 118.16 | 5 |
| 460 | 83.74 | -3.20 | 6.28 | 7.05 | 116.99 | 4 |
| 460 | 84.03 | -3.03 | 5.91 | 6.64 | 117.12 | 4 |
| 460 | 66.25 | -1.06 | 3.92 | 4.06 | 105.07 | 1 |
| 460 | 65.01 | -0.65 | 3.85 | 3.90 | 99.59 | 1 |
| 460 | 71.71 | -2.26 | 8.55 | 8.84 | 104.83 | 1 |
| 460 | 72.00 | -2.56 | 9.12 | 9.47 | 105.68 | 1 |
| 460 | 81.48 | -0.60 | 3.62 | 3.67 | 99.35 | 4 |
| 460 | 82.76 | -0.74 | 3.27 | 3.35 | 102.81 | 4 |
| 460 | 79.26 | -1.93 | 8.83 | 9.03 | 102.31 | 5 |
| 460 | 79.35 | -1.87 | 8.79 | 8.98 | 101.99 | 5 |
| 460 | 85.80 | -1.90 | 4.84 | 5.20 | 111.45 | 4 |
| 460 | 85.89 | -1.94 | 4.84 | 5.21 | 111.82 | 4 |
| 460 | 85.90 | -0.36 | 2.40 | 2.43 | 98.60 | 4 |
| 460 | 86.30 | -0.45 | 2.44 | 2.48 | 100.39 | 4 |
| 460 | 86.17 | -3.24 | 7.73 | 8.39 | 112.75 | 4 |
| 460 | 85.78 | -3.30 | 8.01 | 8.66 | 112.36 | 4 |
| 460 | 63.56 | -3.43 | 12.21 | 12.69 | 105.69 | 2 |
| 460 | 64.47 | -3.65 | 13.72 | 14.19 | 104.90 | 2 |
| 460 | 86.49 | -2.00 | 3.62 | 4.14 | 118.90 | 4 |
| 460 | 86.57 | -1.95 | 3.55 | 4.05 | 118.80 | 4 |
| 460 | 66.14 | 4.40 | -1.15 | 4.55 | 345.35 | 1 |
| 460 | 69.05 | 3.52 | -1.03 | 3.67 | 343.64 | 1 |
| 480 | 80.45 | -5.50 | 14.45 | 15.46 | 110.83 | 5 |
| 480 | 79.87 | -5.41 | 14.42 | 15.40 | 110.56 | 5 |
| 480 | 75.92 | -5.46 | 16.46 | 17.34 | 108.36 | 5 |
| 480 | 76.89 | -5.35 | 15.67 | 16.56 | 108.84 | 5 |
| 480 | 84.17 | -3.94 | 9.33 | 10.13 | 112.87 | 4 |
| 480 | 83.45 | -4.10 | 9.88 | 10.70 | 112.56 | 4 |
| 480 | 70.78 | -2.82 | 11.05 | 11.40 | 104.30 | 1 |
| 480 | 71.06 | -3.05 | 11.86 | 12.25 | 104.44 | 1 |
| 480 | 73.23 | -5.10 | 15.94 | 16.73 | 107.75 | 2 |
| 480 | 73.25 | -5.30 | 16.63 | 17.45 | 107.69 | 2 |
| 480 | 84.02 | -2.43 | 8.16 | 8.52 | 106.56 | 4 |
| 480 | 84.25 | -2.50 | 8.17 | 8.55 | 107.01 | 4 |
| 480 | 79.54 | -3.08 | 12.11 | 12.49 | 104.29 | 5 |
| 480 | 79.11 | -3.10 | 12.40 | 12.78 | 104.03 | 5 |
| 480 | 86.29 | -2.20 | 7.83 | 8.13 | 105.72 | 4 |
| 480 | 86.65 | -2.23 | 7.94 | 8.25 | 105.70 | 4 |
| 480 | 87.83 | -2.14 | 3.98 | 4.51 | 118.25 | 4 |
| 480 | 87.88 | -2.05 | 4.00 | 4.50 | 117.15 | 4 |
| 480 | 84.81 | -3.43 | 9.65 | 10.24 | 109.55 | 4 |
| 480 | 84.84 | -3.26 | 9.53 | 10.08 | 108.88 | 4 |
| 480 | 62.89 | -3.28 | 14.95 | 15.30 | 102.38 | 2 |
| 480 | 62.62 | -3.54 | 16.75 | 17.12 | 101.94 | 2 |
| 480 | 87.09 | -2.68 | 4.65 | 5.37 | 120.01 | 4 |
| 480 | 87.24 | -2.61 | 4.70 | 5.38 | 119.06 | 4 |
| 480 | 67.70 | 1.10 | 3.74 | 3.90 | 73.67 | 1 |
| 480 | 70.53 | 1.36 | 4.11 | 4.33 | 71.68 | 1 |
| 500 | 79.49 | -1.37 | 19.91 | 19.96 | 93.95 | 3 |
| 500 | 80.08 | -4.47 | 19.03 | 19.55 | 103.21 | 5 |
| 500 | 74.93 | -4.44 | 20.83 | 21.30 | 102.04 | 3 |
| 500 | 75.95 | -4.41 | 19.90 | 20.38 | 102.49 | 3 |
| 500 | 83.28 | -4.01 | 12.71 | 13.32 | 107.51 | 5 |
| 500 | 83.94 | -3.84 | 12.07 | 12.67 | 107.66 | 5 |
| 500 | 70.89 | -2.44 | 13.90 | 14.11 | 99.94 | 2 |
| 500 | 69.95 | -2.73 | 16.10 | 16.33 | 99.61 | 2 |
| 500 | 72.30 | -5.18 | 20.62 | 21.26 | 104.11 | 3 |
| 500 | 72.19 | -4.77 | 19.32 | 19.90 | 103.87 | 3 |
| 500 | 83.41 | -2.51 | 11.58 | 11.85 | 102.25 | 5 |
| 500 | 85.07 | -2.54 | 10.84 | 11.13 | 103.19 | 4 |
| 500 | 78.03 | -3.17 | 15.02 | 15.35 | 101.91 | 5 |
| 500 | 78.64 | -3.19 | 14.84 | 15.18 | 102.14 | 5 |
| 500 | 86.26 | -2.13 | 10.33 | 10.55 | 101.65 | 4 |
| 500 | 86.50 | -2.16 | 10.41 | 10.63 | 101.74 | 4 |
| 500 | 87.74 | -2.26 | 5.86 | 6.28 | 111.09 | 4 |
| 500 | 87.96 | -2.20 | 6.02 | 6.41 | 110.06 | 4 |
| 500 | 83.78 | -3.03 | 11.91 | 12.29 | 104.28 | 5 |
| 500 | 84.08 | -2.99 | 11.80 | 12.17 | 104.24 | 5 |
| 500 | 62.53 | -2.84 | 16.34 | 16.59 | 99.87 | 2 |
| 500 | 63.49 | -3.16 | 18.98 | 19.24 | 99.44 | 2 |
| 500 | 87.00 | -2.77 | 6.35 | 6.93 | 113.53 | 4 |
| 500 | 87.32 | -2.65 | 6.23 | 6.77 | 113.03 | 4 |
| 500 | 72.92 | -0.21 | 7.01 | 7.02 | 91.74 | 1 |
| 500 | 71.33 | -0.57 | 6.92 | 6.94 | 94.68 | 1 |
| 520 | 79.77 | -3.73 | 19.68 | 20.02 | 100.72 | 3 |
| 520 | 79.92 | -3.95 | 20.94 | 21.31 | 100.68 | 3 |
| 520 | 75.37 | -3.79 | 22.27 | 22.59 | 99.66 | 3 |
| 520 | 75.64 | -3.76 | 21.67 | 22.00 | 99.85 | 3 |
| 520 | 83.43 | -3.72 | 13.49 | 13.99 | 105.42 | 5 |
| 520 | 82.81 | -3.78 | 13.86 | 14.37 | 105.26 | 5 |
| 520 | 70.47 | -2.03 | 14.96 | 15.09 | 97.73 | 2 |
| 520 | 70.70 | -2.40 | 16.69 | 16.86 | 98.19 | 2 |
| 520 | 72.15 | -4.46 | 20.91 | 21.38 | 102.03 | 3 |
| 520 | 71.88 | -4.87 | 22.55 | 23.07 | 102.18 | 3 |
| 520 | 83.61 | -2.29 | 13.03 | 13.23 | 99.96 | 5 |
| 520 | 85.38 | -2.23 | 12.45 | 12.64 | 100.16 | 4 |
| 520 | 77.18 | -3.05 | 16.31 | 16.59 | 100.58 | 5 |
| 520 | 77.65 | -3.01 | 16.11 | 16.39 | 100.59 | 5 |
| 520 | 85.80 | -1.94 | 11.83 | 11.99 | 99.33 | 4 |
| 520 | 85.54 | -1.87 | 11.71 | 11.86 | 99.09 | 4 |
| 520 | 87.45 | -0.72 | 7.57 | 7.61 | 95.46 | 4 |
| 520 | 87.55 | -2.01 | 7.59 | 7.85 | 104.82 | 4 |
| 520 | 81.95 | -1.88 | 14.83 | 14.95 | 97.22 | 5 |
| 520 | 82.12 | -1.95 | 15.04 | 15.17 | 97.37 | 5 |
| 520 | 62.28 | -2.73 | 18.24 | 18.44 | 98.50 | 2 |
| 520 | 64.15 | -2.82 | 19.77 | 19.97 | 98.13 | 2 |
| 520 | 86.99 | -2.37 | 8.14 | 8.47 | 106.24 | 4 |
| 520 | 86.96 | -2.39 | 8.19 | 8.53 | 106.27 | 4 |
| 520 | 76.10 | -1.06 | 8.45 | 8.52 | 97.15 | 1 |
| 520 | 75.64 | -1.22 | 8.21 | 8.30 | 98.43 | 1 |
| 540 | 79.36 | -3.33 | 22.17 | 22.42 | 98.54 | 3 |
| 540 | 79.72 | -3.37 | 22.23 | 22.49 | 98.61 | 3 |
| 540 | 76.15 | -2.69 | 24.41 | 24.56 | 96.28 | 3 |
| 540 | 75.69 | -2.56 | 25.60 | 25.72 | 95.72 | 3 |
| 540 | 82.83 | -3.64 | 14.97 | 15.40 | 103.67 | 5 |
| 540 | 83.49 | -3.48 | 14.31 | 14.73 | 103.68 | 5 |
| 540 | 71.99 | -1.64 | 15.76 | 15.85 | 95.94 | 2 |
| 540 | 72.48 | -2.00 | 17.40 | 17.51 | 96.55 | 2 |
| 540 | 72.20 | -4.26 | 24.18 | 24.55 | 99.99 | 3 |
| 540 | 72.41 | -4.08 | 22.89 | 23.25 | 100.11 | 3 |
| 540 | 83.06 | -1.70 | 14.15 | 14.25 | 96.86 | 5 |
| 540 | 84.61 | -1.81 | 13.20 | 13.33 | 97.81 | 5 |
| 540 | 78.07 | -2.78 | 17.52 | 17.74 | 99.01 | 5 |
| 540 | 77.95 | -2.70 | 17.36 | 17.57 | 98.84 | 5 |
| 540 | 85.86 | -1.68 | 12.16 | 12.27 | 97.85 | 4 |
| 540 | 86.54 | -1.75 | 11.91 | 12.03 | 98.35 | 4 |
| 540 | 86.60 | -1.70 | 9.15 | 9.31 | 100.54 | 4 |
| 540 | 87.35 | -1.77 | 9.16 | 9.33 | 100.94 | 4 |
| 540 | 80.87 | -1.28 | 17.12 | 17.16 | 94.29 | 5 |
| 540 | 81.31 | -1.19 | 16.77 | 16.82 | 94.07 | 5 |
| 540 | 62.91 | -2.23 | 18.77 | 18.90 | 96.79 | 2 |
| 540 | 63.94 | -2.49 | 20.92 | 21.07 | 96.80 | 2 |
| 540 | 86.77 | -2.15 | 9.37 | 9.61 | 102.92 | 4 |
| 540 | 86.94 | -2.12 | 9.45 | 9.68 | 102.67 | 4 |
| 540 | 78.92 | -1.23 | 8.72 | 8.80 | 98.01 | 5 |
| 540 | 78.90 | -1.18 | 8.58 | 8.66 | 97.86 | 5 |
| 560 | 80.01 | -3.07 | 23.29 | 23.49 | 97.50 | 3 |
| 560 | 79.63 | -2.92 | 22.54 | 22.72 | 97.37 | 3 |
| 560 | 76.18 | -1.75 | 26.17 | 26.23 | 93.83 | 3 |
| 560 | 75.67 | -1.65 | 27.17 | 27.22 | 93.47 | 3 |
| 560 | 82.97 | -3.16 | 15.79 | 16.10 | 101.31 | 5 |
| 560 | 83.32 | -3.18 | 15.43 | 15.75 | 101.65 | 5 |
| 560 | 71.84 | -1.06 | 16.83 | 16.86 | 93.59 | 2 |
| 560 | 72.75 | -1.29 | 18.21 | 18.26 | 94.05 | 2 |
| 560 | 71.91 | -3.47 | 26.18 | 26.41 | 97.54 | 3 |
| 560 | 72.39 | -3.23 | 24.47 | 24.68 | 97.53 | 3 |
| 560 | 82.52 | -1.33 | 14.89 | 14.95 | 95.09 | 5 |
| 560 | 84.16 | -1.42 | 14.28 | 14.35 | 95.69 | 5 |
| 560 | 77.68 | -1.63 | 18.72 | 18.79 | 94.98 | 3 |
| 560 | 76.94 | -1.60 | 19.03 | 19.10 | 94.80 | 3 |
| 560 | 86.00 | -1.32 | 12.26 | 12.33 | 96.15 | 4 |
| 560 | 86.79 | -1.46 | 12.35 | 12.44 | 96.73 | 4 |
| 560 | 86.78 | -1.35 | 10.54 | 10.63 | 97.28 | 4 |
| 560 | 86.83 | -1.42 | 10.83 | 10.92 | 97.47 | 4 |
| 560 | 81.87 | -1.03 | 17.64 | 17.67 | 93.35 | 5 |
| 560 | 82.06 | -1.02 | 17.77 | 17.80 | 93.27 | 5 |
| 560 | 62.99 | -0.91 | 19.85 | 19.87 | 92.63 | 2 |
| 560 | 63.49 | -0.87 | 22.40 | 22.41 | 92.22 | 2 |
| 560 | 86.25 | -1.75 | 11.08 | 11.22 | 98.99 | 4 |
| 560 | 86.75 | -1.75 | 10.78 | 10.92 | 99.22 | 4 |
| 560 | 77.11 | -0.88 | 8.99 | 9.04 | 95.61 | 5 |
| 560 | 76.42 | -0.78 | 8.92 | 8.96 | 95.02 | 1 |
| 580 | 78.87 | -1.64 | 25.43 | 25.48 | 93.69 | 3 |
| 580 | 77.78 | -1.45 | 25.24 | 25.28 | 93.28 | 3 |
| 580 | 73.83 | 0.23 | 29.59 | 29.59 | 89.56 | 3 |
| 580 | 74.54 | -0.03 | 28.61 | 28.61 | 90.07 | 3 |
| 580 | 80.84 | -1.89 | 18.70 | 18.80 | 95.78 | 5 |
| 580 | 81.55 | -1.87 | 17.85 | 17.95 | 95.98 | 5 |
| 580 | 70.81 | -0.16 | 17.92 | 17.92 | 90.52 | 2 |
| 580 | 69.59 | -0.35 | 20.09 | 20.10 | 90.99 | 2 |
| 580 | 81.94 | -1.09 | 15.79 | 15.83 | 93.96 | 5 |
| 580 | 82.99 | -1.20 | 15.26 | 15.31 | 94.50 | 5 |
| 580 | 75.41 | -0.16 | 21.43 | 21.43 | 90.44 | 3 |
| 580 | 74.91 | -0.11 | 21.86 | 21.86 | 90.28 | 3 |
| 580 | 86.04 | -1.28 | 12.13 | 12.20 | 96.02 | 4 |
| 580 | 85.57 | -1.21 | 12.18 | 12.24 | 95.69 | 4 |
| 580 | 60.85 | 1.10 | 21.67 | 21.70 | 87.09 | 2 |
| 580 | 60.27 | 1.58 | 24.39 | 24.44 | 86.29 | 2 |
| 580 | 75.38 | -0.66 | 9.27 | 9.29 | 94.07 | 1 |
| 580 | 79.48 | -0.66 | 9.78 | 9.80 | 93.88 | 5 |
| 600 | 77.78 | 0.15 | 27.26 | 27.26 | 89.68 | 3 |
| 600 | 77.90 | 0.06 | 27.93 | 27.93 | 89.87 | 3 |
| 600 | 74.77 | 0.20 | 28.11 | 28.11 | 89.60 | 3 |
| 600 | 75.98 | 0.18 | 27.80 | 27.80 | 89.64 | 3 |
| 600 | 79.83 | -0.59 | 20.63 | 20.64 | 91.65 | 3 |
| 600 | 79.40 | -0.38 | 21.73 | 21.73 | 90.99 | 3 |
| 600 | 69.45 | 0.41 | 17.95 | 17.95 | 88.68 | 2 |
| 600 | 68.89 | 0.34 | 20.38 | 20.38 | 89.05 | 2 |
| 600 | 81.60 | -0.93 | 15.23 | 15.26 | 93.49 | 5 |
| 600 | 83.87 | -1.09 | 14.50 | 14.54 | 94.31 | 5 |
| 600 | 74.60 | 0.16 | 21.55 | 21.55 | 89.58 | 3 |
| 600 | 73.81 | 0.24 | 21.91 | 21.91 | 89.37 | 3 |
| 600 | 85.29 | -1.24 | 12.87 | 12.93 | 95.52 | 4 |
| 600 | 85.42 | -1.19 | 12.29 | 12.35 | 95.51 | 4 |
| 600 | 63.08 | 1.39 | 21.83 | 21.88 | 86.35 | 2 |
| 600 | 61.29 | 2.04 | 24.92 | 25.01 | 85.32 | 2 |
| 600 | 78.04 | -0.56 | 9.32 | 9.34 | 93.42 | 5 |
| 600 | 79.69 | -0.62 | 9.57 | 9.59 | 93.71 | 5 |

|  | Classification results | | | | | | |
| --- | --- | --- | --- | --- | --- | --- | --- |
|  |  |  |  |  |  |  | total |
|  |  | 1 | 2 | 3 | 4 | 5 |  |
| Count | 1 | 16 | 0 | 0 | 0 | 0 | 16 |
|  | 2 | 0 | 28 | 2 | 0 | 0 | 30 |
|  | 3 | 0 | 0 | 39 | 0 | 0 | 39 |
|  | 4 | 0 | 0 | 0 | 54 | 0 | 54 |
|  | 5 | 1 | 0 | 0 | 0 | 52 | 53 |
| % | 1 | 100 | 0 | 0 | 0 | 0 | 100 |
|  | 2 | 0 | 93.3 | 6.7 | 0 | 0 | 100 |
|  | 3 | 0 | 0 | 100 | 0 | 0 | 100 |
|  | 4 | 0 | 0 | 0 | 100 | 0 | 100 |
|  | 5 | 1.9 | 0 | 0 | 0 | 98.1 | 100 |
| 98.4% of the original group observations were classified correctly. | | | | | | | |

| Table Ⅳ. ANOVA of L*, a*, and b* of citrine color zones | | | | | | |
| --- | --- | --- | --- | --- | --- | --- |
|  | Clustering |  | Error |  | F | sig |
|  | Mean square | df | Mean square | Df |  |  |
| L^*^ | 2151.393 | 4 | 7.851 | 187 | 274.044 | 0 |
| a^*^ | 18.327 | 4 | 2.487 | 187 | 7.368 | 0 |
| b^*^ | 1634.685 | 4 | 9.475 | 187 | 172.525 | 0 |

| Fisher discriminant accuracy | | | | | |
| --- | --- | --- | --- | --- | --- |
|  | 1 | 2 | 3 | 4 | 5 |
| L* | 9.087 | 8.561 | 9.765 | 10.991 | 10.345 |
| a* | -0.316 | -1.116 | -1.446 | -1.035 | -1.437 |
| b* | 0.922 | 2.191 | 2.777 | 1.204 | 1.792 |
| constant | -327.807 | -307.956 | -406.711 | -480.334 | -433.888 |
| Fisher discriminant function | | | |  |  |
| F1=0.987L*-0.326a*+0.922b*-327.807 | | | |  |  |
| F2=8.561L*-1.116a*+2.191b*-307.956 | | | |  |  |
| F3=9.765L*-1.446a*+2.777b*-406.711 | | | |  |  |
| F4=10.991L*-1.035a*+1.204b*-480.334 | | | |  |  |
| F5=10.345L*-1.437a*+1.792b*-433.888 | | | |  |  |
